# The long non-coding RNA *DKFZp434J0226* regulates the alternative splicing process through phosphorylation of SF3B6 in PDAC

**DOI:** 10.1186/s10020-021-00347-7

**Published:** 2021-08-28

**Authors:** Jinglei Li, Hanxing Tong, Dongping Li, Qiuyu Jiang, Yong Zhang, Wenqing Tang, Dayong Jin, She Chen, Xinyu Qin, Si Zhang, Ruyi Xue

**Affiliations:** 1 Department of General Surgery, Zhongshan Hospital, Fudan University, 180 FengLin Road, Shanghai, 200032 China; 2 Department of Gastroenterology and Hepatology, Zhongshan Hospital, Shanghai Institute of Liver Disease, Fudan University, 180 FengLin Road, Shanghai, 200032 China; 3 NHC Key Laboratory of Glycoconjugate Research, Department of Biochemistry and Molecular Biology, School of Basic Medical Sciences, Shanghai Medical College, Fudan University, 130 DongAn Road, Shanghai, 200032 China

**Keywords:** DKFZp434J0226, LncRNA expression signature, Microarray, Pancreatic ductal adenocarcinoma

## Abstract

**Background:**

Long noncoding RNAs (lncRNAs), a type of pervasive genes that regulates various biological processes, are differentially expressed in different types of malignant tumors. The role of lncRNAs in the carcinogenesis of pancreatic ductal adenocarcinoma (PDAC) remains unclear. Here, we investigated the role of the lncRNA *DKFZp434J0226* in PDAC.

**Methods:**

Aberrantly expressed mRNAs and lncRNAs among six PDAC and paired non-tumorous tissues were profiled using microarray analysis. Quantitative real-time polymerase chain reaction was used to evaluate *DKFZp434J0226* expression in PDAC tissues. CCK-8 assay, wound-healing assay, soft agar colony formation assay, and transwell assay were performed to assess the invasiveness and proliferation of PDAC cells. Furthermore, RNA pull-down, immunofluorescence, RNA immunoprecipitation, and western blotting assays were performed to investigate the association between *DKFZp434J0226* and SF3B6. Tumor xenografts in mice were used to test for tumor formation in vivo.

**Results:**

In our study, 222 mRNAs and 128 lncRNAs were aberrantly expressed (≥ twofold change). Of these, 66 mRNAs and 53 lncRNAs were upregulated, while 75 lncRNAs and 156 mRNAs were downregulated. KEGG pathway analysis and the Gene ontology category indicated that these genes were associated with the regulation of mRNA alternative splicing and metabolic balance. Clinical analyses revealed that overexpression of *DKFZp434J0226* was associated with worse tumor grading, frequent perineural invasion, advanced tumor-node-metastasis stage, and decreased overall survival and time to progression. Functional assays demonstrated that *DKFZp434J0226* promoted PDAC cell migration, invasion, and growth in vitro and accelerated tumor proliferation in vivo. Mechanistically, *DKFZp434J0226* interacted with the splicing factor SF3B6 and promoted its phosphorylation, which further regulated the alternative splicing of pre-mRNA.

**Conclusions:**

This study indicates that DKFZp434J0226 regulates alternative splicing through phosphorylation of SF3B6 in PDAC and leads to an oncogenic phenotype in PDAC.

**Supplementary Information:**

The online version contains supplementary material available at 10.1186/s10020-021-00347-7.

## Introduction

Pancreatic cancer is considered a rare type of cancer, with an estimated 57,600 newly diagnosed patients in 2020 in the United States according to the American Cancer Society, accounting for 3.2% (57,600 of 18,06,590) of all cancer cases (Siegel et al. 2020). Although the incidence rate of most cancers has been decreasing recently, the incidence and mortality rates of pancreatic cancer are gradually increasing (Simard et al. 2014). As the most common pancreatic malignant tumor, pancreatic ductal adenocarcinoma (PDAC) accounts for more than 85% of pancreatic cancer cases (Li et al. 2004). After diagnosis, only approximately 35% of the patients survive for > 5 years (Li et al. 2004). Surgical resection is the only available treatment for PDAC. However, only less than 20% of tumors can be surgically removed at diagnosis. In addition, these patients usually respond poorly to chemotherapy. Thus, it is necessary to determine the molecular mechanisms underlying PDAC development.

A recent study has reported that epigenetic alterations influence the regulation of gene function in pancreatic cancers (Omura et al. 2009). Non-coding RNAs (ncRNAs), DNA methylation, and histone modifications are the main causes of epigenetic dysregulation. Most mammalian transcriptome is composed of abundant ncRNAs (Ponting et al. 2009), which can be generally divided into long ncRNAs (lncRNAs; 200 nt to > 100 kb) and small (18–200 nt). Furthermore, lncRNAs have complex biological functions in multiple processes (Wang et al. 2011). Increasing evidence has shown that lncRNAs have a vital effect on oncogenesis in human cancers (Ponting et al. 2009). Aberrant expression of lncRNAs in different types of human malignant tumors has been widely documented (Wapinski et al. 2011; Gibb et al. 2011), promoting a common interest in the use of therapeutic targets and biomarkers such as MALAT-1 in non-small cell lung carcinoma, HOTAIR in breast cancer, and HEIH in hepatocarcinoma (Schmidt et al. 2011; Gupta et al. 2010; Yang et al. 2011).

Studies have reported that ncRNAs may regulate the process of precursor mRNA (pre-mRNA) splicing. Hu et al. (2016) discovered that ncRNAs can regulate gene expression through h5S-OT lncRNA during transcription and pre-mRNA splicing. They identified the splicing regulator U2AF65 as a cofactor of the h5S-OT-dependent alternative splicing pathway. U2AF65 is a core splicing regulator required for the binding of U2 snRNP to the pre-mRNA branch site and is essential for splicing machinery and intron excision. In addition, Tripathi et al. (2010) reported that the lncRNA MALAT1 can influence the distribution of splicing factors in nuclear speckles. In addition, they reported that depletion of MALAT1 can change the splicing pattern of similar endogenous pre-mRNAs by regulating the phosphorylated forms of splicing factors (Tripathi et al. 2010), indicating that phosphorylation of splicing factors may be an essential process in the activation of the spliceosome.

Alternative splicing of pre-mRNAs contributes to the diversification of human transcripts and gene functions. Changes in the phosphorylation of splicing factors can regulate alternative splicing patterns in pre-mRNAs. The regulation of alternative splicing patterns is generally considered to be associated with tumor progression, metastatic dissemination, and survival in patients with PDAC. However, the molecular mechanism underlying the activation and regulation of splicing factors in PDAC remains unclear.

In the present study, we detected lncRNA expression patterns in six pairs of PDAC tumor tissue samples and matched non-tumor tissue samples by microarray and in 109 pairs of PDAC tumor samples and non-tumor tissue samples by quantitative real-time polymerase chain reaction (RT-qPCR). We identified *DKFZp434J0226* as a potential therapeutic target for PDAC. In addition, *DKFZp434J0226* was found to contribute to the phosphorylation of the splicing factor SF3B6, which further regulates the alternative splicing of pre-mRNA.

## Materials and methods

### Ethics statement

Ethical approval for human subjects was obtained from the research ethics committee of Zhongshan Hospital (Y2015-057), and written informed consent was obtained from each patient.

### Patient samples and cell lines

Six paired tumor samples and non-tumor samples from patients with PDAC were randomly obtained from six patients who underwent surgical resection at Zhongshan Hospital, Fudan University, in 2016. Paired PDAC tumor samples and adjacent liver tissue samples were collected from 61 patients (Zhongshan Hospital, 2015–2019) for RT-qPCR analysis. For the clinical significance study, PDAC tissues were collected from 109 patients (Zhongshan Hospital, 2015–2019). The pancreatic cancer cell lines AsPC-1, SW1990, MIAPaCa-2, CFPAC-1, Capan-1, and PANC-1 were purchased from the Cell Bank of Type Culture Collection of the Chinese Academy of Sciences (Shanghai Institute of Cell Biology, Chinese Academy of Sciences) and cultured in Dulbecco’s modified Eagle’s medium (DMEM; Invitrogen, CA, USA) supplemented with 10 U/mL penicillin G (Gibco, MA, USA), 10 U/mL streptomycin, and 10% fetal calf serum (Gibco, MA, USA). The cells were incubated at 37 °C with 5% CO_2_.

### RNA extraction

Total RNA was extracted from pancreatic cancer cells, snap-frozen PDAC samples, and matched normal non-tumor tissue samples using TRIzol reagent (Invitrogen) according to the manufacturer’s instructions. RNA quality and quantification assurance were assessed by NanoDrop ND-1000, gDNA contamination and RNA integrity test were assessed by denaturing agarose gel electrophoresis.

### Microarray and computational analysis

Samples (six PDAC tissues and six matched non-tumor tissues) were used to synthesize double-stranded cDNA, which was then labeled and hybridized to the Human LncRNA Array v2.0 (8 × 60 K, Arraystar, MD, USA). A total of 30,215 coding transcripts and 33,045 lncRNAs were collected from databases such as the Ensembl, UCSC Knowngenes, and RefSEq. After hybridization and washing, the processed array slides were scanned and analyzed using Agilent Scanner G2505C and Agilent Feature Extraction software (version 10.7.3.1), respectively. Subsequent data processing and quantile normalization were performed using the GeneSpring GX v11.5.1 software package (Agilent Technologies, CA, USA).

LncRNAs and mRNAs that were flagged in all six samples as Present or Marginal (“All Targets Value”) were chosen for further data analysis after quantile normalization of the raw data. Differentially expressed mRNAs and lncRNAs with statistical significance within the two groups were identified by volcano plot filtering. Gene ontology (GO) analysis and pathway analysis were performed to explore the roles of aberrantly expressed mRNAs in GO terms or biological pathways. The expression patterns of lncRNA and mRNA samples were examined by hierarchical clustering.

### Construction of the coding–non-coding gene co-expression network

The coding–non-coding gene co-expression (CNC) network construction procedures included the following: (i) preprocessing data: taking the median value of the same coding gene with different transcripts to represent gene expression values without special treatment of lncRNA expression value, (ii) screen data: removing the subset of data according to the lists that show the differential expression of lncRNA and mRNA, (iii) calculating the Pearson correlation coefficient and using the R value to calculate the correlation coefficient of PCC between lncRNA coding genes, and (iv) screening by the Pearson correlation coefficient and selecting the part for which PCC ≥ 0.90 is considered meaningful and draw the CNC network using Cytoscape.

### RT-qPCR

LncRNA and mRNA expression in PDAC tissues and cells was examined by RT-qPCR using SYBR Premix Ex Taq (Takara, Kusatsu, Japan) on an Eppendorf instrument. Additional file 1: Table S1 shows the primers used in this study. All experiments were performed in triplicates. All samples were normalized to GAPDH. Significance was examined by obtaining the average of the GAPDH-normalized 2^−ΔΔCt^ values.

### LncRNA in situ hybridization

A biotin-labeled antisense *DKFZp434J0226* probe (ATGTTCAGAGAAGACTGGTT) was synthesized using EXIQON. The paraffin-embedded tissues were treated with a peroxidase-quenching solution and incubated with a biotin-labeled probe. Then, streptavidin–horseradish peroxidase was reacted with the bound biotin-labeled probe. A TSA amplification kit (Perkin Elmer, Waltham, USA) was used to amplify the signal.

### RNA pull-down

We performed an RNA pull-down assay as previously described (Zhang et al. 2017). Briefly, biotin-labeled RNA was synthesized using in vitro transcription, in which T7 RNA polymerase was used with biotin-UTP. A forward primer (TAATACGACTCACTATAGGG) involving T7 RNA polymerase promoter and a reverse primer (GATTTAGGTGACACTATAG) involving the T3 RNA polymerase promoter were used to amplify the PCR fragments. In vitro transcription was performed using PCR products as DNA templates. Biotinylated RNA probes (approximately 20 pmol) were incubated with cell lysates for 30 min. Streptavidin Sepharose High-Performance beads (GE Healthcare, PA, USA) were used to isolate RNA-protein complexes. Liquid chromatography–mass spectrometry (LC–MS) was used to detect the isolated proteins.

### RNA immunoprecipitation

RNA immunoprecipitation was performed as previously described (Zhang et al. 2017) Briefly, UV-cross-linking of living cells (1 × 10^6^) was performed at 254 nm (2000 J/m^2^). Cells were washed with cold phosphate-buffered saline (PBS) and then lysed in 300 µL of lysis buffer (200 U/mL RNase inhibitor; Thermo Fisher, MA, USA), 0.5% C24H39O4Na, 0.5 % NP40, and protein inhibitor (Thermo Fisher) for 1 h. After treatment with DNase I (NEB, MA, USA) for 20 min, the cell lysate was incubated with anti-P14 antibody or IgG (Sigma) overnight at 4 °C. Then, 50 µL of protein A/G agarose beads (Santa Cruz, CA, USA) were added to the cell lysate. RNA binding with SF3B6 was recovered using Trizol-chloroform and detected using qPCR.

### Construction of cell lines with knockdown or overexpressed DKFZp434J0226

To knock down *DKFZp434J0226*, 50 nM *DKFZp434J0226*siRNA (SI05138511, si 1# and SI05138518, si 2#, Qiagen, Hilden, Germany) was transfected into MIAPaCa-2 cells and PANC-1 cells using Lipofectamine 2000 reagent, according to the manufacturer’s instructions (Wei et al. 2008). Control groups were transfected with the transfection agent, but not siRNA (mock) or scrambled control siRNA (negative control). To clone the full-length*DKFZp434J0226*, PCR was performed with primers 5′-CGGAATTCGCCTTGGTGTTCAAGAAGATTCCAG-3′ and 5′-CGGAATTCTTTATTCTTACTACATAAGATCCAC-3′. Full-length *DKFZp434J0226* was subcloned into the pLVX-IRES-Puro lentiviral expression vector (Clontech, CA, USA). pLVX-IRES-Puro-*DKFZp434J0226* or pLVX-IRES-Puro Packaging psPAX2 and envelope pMD2.G vectors were co-transfected into HEK 293T cells. Lentivirus-containing medium was collected from HEK 293T cells and filtered. In the presence of 8 µg/mL polybrene, AsPC-1 cells and CFPAC-1 cells were infected with the enveloped lentivirus. Stable cell lines were selected using puromycin (10 µg/mL). For the knockdown of SF3B6, 50 nM SF3B6 siRNA (AM16708, Thermo Fisher) was transfected into AsPC-1 cells.

### Cell proliferation assay

Cell proliferation was measured using cell counting kit-8 (Dojindo Co., Kumamoto, Japan) according to the manufacturer’s protocol. Cells were incubated with CCK-8 for 1 h in triplicate. We assessed the cell proliferation rate by measuring the absorbance at 450 nm using a universal microplate reader. Four biological replicates were analyzed.

### Colony formation assay

Soft agar colony formation assay was performed to measure the anchorage-independent growth ability as described previously (Liu et al. 2013). A total of 1 × 10^3^ cells per well were suspended in DMEM containing 0.3% noble agarose (Takara) in six-well plates. The suspension was laid over DMEM containing 0.6% noble agarose and further overlaid with DMEM. After replenishing the medium every other day, the plates were incubated for 14 days in a 5% CO_2_ incubator at 37 °C. A Nikon ECLIPSE TE300 microscope was used to image the colonies. Four biological replicates were analyzed.

### Cell migration and invasion assays

Cell migration capacity was measured by a cell migration (wound-healing) assay, as described previously (Bao et al. 2012). Cells were seeded in a six-well plate and incubated at 37 °C until they reached 90% confluence. The confluent cells were then scratched with a 200 mL pipette tip and washed with PBS, followed by incubation with Mag in complete medium. After 24 h of incubation, the cells were fixed and stained with 2% ethanol containing 0.2% crystal violet powder (15 min), and randomly chosen fields were photographed under a light microscope. The number of cells that migrated into the scratched area was calculated. Cell invasive potential was studied by calculating the number of cells that invaded through Matrigel-coated transwell polycarbonate membrane inserts as described previously (Verma et al. 2006). In brief, transwell inserts with a pore size of 12 Am were coated with 0.78 mg/mL Matrigel in serum-free medium. Cells were recovered by trypsinization, washed, and resuspended in a serum-free medium. Then, 0.5 mL of the cell suspension (0.5 × 10^6^ cells) was added to duplicate wells. After incubation for 48 h, the cells that passed through the filter were stained using the Hema-3 stain kit (Fisher Scientific, Houston, TX, USA). The cells in 10 random fields were counted under a microscope. Both assays were performed in four biological replicates.

### Subcellular fractionation

Cells were resuspended in harvest buffer (10 mM HEPES [pH 7.9], 50 mM NaCl, 0.5 M sucrose, 0.1 mM EDTA, 0.5% Triton-100, phosphatase inhibitors, and PMSF) and incubated on ice for 10 min. Nuclei were obtained by centrifugation at 12,000 rpm for 10 min at 4 °C. Nuclei were washed three times with harvest buffer and dissolved in 1× loading buffer. The supernatant containing the cytosolic extract was also collected and dissolved in 5× loading buffer. The cytoplasmic and nuclear fractions were analyzed by sodium dodecyl sulphate-polyacrylamide gel electrophoresis (SDS-PAGE). The validity of fractionation was detected by western blotting using LaminB as the nuclear protein control and β-tubulin as the cytosolic protein control.

### Co-immunoprecipitation and western blotting

Cells were lysed with lysis buffer (50 mM Tris-HCl pH 7.4, 150 mM NaCl, 1 mM NaF, 1% NP-40, 1 mM EDTA, and 1× protease and phosphatase inhibitor solution) on ice for 30 min. Protein A/G agarose beads were used to pre-clear the supernatants. Anti-SF3B6 antibody (dilution: 1:500; Catalog number PA5-57077, Thermo Scientific, USA) was used for immunoprecipitation. The mixture was incubated with protein A/G-agarose beads (Santa Cruz Biotechnology) overnight on a mechanical shaker at 4 °C. The beads were harvested and rinsed three times with a lysis buffer. Immunoblotting was used to detect bead-captured SF3B6. Proteins were separated by 10% SDS-PAGE and electrophoretically transferred to a polyvinylidene difluoride membrane (Millipore, MA, USA). The membrane was incubated with the indicated antibody at 4 °C overnight after blocking with 5% non-fat dry milk. An enhanced chemiluminescence kit (Tiangen, Beijing, China) was used to develop the blots. Primary antibodies against P-tyrosine (dilution: 1:500; Catalog number sc-207232, Santa Cruz), SF3B6 (dilution: 1:500; Catalog number ARG40015, Arigo Biolaboratories, Taiwan, China), proliferating cell nuclear antigen PCNA, (dilution: 1:2000; Catalog number sc-56, Santa Cruz), GAPDH (dilution: 1:1000; Catalog number sc-47724, Santa Cruz), LaminB (dilution: 1:500; Catalog number ab32535, Abcam, Cambridge, MA), and β-tubulin (dilution: 1:500; Catalog number ab6046, Abcam, Cambridge, MA) were used as indicated. Each western blotting assay was replicated three times.

### Immunofluorescence

Cells were incubated on glass coverslips pre-coated with poly-l-lysine, fixed with 4% paraformaldehyde, permeabilized with 0.2% Triton X-100, and blocked in 10% bovine serum albumin in PBST for 30 min at 25 ℃. After staining with secondary antibodies, the primary antibodies were incubated at 4 °C overnight. Primary antibodies against SF3B6 (dilution: 1:500; Catalog number PA5-57077, Thermo Scientific, USA) and DAPI (Beyotime, Shanghai, China) were used to identify SF3B6 and cell nuclei. A Leica confocal microscope (Leica TCS SP8, Germany) was used to capture the images.

### Animal studies

Animal studies were approved by the Animal Use Committee at Fudan University. Four-week-old female BALB/c nude mice were obtained from the Experimental Animal Center of Fudan University. Animals were housed with a 12-h light/dark cycle, and MiaPaCa-2 cells infected with control lentivirus and *DKFZp434J0226* lentivirus (2 × 10^6^ viable cells/mouse) were resuspended in PBS (0.1 mL) and injected subcutaneously into the right dorsal flank of BALB/c-nu/nu mice. After the tumor volume reached 200 mm^3^, control group (n = 6) and *DKFZp434J0226* overexpressed group (n = 7) were analyzed. The tumor volume was calculated as

$$\text{V} (\text{mm}^3) = (\text{ab}^2)/2,$$

where “a” indicates the tumor length and “b” indicates the tumor width. The mice were anesthetized by intraperitoneal administration of pentobarbital (75 mg/kg) and tumors were harvested by surgery once tumor size reached 1200 mm^3^. Tumors were measured every 3–4 days. Mice bearing large tumors were carefully monitored for any signs of discomfort.

### Statistical analysis

Statistical analyses were performed using GraphPad Prism 5.0 software (GraphPad Prism Software Inc., San Diego, CA). Unpaired Student’s t-test or the Mann–Whitney U test was used for comparing two groups. Paired data were compared using a paired t-test. Analysis of variance (ANOVA) was used to compare three or more groups. Ordinary one-way ANOVA with the Sidak test was used for multiple comparisons between different groups. Categorical variables were compared using the χ^2^ test. Independent two-sample t-tests were used to compare continuous variables. Univariate and multivariate analyses were performed using the Cox proportional hazards model. Survival curves were obtained using the Kaplan–Meier method with the log-rank test. Data are presented as mean ± standard deviation (SD) of three independent experiments. All tests were two tailed, and a *P*-value of < 0.05 was considered statistically significant.

## Results

### LncRNA expression profile in PDAC

**Fig. 1:**
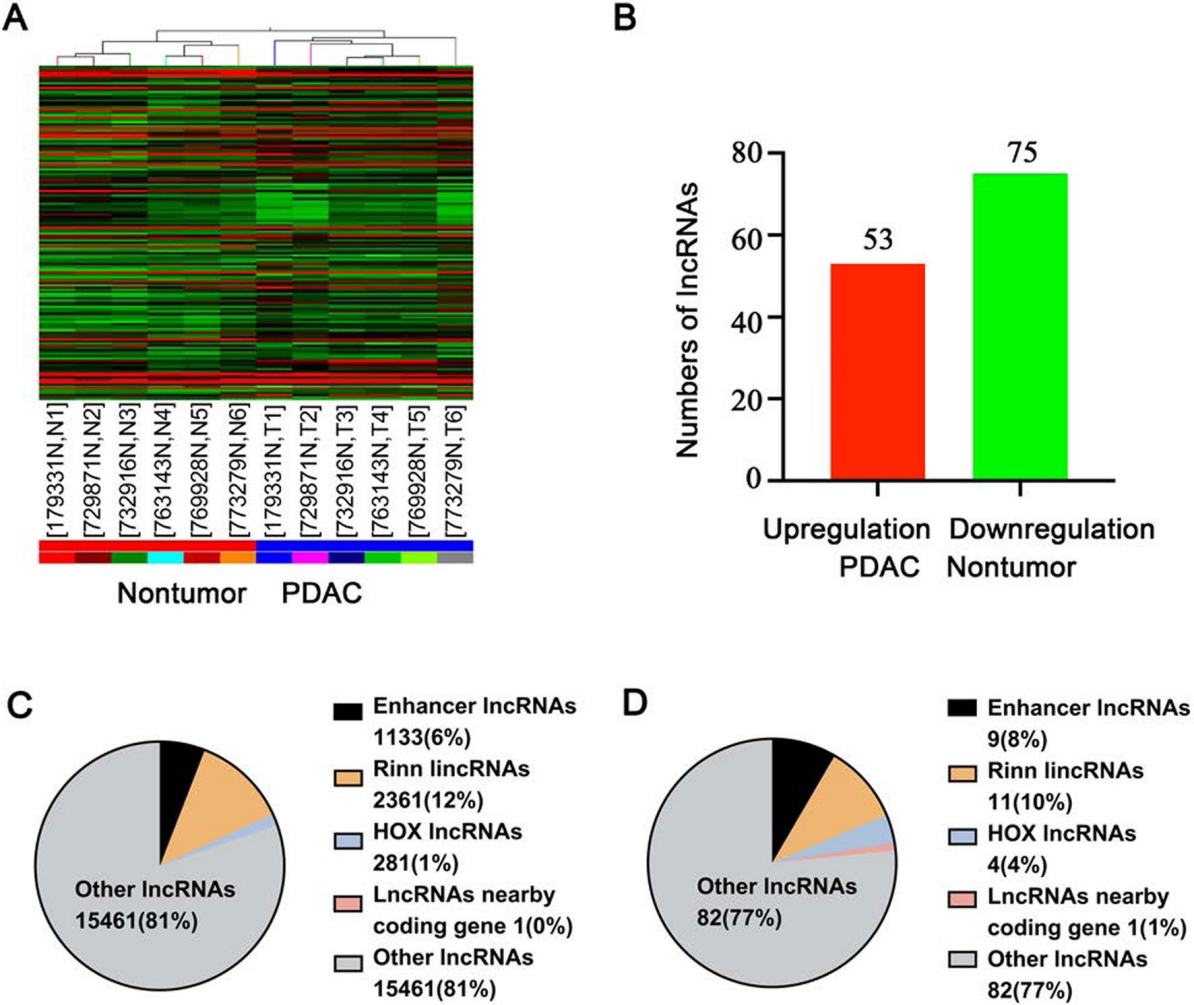
Microarray analysis of differentially expressed lncRNAs between six pancreatic ductal adenocarcinoma (T) samples and paired non-tumor (N) samples. **A** Hierarchical cluster analysis of differentially expressed (≥ twofold change) lncRNAs between six pancreatic ductal adenocarcinoma (T) samples and paired nontumor (N) samples. The red color indicates upregulation, while the green color indicates downregulation. **B** A total of 128 lncRNAs, with the upregulation of 53 lncRNAs and downregulation of 75 lncRNAs, were selected for clustering. **C** Classifications of detected lncRNAs in pancreatic ductal adenocarcinoma samples. **D** Classifications of significantly differentially expressed lncRNAs between pancreatic ductal adenocarcinoma samples and paired nontumor samples

Systematic variations in the expression of lncRNAs between six PDAC samples and paired non-tumor samples were demonstrated by hierarchical clustering (Fig. 1A). Additional file 1: Table S2 shows the clinical characteristics of the patients. The expression levels of lncRNAs in paired samples were clearly shown by calculating the log fold change Tumor/Normal (T/N). We identified 128 aberrantly expressed lncRNAs (≥ twofold, *P* < 0.05) in RefSeq_NR, Fantom, lncRNA, UCR Ensembl, misc_lncRNA, Fantom_stringent, UCSC_knowngene, H-invDB, NRED, and RNAdb, with 53 upregulated lncRNAs and 75 downregulated lncRNAs (Fig. 1B and Additional file 1: Table S3).

LncRNAs were classified as Enhancer lncRNAs, Rinn lincRNAs, HOX lncRNAs, and LincRNAs nearby coding genes (Ørom et al. 2010; Rinn et al. 2007). Human Homeobox transcription factor (HOX) clusters have been found to contribute to the formation of numerous lncRNAs (Rinn et al. 2007). The lncRNAs expressed in site-specific fashion may have a general regulating ability as the HOX and used similar enhancers as HOX genes. In our study, approximately 281 HOX lncRNAs were detected, four of which were aberrantly expressed in human HOX loci in PDAC (Additional file 1: Table S4). Eleven of the 2341 lncRNAs were found to be aberrantly expressed, and the profiling data of Rinn lncRNAs are provided in Additional file 1: Table S5. Nine of the 1m133 enhancer lncRNAs were detected with different expressions, and the profiling data are shown in Additional file 1: Table S6. However, we did not find any enhancer lncRNAs nearby coding genes (distance, 300 kb), except for one lncRNA (distance, 300 kb; lncRNA-AK000839 and coding gene-NM_001075099). The classification of the detected lncRNAs and differentially expressed lncRNAs in paired samples is summarized in Fig. 1C, D, respectively.

### Overview of mRNA profiles

**Fig. 2:**
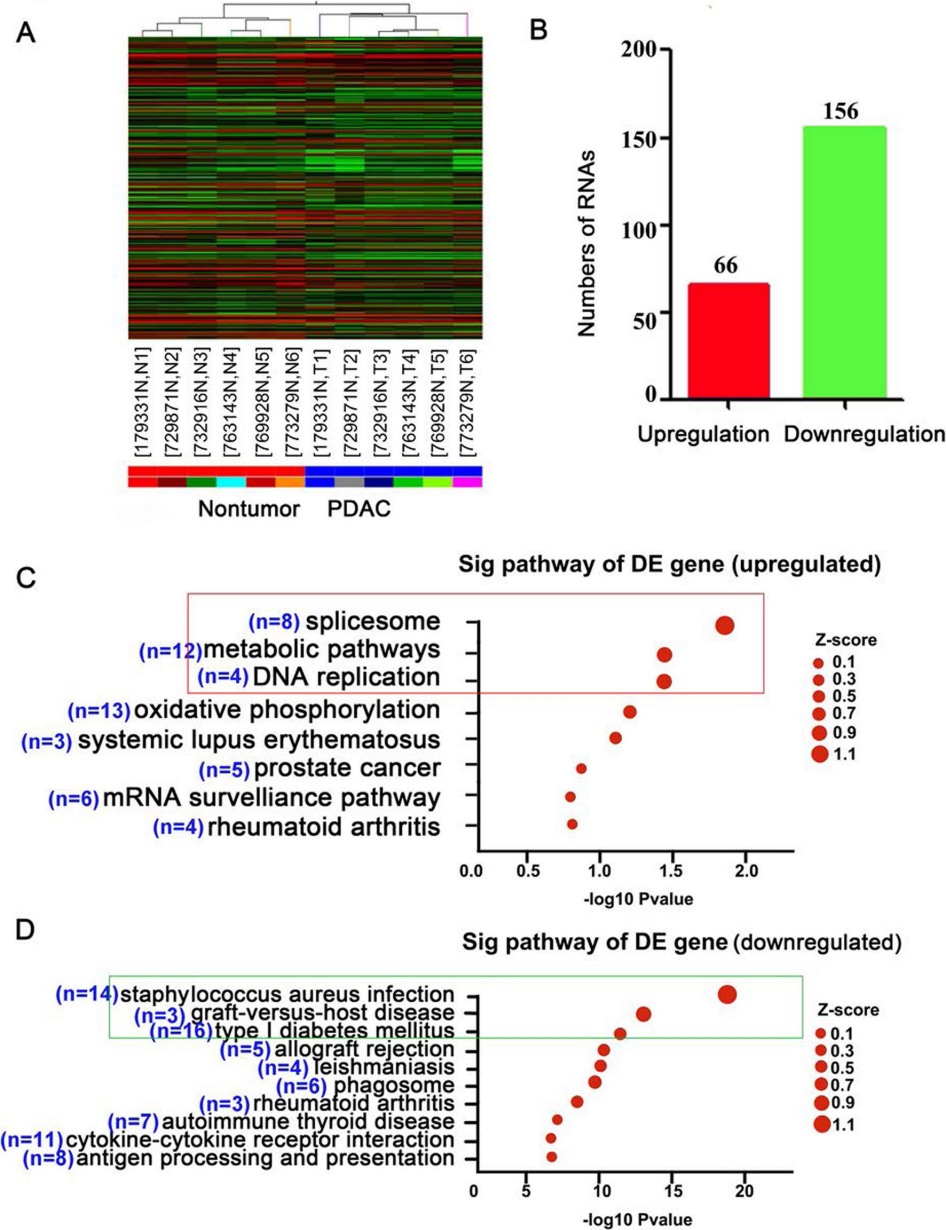
Microarray analysis of differentially expressed mRNAs between six pancreatic ductal adenocarcinoma (T) samples and paired non-tumor (N) samples. **A** Hierarchical cluster analysis of differentially expressed (≥ twofold change) mRNAs between six pancreatic ductal adenocarcinoma (T) samples and paired non-tumor (N) samples. The red color indicates upregulation, while the green color indicates downregulation. **B** A total of 222 mRNAs, with the upregulation of 66 mRNAs and downregulation of 156 mRNAs, were selected for clustering. **C** Gene ontology analysis of the differentially expressed genes (DE) between six PDAC tumors and paired non-tumor samples. The upregulated mRNAs are mainly aggregated in (a) the “spliceosome,” (b) “metabolic pathway,” and (c) “glycosaminoglycan and keratan sulfate” (framed in red). The downregulated mRNAs mainly associated with (d) “*Staphylococcus aureus* infection,” (e) “graft-versus-host disease,” and (f) “type I diabetes mellitus” (framed in green)

Systematic variations in the expression of protein-coding mRNAs in the six PDAC samples and paired non-tumor tissues are shown by hierarchical clustering (Fig. 2A). A total of 222 mRNAs were differentially expressed among the six pairs of samples, with the upregulation of 66 mRNAs and downregulation of 156 mRNAs in PDAC compared to that in the corresponding normal samples (Fig. 2B and Additional file 1: Table S7). GO analysis indicated the upregulated mRNAs were mostly aggregated in the “spliceosome,” the “metabolic pathway,” and “glycosaminoglycan and keratan sulfate” (Fig. 2C), and the downregulated mRNAs were mainly associated with “*Staphylococcus aureus* infection,” “graft-versus-host disease,” and “type I diabetes mellitus” (Fig. 2D).

### *DKFZp434J0226* can be an independent prognostic factor in patients with PDAC

**Fig. 3:**
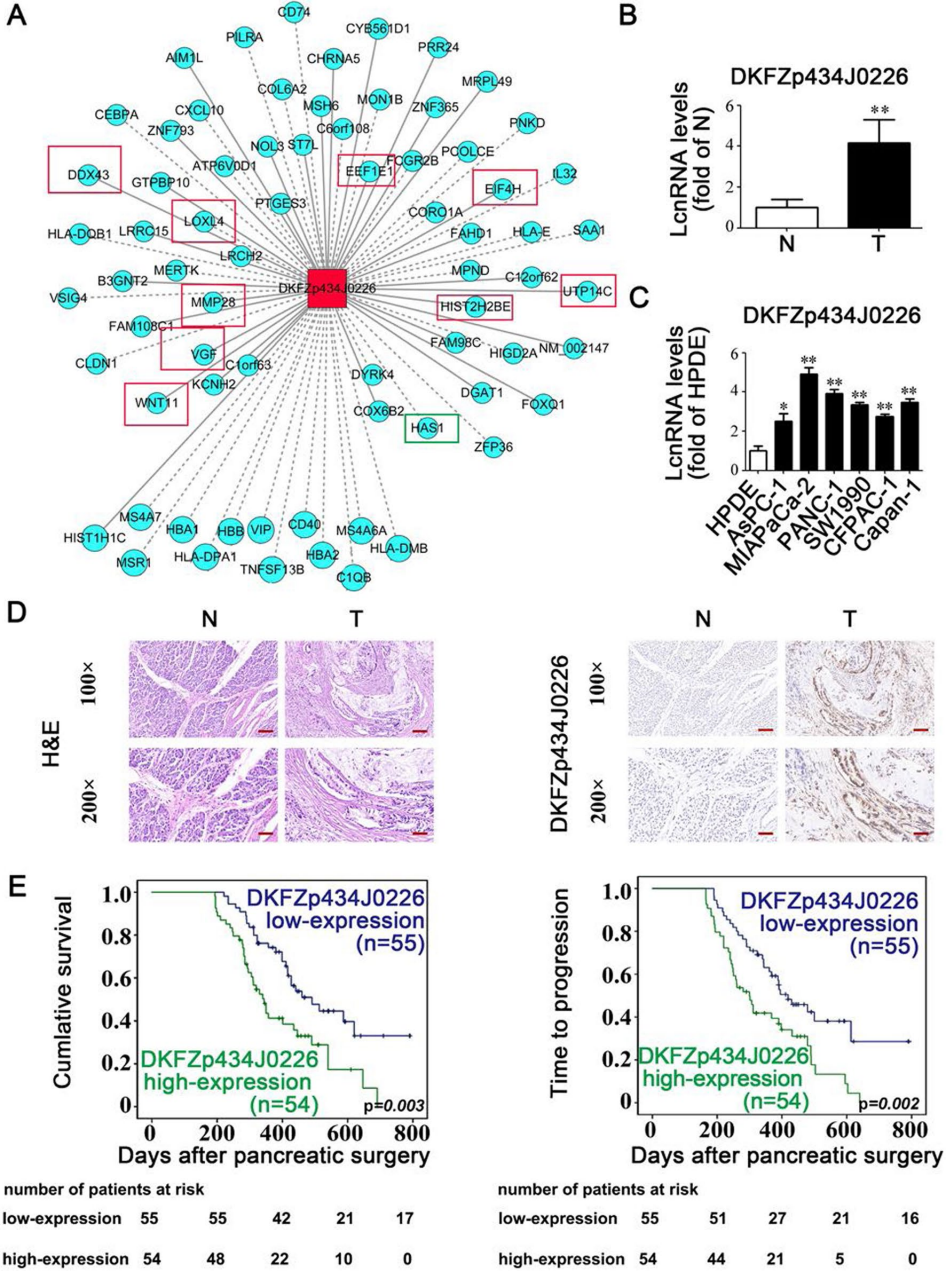
Increased *DKFZp434J0226* (DKFZ) expression is positively associated with poor prognosis of pancreatic ductal adenocarcinoma. **A** Coding-non-coding gene co-expression network analysis showing the association between DKFZ and genes involved in cancer growth (framed in red) and metastasis (framed in green). **B** RT-qPCR analysis showing the increased expression of DKFZ in PDAC compared to that in the corresponding non-tumor tissues (n = 109). **C** RT-qPCR analysis demonstrating the increased expression of DKFZ in six PDAC cell lines (AsPC-1, MIAPaCa-2, PANC-1, SW1990, CFPAC-1, and Capan-1) compared to that in immortal, transformed, human pancreatic ductal epithelial cells. **D** Typical patterns of DKFZ expression in paired PDAC tissues. Left panel: HE staining of paired PDAC tissue. Right panel: in situ hybridization of DKFZ in paired PDAC tissues. N, adjacent nontumorous tissues; T, tumor tissues. **E** Kaplan–Meier analysis for OS and TTR according to DKFZ expression (cutoff value: median value) in 109 patients with PDAC. Data are shown as mean ± SD; *P < 0.05, **P < 0.01

We investigated the relationship between *DKFZp434J0226* (one with the greatest changes in expression) and the protein-coding genes using an absolute correlation coefficient cutoff of > 0.85 and an FRD of < 0.1. We found that hundreds of coding genes involved in cancer growth and metastasis were significantly associated with the lncRNA *DKFZp434J0226* (Fig. 3A). We further examined the expression of *DKFZp434J0226* in 109 pairs of PDAC and corresponding non-tumor tissue samples using RT-qPCR. Our data confirmed that *DKFZp434J0226* was overexpressed in PDAC tissues compared to non-tumor tissues (Fig. 3B). Additionally, similar expression patterns were observed in the six PDAC cell lines compared to the immortal human pancreatic ductal epithelial (HPDE) cell line (Fig. 3C). Thus, our data demonstrated a strong consistency between the microarray data and qPCR results.

In the CNC network, *DKFZp434J0226* was connected to a variety of protein-coding genes involved in cancer metastasis and growth (Fig. 3A). Therefore, we studied the role of the lncRNA *DKFZp434J0226* in PDAC. In situ hybridization confirmed that the expression of *DKFZp434J0226* was increased in PDAC tissues and was localized in both the nucleus and cytoplasm (Fig. 3D). We further examined the association between *DKFZp434J0226* expression and clinical characteristics of 109 PDAC tissue samples from patients. Additional file 1: Table S8 presents the clinical characteristics of the patients. Clinicopathological analysis showed that high *DKFZp434J0226* levels in PDAC tissues were significantly correlated with tumor grade, perineural invasion, and tumor-node-metastasis (TNM) stage. However, *DKFZp434J0226* levels failed to match other clinicopathological data (Additional file 1: Table S9). Kaplan–Meier curves showed that high *DKFZp434J0226* expression in tumor samples was positively correlated with shorter overall survival (OS) and time to progression (TTP) (*P* = 0.003 and *P* = 0.002, respectively; Fig. 3E). Univariate analysis indicated that high *DKFZp434J0226* expression was correlated with both OS (*P* = 0.002) and TTP (*P* = 0.003). In addition, other factors indicative of shorter OS was tumor grading, tumor size, perineural invasion, vascular invasion, lymph node metastasis, and TNM stage. The factors associated with lymph node metastasis, tumor grading, vascular invasion, and TTP included age, tumor size, perineural invasion, and TNM stage, as shown in Additional file 1: Table S10. Multivariate analyses revealed that high *DKFZp434J0226* levels were independently associated with worse OS (*P* = 0.014) and higher PDAC recurrence (TTP, *P* = 0.024; Table 1).

**Table 1:** Multivariate analyses of factors associated with OS and TTP

	Hazard ratio (95% CI)	*P*
OS		
Tumor differentiation (I vs. II vs. III)	0.940 (0.488–1.811)	0.853
Tumor size (T1 vs. T2 vs. T3)	1.817 (0.668–4.944)	0.243
Lymph node metastasis (negative vs. positive)	1.263 (0.722–2.209)	0.414
Perineural invasion (negative vs. positive)	0.684 (0.336–1.392)	0.295
Vascular invasion (negative vs. positive)	1.336 (0.770–2.318)	**0.002**
TNM stage (I vs. II)	1.913 (0.470–7.795)	0.365
DKFZp434J0226 expression (low vs. high)	2.017 (1.154–3.527)	**0.014**
TTP		
Age, years (≤ 60 vs. >60)	1.523 (0.877–2.647)	0.135
Tumor grade (I vs. II vs. III)	1.040 (0.566–1.912)	0.899
Tumor size (T1 vs. T2 vs. T3)	1.966 (0.812–4.764)	0.134
Lymph node metastasis (negative vs. positive)	1.481 (0.866–2.534)	0.151
Perineural invasion (negative vs. positive)	0.567 (0.289–1.111)	0.098
Vascular invasion (negative vs. positive)	1.385 (0.790–2.428)	**0.006**
TNM stage (I vs. II)	1.683 (0.474–5.970)	0.421
DKFZp434J0226 expression (low vs. high)	1.875 (1.085–3.237)	**0.024**

*OS* overall survival, *TTP* time to progression

Variables were adopted for prognostic significance using univariate analysis (*P* < 0.05). Bold *P* values less than 0.05, indicating statistical significance

### Effect of *DKFZp434J0226* on PDAC cell proliferation, invasion, and migration

**Fig. 4:**
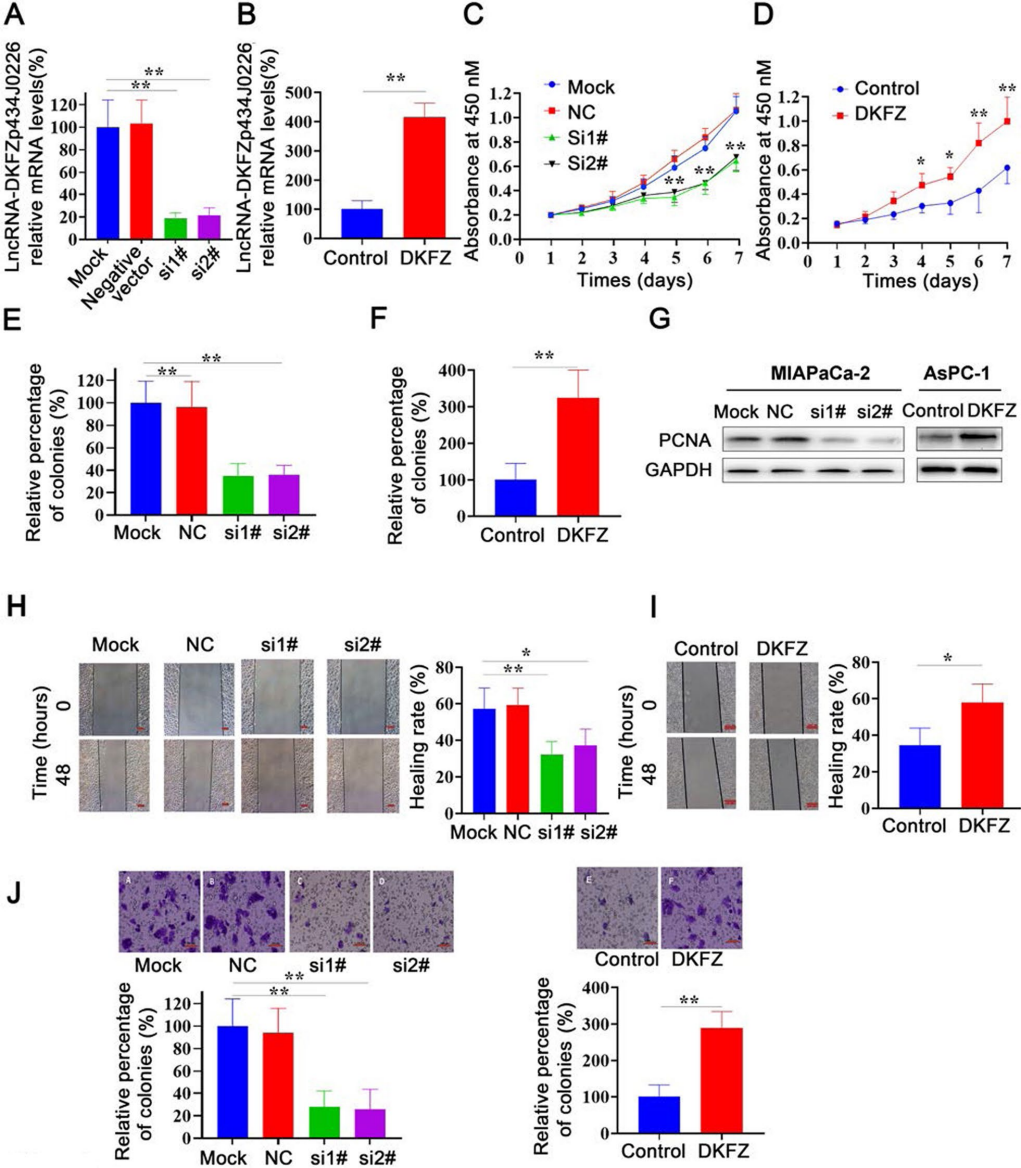
*DKFZp434J0226* (DKFZ) promotes PDAC cell proliferation and migration. **A, B** Verification of DKFZ knockdown (**A**) and DKFZ overexpression (**B**) efficiency in MIAPaCa-2 and AsPC-1 cells (n = 3). Data are shown as mean ± SD; *P < 0.05, **P < 0.01. **C** Cell growth curves of lipofectamine-treated MIAPaCa-2 (Mock) cells, negative siRNA-transfected MIAPaCa-2 (NC) cells, and two DKFZ siRNA-transfected MIAPaCa-2 (si1 and si2; n = 4). Data are shown as mean ± SD; *P < 0.05 vs. Mock and NC; **P < 0.01 vs. Mock and NC. **D** Cell growth curves of control lentivirus-infected AsPC-1 cells (control) and DKFZ lentivirus-infected AsPC-1 cells (DKFZ) (n = 4). Data are shown as mean ± SD; *P < 0.05 vs. Mock and NC; **P < 0.01 vs. Mock and NC. **E** Soft agar colony formation assay and relative levels of colonies in Mock, NC, si1, and si2 cells (n = 4). **F** Soft agar colony formation assay and relative levels of colonies in control and DKFZ cell (n = 4). **G** Western blotting analysis of PCNA expression in Mock, NC, si1 and si2 MIAPaCa-2 cells. Western blotting analysis of PCNA expression in control and overexpression AsPc-1 cells. **H**–**J** Wound healing (**H, I**) and migration (**J**) assays using the referred PDAC cells. Quantification using ImageJ (n = 4). **E–J** Data are shown as mean ± SD; *P < 0.05, **P < 0.01. Scale bars refer to 100 μm

To estimate the effect of *DKFZp434J0226* on cell biological behaviors, we established MIAPaCa-2 cell line with *DKFZp434J0226* knockdown (Fig. 4A) and AsPC-1 cell line with *DKFZp434J0226* overexpression (Fig. 4B). Cell growth was inhibited by *DKFZp434J0226* knockdown using cell counting kit-8 assays (Fig. 4C) and elevated by *DKFZp434J0226* overexpression (Fig. 4D). Soft agar colony formation assay indicated that the colony formation ability was decreased by *DKFZp434J0226* knockdown (Fig. 4E), and increased by DKFZp434J0226 overexpression (Fig. 4F). Consistent with the results of cell proliferation, DKFZp434J0226-knockdown MIAPaCa-2 and PANC-1 cells and *DKFZp434J0226-*overexpressing AsPC-1 and CFPAC-1 cells showed lower and higher expression of PCNA, respectively, compared to control cells (Fig. 4G). Cell scratch assay demonstrated that the capacity of cells to migrate was attenuated by *DKFZp434J0226* knockdown and enhanced by *DKFZp434J0226* overexpression (Fig. 4H, I). Transwell invasion assay indicated that the invasiveness of cells was inhibited by DKFZp434J0226 knockdown and promoted by *DKFZp434J0226* overexpression (Fig. 4J). The similar effects of *DKFZp434J0226* on cell biological behaviors were also observed in PANC-1 cell line with *DKFZp434J0226* knockdown and CFPAC-1 cell line with *DKFZp434J0226* overexpression (Additional file 2: Fig. S1A–I).

### *DKFZp434J0226* interacts with the splicing factor SF3B6 and promotes its phosphorylation

**Fig. 5:**
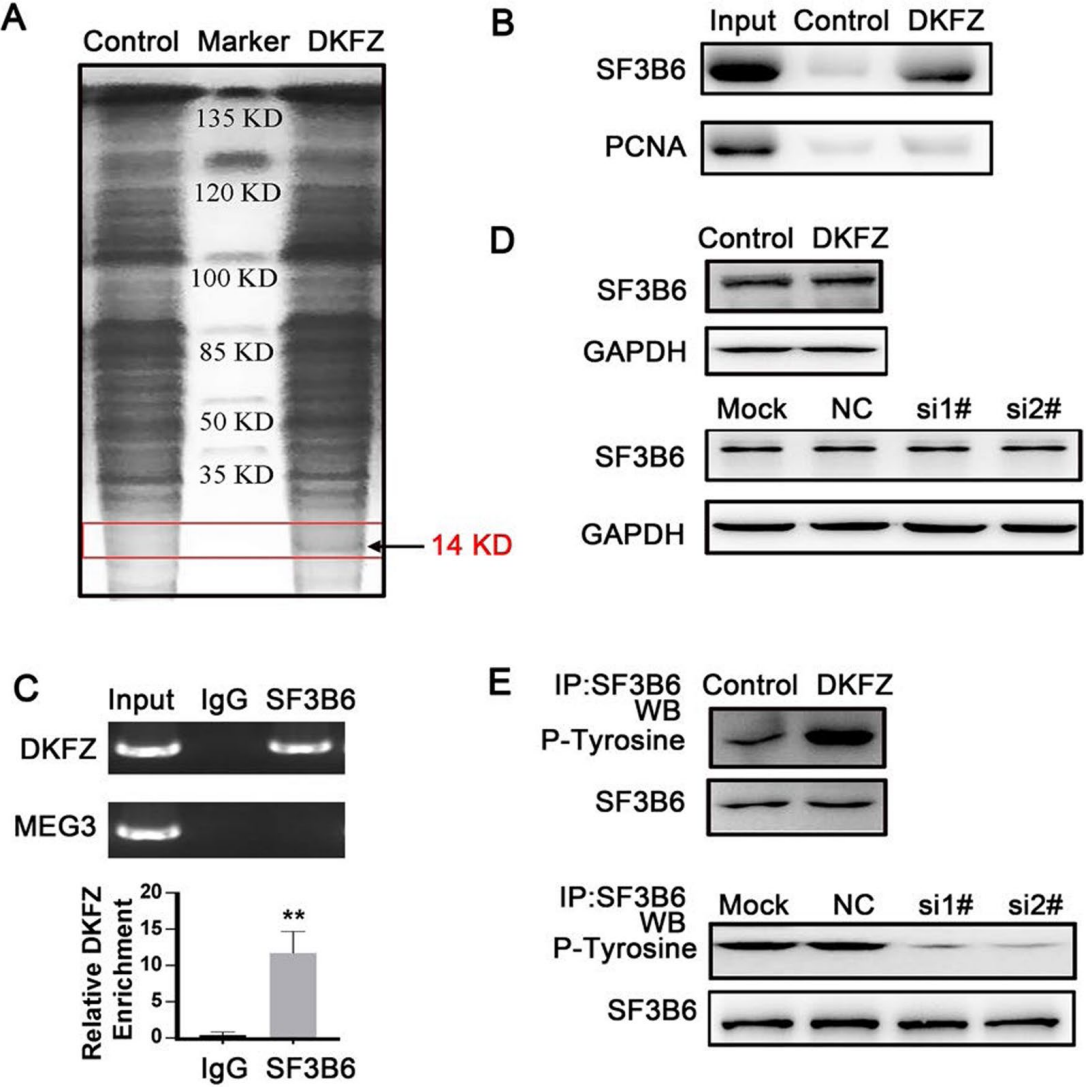
SF3B6 serves as a *DKFZp434J0226* (DKFZ)-binding protein. **A** RNA pull-down assay performed in the HEK293T cell lysates with biotin-labeled oligos. After pull-down, proteins were subjected to SDS-PAGE and stained by Coomassie brilliant blue. The band indicated by the arrow was subjected to mass spectrometry. **B** Western blotting analysis determined the specific interaction of sense DKFZ with SF3B6 protein, but not with PCNA protein (negative control). **C** RNA immunoprecipitation (RIP) of SF3B6 interaction with DKFZ in the HEK293T cells. RNA-protein complexes immunoprecipitated by anti-SF3B6 or control IgG were determined by RT-qPCR using specific primers for DKFZ or MEG3 (negative control). Data are shown as mean ± SD (n = 4); **P < 0.01. **D** Western blotting analysis of SF3B6 levels in the indicated DKFZ overexpression AsPC-1 cells or DKFZ knockdown MIAPaCa-2 cells. **E** Western blotting analysis of SF3B6 phosphorylation levels in the indicated DKFZ overexpression AsPC-1 cells or DKFZ knockdown MIAPaCa-2 cells. For SF3B6 phosphorylation detection, cell lysates were prepared and subjected to immunoprecipitation (IP) with anti-SF3B6 antibody, followed by immunoblotting analysis with the anti-phospho-tyrosine antibody

To identify *DKFZp434J0226*-interacting proteins, we performed native RNA pull-down assay combined with mass spectrometry (RAP-MS) using MIAPaCa-2 cells (Fig. 5A). Mass spectrometry analysis revealed that a 14-KDa protein, SF3B6, interacted with *DKFZp434J0226*. Western blotting with anti-SF3B6 antibody confirmed that SF3B6 interacted with *DKFZp434J0226* (Fig. 5B). In contrast, *DKFZp434J0226* was not associated with PCNA protein (a negative control). Cross-linked RIP assays demonstrated an endogenous association between SF3B6 and *DKFZp434J0226* (Fig. 5C). The expression of *DKFZp434J0226*, but not *MEG3* (a negative control), was upregulated in samples immunoprecipitated with anti-SF3B6 antibodies compared to that in samples immunoprecipitated with lgG. These results demonstrated that the SF3B6 protein can bind to *DKFZp434J0226*RNA. The splicing pathway in eukaryotes contains the U2- and U12-dependent pathways, splicing-specific pre-mRNA introns differing in the branch point and splice site consensus sequences. SF3B6 is a member of the SF3B spliceosome and is present in the U2- and U12-dependent pathways, responsible for the first catalytic step of the splicing reaction (Yokoi et al. 2011; Will et al. 2001). It has a close contact with branch site A in the assembled spliceosome, indicating that SF3B6 plays a crucial role in the splicing process (Perea et al. 2016). Next, we examined the protein levels of SF3B6 in*DKFZp434J0226* overexpressed and knockdown cells (Fig. 5C). No significant difference in SF3B6 protein levels was observed in overexpressed or knockdown cells (Fig. 5D). As previous studies have reported the importance of phosphorylation in alternative splicing, we examined the level of SF3B6 phosphorylation in both overexpressed and knockdown cells and found that SF3B6 phosphorylation levels were significantly increased in *DKFZp434J0226-*overexpressed cells and decreased in *DKFZp434J0226*-knockdown cells (Fig. 5E).

### *DKFZp434J0226* promotes translocation of SF3B6 from the cytoplasm to the nucleus and modulates alternative splicing

**Fig. 6:**
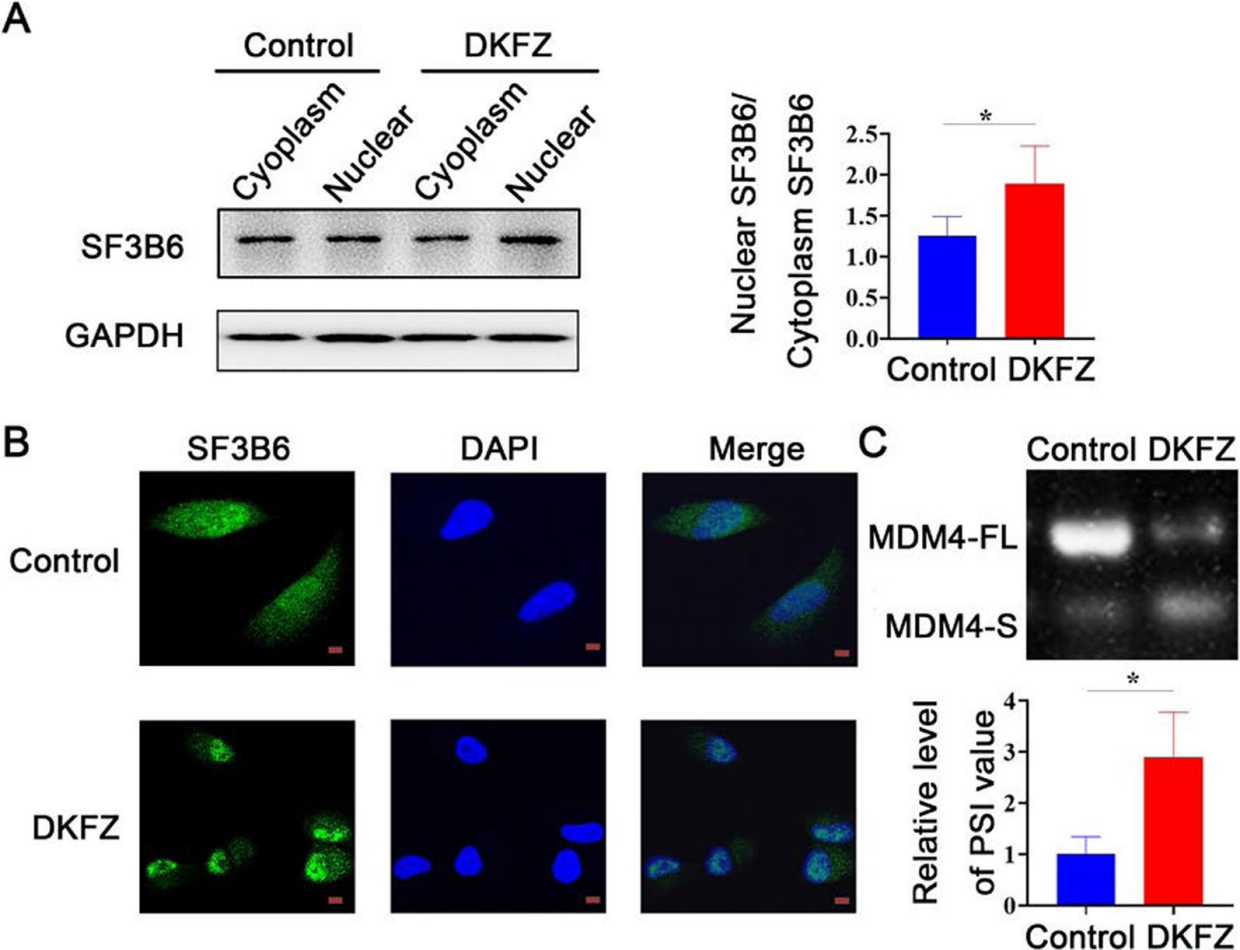
*DKFZp434J0226* (DKFZ) increases the nuclear localization of SF3B6. **A** Western analysis and quantification of nuclear and cytoplasmic SF3B6 in control and DKFZ-overexpressed AsPC-1 cells. Nuclear and cytoplasmic subcellular fractions were extracted as described in “Materials and methods” section. **B** Protein levels of SF3B6 in the nucleus and cytoplasm observed using confocal microscope. **C** DKFZ regulates alternative splicing of SF3B6 downstream MDM4. The alternative spliced transcripts (MDM4-FL, MDM4 full-length transcript; MDM4-S, MDM4 S transcript) in control and DKFZ-overexpressed AsPC-1 cells were verified by RT-qPCR. The percent spliced in index (PSI) was quantified for the alternative splicing events (n = 5). **A**, **C** Data are shown as mean ± SD; *P < 0.05, **P < 0.01. Scale bars refer to 20 μm

To identify the effect of *DKFZp434J0226* on splicing factor SF3B6, we examined the expression of SF3B6 in the nucleus and cytoplasm of *DKFZp434J0226*-overexpressed AsPC-1 cells using western blotting. *DKFZp434J0226* overexpression promoted the translocation of SF3B6 from the cytoplasm to the nucleus (Fig. 6A). Furthermore, we performed immunofluorescence analysis using *DKFZp434J0226*-overexpressed and control cells. In *DKFZp434J0226-*overexpression AsPC-1 cells, SF3B6 signals aggregated in the nucleus (Fig. 6B). Together, these results indicated that the lncRNA *DKFZp434J0226* may contribute to the translocation of SF3B6 from the cytoplasm to the nucleus in PDAC cells.

To investigate the effect of *DKFZp434J0226*on alternative splicing, we analyzed the alternative splicing of the MDM4 transcript, which has been recently reported as an SF3B6 target mRNA (Siebring-van Olst et al. 2017). RT-qPCR indicated that *DKFZp434J0226* overexpression promoted the expression of the MDM4-S transcript and decreased the expression of the MDM4-FL transcript in AsPC-1 cells (Fig. 6C). These results indicated a potential role of *DKFZp434J0226* in modulating alternative splicing events.

### SF3B6 is required in *DKFZp434J0226*-induced cell proliferation and migration

**Fig. 7:**
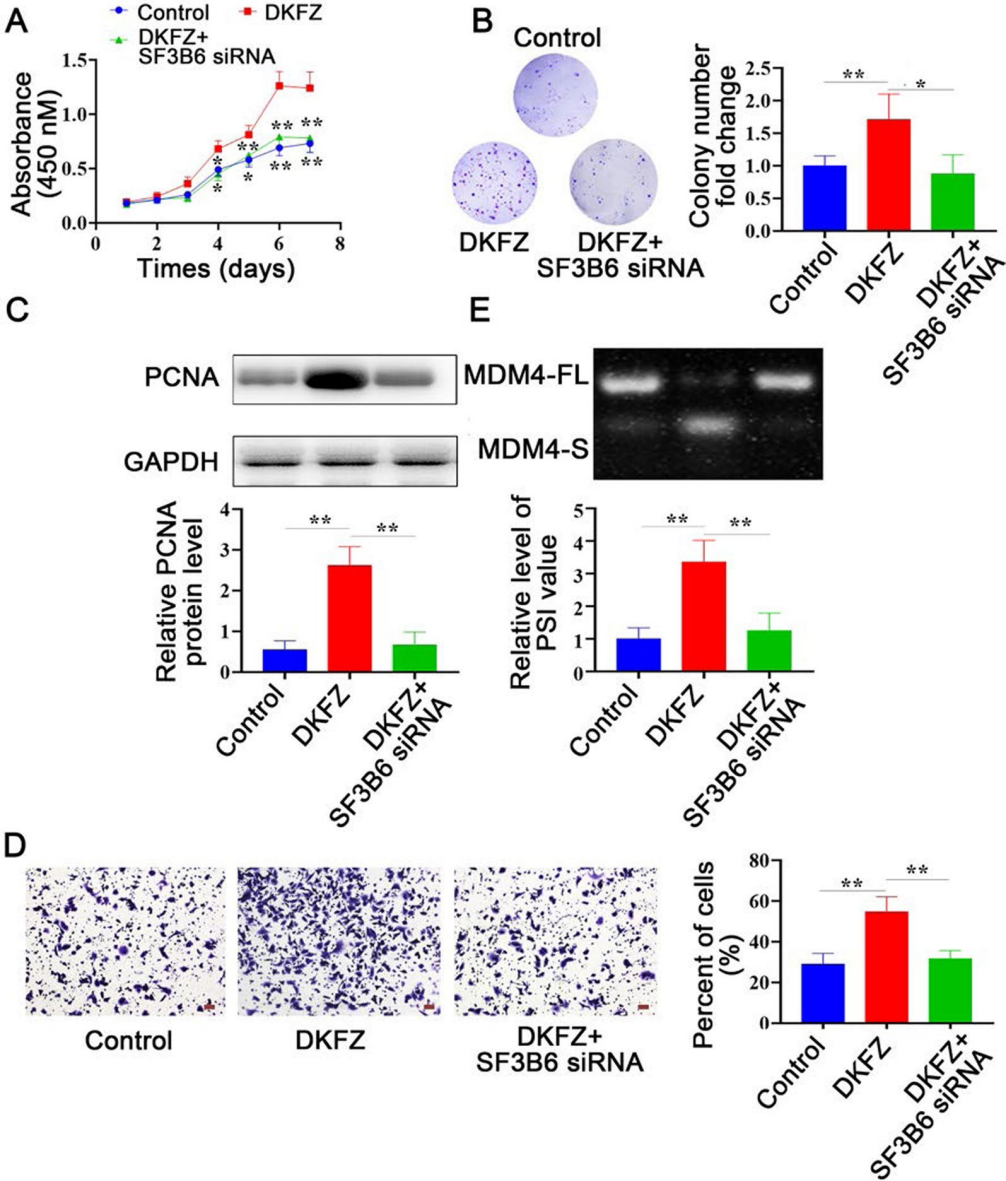
SF3B6 is required in *DKFZp434J0226* (DKFZ)-induced proliferation and migration. **A–C** Cell growth curves assay (**A**), soft agar colony formation assay (**B**), and western blotting of PCNA (**C**) demonstrating that SF3B6 knockdown rescues DKFZ-induced cell proliferation. **D** Migration assay demonstrating that SF3B6 knockdown rescues DKFZ-induced cell migration. **E** RT-qPCR demonstrating that SF3B6 knockdown rescues DKFZ-induced alternative splicing of MDM-4. **A** Data are shown as mean ± SD; *P < 0.05 vs. Mock and N; **P < 0.01 vs. DKFZ. **B**–**E** Data are shown as mean ± SD; *P < 0.05, **P < 0.01. Scale bars refer to 100 μm

To study whether *DKFZp434J0226* increases cell migration and proliferation via SF3B6, we knocked down SF3B6 in *DKFZp434J0226*-overexpressed AsPC-1 and CFPAC-1 cell lines. We found that DKFZ-induced cell proliferation was rescued by SF3B6 knockdown, as evidenced by the results of the cell growth curve assay (Fig. 7A and (Additional file 2: Fig. S2A), soft agar colony formation assay (Fig. 7B and (Additional file 2: Fig. S2B), and western blotting of PCNA (Fig. 7C and (Additional file 2: Fig. S2C). In addition, SF3B6 silencing inhibited *DKFZp434J0226* overexpression-induced cell migration, as measured by migration invasion assays (Fig. 7D and (Additional file 2: Fig. S2D). Furthermore, SF3B6 silencing reduced *DKFZp434J0226* overexpression-induced alternative splicing of MDM-4 (Fig. 7E and (Additional file 2: Fig. S2E). These results indicated that SF3B6 was necessary for *DKFZp434J0226*-induced cell growth and migration.

### *DKFZp434J0226* promotes tumor growth in vivo

**Fig. 8:**
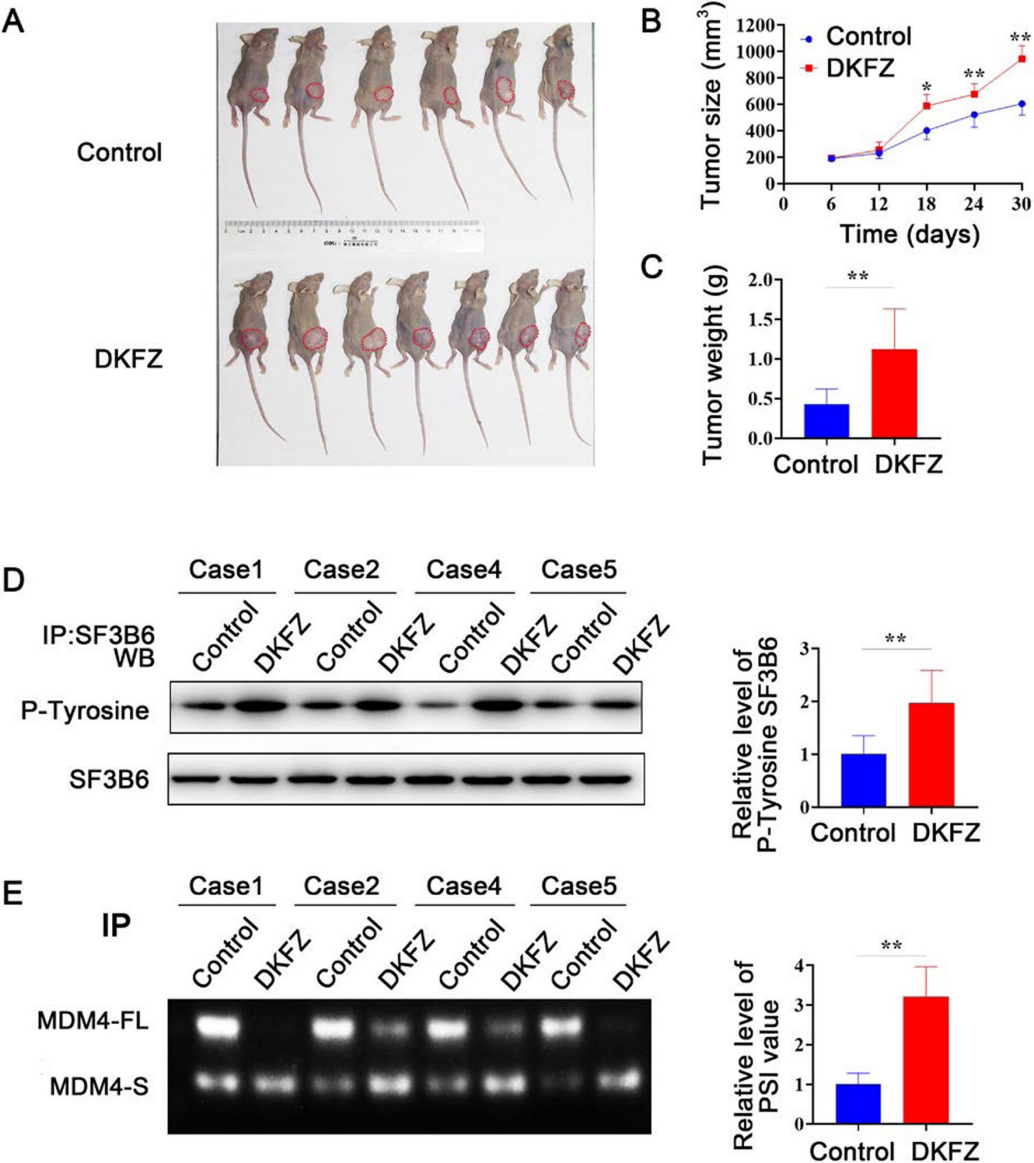
*DKFZp434J0226* (DKFZ) inhibits xenograft tumor growth. MiaPaCa-2 cells infected with control lentivirus (control, n = 6) and DKFZ lentivirus (DKFZ, n = 7) were injected subcutaneously into nude mice. **A** Tumor size. **B** In vivo subcutaneous tumor growth curves. Data are shown as mean ± SD; *P < 0.05 vs. Mock and NC; **P < 0.01 vs. control. **C** Total tumor weight of each group of mice. **D** Western blotting analysis and quantification of SF3B6 phosphorylation levels in indicated tumors (n = 4). **E** The alternative spliced transcripts (MDM4-FL, MDM4 full-length transcript; MDM4-S, MDM4 S transcript) in the indicated tumors verified by RT-qPCR. The percent spliced in index was quantified for the alternative splicing events (n = 4). **C**–**E** Data are shown as mean ± SD; *P < 0.05, **P < 0.01

To examine the biological effect of *DKFZp434J0226* in vivo, we subcutaneously injected AsPC-1 cells infected with control or DKFZp434J0226 lentiviruses into nude mice. *DKFZp434J0226* overexpression significantly promoted tumor growth, assessed by tumor size (Fig. 8A, B) and weight (Fig. 8C). These results confirmed that *DKFZp434J0226* promoted tumor growth and may have oncogenic functions in vivo. Next, we examined the phosphorylation of SF3B6 in mouse tumor tissues. Western blotting showed that the phosphorylation level of SF3B6 was greatly increased in the *DKFZp434J0226* overexpression group (Fig. 8D). Furthermore, we examined the expression of MDM4 transcripts in tumor samples. An increase in the expression of the MDM4-S transcript was observed in *DKFZp434J0226*-overexpressed tumor tissues (Fig. 8E). Together, the results provided in vivo evidence that *DKFZp434J0226* can contribute to SF3B6 phosphorylation and alternative splicing of SF3B6 downstream genes.

## Discussion

Over the past decades, several studies have shown that ncRNAs have regulatory potential both in transcription and post-transcription and play important biological roles in human diseases (Mercer et al. 2009; Wilusz et al. 2009). Furthermore, lncRNAs have been shown to be associated with a spectrum of biological processes, such as modulation of alternative splicing, protein activity, and epigenetic regulation (Sana et al. 2012; Werner et al. 2017). In this study, we studied the profiles of lncRNA expression between PDAC tissues and adjacent normal tissues using human lncRNA/mRNA expression microarray analysis. The lncRNA*DKFZp434J0226* was found to be aberrantly increased in PDAC tissues, and increased *DKFZp434J0226* expression was positively associated with poor prognosis and aggressive phenotypes in patients with PDAC. Furthermore, the study demonstrated that *DKFZp434J0226* promotes the phosphorylation and translocation of SF3B6, an important splicing factor, and modulates the alternative splicing process in PDAC cells.

Using human lncRNA/mRNA expression microarray analysis, we identified 106 differentially expressed lncRNAs and 222 aberrantly expressed mRNAs in 33,045 lncRNAs and 30,215 coding transcripts, respectively. GO analysis suggested that the upregulated mRNAs mainly aggregated in the “spliceosome,” the “metabolic pathway,” and “glycosaminoglycan and keratan sulfate.” These data are consistent with the viewpoint that PDAC is a disease of metabolic aberration and immune imbalance (Stolzenberg-Solomon et al. 2013; Matsuda et al. 2013; Sideras et al. 2013); glycosaminoglycan and keratan sulfate are important components of the extracellular matrix, which broadly participate in extracellular signal transduction and are closely associated with the metastasis and invasion of tumor cells (Shrimali et al. 2013; Heinemann et al. 2014). Aberrant expression of genes included in glycosaminoglycan and keratan sulfate biosynthesis in PDAC may provide insights into the molecular pathogenesis and may explain the highly aggressive behavior of PDAC.

The expression pattern of lncRNAs has been documented in different types of human malignant tumors (Gupta et al. 2010; Yang et al. 2011; Ji et al. 2003; Schmitt et al. 2016), but only a few studies have reported the roles of some specific lncRNAs in pancreatic cancer. Liu et al. (2014) reported that MALAT1 could be an independent predictor of disease-specific survival of PDAC (Tahira et al. 2011). They studied the role of intronic and intergenic lncRNAs in PDAC and found that loci harboring intronic lncRNAs were aberrantly expressed in PDAC metastases. We found that*DKFZp434J0226*, a 1635-bp intergenic lncRNA located in the region of chromosome 19q13.3, is upregulated in PDAC. To date, only one study has reported the function of *DKFZp434J0226*; the results showed a possible association between *DKFZp434J0226*expression and prognosis of colorectal cancer (Zhao et al. 2018). In our study, high*DKFZp434J0226* levels were positively correlated with tumor phenotypes such as tumor grading, TNM stage, and perineural invasion. Loss-and gain-of-function assays in vitro revealed that *DKFZp434J0226* promoted cell migration, invasion, and cell proliferation in PDAC cells.

Recent studies have suggested that lncRNAs could regulate alternative splicing (Wang et al. 1998; Eto et al. 2010; Misteli et al. 1997). The main mechanisms can be categorized into three types: (i) lncRNAs interact with specific splicing factors, (ii) lncRNAs bind to pre-mRNA molecules, and (iii) lncRNAs affect chromatin remodeling. A subset of lncRNAs has been reported to bind splicing factors and affect their activity by (i) regulating their post-translational modification (such as phosphorylation) or (ii) modulating their interactions with other splicing factors (Tripathi et al. 2010). Analyses have revealed that phosphorylation of SF3B1, an important member of SF3b spliceosome, is essential for splicing (Wang et al. 1998). Phosphorylated SF3B1 has been reported in nuclear structures, whereas non-phosphorylated SF3B1 has been found in the nucleoplasm, suggesting that phosphorylation of SFB1 may play a vital role in pre-mRNA splicing on chromatin concomitant with transcription (Eto et al. 2010). In addition, SR proteins and some other snRNP proteins were found to be phosphorylated to form functional spliceosomes (Misteli et al. 1997). These findings indicate that phosphorylation of some splicing factors might not only contribute to the assembly of the spliceosome but also to intranuclear transportation. Consistent with these studies, we found that*DKFZp434J0226* promotes the phosphorylation of the splicing factor SF3B6, elevates its nuclear translocation, and consequently regulates alternative splicing.

As a multiprotein complex component of the spliceosome, the splicing factor SF3B complex is necessary for branch site selection and recognition in the splicing process. It is made of seven proteins: SF3B6/p14, SF3B1/SAP155, SF3B2/SAP145, SF3B3/SAP130, SF3B4/SAP49, SF3B5 and SF3B14b (Sun and Sun 2020). In the spliceosome, SF3B proteins in contact with pre-mRNA around the branch site strengthen the U2 snRNA/BS base-pairing interaction, thereby playing a vital role in branch site recognition and splicing. Among them, SF3B6 is located near the catalytic center, associated with the first step of the splicing reaction (Will et al. 2001; Spadaccini et al. 2006), indicating that SF3B6 plays a vital role in the early splicing process (Perea et al. 2016). Although SF3B6 plays a crucial role in alternative splicing, there are only a few studies on the target pre-mRNA of SF3B6. Recently, MDM4 was identified as a target pre-mRNA of SF3B6 (Siebring-van Olst et al. 2017). Its alternative splicing product, the MDM4-S transcript, encodes a truncated Mdm4 protein with the N-terminal 114 amino acid p53-binding domain and C-terminal 26 aa residues (Rallapalli et al. 1999). Previous overexpression studies have shown that MDM4-S lacks an internal autoinhibitory sequence. Moreover, nuclear-localized MDM4-S can act as a strong p53 inhibitor; therefore, it likely functions as an oncogene (Rallapalli et al. 2003). We found that*DKFZp434J0226* increased the level of MDM4-S transcript, indicating a role of *DKFZp434J0226* in the promotion of oncogene production by regulating alternative splicing.

## Conclusions

Taken together, our results, for the first time, identified *DKFZp434J0226* as an oncogenic lncRNA in PDAC. *DKFZp434J0226* promotes oncogenesis of PDAC by interacting with SF3B6 and regulating alternative splicing.

## Supplementary Information

**Additional file 1: Table S1.** Primer sequences used in this study. **Table S2.** Clinical characteristics of 6 patients with PDAC for microarray. **Table S3.** 128 differentially expressed lncRNAs (≥ 2-fold, P < 0.05) between six PDAC samples and paired nontumor samples. **Table S4.** 281 HOX lncRNAs detected in PDAC. **Table S5.** 2341 Rinn lncRNAs detected in PDAC. **Table S6.** 1133 Enhancer lncRNAs detected in PDAC. **Table S7.** 222 differentially expressed mRNAs (≥ 2-fold, P < 0.05) between six PDAC samples and paired nontumor samples. **Table S8.** Clinical characteristics in 109 patients with pancreatic cancer. **Table S9.** Correlation between DKFZp434J0226 and clinical characteristics. **Table S10.** Univariate analysis of factors associated with survival and recurrence.

**Additional file 2: Figure S1.***DKFZp434J0226* (DKFZ) promotes PDAC cell proliferation and migration. (A, B) Verification of DKFZ knockdown (A) and DKFZ overexpression (B) efficiency in PANC-1 and CFPAC-1 cells (n = 3). Data are shown as mean ± SD; *P < 0.05, **P < 0.01. (C) Cell growth curves of lipofectamine-treated PANC-1 (Mock) cells, negative siRNA-transfected PANC-1 (NC) cells, and two DKFZ siRNA-transfected PANC-1 (si1 and si2; n = 4). Data are shown as mean ± SD; *P < 0.05 vs. Mock and NC; **P < 0.01 vs. Mock and NC. (D) Cell growth curves of control lentivirus-infected CFPAC-1 cells (control) and DKFZ lentivirus-infected CFPAC-1 cells (DKFZ) (n = 4). Data are shown as mean ± SD; *P < 0.05 vs. Mock and NC; **P < 0.01 vs. Mock and NC. (**E**) Western blotting analysis of PCNA expression in the indicated PDAC cells. (F-I) Wound healing (F, G), migration (H) assays and soft agar colony formation assay (**I**) using the indicated PDAC cells. (n = 4). (F-I) Data are shown as mean ± SD; *P < 0.05, **P < 0.01. Scale bars refer to 100 μm. Figure S2. (A–C) Cell growth curves assay (A), soft agar colony formation assay (B), and western blotting of PCNA (C) demonstrating that SF3B6 knockdown rescues DKFZ-induced cell proliferation in CFPAC-1 cells. (D) Migration assay demonstrating that SF3B6 knockdown rescues DKFZ-induced cell migration in CFPAC-1 cells. (E) RT-qPCR demonstrating that SF3B6 knockdown rescues DKFZ-induced alternative splicing of MDM-4. (A) Data are shown as mean ± SD; *P < 0.05 vs. DKFZ; **P < 0.01 vs. DKFZ. (B–E) Data are shown as mean ± SD; *P < 0.05, **P < 0.01. Scale bars refer to 100 μm.

## Data Availability

The datasets used and/or analyzed during the current study are available from the corresponding author on reasonable request.

## Abbreviations

lncRNA: Long noncoding RNA
PDAC: Pancreatic ductal adenocarcinoma
ncRNAs: Non-coding RNAs
pre-mRNA: Precursor mRNA
RT-qPCR: Quantitative real-time polymerase chain reaction
GO: Gene ontology
CNC: Coding–non-coding gene co-expression
PCR: Polymerase chain reaction
PBS: Phosphate-buffered saline
SDS-PAGE: Sodium dodecyl sulphate-polyacrylamide gel electrophoresis
ANOVA: Analysis of variance
SD: Standard deviation
T/N: Tumor/normal
HOX: Human Homeobox transcription factor
HPDE: Human pancreatic ductal epithelial
OS: Overall survival
TTP: Time to progression
CCK-8: Cell counting kit-8
RAP-MS: RNA pull-down assay combined with mass spectrometry
DE: Differentially expressed genes
RIP: RNA immunoprecipitation
IP: Immunoprecipitation
PSI: Percent spliced in index
