# Supplementary material for: The long non-coding RNA DKFZp434J0226 regulates the alternative splicing process through phosphorylation of SF3B6 in PDAC
Source: Mol Med. 2021 Aug 28;27:95. doi: 10.1186/s10020-021-00347-7 (PMC8411526; doi:10.1186/s10020-021-00347-7)
Supplement: Supplementary file 1 — Additional file 1: Table S1. Primer sequences used in this study. Table S2. Clinical characteristics of 6 patients with PDAC for microarray. Table S3. 128 differentially expressed lncRNAs (≥ 2-fold, P < 0.05) between six PDAC samples and paired nontumor samples. Table S4. 281 HOX lncRNAs detected in PDAC. Table S5. 2341 Rinn lncRNAs detected in PDAC. Table S6. 1133 Enhancer lncRNAs detected in PDAC. Table S7. 222 differentially expressed mRNAs (≥ 2-fold, P < 0.05) between six PDAC samples and paired nontumor samples. Table S8. Clinical characteristics in 109 patients with pancreatic cancer. Table S9. Correlation between DKFZp434J0226 and clinical characteristics. Table S10. Univariate analysis of factors associated with survival and recurrence. [file 10020_2021_347_MOESM1_ESM.docx]

**Table S1** Primer sequences used in this study

| **LncRNA/Gene** | **Forward chain(5’-3’)** | **Reverse chain(5’-3’)** |
| --- | --- | --- |
| DKFZp434J0226 | CGTCCTTCTGAAACCCGGAA | GTGCCAGATGAGGTGATGCT |
| MEG3 | ATGAGAGCAACCTCCTAGGGTTG  TTGTGAG | CCCGCCAGGAAGAAGNACTTG  GGTCCGG |
| MDM4-FL | AGATGCTGCTCAGACTCTCG | TGGCAGTACCCACATCCTGA |
| MDM4-S | CAGCAGGTGCGCAAGGTGAA | GCACTTTGCTGTAGTAGCAGTG |
| GAPDH | CTCTCTGCTCCTCCTGTTCGAC | TGAGCGATGTGGCTCGGCT |

**Table S2** Clinical characteristics of 6 patients with PDAC for microarray

| **Specimen code** | **Gender** | | **Ages**  **(year)** | | **Grade** | **TNM Stage** | | **Nerve invasion** | **Lymphovascular invasion** | **Vascular invasion** | |
| --- | --- | --- | --- | --- | --- | --- | --- | --- | --- | --- | --- |
| 179331 | male | 51 | | II | | | II | NO | NO | NO |  |
| 729871 | male | 56 | | III | | | II | YES | YES | NO |  |
| 732916 | male | 69 | | II | | | II | NO | NO | NO |  |
| 763143 | male | 63 | | III | | | II | YES | YES | NO |  |
| 769928 | male | 57 | | III | | | II | NO | NO | NO |  |
| 773279 | male | 68 | | II | | | II | YES | NO | NO |  |

**Table S3** 128 differentially expressed lncRNAs (≥ 2-fold, *P* < 0.05) between six PDAC samples and paired nontumor samples

| **P-value** | **Absolute fold change**  **([T1] vs [N1])** | **Regulation** | **Seqname** | | **GeneSymbol** | | **RNA**  **length** |  |
| --- | --- | --- | --- | --- | --- | --- | --- | --- |
| 0.027355522 | 2.007712802 | up | ENST00000458297 | | AC010632.1 | | 387 |  |
| 0.026347542 | 2.00908539 | up | NR_024368 | | FLJ45340 | | 10263 |  |
| 0.049363914 | 2.045834254 | up | uc001rab.1 | | BC038742 | | 1305 |  |
| 0.019558869 | 2.056364359 | up | NR_027282 | | LOC399815 | | 2112 |  |
| 0.019558869 | 2.056364359 | up | NR_027282 | | LOC399815 | | 2112 |  |
| 0.007681505 | 2.062401327 | up | AK002107 | |  | | 2714 |  |
| 0.027784187 | 2.079738311 | up | AL049314 | |  | | 1767 |  |
| 0.027784187 | 2.079738311 | up | AL049314 | |  | | 1767 |  |
| 0.027784187 | 2.079738311 | up | AL049314 | |  | | 1767 |  |
| 0.027784187 | 2.079738311 | up | AL049314 | |  | | 1767 |  |
| 0.002725453 | 2.104995911 | up | BC015064 | |  | | 575 |  |
| 0.009382136 | 2.111467724 | up | uc010wia.1 | | AK027091 | | 845 |  |
| 0.032079339 | 2.117855628 | up | ENST00000503796 | | RP11-1134I14.3 | | 351 |  |
| 0.014109777 | 2.117907212 | up | uc.209+ | | uc.209 | | 250 |  |
| 0.027158161 | 2.153623237 | up | HIT000265838 | |  | | 1451 |  |
| 0.027158161 | 2.153623237 | up | HIT000265838 | |  | | 1451 |  |
| 0.034574482 | 2.159653809 | up | ENST00000404655 | | RP1-91J24.1 | | 591 |  |
| 0.011509033 | 2.166858211 | up | ENST00000477681 | | CTD-2015H6.2 | | 438 |  |
| 0.014319646 | 2.182040886 | up | ENST00000447343 | | RP4-583P15.10 | | 464 |  |
| 0.046489089 | 2.232624016 | up | AK055024 | |  | | 2178 |  |
| 0.046489089 | 2.232624016 | up | AK055024 | |  | | 2178 |  |
| 0.046489089 | 2.232624016 | up | AK055024 | |  | | 2178 |  |
| 0.046489089 | 2.232624016 | up | AK055024 | |  | | 2178 |  |
| 0.039289298 | 2.283594635 | up | uc001pfi.1 | | AK055250 | | 1926 |  |
| 0.040583457 | 2.290892907 | up | AK128128 | |  | | 3392 |  |
| 0.040591839 | 2.302979881 | up | ENST00000428055 | | AC103563.2 | | 354 |  |
| 0.023353359 | 2.30454257 | up | AF086011 | |  | | 389 |  |
| 0.023353359 | 2.30454257 | up | AF086011 | |  | | 389 |  |
| 0.006564545 | 2.307444116 | up | BC042589 | |  | | 3223 |  |
| 0.010423263 | 2.312333698 | up | AK093981 | |  | | 2799 |  |
| 0.023366976 | 2.313236647 | up | AL049990 | |  | | 1719 |  |
| 0.048153921 | 2.327240349 | up | uc002kmd.1 | | AX721193 | | 323 |  |
| 0.032195609 | 2.353656547 | up | ENST00000507795 | | RP11-586D5.1 | | 805 |  |
| 0.028660408 | 2.370808848 | up | uc003vkf.1 | | AK057037 | | 1027 |  |
| 0.036850791 | 2.374451301 | up | BC036914 | |  | | 1397 |  |
| 0.03203993 | 2.380545024 | up | AF052107 | |  | | 1576 |  |
| 0.044986358 | 2.386323141 | up | HIT000250396 | |  | | 625 |  |
| 0.047058579 | 2.448382554 | up | AK127274 | |  | | 3833 |  |
| 0.039054745 | 2.538863151 | up | ENST00000501433 | | AC083868.1 | | 1260 |  |
| 0.041084182 | 2.590645743 | up | ENST00000486345 | | AC005205.2 | | 171 |  |
| 0.040414331 | 2.651531158 | up | HIT000325634 | |  | | 635 |  |
| 0.030415094 | 2.845446349 | up | CR624187 | |  | | 2237 |  |
| 0.037633584 | 2.908071597 | up | ENST00000429316 | | AC103563.5 | | 123 |  |
| 0.046567822 | 3.049862837 | up | ENST00000433950 | | RP11-575M4.1 | | 276 |  |
| 0.020974737 | 3.057839666 | up | ENST00000506899 | | RP11-22A3.2 | | 2694 |  |
| 0.021392759 | 3.133782618 | up | BX641027 | |  | | 4510 |  |
| 0.021392759 | 3.133782618 | up | BX641027 | |  | | 4510 |  |
| 0.003950337 | 3.27325775 | up | AK093732 | |  | | 1897 |  |
| 0.012685152 | 3.358214947 | up | AM259138 | |  | | 378 |  |
| 0.008936458 | 3.624533073 | up | BX640629 | |  | | 4365 |  |
| 0.016651537 | 4.593227454 | up | nc-HOXA11-86+ | | nc-HOXA11-86 | | 128 |  |
| 0.002899141 | 8.537781982 | up | NR_027003 | | **DKFZp434J0226** | | 1635 |  |
| 0.00629189 | 25.52537165 | up | NR_002181 | | PPY2 | | 660 |  |
| 0.021936135 | 2.015723064 | down | | NR_027391 | | SLC15A3 | 1965 | |
| 0.021936135 | 2.015723064 | down | | NR_027391 | | SLC15A3 | 1965 | |
| 0.021936135 | 2.015723064 | down | | NR_027391 | | SLC15A3 | 1965 | |
| 0.042661936 | 2.02499154 | down | | G30807 | |  | 596 | |
| 0.042661936 | 2.02499154 | down | | G30807 | |  | 596 | |
| 0.008221572 | 2.027868695 | down | | NR_029462 | | LST1 | 608 | |
| 0.008221572 | 2.027868695 | down | | NR_029462 | | LST1 | 608 | |
| 0.008221572 | 2.027868695 | down | | NR_029462 | | LST1 | 608 | |
| 0.008221572 | 2.027868695 | down | | NR_029462 | | LST1 | 608 | |
| 0.008221572 | 2.027868695 | down | | NR_029462 | | LST1 | 608 | |
| 0.008221572 | 2.027868695 | down | | NR_029462 | | LST1 | 608 | |
| 0.008221572 | 2.027868695 | down | | NR_029462 | | LST1 | 608 | |
| 0.008221572 | 2.027868695 | down | | NR_029462 | | LST1 | 608 | |
| 0.045006438 | 2.034299659 | down | | ENST00000508217 | | CTC-347C20.1 | 330 | |
| 0.002240936 | 2.035224583 | down | | uc003wns.2 | | BC034557 | 4298 | |
| 0.002240936 | 2.035224583 | down | | uc003wns.2 | | BC034557 | 4298 | |
| 0.002240936 | 2.035224583 | down | | uc003wns.2 | | BC034557 | 4298 | |
| 0.022120938 | 2.041805313 | down | | ENST00000505001 | | RP11-1023L17.2 | 3669 | |
| 0.0076563 | 2.043100975 | down | | AK026743 | |  | 3230 | |
| 0.04736773 | 2.050183449 | down | | Y00062 | | lincRNA-ATP6V1G3-2 | 4597 | |
| 0.04736773 | 2.050183449 | down | | Y00062 | | lincRNA-ATP6V1G3-2 | 4597 | |
| 0.04736773 | 2.050183449 | down | | Y00062 | | lincRNA-ATP6V1G3-2 | 4597 | |
| 0.04736773 | 2.050183449 | down | | Y00062 | | lincRNA-ATP6V1G3-2 | 4597 | |
| 0.04736773 | 2.050183449 | down | | Y00062 | | lincRNA-ATP6V1G3-2 | 4597 | |
| 0.04736773 | 2.050183449 | down | | Y00062 | | lincRNA-ATP6V1G3-2 | 4597 | |
| 0.03623592 | 2.050655542 | down | | uc003ysm.1 | | TMEM75 | 2165 | |
| 0.008381367 | 2.054993653 | down | | NR_024151 | | HSPA7 | 2493 | |
| 0.011711479 | 2.074848592 | down | | uc010fjq.2 | | LIMS3 | 2071 | |
| 0.011711479 | 2.074848592 | down | | uc010fjq.2 | | LIMS3 | 2071 | |
| 0.016554825 | 2.076576384 | down | | uc001qjy.2 | | CACNA1C | 137 | |
| 0.005994659 | 2.079679085 | down | | AK131566 | | lincRNA-NR4A1 | 3513 | |
| 0.005994659 | 2.079679085 | down | | AK131566 | | lincRNA-NR4A1 | 3513 | |
| 0.005994659 | 2.079679085 | down | | AK131566 | | lincRNA-NR4A1 | 3513 | |
| 0.043150085 | 2.080178783 | down | | ENST00000458366 | | SOCS2P2 | 574 | |
| 0.0456676 | 2.080537559 | down | | ENST00000432567 | | RP11-364B6.3 | 525 | |
| 0.038142401 | 2.107440008 | down | | AK094813 | |  | 3528 | |
| 0.001655732 | 2.108487579 | down | | ENST00000432950 | | RP11-397P14.3 | 983 | |
| 0.033896507 | 2.123386432 | down | | ENST00000305623 | | RP11-445P17.4 | 895 | |
| 0.01507445 | 2.15732788 | down | | ENST00000417884 | | RP3-340N1.2 | 1083 | |
| 0.004790751 | 2.165778109 | down | | ENST00000445535 | | RP11-25K21.4 | 1927 | |
| 0.023742262 | 2.172851381 | down | | ENST00000500445 | | AC009094.1 | 6580 | |
| 0.000450616 | 2.177045869 | down | | uc010jub.1 | | AK293020 | 375 | |
| 0.033363526 | 2.183509085 | down | | ENST00000512875 | | CTB-46B19.2 | 735 | |
| 0.033363526 | 2.183509085 | down | | ENST00000512875 | | CTB-46B19.2 | 735 | |
| 0.023240512 | 2.194605722 | down | | ENST00000501740 | | AC020926.1 | 3309 | |
| 0.006550621 | 2.252776343 | down | | NR_029461 | | LST1 | 653 | |
| 0.006550621 | 2.252776343 | down | | NR_029461 | | LST1 | 653 | |
| 0.006550621 | 2.252776343 | down | | NR_029461 | | LST1 | 653 | |
| 0.006550621 | 2.252776343 | down | | NR_029461 | | LST1 | 653 | |
| 0.006550621 | 2.252776343 | down | | NR_029461 | | LST1 | 653 | |
| 0.006550621 | 2.252776343 | down | | NR_029461 | | LST1 | 653 | |
| 0.006550621 | 2.252776343 | down | | NR_029461 | | LST1 | 653 | |
| 0.006550621 | 2.252776343 | down | | NR_029461 | | LST1 | 653 | |
| 0.017079319 | 2.375834336 | down | | ENST00000514034 | | CTC-251I16.1 | 1154 | |
| 0.003742522 | 2.405015744 | down | | NR_027143 | | LOC440895 | 871 | |
| 0.014568645 | 2.440052461 | down | | ENST00000507684 | | TNXA | 2038 | |
| 0.014568645 | 2.440052461 | down | | ENST00000507684 | | TNXA | 2038 | |
| 0.014568645 | 2.440052461 | down | | ENST00000507684 | | TNXA | 2038 | |
| 0.004553982 | 2.513662664 | down | | AK095069 | |  | 2108 | |
| 0.004553982 | 2.513662664 | down | | AK095069 | |  | 2108 | |
| 0.004553982 | 2.513662664 | down | | AK095069 | |  | 2108 | |
| 0.014981766 | 2.54597721 | down | | ENST00000503900 | | RP11-524L6.1 | 1303 | |
| 0.016470553 | 2.583981723 | down | | ENST00000504996 | | RP11-429B11.1 | 1530 | |
| 0.025904341 | 2.661210832 | down | | AX721088 | |  | 1642 | |
| 0.019271355 | 2.874749183 | down | | ENST00000504765 | | CTD-2296D1.3 | 407 | |
| 0.006525828 | 3.09682014 | down | | NR_002798 | | NAPSB | 1398 | |
| 0.000284945 | 3.501363365 | down | | NR_027484 | | FCGR1C | 1338 | |
| 0.000244972 | 4.144055141 | down | | uc009wkz.1 | | FCGR1C | 566 | |
| 0.037403307 | 4.256064134 | down | | NR_028412 | | APOC1P1 | 939 | |
| 0.000144639 | 4.339432354 | down | | BC015134 | |  | 1142 | |
| 0.048336856 | 5.242499674 | down | | ENST00000499752 | | AC091013.1 | 3722 | |
| 0.048336856 | 5.242499674 | down | | ENST00000499752 | | AC091013.1 | 3722 | |
| 0.048336856 | 5.242499674 | down | | ENST00000499752 | | AC091013.1 | 3722 | |
| 0.048336856 | 5.242499674 | down | | ENST00000499752 | | AC091013.1 | 3722 | |
| 0.001692348 | 5.391459584 | down | | NR_002712 | | CXCR2P1 | 2153 | |
|  |  |  | |  | |  |  | |

There are upregulation of 53 lncRNAs and downregulation of 75 lncRNAs respectively. Bold indicates lncRNA DKFZp434J0226.

**Table S4** 281 HOX lncRNAs detected in PDAC

| **Fold change** | **Seqname** | **GeneSymbol** | **Source** | **RNA**  **length** |
| --- | --- | --- | --- | --- |
| 0.3489369 | nc-HOXC8-145+ | nc-HOXC8-145 | HOX cluster | 308 |
| 0.4153929 | nc-HOXC10-122+ | nc-HOXC10-122 | HOX cluster | 108 |
| 0.4269838 | nc-HOXC6-245+ | nc-HOXC6-245 | HOX cluster | 218 |
| 0.433404 | nc-HOXC10-129+ | nc-HOXC10-129 | HOX cluster | 260 |
| 0.5049986 | nc-HOXA6-69+ | nc-HOXA6-69 | HOX cluster | 128 |
| 0.5797465 | nc-HOXD10-11+ | nc-HOXD10-11 | HOX cluster | 118 |
| 0.5947788 | nc-HOXB2-160+ | nc-HOXB2-160 | HOX cluster | 310 |
| 0.6390158 | nc-HOXB9-189+ | nc-HOXB9-189 | HOX cluster | 138 |
| 0.6693314 | nc-HOXA13-97+ | nc-HOXA13-97 | HOX cluster | 348 |
| 0.6878905 | nc-HOXB9-205- | nc-HOXB9-205 | HOX cluster | 838 |
| 0.696677 | nc-HOXB4-167+ | nc-HOXB4-167 | HOX cluster | 348 |
| 0.7050763 | nc-HOXD3-42+ | nc-HOXD3-42 | HOX cluster | 178 |
| 0.7063882 | nc-HOXB9-186- | nc-HOXB9-186 | HOX cluster | 168 |
| 0.7113254 | nc-HOXB4-166+ | nc-HOXB4-166 | HOX cluster | 263 |
| 0.7124155 | nc-HOXA4-66+ | nc-HOXA4-66 | HOX cluster | 307 |
| 0.7194635 | nc-HOXA11-88+ | nc-HOXA11-88 | HOX cluster | 278 |
| 0.729567 | nc-HOXC10-124- | nc-HOXC10-124 | HOX cluster | 248 |
| 0.730486 | nc-HOXA7-75+ | nc-HOXA7-75 | HOX cluster | 108 |
| 0.732902 | nc-HOXD12-3- | nc-HOXD12-3 | HOX cluster | 858 |
| 0.734237 | nc-HOXA9-79- | nc-HOXA9-79 | HOX cluster | 248 |
| 0.736161 | ENST00000513381 | RP11-834C11.5 | Ensembl | 499 |
| 0.7386001 | ENST00000480386 | RP11-357H14.4 | Ensembl | 566 |
| 0.741746 | nc-HOXC10-125+ | nc-HOXC10-125 | HOX cluster | 128 |
| 0.7420756 | nc-HOXB4-172+ | nc-HOXB4-172 | HOX cluster | 148 |
| 0.7457614 | nc-HOXC10-118+ | nc-HOXC10-118 | HOX cluster | 118 |
| 0.7461604 | nc-HOXB6-180- | nc-HOXB6-180 | HOX cluster | 108 |
| 0.7481099 | nc-HOXC10-119- | nc-HOXC10-119 | HOX cluster | 138 |
| 0.7525826 | nc-HOXC10-123+ | nc-HOXC10-123 | HOX cluster | 148 |
| 0.7802951 | nc-HOXC11-110+ | nc-HOXC11-110 | HOX cluster | 838 |
| 0.7838015 | nc-HOXB2-162+ | nc-HOXB2-162 | HOX cluster | 178 |
| 0.7840126 | nc-HOXA11-89+ | nc-HOXA11-89 | HOX cluster | 478 |
| 0.7852282 | nc-HOXB5-179- | nc-HOXB5-179 | HOX cluster | 101 |
| 0.7884795 | nc-HOXB8-183+ | nc-HOXB8-183 | HOX cluster | 158 |
| 0.7908607 | nc-HOXB4-175- | nc-HOXB4-175 | HOX cluster | 148 |
| 0.7917252 | nc-HOXC9-133+ | nc-HOXC9-133 | HOX cluster | 108 |
| 0.793461 | ENST00000440016 | AC009336.21 | Ensembl | 582 |
| 0.7963888 | nc-HOXA1-58- | nc-HOXA1-58 | HOX cluster | 148 |
| 0.7974076 | nc-HOXC6-248+ | nc-HOXC6-248 | HOX cluster | 948 |
| 0.7978206 | nc-HOXB4-169+ | nc-HOXB4-169 | HOX cluster | 1198 |
| 0.8051206 | nc-HOXC11-107- | nc-HOXC11-107 | HOX cluster | 148 |
| 0.8060357 | nc-HOXC9-141- | nc-HOXC9-141 | HOX cluster | 478 |
| 0.8077663 | nc-HOXD8-21- | nc-HOXD8-21 | HOX cluster | 348 |
| 0.8096365 | nc-HOXD9-17+ | nc-HOXD9-17 | HOX cluster | 168 |
| 0.8121761 | nc-HOXA13-96+ | nc-HOXA13-96 | HOX cluster | 318 |
| 0.8137924 | nc-HOXB2-162- | nc-HOXB2-162 | HOX cluster | 178 |
| 0.814917 | ENST00000405359 | AC016739.2 | Ensembl | 345 |
| 0.8149358 | nc-HOXD11-4+ | nc-HOXD11-4 | HOX cluster | 488 |
| 0.8154311 | nc-HOXC10-126- | nc-HOXC10-126 | HOX cluster | 228 |
| 0.818243 | nc-HOXC11-114- | nc-HOXC11-114 | HOX cluster | 128 |
| 0.8249089 | nc-HOXA7-75- | nc-HOXA7-75 | HOX cluster | 108 |
| 0.8259124 | nc-HOXA6-71- | nc-HOXA6-71 | HOX cluster | 218 |
| 0.8275955 | nc-HOXB9-201+ | nc-HOXB9-201 | HOX cluster | 238 |
| 0.8295362 | nc-HOXC10-126+ | nc-HOXC10-126 | HOX cluster | 228 |
| 0.8309445 | nc-HOXA11-83+ | nc-HOXA11-83 | HOX cluster | 223 |
| 0.8321131 | nc-HOXA2-60- | nc-HOXA2-60 | HOX cluster | 148 |
| 0.8325352 | nc-HOXD1-48- | nc-HOXD1-48 | HOX cluster | 138 |
| 0.8386328 | nc-HOXD10-14- | nc-HOXD10-14 | HOX cluster | 258 |
| 0.8401576 | nc-HOXD11-5+ | nc-HOXD11-5 | HOX cluster | 228 |
| 0.8404372 | nc-HOXC6-238+ | nc-HOXC6-238 | HOX cluster | 238 |
| 0.8409236 | nc-HOXC12-104+ | nc-HOXC12-104 | HOX cluster | 148 |
| 0.8416419 | nc-HOXC9-135+ | nc-HOXC9-135 | HOX cluster | 138 |
| 0.8428497 | nc-HOXB9-192- | nc-HOXB9-192 | HOX cluster | 318 |
| 0.8429226 | nc-HOXC4-153+ | nc-HOXC4-153 | HOX cluster | 519 |
| 0.8448981 | nc-HOXD4-33+ | nc-HOXD4-33 | HOX cluster | 208 |
| 0.8467924 | nc-HOXC10-127- | nc-HOXC10-127 | HOX cluster | 248 |
| 0.8468556 | nc-HOXA7-74+ | nc-HOXA7-74 | HOX cluster | 238 |
| 0.8478315 | nc-HOXB2-161- | nc-HOXB2-161 | HOX cluster | 418 |
| 0.8479968 | nc-HOXC9-142+ | nc-HOXC9-142 | HOX cluster | 400 |
| 0.8499502 | NR_026655 | FLJ12825 | RefSeq_NR | 3942 |
| 0.85031 | nc-HOXD3-44- | nc-HOXD3-44 | HOX cluster | 98 |
| 0.8592468 | nc-HOXB9-185- | nc-HOXB9-185 | HOX cluster | 148 |
| 0.8592793 | nc-HOXA1-54+ | nc-HOXA1-54 | HOX cluster | 128 |
| 0.8604527 | nc-HOXD3-41+ | nc-HOXD3-41 | HOX cluster | 128 |
| 0.8623531 | nc-HOXD4-35+ | nc-HOXD4-35 | HOX cluster | 188 |
| 0.8637388 | nc-HOXC10-123- | nc-HOXC10-123 | HOX cluster | 148 |
| 0.864362 | nc-HOXD1-49- | nc-HOXD1-49 | HOX cluster | 108 |
| 0.8646721 | nc-HOXC6-245- | nc-HOXC6-245 | HOX cluster | 218 |
| 0.8650919 | nc-HOXB1-156+ | nc-HOXB1-156 | HOX cluster | 128 |
| 0.8662541 | nc-HOXA1-58+ | nc-HOXA1-58 | HOX cluster | 148 |
| 0.8685349 | nc-HOXB9-188+ | nc-HOXB9-188 | HOX cluster | 98 |
| 0.8694931 | nc-HOXC5-256- | nc-HOXC5-256 | HOX cluster | 128 |
| 0.8696255 | nc-HOXC9-132- | nc-HOXC9-132 | HOX cluster | 148 |
| 0.8729193 | nc-HOXD4-29- | nc-HOXD4-29 | HOX cluster | 288 |
| 0.8736315 | nc-HOXD4-31+ | nc-HOXD4-31 | HOX cluster | 108 |
| 0.8756765 | nc-HOXC6-247- | nc-HOXC6-247 | HOX cluster | 138 |
| 0.8810042 | nc-HOXB9-188- | nc-HOXB9-188 | HOX cluster | 98 |
| 0.8811944 | nc-HOXA13-101- | nc-HOXA13-101 | HOX cluster | 138 |
| 0.8815533 | nc-HOXC9-131+ | nc-HOXC9-131 | HOX cluster | 198 |
| 0.8818441 | nc-HOXD4-25- | nc-HOXD4-25 | HOX cluster | 228 |
| 0.8825106 | nc-HOXA10-82+ | nc-HOXA10-82 | HOX cluster | 370 |
| 0.8842473 | nc-HOXD4-25+ | nc-HOXD4-25 | HOX cluster | 228 |
| 0.8877806 | nc-HOXC6-240+ | nc-HOXC6-240 | HOX cluster | 288 |
| 0.8909496 | nc-HOXC6-239- | nc-HOXC6-239 | HOX cluster | 538 |
| 0.8920031 | nc-HOXA6-70- | nc-HOXA6-70 | HOX cluster | 178 |
| 0.8937126 | nc-HOXD9-17- | nc-HOXD9-17 | HOX cluster | 168 |
| 0.8953337 | nc-HOXD10-10- | nc-HOXD10-10 | HOX cluster | 128 |
| 0.8966931 | nc-HOXD4-28- | nc-HOXD4-28 | HOX cluster | 248 |
| 0.8989236 | nc-HOXC11-106- | nc-HOXC11-106 | HOX cluster | 158 |
| 0.9016399 | NR_024103 | C17orf93 | RefSeq_NR | 564 |
| 0.9033701 | nc-HOXD4-30- | nc-HOXD4-30 | HOX cluster | 108 |
| 0.9070636 | nc-HOXA1-56- | nc-HOXA1-56 | HOX cluster | 318 |
| 0.9071343 | nc-HOXB9-185+ | nc-HOXB9-185 | HOX cluster | 148 |
| 0.9088388 | nc-HOXC12-105- | nc-HOXC12-105 | HOX cluster | 188 |
| 0.9105918 | nc-HOXD10-9+ | nc-HOXD10-9 | HOX cluster | 178 |
| 0.9114115 | nc-HOXB4-168- | nc-HOXB4-168 | HOX cluster | 1808 |
| 0.9116536 | ENST00000455246 | HOTAIR | Ensembl | 918 |
| 0.9131368 | nc-HOXB2-163- | nc-HOXB2-163 | HOX cluster | 208 |
| 0.9148219 | nc-HOXC10-121- | nc-HOXC10-121 | HOX cluster | 248 |
| 0.9158712 | nc-HOXC9-135- | nc-HOXC9-135 | HOX cluster | 138 |
| 0.917757 | nc-HOXC6-251- | nc-HOXC6-251 | HOX cluster | 278 |
| 0.9184549 | nc-HOXB4-176- | nc-HOXB4-176 | HOX cluster | 158 |
| 0.9185791 | nc-HOXC13-102- | nc-HOXC13-102 | HOX cluster | 108 |
| 0.9200012 | nc-HOXA11-91+ | nc-HOXA11-91 | HOX cluster | 118 |
| 0.9228938 | nc-HOXB1-159+ | nc-HOXB1-159 | HOX cluster | 608 |
| 0.9236811 | nc-HOXC10-119+ | nc-HOXC10-119 | HOX cluster | 138 |
| 0.9253082 | nc-HOXB13-207- | nc-HOXB13-207 | HOX cluster | 108 |
| 0.9303798 | nc-HOXD3-45+ | nc-HOXD3-45 | HOX cluster | 618 |
| 0.9312316 | nc-HOXC10-129- | nc-HOXC10-129 | HOX cluster | 260 |
| 0.931919 | nc-HOXB6-181+ | nc-HOXB6-181 | HOX cluster | 278 |
| 0.9323875 | nc-HOXA4-64+ | nc-HOXA4-64 | HOX cluster | 138 |
| 0.932775 | nc-7-52- | nc-7-52 | HOX cluster | 148 |
| 0.9334935 | nc-HOXD10-15+ | nc-HOXD10-15 | HOX cluster | 130 |
| 0.9340689 | nc-HOXA6-70+ | nc-HOXA6-70 | HOX cluster | 178 |
| 0.9356613 | nc-HOXB9-204+ | nc-HOXB9-204 | HOX cluster | 198 |
| 0.936869 | nc-HOXC6-351- | nc-HOXC6-351 | HOX cluster | 875 |
| 0.938591 | uc002inu.2 | LOC404266 | UCSC_knowngene | 427 |
| 0.9390163 | nc-HOXC10-128+ | nc-HOXC10-128 | HOX cluster | 158 |
| 0.9393723 | uc003syg.2 | BC031342 | UCSC_knowngene | 721 |
| 0.940127 | nc-HOXD4-22- | nc-HOXD4-22 | HOX cluster | 706 |
| 0.941061 | nc-HOXA6-72- | nc-HOXA6-72 | HOX cluster | 1151 |
| 0.9411289 | nc-HOXC6-247+ | nc-HOXC6-247 | HOX cluster | 138 |
| 0.9412032 | nc-HOXC11-108+ | nc-HOXC11-108 | HOX cluster | 2358 |
| 0.9421966 | nc-HOXA13-95- | nc-HOXA13-95 | HOX cluster | 218 |
| 0.9478478 | nc-HOXA1-53- | nc-HOXA1-53 | HOX cluster | 138 |
| 0.9482672 | ENST00000417086 | AC009336.23 | Ensembl | 984 |
| 0.9506714 | nc-HOXB9-189- | nc-HOXB9-189 | HOX cluster | 138 |
| 0.9532074 | nc-HOXA4-62+ | nc-HOXA4-62 | HOX cluster | 148 |
| 0.9547707 | nc-HOXA4-63- | nc-HOXA4-63 | HOX cluster | 108 |
| 0.956231 | nc-HOXD10-12- | nc-HOXD10-12 | HOX cluster | 158 |
| 0.9589524 | nc-HOXB4-173+ | nc-HOXB4-173 | HOX cluster | 138 |
| 0.9607986 | NR_033201 | LOC404266 | RefSeq_NR | 570 |
| 0.9619642 | nc-HOXD4-26+ | nc-HOXD4-26 | HOX cluster | 1178 |
| 0.9622709 | nc-HOXB1-159- | nc-HOXB1-159 | HOX cluster | 608 |
| 0.9654016 | ENST00000489624 | COX6B1P2 | Ensembl | 216 |
| 0.966523 | ENST00000513533 | AC012531.16 | Ensembl | 504 |
| 0.9683823 | nc-HOXC4-264+ | nc-HOXC4-264 | HOX cluster | 768 |
| 0.9697457 | nc-HOXD4-36- | nc-HOXD4-36 | HOX cluster | 262 |
| 0.973008 | nc-HOXC8-149- | nc-HOXC8-149 | HOX cluster | 228 |
| 0.9744813 | nc-HOXC6-252+ | nc-HOXC6-252 | HOX cluster | 442 |
| 0.9751125 | nc-HOXC11-116- | nc-HOXC11-116 | HOX cluster | 228 |
| 0.9788901 | nc-HOXC11-111+ | nc-HOXC11-111 | HOX cluster | 698 |
| 0.9813487 | nc-HOXA9-81+ | nc-HOXA9-81 | HOX cluster | 198 |
| 0.9828759 | nc-HOXC11-115+ | nc-HOXC11-115 | HOX cluster | 298 |
| 0.9832721 | nc-HOXD4-31- | nc-HOXD4-31 | HOX cluster | 108 |
| 0.9852808 | nc-HOXB9-184- | nc-HOXB9-184 | HOX cluster | 198 |
| 0.9879097 | nc-HOXB4-174+ | nc-HOXB4-174 | HOX cluster | 338 |
| 0.9884345 | nc-HOXC6-250+ | nc-HOXC6-250 | HOX cluster | 1518 |
| 0.9915645 | nc-HOXC12-103- | nc-HOXC12-103 | HOX cluster | 148 |
| 0.9917359 | nc-HOXD4-34+ | nc-HOXD4-34 | HOX cluster | 158 |
| 0.9922811 | nc-HOXA7-73+ | nc-HOXA7-73 | HOX cluster | 148 |
| 0.9941409 | nc-HOXC4-152- | nc-HOXC4-152 | HOX cluster | 229 |
| 0.9957884 | nc-HOXC9-130- | nc-HOXC9-130 | HOX cluster | 151 |
| 0.9976857 | nc-HOXB4-167- | nc-HOXB4-167 | HOX cluster | 348 |
| 0.9997746 | nc-HOXC5-253- | nc-HOXC5-253 | HOX cluster | 278 |
| 1.0002685 | nc-HOXB9-200+ | nc-HOXB9-200 | HOX cluster | 198 |
| 1.0003427 | nc-HOXA4-63+ | nc-HOXA4-63 | HOX cluster | 108 |
| 1.0009072 | nc-HOXD4-23+ | nc-HOXD4-23 | HOX cluster | 178 |
| 1.0032195 | nc-HOXC11-109- | nc-HOXC11-109 | HOX cluster | 1088 |
| 1.0042525 | nc-HOXA11-85- | nc-HOXA11-85 | HOX cluster | 1838 |
| 1.0043614 | nc-HOXB9-187- | nc-HOXB9-187 | HOX cluster | 168 |
| 1.0043616 | nc-HOXD10-8+ | nc-HOXD10-8 | HOX cluster | 1788 |
| 1.0066637 | nc-HOXB9-192+ | nc-HOXB9-192 | HOX cluster | 318 |
| 1.009222 | nc-HOXD10-13+ | nc-HOXD10-13 | HOX cluster | 668 |
| 1.0093296 | nc-HOXB4-175+ | nc-HOXB4-175 | HOX cluster | 148 |
| 1.0105289 | nc-HOXC9-132+ | nc-HOXC9-132 | HOX cluster | 148 |
| 1.0111271 | nc-HOXA1-55+ | nc-HOXA1-55 | HOX cluster | 328 |
| 1.0123333 | nc-HOXA1-59+ | nc-HOXA1-59 | HOX cluster | 1265 |
| 1.0125487 | nc-HOXB9-202- | nc-HOXB9-202 | HOX cluster | 108 |
| 1.0135918 | nc-HOXC6-243+ | nc-HOXC6-243 | HOX cluster | 958 |
| 1.0148755 | nc-HOXC6-244+ | nc-HOXC6-244 | HOX cluster | 1488 |
| 1.0157183 | NR_033204 | LOC404266 | RefSeq_NR | 486 |
| 1.0172402 | nc-HOXB9-204- | nc-HOXB9-204 | HOX cluster | 198 |
| 1.019539 | nc-HOXD4-32+ | nc-HOXD4-32 | HOX cluster | 118 |
| 1.0195412 | nc-HOXC9-137+ | nc-HOXC9-137 | HOX cluster | 558 |
| 1.0197161 | nc-HOXA6-71+ | nc-HOXA6-71 | HOX cluster | 218 |
| 1.0198648 | nc-HOXA2-60+ | nc-HOXA2-60 | HOX cluster | 148 |
| 1.0227129 | nc-HOXA9-77+ | nc-HOXA9-77 | HOX cluster | 678 |
| 1.0270835 | nc-HOXA4-61+ | nc-HOXA4-61 | HOX cluster | 158 |
| 1.0273922 | nc-HOXB9-199- | nc-HOXB9-199 | HOX cluster | 188 |
| 1.0282816 | nc-HOXB4-173- | nc-HOXB4-173 | HOX cluster | 138 |
| 1.0286275 | nc-HOXA9-81- | nc-HOXA9-81 | HOX cluster | 198 |
| 1.0286594 | nc-HOXC10-121+ | nc-HOXC10-121 | HOX cluster | 248 |
| 1.0305249 | nc-HOXB3-165+ | nc-HOXB3-165 | HOX cluster | 346 |
| 1.0326008 | ENST00000434063 | HOTAIRM1 | Ensembl | 659 |
| 1.0347192 | nc-HOXA7-73- | nc-HOXA7-73 | HOX cluster | 148 |
| 1.0347448 | nc-HOXA7-74- | nc-HOXA7-74 | HOX cluster | 238 |
| 1.0367731 | ENST00000461606 | CTD-2377D24.1 | Ensembl | 579 |
| 1.0416282 | nc-HOXD8-21+ | nc-HOXD8-21 | HOX cluster | 348 |
| 1.0416362 | nc-HOXB9-197+ | nc-HOXB9-197 | HOX cluster | 198 |
| 1.0429248 | nc-HOXC10-117+ | nc-HOXC10-117 | HOX cluster | 238 |
| 1.04339 | nc-HOXC10-120- | nc-HOXC10-120 | HOX cluster | 208 |
| 1.0462482 | nc-HOXC8-150+ | nc-HOXC8-150 | HOX cluster | 723 |
| 1.0509579 | nc-HOXC8-146+ | nc-HOXC8-146 | HOX cluster | 108 |
| 1.0513418 | nc-HOXD3-43- | nc-HOXD3-43 | HOX cluster | 368 |
| 1.0521274 | nc-HOXB9-187+ | nc-HOXB9-187 | HOX cluster | 168 |
| 1.0522381 | nc-HOXC9-138- | nc-HOXC9-138 | HOX cluster | 148 |
| 1.0555945 | nc-HOXD13-2+ | nc-HOXD13-2 | HOX cluster | 188 |
| 1.0570402 | nc-HOXD10-14+ | nc-HOXD10-14 | HOX cluster | 258 |
| 1.0584188 | nc-HOXB9-184+ | nc-HOXB9-184 | HOX cluster | 198 |
| 1.0622234 | nc-HOXA1-53+ | nc-HOXA1-53 | HOX cluster | 138 |
| 1.0653174 | nc-HOXD3-44+ | nc-HOXD3-44 | HOX cluster | 98 |
| 1.0656031 | nc-HOXD4-33- | nc-HOXD4-33 | HOX cluster | 208 |
| 1.0675579 | nc-HOXD4-27- | nc-HOXD4-27 | HOX cluster | 398 |
| 1.0701914 | nc-HOXA4-65- | nc-HOXA4-65 | HOX cluster | 178 |
| 1.0779246 | ENST00000495536 | CTD-2377D24.4 | Ensembl | 532 |
| 1.0798164 | nc-HOXB9-195+ | nc-HOXB9-195 | HOX cluster | 138 |
| 1.0853495 | nc-HOXC10-120+ | nc-HOXC10-120 | HOX cluster | 208 |
| 1.0963314 | nc-HOXB4-171+ | nc-HOXB4-171 | HOX cluster | 1148 |
| 1.1088319 | nc-HOXA4-65+ | nc-HOXA4-65 | HOX cluster | 178 |
| 1.1098212 | nc-HOXC8-147+ | nc-HOXC8-147 | HOX cluster | 208 |
| 1.1126778 | nc-HOXD10-11- | nc-HOXD10-11 | HOX cluster | 118 |
| 1.1161191 | nc-HOXB9-203+ | nc-HOXB9-203 | HOX cluster | 118 |
| 1.1165625 | nc-HOXD9-16+ | nc-HOXD9-16 | HOX cluster | 558 |
| 1.1230943 | nc-HOXC8-143+ | nc-HOXC8-143 | HOX cluster | 298 |
| 1.1243162 | nc-HOXD8-19- | nc-HOXD8-19 | HOX cluster | 160 |
| 1.12613 | nc-HOXC9-139- | nc-HOXC9-139 | HOX cluster | 148 |
| 1.1322612 | nc-HOXB13-207+ | nc-HOXB13-207 | HOX cluster | 108 |
| 1.1361581 | nc-HOXC5-255- | nc-HOXC5-255 | HOX cluster | 468 |
| 1.1412888 | nc-HOXA1-57+ | nc-HOXA1-57 | HOX cluster | 458 |
| 1.1461465 | nc-HOXB9-193- | nc-HOXB9-193 | HOX cluster | 858 |
| 1.149426 | nc-HOXA11-90- | nc-HOXA11-90 | HOX cluster | 168 |
| 1.1588005 | nc-HOXC9-142- | nc-HOXC9-142 | HOX cluster | 400 |
| 1.163314 | nc-HOXC11-116+ | nc-HOXC11-116 | HOX cluster | 228 |
| 1.1644678 | nc-HOXC9-131- | nc-HOXC9-131 | HOX cluster | 198 |
| 1.1651251 | nc-HOXB9-199+ | nc-HOXB9-199 | HOX cluster | 188 |
| 1.1726494 | nc-HOXC8-150- | nc-HOXC8-150 | HOX cluster | 723 |
| 1.1778093 | nc-HOXA4-61- | nc-HOXA4-61 | HOX cluster | 158 |
| 1.1786999 | nc-HOXC5-254+ | nc-HOXC5-254 | HOX cluster | 128 |
| 1.1800139 | NR_033203 | LOC404266 | RefSeq_NR | 452 |
| 1.1828228 | uc002uku.2 | BC047481 | UCSC_knowngene | 4061 |
| 1.1855571 | nc-HOXA13-100+ | nc-HOXA13-100 | HOX cluster | 916 |
| 1.1900939 | nc-HOXD3-41- | nc-HOXD3-41 | HOX cluster | 128 |
| 1.1908595 | nc-HOXC10-128- | nc-HOXC10-128 | HOX cluster | 158 |
| 1.1923041 | nc-HOXD3-37- | nc-HOXD3-37 | HOX cluster | 168 |
| 1.1943268 | nc-HOXB9-186+ | nc-HOXB9-186 | HOX cluster | 168 |
| 1.1983565 | nc-HOXC9-133- | nc-HOXC9-133 | HOX cluster | 108 |
| 1.229722 | uc002ukl.1 | AX747372 | UCSC_knowngene | 641 |
| 1.2310804 | nc-HOXC4-263+ | nc-HOXC4-263 | HOX cluster | 152 |
| 1.2337742 | nc-HOXC6-251+ | nc-HOXC6-251 | HOX cluster | 278 |
| 1.2370623 | nc-HOXC9-140- | nc-HOXC9-140 | HOX cluster | 938 |
| 1.2466507 | nc-HOXB9-193+ | nc-HOXB9-193 | HOX cluster | 858 |
| 1.2532074 | nc-HOXB2-160- | nc-HOXB2-160 | HOX cluster | 310 |
| 1.2537306 | nc-HOXB2-164- | nc-HOXB2-164 | HOX cluster | 1039 |
| 1.2636503 | nc-HOXC9-139+ | nc-HOXC9-139 | HOX cluster | 148 |
| 1.2657097 | ENST00000297027 | AC010990.1 | Ensembl | 1962 |
| 1.2738423 | nc-HOXB4-177- | nc-HOXB4-177 | HOX cluster | 108 |
| 1.2829217 | ENST00000474761 | CTD-2377D24.2 | Ensembl | 144 |
| 1.2944757 | nc-HOXA1-57- | nc-HOXA1-57 | HOX cluster | 458 |
| 1.3056169 | nc-HOXD13-2- | nc-HOXD13-2 | HOX cluster | 188 |
| 1.3128989 | ENST00000426615 | AC009336.24 | Ensembl | 658 |
| 1.3182471 | nc-HOXB2-164+ | nc-HOXB2-164 | HOX cluster | 1039 |
| 1.3245192 | nc-HOXD1-47+ | nc-HOXD1-47 | HOX cluster | 1188 |
| 1.3294619 | nc-HOXC10-118- | nc-HOXC10-118 | HOX cluster | 118 |
| 1.3766093 | uc002ukq.2 | BC034000 | UCSC_knowngene | 690 |
| 1.393796 | nc-HOXB9-196+ | nc-HOXB9-196 | HOX cluster | 208 |
| 1.4386274 | nc-HOXA11-91- | nc-HOXA11-91 | HOX cluster | 118 |
| 1.4995487 | nc-HOXD1-48+ | nc-HOXD1-48 | HOX cluster | 138 |
| 1.6026342 | nc-HOXA13-96- | nc-HOXA13-96 | HOX cluster | 318 |
| 1.6350056 | NR_003716 | HOTAIR | RefSeq_NR | 2337 |
| 1.694564 | nc-HOXA9-77- | nc-HOXA9-77 | HOX cluster | 678 |
| 1.7324157 | nc-HOXA13-98+ | nc-HOXA13-98 | HOX cluster | 208 |
| 1.7387625 | nc-HOXC6-246+ | nc-HOXC6-246 | HOX cluster | 968 |
| 1.7426486 | nc-HOXA4-66- | nc-HOXA4-66 | HOX cluster | 307 |
| 1.8147338 | AK000839 | misc_RNA | misc_RNA | 1951 |
| 1.8152593 | nc-HOXC6-241+ | nc-HOXC6-241 | HOX cluster | 208 |
| 1.8622673 | ENST00000478824 | CTD-2377D24.6 | Ensembl | 1436 |
| 1.9970622 | nc-HOXB9-203- | nc-HOXB9-203 | HOX cluster | 118 |
| **2.1327239** | **Z49995** | **RNAdb** | **RNAdb** | **2615** |
| **2.2264481** | **nc-HOXC6-244-** | **nc-HOXC6-244** | **HOX cluster** | **1488** |
| **3.070106** | **HIT000098379 H-invDB** | **H-invDB** | **227** |  |
| **4.5932275** | **nc-HOXA11-86+** | **nc-HOXA11-86** | **HOX cluster** | **128** |
|  |  |  |  |  |
| The bold indicates 4 differently expressed lncRNAs (≥ 2-fold, *P* < 0.05) between six PDAC samples and paired nontumor samples. | | | | |

**Table S5** 2341 Rinn lncRNAs detected in PDAC

| **Fold change** | **Seqname** | **GeneSymbol** | **Source** | **RNA**  **length** |
| --- | --- | --- | --- | --- |
| 0.2756167 | ENST00000455473 | AC008280.3 | Ensembl | 307 |
| 0.3326498 | ENST00000493458 | AC009120.3 | Ensembl | 463 |
| 0.3531964 | ENST00000415709 | CRYBB2P1 | Ensembl | 538 |
| 0.3938607 | uc001zks.1 | AK127696 | UCSC_knowngene | 2317 |
| 0.4258089 | uc001yjz.1 | AK094562 | UCSC_knowngene | 2638 |
| 0.4300229 | BF589839 | lincRNA-MMP7 | lincRNA | 479 |
| 0.4326877 | uc003dml.2 | BC040632 | UCSC_knowngene | 2179 |
| 0.4557025 | AW179216 | lincRNA-SCARB1 | lincRNA | 597 |
| 0.4658515 | BC017340 | lincRNA-TGM3 | lincRNA | 1615 |
| 0.4686026 | BG273004 | lincRNA-CD83-1 | lincRNA | 332 |
| 0.4749159 | ENST00000373171 | RP11-552E20.3 | Ensembl | 554 |
| 0.4808434 | AK131566 | lincRNA-NR4A1 | lincRNA | 3513 |
| 0.4851962 | BC037953 |  | misc_RNA | 1378 |
| 0.4853233 | NR_003587 | MYO15B | RefSeq_NR | 9424 |
| 0.4877612 | Y00062 | lincRNA-ATP6V1G3-2 | lincRNA | 4597 |
| 0.4943965 | AI307480 | lincRNA-PTPRC-5 | lincRNA | 186 |
| 0.4989467 | uc004bcw.2 | BC039487 | UCSC_knowngene | 1005 |
| 0.5039591 | exon847- | lincRNA-TMPRSS5 | lincRNA | 440 |
| 0.5066301 | AK304777 | lincRNA-FBLN2-2 | lincRNA | 3873 |
| 0.5076317 | chr18:46516702-46521932+ | lincRNA-DYM-2 | lincRNA | 5231 |
| 0.5129656 | chr4:158983666-159005030- | lincRNA-GRIA2-1 | lincRNA | 21365 |
| 0.5131277 | ENST00000392131 | AC079305.6 | Ensembl | 550 |
| 0.5148485 | uc004exm.2 | BC007360 | UCSC_knowngene | 646 |
| 0.5252591 | uc004bxt.1 | AX747401 | UCSC_knowngene | 1772 |
| 0.5263956 | ENST00000435702 | AP001046.6 | Ensembl | 2816 |
| 0.5292595 | BE048618 | lincRNA-PTHLH-2 | lincRNA | 518 |
| 0.5337012 | uc001qgd.2 | AX747213 | UCSC_knowngene | 685 |
| 0.5349027 | BC031319 |  | misc_RNA | 701 |
| 0.5367563 | chr4:3829252-3852702+ | lincRNA-OTOP1 | lincRNA | 23451 |
| 0.5460004 | ENST00000450500 | RP11-2L8.1 | Ensembl | 560 |
| 0.5464165 | BF058204 | lincRNA-NANOS3-2 | lincRNA | 422 |
| 0.5479172 | AA179681 | lincRNA-KCNA10 | lincRNA | 502 |
| 0.5505676 | AK310298 | lincRNA-ZNF385D | lincRNA | 1050 |
| 0.5568775 | chr5:170992545-171005495+ | lincRNA-FBXW11-1 | lincRNA | 12951 |
| 0.5571375 | AW954309 | lincRNA-JUN-3 | lincRNA | 668 |
| 0.5650039 | chrX:38863681-38944131- | lincRNA-MID1IP1-2 | lincRNA | 80451 |
| 0.5653912 | NR_026906 | C17orf69 | RefSeq_NR | 942 |
| 0.566264 | CB053878 | lincRNA-CHD7-2 | lincRNA | 473 |
| 0.5685043 | chr5:116378824-116397091- | lincRNA-SEMA6A-6 | lincRNA | 18268 |
| 0.5690456 | BM750448 | lincRNA-FAM70A | lincRNA | 517 |
| 0.5711316 | ENST00000443523 | RP11-556E13.1 | Ensembl | 369 |
| 0.5749772 | AI798958 | lincRNA-TOX | lincRNA | 471 |
| 0.5767763 | AA234012 | lincRNA-AIFM1 | lincRNA | 486 |
| 0.5785042 | CD000492 | lincRNA-WISP3-2 | lincRNA | 304 |
| 0.5787335 | ENST00000418620 | AC106900.6 | Ensembl | 456 |
| 0.5795684 | chr4:178294426-178298806+ | lincRNA-AGA-2 | lincRNA | 4381 |
| 0.5802452 | AL133087 | lincRNA-PGAP1 | lincRNA | 2825 |
| 0.5806026 | chr9:21753446-21790681- | lincRNA-IFNE | lincRNA | 37236 |
| 0.5831476 | AI347010 | lincRNA-SETMAR-4 | lincRNA | 471 |
| 0.5855768 | BU659034 | lincRNA-SYK | lincRNA | 548 |
| 0.5860294 | ENST00000423943 | RP11-48O20.4 | Ensembl | 1113 |
| 0.5869861 | uc003kjh.3 | LOC645323 | UCSC_knowngene | 3406 |
| 0.5883583 | uc004bxu.2 | BC094873 | UCSC_knowngene | 1213 |
| 0.5985232 | ENST00000514844 | RP11-46C20.1 | Ensembl | 667 |
| 0.605585 | chr15:46971964-46982563+ | lincRNA-SEMA6D-6 | lincRNA | 10600 |
| 0.6070217 | uc002dax.1 | AK126539 | UCSC_knowngene | 3552 |
| 0.6075724 | chr5:171107070-171142645+ | lincRNA-FBXW11-3 | lincRNA | 35576 |
| 0.6082363 | ENST00000485282 | CTB-111H14.1 | Ensembl | 543 |
| 0.6104476 | ENST00000414308 | RP11-342D11.3 | Ensembl | 552 |
| 0.6115288 | chr8:49776604-49779322- | lincRNA-EFCAB1-1 | lincRNA | 2719 |
| 0.6124503 | AK074767 | lincRNA-ALDH1A2 | lincRNA | 2922 |
| 0.6124906 | BX096457 | lincRNA-KLF13-1 | lincRNA | 306 |
| 0.6126546 | chr4:114709776-114720551- | lincRNA-CAMK2D | lincRNA | 10776 |
| 0.6140758 | ENST00000504259 | CTD-2061E19.5 | Ensembl | 737 |
| 0.6144461 | ENST00000437523 | AL592494.4 | Ensembl | 369 |
| 0.6163122 | ENST00000475886 | RP11-129K20.2 | Ensembl | 758 |
| 0.6164064 | DQ896053 | lincRNA-TWIST2-1 | lincRNA | 523 |
| 0.616905 | BM979131 | lincRNA-DZIP1L | lincRNA | 751 |
| 0.6241621 | NR_026656 | LOC400043 | RefSeq_NR | 1411 |
| 0.6255847 | AV723845 | lincRNA-KIAA1586 | lincRNA | 673 |
| 0.6263264 | ENST00000440570 | AC004383.4 | Ensembl | 760 |
| 0.6273985 | ENST00000455449 | AC073115.5 | Ensembl | 532 |
| 0.6289408 | AA905299 | lincRNA-EFHA2-2 | lincRNA | 462 |
| 0.631329 | ENST00000444114 | RP5-1172A22.1 | Ensembl | 637 |
| 0.6365292 | chrX:11801246-11812911+ | lincRNA-FRMPD4 | lincRNA | 11666 |
| 0.6386082 | BC105019 |  | NRED | 339 |
| 0.6398144 | ENST00000421191 | XXbac-BPG248L24.10 | Ensembl | 306 |
| 0.6426668 | ENST00000396909 | RP1-190J20.2 | Ensembl | 869 |
| 0.6429242 | ENST00000435800 | RP11-31F15.1 | Ensembl | 1747 |
| 0.6431902 | NR_001564 | XIST | RefSeq_NR | 19271 |
| 0.6438325 | uc004aul.1 | DM004128 | UCSC_knowngene | 103 |
| 0.6480176 | chr4:6753049-6763474+ | lincRNA-KIAA0232 | lincRNA | 10426 |
| 0.6531335 | NR_027349 | MIR17HG | RefSeq_NR | 927 |
| 0.653706 | ENST00000418778 | RP11-488P11.1 | Ensembl | 1528 |
| 0.6555799 | BG206259 | lincRNA-JRKL-1 | lincRNA | 774 |
| 0.6577763 | ENST00000482382 | RP11-246A10.1 | Ensembl | 554 |
| 0.6589376 | AK300950 | lincRNA-FAM65B-1 | lincRNA | 2415 |
| 0.6597161 | EU831898 | lincRNA-RPL29 | lincRNA | 3142 |
| 0.6612559 | chr8:127380668-127399918+ | lincRNA-FAM84B-4 | lincRNA | 19251 |
| 0.6661622 | chr1:188356202-188401002- | lincRNA-PLA2G4A-3 | lincRNA | 44801 |
| 0.6663488 | chr5:126903301-126920351- | lincRNA-PRRC1 | lincRNA | 17051 |
| 0.6663623 | ENST00000391359 | RP1-122K4.3 | Ensembl | 544 |
| 0.6677398 | ENST00000504609 | RP11-461C13.1 | Ensembl | 704 |
| 0.6687722 | AW082319 | lincRNA-CBLN2-2 | lincRNA | 470 |
| 0.6690056 | chr13:86214299-86278949+ | lincRNA-SLITRK6-5 | lincRNA | 64651 |
| 0.6713978 | AK097298 |  | misc_RNA | 2131 |
| 0.6720998 | CD365428 | lincRNA-NFKBIA-2 | lincRNA | 622 |
| 0.6723606 | DC428746 | lincRNA-ARHGAP15 | lincRNA | 577 |
| 0.673518 | ENST00000416534 | AC007463.2 | Ensembl | 734 |
| 0.6748588 | chrX:39547291-39551460- | lincRNA-MID1IP1-8 | lincRNA | 4170 |
| 0.6749281 | BC009884 |  | misc_RNA | 1309 |
| 0.6761632 | chr13:71907099-71940949- | lincRNA-KLHL1-2 | lincRNA | 33851 |
| 0.6773057 | chr5:67675269-67719019+ | lincRNA-SLC30A5-1 | lincRNA | 43751 |
| 0.6775197 | BF448634 | lincRNA-DACT2-1 | lincRNA | 493 |
| 0.6783416 | DQ269985 | lincRNA-AKR1C3-2 | lincRNA | 972 |
| 0.679161 | AA626752 | lincRNA-STIM2-3 | lincRNA | 287 |
| 0.6805584 | DW409420 | lincRNA-CYTH4 | lincRNA | 339 |
| 0.6814627 | BC034615 |  | misc_RNA | 2024 |
| 0.6815907 | ENST00000449812 | RP11-145M4.3 | Ensembl | 623 |
| 0.6841386 | BQ000389 | lincRNA-C8orf38-1 | lincRNA | 727 |
| 0.6843245 | ENST00000507887 | CTD-2306M5.1 | Ensembl | 638 |
| 0.6862477 | chr4:147979327-148023300- | lincRNA-TTC29-1 | lincRNA | 43974 |
| 0.6868131 | uc004dgl.1 | BC015977 | UCSC_knowngene | 271 |
| 0.687835 | NR_027131 | CXorf42 | RefSeq_NR | 2434 |
| 0.689829 | uc002yug.2 | AK025786 | UCSC_knowngene | 2525 |
| 0.6898549 | ENST00000450314 | EMX2OS | Ensembl | 520 |
| 0.6905413 | ENST00000424116 | AC098617.2 | Ensembl | 486 |
| 0.6918496 | AW177676 | lincRNA-CAPN13 | lincRNA | 119 |
| 0.6929061 | ENST00000503091 | AC008592.5 | Ensembl | 665 |
| 0.6930853 | AA863287 | lincRNA-OR2AG2 | lincRNA | 519 |
| 0.6941493 | AI553950 | lincRNA-INSL6 | lincRNA | 449 |
| 0.6969815 | ENST00000456100 | AL163953.3 | Ensembl | 562 |
| 0.6990638 | AA224517 | lincRNA-TLR5 | lincRNA | 553 |
| 0.6998131 | BF958740 | lincRNA-CRYBA4-2 | lincRNA | 226 |
| 0.6999523 | ENST00000503142 | RP11-46A10.6 | Ensembl | 537 |
| 0.7000882 | ENST00000502335 | AC105201.1 | Ensembl | 1639 |
| 0.7006293 | BX647686 | lincRNA-CCR6 | lincRNA | 5327 |
| 0.7010975 | AK123068 | lincRNA-KALRN | lincRNA | 2096 |
| 0.7019955 | chr6:132581948-132592727+ | lincRNA-MOXD1-3 | lincRNA | 10780 |
| 0.7033531 | ENST00000457653 | RP1-238O23.5 | Ensembl | 3132 |
| 0.7044842 | EH335967 | lincRNA-LMO4-1 | lincRNA | 66 |
| 0.704651 | AK097857 |  | misc_RNA | 1950 |
| 0.7064406 | AA613157 | lincRNA-FAM135B | lincRNA | 342 |
| 0.7074578 | ENST00000380334 | CA5BP | Ensembl | 889 |
| 0.7077448 | BG200952 | lincRNA-KCNU1-2 | lincRNA | 653 |
| 0.7080622 | ENST00000437648 | RP11-146I2.1 | Ensembl | 1017 |
| 0.7080633 | chr6:63073666-63086291+ | lincRNA-LGSN | lincRNA | 12626 |
| 0.7086575 | uc004cuh.2 | AY660577 | UCSC_knowngene | 823 |
| 0.7104646 | uc001unz.1 | AK093279 | UCSC_knowngene | 2100 |
| 0.7105189 | AI902205 | lincRNA-OXGR1-2 | lincRNA | 448 |
| 0.7105329 | BC033539 |  | misc_RNA | 2851 |
| 0.7105489 | ENST00000317122 | RP11-169K16.9 | Ensembl | 2468 |
| 0.710797 | chr2:197823955-197835530+ | lincRNA-ANKRD44 | lincRNA | 11576 |
| 0.7114096 | AI017477 | lincRNA-TSN-2 | lincRNA | 464 |
| 0.71222 | AI806168 | lincRNA-CPM | lincRNA | 472 |
| 0.7123608 | AA101261 | lincRNA-BCOR-3 | lincRNA | 381 |
| 0.7135614 | chr14:99226072-99237672- | lincRNA-C14orf177-1 | lincRNA | 11601 |
| 0.7141908 | EC495588 | lincRNA-RCN2 | lincRNA | 90 |
| 0.7158603 | chr11:57057697-57066754- | lincRNA-APLNR | lincRNA | 9058 |
| 0.7159882 | AI214699 | lincRNA-HES1-5 | lincRNA | 382 |
| 0.7162078 | uc002nqe.2 | CR590976 | UCSC_knowngene | 1582 |
| 0.716209 | chr9:110999314-111011179- | lincRNA-KLF4-5 | lincRNA | 11866 |
| 0.7165891 | chr7:73684091-73687309+ | lincRNA-CLIP2 | lincRNA | 3219 |
| 0.7172021 | ENST00000423122 | RP11-65J3.1 | Ensembl | 545 |
| 0.7173501 | AK094780 | lincRNA-GALNTL2-1 | lincRNA | 3105 |
| 0.7176734 | DB341353 | lincRNA-CXCR7-2 | lincRNA | 536 |
| 0.7180589 | DB325375 | lincRNA-SIX6 | lincRNA | 551 |
| 0.7182605 | AK090977 |  | misc_RNA | 2052 |
| 0.7188165 | ENST00000452612 | RP3-477H23.3 | Ensembl | 746 |
| 0.7190473 | BU681290 | lincRNA-EFCAB1-3 | lincRNA | 716 |
| 0.720102 | AK293821 | lincRNA-SYNCRIP-2 | lincRNA | 1499 |
| 0.7219181 | AK026788 |  | misc_RNA | 2216 |
| 0.7247295 | chr2:129541655-129552355- | lincRNA-HS6ST1-6 | lincRNA | 10701 |
| 0.724923 | ENST00000434530 | AC018816.4 | Ensembl | 2216 |
| 0.725939 | chrX:118619272-118629135+ | lincRNA-CXorf56-1 | lincRNA | 9864 |
| 0.7264786 | uc002uns.1 | AK125001 | UCSC_knowngene | 1765 |
| 0.7276183 | ENST00000417795 | AC034220.3 | Ensembl | 1792 |
| 0.7278533 | chr8:99342599-99355474+ | lincRNA-KCNS2-1 | lincRNA | 12876 |
| 0.7281647 | NR_027697 | FLJ39653 | RefSeq_NR | 2496 |
| 0.7293719 | DA579624 | lincRNA-GSDMC-8 | lincRNA | 568 |
| 0.7295321 | AK091319 |  | misc_RNA | 2297 |
| 0.7297646 | AI902449 | lincRNA-MRPS9-3 | lincRNA | 174 |
| 0.7303636 | ENST00000435967 | AC007128.1 | Ensembl | 557 |
| 0.7323109 | uc001fuu.2 | AF088076 | UCSC_knowngene | 423 |
| 0.7339061 | AK297077 | lincRNA-FYB | lincRNA | 2747 |
| 0.7339096 | exon678+ | lincRNA-KCNA4-3 | lincRNA | 279 |
| 0.7343422 | AA420706 | lincRNA-P2RY1-2 | lincRNA | 340 |
| 0.7344987 | chr15:38666901-38708821- | lincRNA-SPRED1-1 | lincRNA | 41921 |
| 0.7349691 | ENST00000442197 | AL132709.9 | Ensembl | 625 |
| 0.7356493 | AF422192 |  | misc_RNA | 1422 |
| 0.7356885 | uc003wpf.1 | AK128400 | UCSC_knowngene | 3993 |
| 0.7357259 | BP360743 | lincRNA-GPR98 | lincRNA | 582 |
| 0.736161 | ENST00000513381 | RP11-834C11.5 | Ensembl | 499 |
| 0.7361798 | BU570390 | lincRNA-GDF6-2 | lincRNA | 413 |
| 0.7362079 | NR_027007 | LOC440944 | RefSeq_NR | 1907 |
| 0.7366356 | AA164337 | lincRNA-AJAP1-2 | lincRNA | 585 |
| 0.7370987 | chr13:43764375-43778425- | lincRNA-DNAJC15 | lincRNA | 14051 |
| 0.7386001 | ENST00000480386 | RP11-357H14.4 | Ensembl | 566 |
| 0.7390703 | BJ997865 | lincRNA-SUCLG2-1 | lincRNA | 540 |
| 0.7395312 | chr18:76387637-76400887- | lincRNA-GALR1-1 | lincRNA | 13251 |
| 0.7400464 | BQ720489 | lincRNA-CDH19 | lincRNA | 965 |
| 0.7404608 | uc002yli.1 | AK124194 | UCSC_knowngene | 2908 |
| 0.7409368 | NR_024506 | LOC646982 | RefSeq_NR | 3834 |
| 0.7420391 | ENST00000509139 | CTC-529P8.1 | Ensembl | 654 |
| 0.7425127 | exon2749- | lincRNA-PLXNA1-4 | lincRNA | 223 |
| 0.7425212 | AK309032 | lincRNA-LMO4-3 | lincRNA | 1152 |
| 0.7428177 | ENST00000425205 | RP11-40H20.4 | Ensembl | 379 |
| 0.7428641 | ENST00000429730 | AC079767.4 | Ensembl | 492 |
| 0.7430186 | BE065042 | lincRNA-TPBG-2 | lincRNA | 676 |
| 0.7433481 | BM043959 | lincRNA-ADAMTSL4-2 | lincRNA | 754 |
| 0.7435691 | uc001dqu.2 | BC030750 | UCSC_knowngene | 1327 |
| 0.7441491 | DA800392 | lincRNA-CDON-1 | lincRNA | 445 |
| 0.7449089 | ENST00000446281 | AC087859.1 | Ensembl | 932 |
| 0.7456863 | ENST00000507989 | RP11-661C8.2 | Ensembl | 559 |
| 0.7457117 | uc002vwf.1 | AK056246 | UCSC_knowngene | 2341 |
| 0.7459759 | uc003adh.2 | BC015159 | UCSC_knowngene | 687 |
| 0.7464713 | chr12:106625035-106628293- | lincRNA-NUAK1 | lincRNA | 3259 |
| 0.7468157 | chr7:152596217-152621642+ | lincRNA-DPP6-2 | lincRNA | 25426 |
| 0.7478124 | ENST00000512717 | RP11-700H6.2 | Ensembl | 789 |
| 0.7478838 | ENST00000441380 | IFITM4P | Ensembl | 420 |
| 0.7481505 | ENST00000419595 | RP11-458F8.1 | Ensembl | 298 |
| 0.7487275 | AA999834 | lincRNA-MRPS28 | lincRNA | 536 |
| 0.7489285 | BF850330 | lincRNA-MESDC2 | lincRNA | 177 |
| 0.7490899 | NR_027441 | FLJ36777 | RefSeq_NR | 2140 |
| 0.7506899 | chr1:228917027-228938752- | lincRNA-RHOU-6 | lincRNA | 21726 |
| 0.751091 | exon1963- | lincRNA-CTAGE1-2 | lincRNA | 329 |
| 0.7511659 | DA243478 | lincRNA-CRH-1 | lincRNA | 596 |
| 0.751246 | CD357052 | lincRNA-MED30-2 | lincRNA | 743 |
| 0.7513928 | ENST00000436045 | FMO7P | Ensembl | 768 |
| 0.7514413 | ENST00000511169 | AC006499.7 | Ensembl | 607 |
| 0.7525468 | EG328292 | lincRNA-TMEM183A | lincRNA | 169 |
| 0.7526055 | BC050642 | lincRNA-ENO1-1 | lincRNA | 1898 |
| 0.7528157 | BC036122 | lincRNA-LRRC8D-2 | lincRNA | 3001 |
| 0.7536613 | ENST00000445814 | XIST | Ensembl | 437 |
| 0.7543249 | AA583819 | lincRNA-LGI2 | lincRNA | 257 |
| 0.7553508 | uc003tsv.2 | BC042811 | UCSC_knowngene | 635 |
| 0.7558076 | DA271257 | lincRNA-CALB1-1 | lincRNA | 509 |
| 0.7569527 | uc009yjc.1 | EF565102 | UCSC_knowngene | 403 |
| 0.7573697 | DA521516 | lincRNA-MEX3B-1 | lincRNA | 565 |
| 0.7576287 | AK309519 | lincRNA-INMT | lincRNA | 1057 |
| 0.757714 | BF675557 | lincRNA-SUSD1 | lincRNA | 772 |
| 0.7579923 | BC043564 | lincRNA-KCNA2-1 | lincRNA | 2022 |
| 0.7585635 | chr9:16334709-16341952- | lincRNA-C9orf93-1 | lincRNA | 7244 |
| 0.7588479 | chr2:224986097-224992215+ | lincRNA-FAM124B-2 | lincRNA | 6119 |
| 0.759122 | DB239440 | lincRNA-SLC7A13 | lincRNA | 578 |
| 0.7601197 | AV730166 | lincRNA-TBC1D19 | lincRNA | 684 |
| 0.760176 | chr1:200231279-200242758+ | lincRNA-ZNF281-1 | lincRNA | 11480 |
| 0.7612987 | ENST00000425911 | AC092638.2 | Ensembl | 885 |
| 0.7613912 | chr8:8142808-8153502+ | lincRNA-SGK223-1 | lincRNA | 10695 |
| 0.7615038 | uc001gov.2 | AF387615 | UCSC_knowngene | 137 |
| 0.7621087 | AU311832 | lincRNA-SHQ1-1 | lincRNA | 571 |
| 0.7626145 | BU149307 | lincRNA-C1orf55 | lincRNA | 973 |
| 0.7627951 | ENST00000446360 | RP4-620F22.3 | Ensembl | 662 |
| 0.7629206 | CN289466 | lincRNA-MEIS1-1 | lincRNA | 784 |
| 0.763151 | chr5:64421409-64427917+ | lincRNA-ADAMTS6 | lincRNA | 6509 |
| 0.7634821 | chr2:107718693-107737818+ | lincRNA-RGPD4-2 | lincRNA | 19126 |
| 0.7638597 | chr10:44829994-44851569+ | lincRNA-CXCL12-3 | lincRNA | 21576 |
| 0.7642065 | AK022073 |  | misc_RNA | 1873 |
| 0.7649205 | AK055754 |  | misc_RNA | 1839 |
| 0.7649845 | BG210085 | lincRNA-PBX1-1 | lincRNA | 282 |
| 0.7652964 | chr3:168755756-168781330+ | lincRNA-MECOM-3 | lincRNA | 25575 |
| 0.7658541 | uc003nkk.1 | AK127889 | UCSC_knowngene | 2163 |
| 0.7663662 | uc003wyp.1 | CR613267 | UCSC_knowngene | 2275 |
| 0.7664812 | chr5:81089894-81100394- | lincRNA-SSBP2 | lincRNA | 10501 |
| 0.7668852 | NR_003530 | MEG3 | RefSeq_NR | 1855 |
| 0.7669177 | ENST00000499953 | AC026250.1 | Ensembl | 927 |
| 0.7674272 | N36328 | lincRNA-TTC18 | lincRNA | 362 |
| 0.7686014 | chr6:47389241-47404066- | lincRNA-TNFRSF21-2 | lincRNA | 14826 |
| 0.769488 | BE179605 | lincRNA-TRPC4-3 | lincRNA | 541 |
| 0.7694969 | uc001iuq.1 | LOC387647 | UCSC_knowngene | 845 |
| 0.7699326 | ENST00000427276 | RP11-359N11.2 | Ensembl | 3152 |
| 0.770311 | ENST00000512628 | RP11-545H22.1 | Ensembl | 624 |
| 0.7703199 | AI904030 | lincRNA-KIAA1109-2 | lincRNA | 390 |
| 0.7704618 | ENST00000429250 | RP11-90J7.3 | Ensembl | 607 |
| 0.7705957 | ENST00000437705 | RP11-248N6.2 | Ensembl | 862 |
| 0.7708094 | G36719 |  | misc_RNA | 454 |
| 0.7712062 | chr2:134460980-134475605- | lincRNA-NCKAP5-2 | lincRNA | 14626 |
| 0.7712551 | ENST00000449605 | RP11-147O5.1 | Ensembl | 7619 |
| 0.7713895 | uc002pxl.3 | UNQ2487 | UCSC_knowngene | 1893 |
| 0.7715584 | AW872580 | lincRNA-MYO1B-3 | lincRNA | 381 |
| 0.771855 | ENST00000506121 | C8orf75 | Ensembl | 1301 |
| 0.7721129 | DA312854 | lincRNA-KCNT2-6 | lincRNA | 548 |
| 0.7721303 | ENST00000423803 | RP11-292F22.2 | Ensembl | 1604 |
| 0.7721889 | chr1:54928212-54960380- | lincRNA-SSBP3 | lincRNA | 32169 |
| 0.7722321 | ENST00000451749 | AC073409.1 | Ensembl | 269 |
| 0.7724571 | BF352825 | lincRNA-DYRK2-2 | lincRNA | 324 |
| 0.7726445 | chr8:21138470-21155170- | lincRNA-LZTS1-8 | lincRNA | 16701 |
| 0.773278 | AW452601 | lincRNA-MTX3 | lincRNA | 524 |
| 0.7732988 | AB002362 | lincRNA-IGSF1 | lincRNA | 5413 |
| 0.7735224 | uc001hpe.1 | AK124056 | UCSC_knowngene | 1530 |
| 0.7752174 | BC033956 |  | misc_RNA | 886 |
| 0.7753055 | AK097171 |  | misc_RNA | 2511 |
| 0.7757694 | chr1:231020477-231031277- | lincRNA-C1orf198 | lincRNA | 10801 |
| 0.7758876 | ENST00000401460 | RP1-72A23.3 | Ensembl | 267 |
| 0.7766762 | exon91+ | lincRNA-KNCN | lincRNA | 175 |
| 0.7767954 | BX105648 | lincRNA-ZNF182 | lincRNA | 503 |
| 0.7779518 | ENST00000435485 | RP11-308N19.1 | Ensembl | 1686 |
| 0.7781553 | chrX:40874482-40878593- | lincRNA-MED14-4 | lincRNA | 4112 |
| 0.7782252 | chrX:30806304-30826229+ | lincRNA-TAB3 | lincRNA | 19926 |
| 0.77842 | BE967171 | lincRNA-LPP-3 | lincRNA | 945 |
| 0.7786268 | CF891057 | lincRNA-DNAJB12 | lincRNA | 691 |
| 0.7788829 | ENST00000502521 | RP11-933H2.4 | Ensembl | 1978 |
| 0.7789097 | DR007897 | lincRNA-POU2AF1 | lincRNA | 521 |
| 0.7799724 | NR_026867 | LOC134466 | RefSeq_NR | 2420 |
| 0.7800696 | BE617381 | lincRNA-GDF6-1 | lincRNA | 1097 |
| 0.7810924 | BC028700 |  | misc_RNA | 3838 |
| 0.7813718 | AW299747 | lincRNA-SUMF1-1 | lincRNA | 464 |
| 0.7814568 | ENST00000453828 | RP11-520A21.1 | Ensembl | 670 |
| 0.7822266 | ENST00000455966 | RP1-224A6.3 | Ensembl | 615 |
| 0.7831949 | ENST00000425071 | AC010082.1 | Ensembl | 471 |
| 0.7836025 | BG260387 | lincRNA-KRR1 | lincRNA | 749 |
| 0.7846477 | AA219324 | lincRNA-PDE4D-1 | lincRNA | 396 |
| 0.7854336 | chr9:81923716-81932183- | lincRNA-PSAT1-3 | lincRNA | 8468 |
| 0.7855111 | ENST00000450208 | RP11-193H5.2 | Ensembl | 775 |
| 0.7858892 | AY927465 |  | RNAdb | 209 |
| 0.7861216 | AA780570 | lincRNA-JARID2-3 | lincRNA | 284 |
| 0.786156 | uc002uvg.2 | BC039445 | UCSC_knowngene | 944 |
| 0.7864065 | CD511325 | lincRNA-NAT8B | lincRNA | 784 |
| 0.7872679 | CA414224 | lincRNA-CHST11 | lincRNA | 524 |
| 0.7873049 | ENST00000512406 | RP11-480D4.2 | Ensembl | 440 |
| 0.7873941 | BC016673 |  | misc_RNA | 1443 |
| 0.7878549 | ENST00000439279 | RP11-436D23.1 | Ensembl | 226 |
| 0.7883644 | ENST00000417112 | RP11-554I8.2 | Ensembl | 917 |
| 0.7889692 | AW075897 | lincRNA-SIN3A | lincRNA | 521 |
| 0.7894813 | ENST00000515077 | CTD-2187J20.1 | Ensembl | 509 |
| 0.7898169 | DB317657 | lincRNA-RPS6KC1-2 | lincRNA | 543 |
| 0.7899122 | BX090019 | lincRNA-SLC25A37 | lincRNA | 743 |
| 0.7904785 | ENST00000511272 | RP11-324H7.1 | Ensembl | 1646 |
| 0.7904886 | chrX:38716431-38792681- | lincRNA-MID1IP1-9 | lincRNA | 76251 |
| 0.7904977 | chr13:20881350-20901600+ | lincRNA-CRYL1 | lincRNA | 20251 |
| 0.7905199 | AA528614 | lincRNA-MYC-10 | lincRNA | 307 |
| 0.7907674 | HIT000395572 | | H-invDB | 354 |
| 0.7913085 | BX483760 | lincRNA-SEMA6A-4 | lincRNA | 683 |
| 0.7920622 | ENST00000418025 | AC097713.3 | Ensembl | 562 |
| 0.7932296 | chr1:54928212-54960380+ | lincRNA-ACOT11 | lincRNA | 32169 |
| 0.7938901 | BX491518 | lincRNA-MFSD5 | lincRNA | 466 |
| 0.7940248 | chr3:126825067-126831976+ | lincRNA-TPRA1-3 | lincRNA | 6910 |
| 0.7947804 | chr2:107718693-107737818- | lincRNA-ST6GAL2-3 | lincRNA | 19126 |
| 0.7953416 | ENST00000394662 | RP4-644F6.3 | Ensembl | 1740 |
| 0.7953905 | uc003ylf.2 | AK125733 | UCSC_knowngene | 1773 |
| 0.7961508 | uc002bnt.2 | LOC254559 | UCSC_knowngene | 4778 |
| 0.7968863 | BC040186 |  | RNAdb | 4580 |
| 0.7975836 | chr1:193785802-193801027+ | lincRNA-KCNT2-3 | lincRNA | 15226 |
| 0.797964 | AK098581 | lincRNA-NFATC1-1 | lincRNA | 1110 |
| 0.7979686 | chr12:114662611-114678879+ | lincRNA-TBX5-3 | lincRNA | 16269 |
| 0.7980315 | ENST00000432701 | RP11-269C23.3 | Ensembl | 858 |
| 0.7988163 | ENST00000512947 | CTC-236F12.1 | Ensembl | 712 |
| 0.7990567 | AI910857 | lincRNA-GLIPR1L2 | lincRNA | 485 |
| 0.7991655 | ENST00000431943 | RP11-191G24.2 | Ensembl | 946 |
| 0.7993037 | D38733 | lincRNA-EFR3A | lincRNA | 170 |
| 0.8003199 | chr13:22305525-22317225+ | lincRNA-SGCG-4 | lincRNA | 11701 |
| 0.8005688 | ENST00000507813 | CTC-339F2.2 | Ensembl | 206 |
| 0.8007506 | BQ638596 | lincRNA-TRHR-2 | lincRNA | 619 |
| 0.8018435 | AW879648 | lincRNA-SLC37A3-2 | lincRNA | 366 |
| 0.8023868 | ENST00000445631 | RP11-91N2.3 | Ensembl | 206 |
| 0.8035868 | ENST00000503465 | RP11-83C7.1 | Ensembl | 500 |
| 0.8036828 | BF531040 | lincRNA-AGPAT5-2 | lincRNA | 862 |
| 0.803882 | ENST00000477702 | NCRNA00173 | Ensembl | 314 |
| 0.8039406 | CF135687 | lincRNA-LHFPL2 | lincRNA | 600 |
| 0.804601 | AI376773 | lincRNA-P2RY2 | lincRNA | 495 |
| 0.8046093 | ENST00000447571 | AC079807.4 | Ensembl | 1808 |
| 0.8052108 | BG946037 | lincRNA-MYEOV-3 | lincRNA | 657 |
| 0.8056947 | BU657491 | lincRNA-CACNA1C | lincRNA | 581 |
| 0.8059505 | ENST00000405813 | AP003356.1 | Ensembl | 534 |
| 0.8060315 | chr12:114569667-114608017- | lincRNA-RBM19-1 | lincRNA | 38351 |
| 0.806098 | ENST00000412381 | AC142119.1 | Ensembl | 642 |
| 0.8062925 | chr2:102880643-102894318+ | lincRNA-IL1RL1 | lincRNA | 13676 |
| 0.8064238 | ENST00000415349 | AC125238.4 | Ensembl | 1299 |
| 0.8065333 | AA586761 | lincRNA-GFRA2-3 | lincRNA | 306 |
| 0.8066794 | DA150164 | lincRNA-LINGO1-3 | lincRNA | 573 |
| 0.8069563 | BF509048 | lincRNA-FAM9C-3 | lincRNA | 740 |
| 0.8073948 | uc002the.2 | LOC541471 | UCSC_knowngene | 1583 |
| 0.8076791 | AA493815 | lincRNA-JUN-2 | lincRNA | 355 |
| 0.8079818 | BG254544 | lincRNA-SPDEF | lincRNA | 1043 |
| 0.8081242 | DA330754 | lincRNA-TMSB10 | lincRNA | 558 |
| 0.8086502 | CR741480 | lincRNA-FBN1 | lincRNA | 552 |
| 0.8086756 | BF108976 | lincRNA-MYO1B-4 | lincRNA | 509 |
| 0.8088473 | uc003wst.1 | LOC157627 | UCSC_knowngene | 869 |
| 0.8089403 | BG925420 | lincRNA-PIK3R1-1 | lincRNA | 694 |
| 0.8095026 | AY927588 |  | misc_RNA | 1178 |
| 0.8097085 | chrX:68350530-68358250+ | lincRNA-PJA1-4 | lincRNA | 7721 |
| 0.8103847 | N48415 | lincRNA-ATP11C-1 | lincRNA | 541 |
| 0.8104138 | BX501243 | lincRNA-HABP2-1 | lincRNA | 591 |
| 0.8107271 | chr2:122573205-122584730+ | lincRNA-CNTNAP5-2 | lincRNA | 11526 |
| 0.810901 | ENST00000417149 | RP11-298E9.6 | Ensembl | 390 |
| 0.8115053 | BX641108 | lincRNA-TPRG1-2 | lincRNA | 3994 |
| 0.811835 | chr2:223964483-223968702- | lincRNA-KCNE4-1 | lincRNA | 4220 |
| 0.8120574 | AI004797 | lincRNA-HAS2-1 | lincRNA | 460 |
| 0.8121204 | ENST00000454588 | RP1-45I4.3 | Ensembl | 552 |
| 0.8121617 | chr6:24977604-24998368+ | lincRNA-LRRC16A-2 | lincRNA | 20765 |
| 0.8123126 | NR_001590 | IFITM4P | RefSeq_NR | 342 |
| 0.8123973 | ENST00000412606 | AC096559.1 | Ensembl | 578 |
| 0.8133503 | BM565532 | lincRNA-CACNA2D1 | lincRNA | 599 |
| 0.8135306 | chr12:55389508-55403008+ | lincRNA-NEUROD4 | lincRNA | 13501 |
| 0.8136737 | AW661714 | lincRNA-TP53INP1-2 | lincRNA | 451 |
| 0.8138119 | NR_024396 | MGC23270 | RefSeq_NR | 2518 |
| 0.8140107 | BX503241 | lincRNA-FZD6-1 | lincRNA | 473 |
| 0.8144724 | BC043232 | lincRNA-ZSCAN5A | lincRNA | 2472 |
| 0.8147971 | BG898852 | lincRNA-RAB3IP | lincRNA | 605 |
| 0.8148077 | ENST00000481148 | RP11-398A8.3 | Ensembl | 492 |
| 0.8154547 | chr2:142935555-142951705- | lincRNA-LRP1B-2 | lincRNA | 16151 |
| 0.8155919 | DA096519 | lincRNA-TLE4-2 | lincRNA | 553 |
| 0.8156182 | ENST00000451364 | RP11-429G19.2 | Ensembl | 716 |
| 0.815665 | ENST00000457217 | RP11-222A5.1 | Ensembl | 1809 |
| 0.8166878 | CB528940 | lincRNA-SDC1 | lincRNA | 663 |
| 0.8167132 | ENST00000435695 | AC002454.1 | Ensembl | 432 |
| 0.816818 | AA593324 | lincRNA-FGFR3 | lincRNA | 413 |
| 0.8174266 | H00916 | lincRNA-NTF3-1 | lincRNA | 500 |
| 0.8176308 | chr3:67128385-67147960- | lincRNA-KBTBD8-2 | lincRNA | 19576 |
| 0.8178791 | ENST00000415726 | RP11-336K24.5 | Ensembl | 401 |
| 0.8179174 | AK126393 |  | misc_RNA | 2855 |
| 0.8188401 | ENST00000414721 | AP001496.2 | Ensembl | 607 |
| 0.8188463 | AK057683 |  | misc_RNA | 3087 |
| 0.8189001 | chr5:171991045-172004045- | lincRNA-SH3PXD2B | lincRNA | 13001 |
| 0.8191624 | BC035264 |  | misc_RNA | 3424 |
| 0.8193197 | chr4:15911552-15921927+ | lincRNA-FGFBP1 | lincRNA | 10376 |
| 0.819403 | chrX:20419404-20431504+ | lincRNA-CNKSR2-2 | lincRNA | 12101 |
| 0.8194147 | ENST00000444229 | RP11-527F13.1 | Ensembl | 583 |
| 0.8194603 | AW444688 | lincRNA-RFT1-1 | lincRNA | 486 |
| 0.8197563 | DB452487 | lincRNA-EN1-2 | lincRNA | 475 |
| 0.8199216 | BU188646 | lincRNA-FCRL5-2 | lincRNA | 790 |
| 0.8201873 | AK124257 |  | misc_RNA | 2362 |
| 0.8202935 | chr8:86474623-86517048- | lincRNA-CA2 | lincRNA | 42426 |
| 0.8203277 | ENST00000514574 | CTC-327F10.1 | Ensembl | 1016 |
| 0.8204931 | BM923628 | lincRNA-BCOR-5 | lincRNA | 1670 |
| 0.8205303 | exon850- | lincRNA-TMPRSS5 | lincRNA | 308 |
| 0.8207075 | AK296262 | lincRNA-PTEN-1 | lincRNA | 1121 |
| 0.8207741 | CA395304 | lincRNA-CCL2 | lincRNA | 601 |
| 0.8208914 | DB451083 | lincRNA-SALL3-2 | lincRNA | 481 |
| 0.8211182 | AI655567 | lincRNA-FKBP1A | lincRNA | 433 |
| 0.8213477 | uc002dzu.2 | BC073928 | UCSC_knowngene | 1121 |
| 0.8216377 | AK126918 | lincRNA-GLIS2 | lincRNA | 4466 |
| 0.821817 | ENST00000434947 | RP3-448I9.1 | Ensembl | 405 |
| 0.8220101 | ENST00000429601 | RP11-368D24__A.1 | Ensembl | 487 |
| 0.8221882 | DB335899 | lincRNA-TMEM177 | lincRNA | 513 |
| 0.8226827 | exon95+ | lincRNA-KNCN | lincRNA | 271 |
| 0.8232301 | N80091 | lincRNA-SPATA8-3 | lincRNA | 436 |
| 0.8233984 | uc010lac.1 | LOC493754 | UCSC_knowngene | 4265 |
| 0.823669 | uc001czu.2 | BC030753 | UCSC_knowngene | 1269 |
| 0.823909 | AA558434 | lincRNA-DCAF12L1 | lincRNA | 556 |
| 0.8244802 | NR_028344 | RP1-177G6.2 | RefSeq_NR | 1398 |
| 0.8246708 | AI038085 | lincRNA-ZHX2-1 | lincRNA | 505 |
| 0.8247051 | ENST00000441001 | RP11-491H19.1 | Ensembl | 477 |
| 0.8251421 | AA176626 | lincRNA-KCNA2-2 | lincRNA | 510 |
| 0.8257318 | BM666256 | lincRNA-C3orf39 | lincRNA | 589 |
| 0.825755 | AI763350 | lincRNA-SREBF2 | lincRNA | 486 |
| 0.8257738 | CD557263 | lincRNA-GABRP-1 | lincRNA | 870 |
| 0.8258238 | ENST00000429843 | AP000240.7 | Ensembl | 406 |
| 0.8258243 | AK021484 | lincRNA-SYNPO2 | lincRNA | 3422 |
| 0.8261153 | chr1:2952104-2969716- | lincRNA-ACTRT2 | lincRNA | 17613 |
| 0.8264262 | BQ187752 | lincRNA-ATP13A3 | lincRNA | 642 |
| 0.826479 | DB302574 | lincRNA-INSM2 | lincRNA | 570 |
| 0.8268997 | ENST00000502790 | RP11-692D12.1 | Ensembl | 685 |
| 0.8270552 | uc003ftp.3 | BC038368 | UCSC_knowngene | 2666 |
| 0.8270674 | AA935188 | lincRNA-MCM9 | lincRNA | 178 |
| 0.8272189 | ENST00000457273 | RP11-475O6.1 | Ensembl | 1991 |
| 0.8277654 | ENST00000424837 | AP000282.3 | Ensembl | 576 |
| 0.8277971 | AY927492 |  | RNAdb | 503 |
| 0.8278116 | chr2:234783886-234795411+ | lincRNA-TRPM8 | lincRNA | 11526 |
| 0.8278625 | AA431609 | lincRNA-ZNF706-1 | lincRNA | 499 |
| 0.8280461 | CR738423 | lincRNA-CTTNBP2NL-1 | lincRNA | 700 |
| 0.8281584 | chr11:44557524-44573924- | lincRNA-ALX4 | lincRNA | 16401 |
| 0.8285661 | ENST00000454403 | RP11-396A22.4 | Ensembl | 411 |
| 0.8285886 | ENST00000426799 | RP11-314A15.2 | Ensembl | 995 |
| 0.8289993 | AV731505 | lincRNA-PDE7A | lincRNA | 403 |
| 0.829448 | BQ006080 | lincRNA-CWC22-2 | lincRNA | 786 |
| 0.8295078 | BI038043 | lincRNA-AGK | lincRNA | 564 |
| 0.8296748 | AK093610 |  | misc_RNA | 2514 |
| 0.8299523 | NR_023918 | HSPC157 | RefSeq_NR | 1103 |
| 0.8301981 | BC028406 |  | misc_RNA | 3478 |
| 0.8306145 | ENST00000465177 | MT-ND1 | Ensembl | 898 |
| 0.830759 | ENST00000439156 | RP11-776H12.1 | Ensembl | 1796 |
| 0.8309871 | ENST00000447462 | RP11-528A4.3 | Ensembl | 323 |
| 0.8316126 | AK090538 |  | misc_RNA | 3438 |
| 0.8319801 | ENST00000511921 | AC034199.2 | Ensembl | 2182 |
| 0.8322639 | AW850155 | lincRNA-AEBP2-1 | lincRNA | 700 |
| 0.8324326 | chr2:187731830-187759380- | lincRNA-ZSWIM2-2 | lincRNA | 27551 |
| 0.8326208 | AA629022 | lincRNA-GLS-1 | lincRNA | 458 |
| 0.8333686 | BC063314 |  | misc_RNA | 2844 |
| 0.8334485 | uc010bbd.1 | BC052811 | UCSC_knowngene | 966 |
| 0.8334625 | exon397+ | lincRNA-FAM107B | lincRNA | 599 |
| 0.8337669 | T19937 | lincRNA-LRRC6 | lincRNA | 209 |
| 0.8340142 | chr1:214288602-214300602- | lincRNA-PROX1-1 | lincRNA | 12001 |
| 0.8340813 | NR_026769 | PPBPL2 | RefSeq_NR | 1362 |
| 0.8341834 | BF513755 | lincRNA-PRKRIR | lincRNA | 493 |
| 0.8342944 | ENST00000503106 | RP11-434D9.1 | Ensembl | 557 |
| 0.8343374 | exon2439+ | lincRNA-PTPN1-2 | lincRNA | 272 |
| 0.8344808 | AW851687 | lincRNA-JHDM1D-2 | lincRNA | 567 |
| 0.8345276 | uc001iot.1 | BC078172 | UCSC_knowngene | 917 |
| 0.8348941 | chr11:126975121-126979584+ | lincRNA-ETS1-1 | lincRNA | 4464 |
| 0.8350309 | AI201251 | lincRNA-LITAF-2 | lincRNA | 489 |
| 0.8357461 | ENST00000421994 | RP4-633H17.2 | Ensembl | 653 |
| 0.8361995 | ENST00000392635 | RP11-252O18.3 | Ensembl | 486 |
| 0.8363156 | AA731927 | lincRNA-CDCA7-2 | lincRNA | 270 |
| 0.8364226 | ENST00000499560 | Z95152.2 | Ensembl | 1227 |
| 0.8367515 | NR_015381 | TP53TG1 | RefSeq_NR | 751 |
| 0.8369693 | chr8:37531517-37542367+ | lincRNA-ZNF703-1 | lincRNA | 10851 |
| 0.8369957 | chr6:23664596-23678671+ | lincRNA-NRSN1-2 | lincRNA | 14076 |
| 0.8371613 | chr14:93595225-93603463+ | lincRNA-MOAP1 | lincRNA | 8239 |
| 0.8371626 | ENST00000436113 | RP3-449H6.1 | Ensembl | 473 |
| 0.8372877 | uc003qac.2 | BC036196 | UCSC_knowngene | 2629 |
| 0.8374044 | uc003ktd.2 | BC043373 | UCSC_knowngene | 1650 |
| 0.8375981 | ENST00000498198 | RP11-428G5.1 | Ensembl | 289 |
| 0.8377564 | ENST00000418607 | BX004987.7 | Ensembl | 964 |
| 0.8386561 | ENST00000415252 | RP1-3D11.2 | Ensembl | 457 |
| 0.8388888 | NR_024467 | LOC100188947 | RefSeq_NR | 1038 |
| 0.8389943 | NR_024383 | LOC645323 | RefSeq_NR | 3360 |
| 0.83922 | ENST00000466206 | RP11-657O9.1 | Ensembl | 562 |
| 0.8393139 | AA587541 | lincRNA-MEF2A | lincRNA | 441 |
| 0.8397408 | uc001vrj.1 | CR604228 | UCSC_knowngene | 1459 |
| 0.8399398 | ENST00000392819 | AC016732.2 | Ensembl | 969 |
| 0.8402547 | AI251987 | lincRNA-MYSM1-1 | lincRNA | 204 |
| 0.8403259 | chr14:25152560-25164885- | lincRNA-GZMB | lincRNA | 12326 |
| 0.8404892 | exon679- | lincRNA-METT5D1-4 | lincRNA | 241 |
| 0.8405401 | chr7:127748489-127763889+ | lincRNA-LEP-1 | lincRNA | 15401 |
| 0.8405449 | FN091184 | lincRNA-INHBA-1 | lincRNA | 83 |
| 0.8409132 | uc001lkl.2 | CR592586 | UCSC_knowngene | 870 |
| 0.8417837 | BQ638469 | lincRNA-LEKR1-1 | lincRNA | 586 |
| 0.8418608 | BC042009 |  | misc_RNA | 1288 |
| 0.841952 | chr11:79223852-79229652- | lincRNA-ODZ4 | lincRNA | 5801 |
| 0.8422747 | uc001nng.1 | AX746988 | UCSC_knowngene | 2603 |
| 0.8423292 | NR_023919 | HSPC157 | RefSeq_NR | 1039 |
| 0.8433339 | CB047659 | lincRNA-JARID2-2 | lincRNA | 386 |
| 0.8435046 | AK125219 | lincRNA-HAUS7 | lincRNA | 2124 |
| 0.8435199 | exon2436+ | lincRNA-PTPN1-2 | lincRNA | 264 |
| 0.8435692 | BX104325 | lincRNA-FAM102B-1 | lincRNA | 451 |
| 0.8442685 | AK291739 | lincRNA-CYSLTR2-1 | lincRNA | 1908 |
| 0.8445047 | ENST00000413151 | AC009505.4 | Ensembl | 424 |
| 0.8450819 | AJ227862 |  | misc_RNA | 457 |
| 0.8453237 | BF379117 | lincRNA-OPRD1 | lincRNA | 350 |
| 0.8453623 | ENST00000450310 | RP3-471C18.2 | Ensembl | 191 |
| 0.8458808 | BX111887 | lincRNA-ZEB1-5 | lincRNA | 500 |
| 0.8459701 | uc003tdd.2 | RP9P | UCSC_knowngene | 1403 |
| 0.8460258 | ENST00000505828 | CTC-349C3.1 | Ensembl | 561 |
| 0.8460293 | uc004dvh.2 | LOC92249 | UCSC_knowngene | 2713 |
| 0.8462667 | chr20:29804014-29834814+ | lincRNA-DEFB115 | lincRNA | 30801 |
| 0.8464768 | AW391396 | lincRNA-ATP11C-2 | lincRNA | 766 |
| 0.8465502 | chr4:1774152-1786902- | lincRNA-TACC3 | lincRNA | 12751 |
| 0.8465596 | uc003qvy.1 | AL832737 | UCSC_knowngene | 3867 |
| 0.8467442 | AW974611 | lincRNA-PARP11-4 | lincRNA | 620 |
| 0.8468275 | ENST00000435408 | HCG9P5 | Ensembl | 225 |
| 0.8468987 | DB034728 | lincRNA-OSGIN1 | lincRNA | 572 |
| 0.8469697 | chr7:25891558-25899479- | lincRNA-NPVF-3 | lincRNA | 7922 |
| 0.8470801 | chr5:49632068-49662143- | lincRNA-HCN1-1 | lincRNA | 30076 |
| 0.8473762 | chr22:39275867-39279307+ | lincRNA-APOBEC3A | lincRNA | 3441 |
| 0.8474943 | AK057381 |  | misc_RNA | 1873 |
| 0.8477327 | AU185412 | lincRNA-LIG3 | lincRNA | 629 |
| 0.8481303 | BG287098 | lincRNA-CCND2-1 | lincRNA | 920 |
| 0.8487246 | DA807888 | lincRNA-LMX1A | lincRNA | 596 |
| 0.849172 | CR745936 | lincRNA-BANF2-1 | lincRNA | 532 |
| 0.8493684 | AK226132 | lincRNA-ZFP106 | lincRNA | 5684 |
| 0.8493842 | BI753279 | lincRNA-EIF4E3-2 | lincRNA | 670 |
| 0.8498109 | CB069168 | lincRNA-GALR1-4 | lincRNA | 507 |
| 0.8499502 | NR_026655 | FLJ12825 | RefSeq_NR | 3942 |
| 0.8499904 | AW167909 | lincRNA-WRNIP1 | lincRNA | 518 |
| 0.8500211 | AI902766 | lincRNA-GPHN-1 | lincRNA | 416 |
| 0.850648 | BG013335 | lincRNA-DIRAS2-2 | lincRNA | 249 |
| 0.8506622 | chr17:21233207-21252532+ | lincRNA-KCNJ12 | lincRNA | 19326 |
| 0.8507823 | BC035266 | lincRNA-BRD4 | lincRNA | 3255 |
| 0.8509447 | exon2235- | lincRNA-INHBB-4 | lincRNA | 102 |
| 0.8512053 | chr5:172696910-172713472- | lincRNA-NKX2-5-2 | lincRNA | 16563 |
| 0.8517506 | ENST00000480063 | AC083874.3 | Ensembl | 232 |
| 0.8523169 | DA254994 | lincRNA-EMCN | lincRNA | 571 |
| 0.8523828 | uc010fkq.1 | AK124342 | UCSC_knowngene | 2022 |
| 0.8524425 | NR_027114 | LOC285740 | RefSeq_NR | 1915 |
| 0.8524571 | BF808915 | lincRNA-VOPP1 | lincRNA | 284 |
| 0.8525109 | ENST00000419425 | AC006369.2 | Ensembl | 548 |
| 0.8529106 | ENST00000422521 | AC008280.5 | Ensembl | 1115 |
| 0.8529434 | BF002418 | lincRNA-BTG1-3 | lincRNA | 457 |
| 0.8536034 | uc002xyu.1 | CR612603 | UCSC_knowngene | 2570 |
| 0.8538839 | EL949266 | lincRNA-CENPW-2 | lincRNA | 639 |
| 0.853968 | uc003ysg.2 | BC042052 | UCSC_knowngene | 3296 |
| 0.8540815 | chr20:24778575-24790550- | lincRNA-TMEM90B-2 | lincRNA | 11976 |
| 0.8542678 | BF667001 | lincRNA-ST8SIA6 | lincRNA | 879 |
| 0.854839 | BG427746 | lincRNA-VLDLR-2 | lincRNA | 882 |
| 0.8549119 | uc001pcd.2 | AL832007 | UCSC_knowngene | 5341 |
| 0.8553142 | ENST00000476232 | RP11-568K15.1 | Ensembl | 2921 |
| 0.8554895 | ENST00000438692 | AC013400.2 | Ensembl | 2239 |
| 0.8559933 | chr9:31342775-31353225+ | lincRNA-ACO1-3 | lincRNA | 10451 |
| 0.8560163 | ENST00000426539 | AC130710.1 | Ensembl | 1096 |
| 0.8560485 | AK124439 | lincRNA-SLC4A1AP | lincRNA | 1883 |
| 0.8561829 | ENST00000435044 | RP11-490N5.1 | Ensembl | 374 |
| 0.8563592 | chr2:118805569-118808550- | lincRNA-CCDC93 | lincRNA | 2982 |
| 0.8563633 | chr8:53507546-53529327- | lincRNA-FAM150A | lincRNA | 21782 |
| 0.8564069 | BC009060 |  | misc_RNA | 1247 |
| 0.856654 | FN177586 | lincRNA-IRF2BP2-3 | lincRNA | 119 |
| 0.8568094 | AK225978 | lincRNA-SPARC | lincRNA | 1520 |
| 0.8568427 | chr1:198335952-198347302- | lincRNA-NEK7 | lincRNA | 11351 |
| 0.8569562 | CN288152 | lincRNA-SPINK5 | lincRNA | 741 |
| 0.8575223 | AL599814 | lincRNA-ITGA6-1 | lincRNA | 655 |
| 0.8577082 | BU624419 | lincRNA-HAS2-3 | lincRNA | 656 |
| 0.8579322 | ENST00000490750 | RPLP0P2 | Ensembl | 961 |
| 0.8581818 | chr7:1348449-1366599- | lincRNA-UNCX | lincRNA | 18151 |
| 0.8582795 | NR_026857 | FLJ90757 | RefSeq_NR | 4421 |
| 0.8589314 | AI827996 | lincRNA-CDON-2 | lincRNA | 388 |
| 0.8590457 | BI763082 | lincRNA-TBC1D22A | lincRNA | 830 |
| 0.859231 | CR610499 |  | misc_RNA | 2493 |
| 0.8592805 | ENST00000448347 | RP11-282I1.1 | Ensembl | 831 |
| 0.8593586 | BX115137 | lincRNA-LRRC4C-3 | lincRNA | 391 |
| 0.8598294 | BU622891 | lincRNA-ATXN7L1 | lincRNA | 589 |
| 0.860299 | BG185474 | lincRNA-VASH1 | lincRNA | 557 |
| 0.8606098 | chr5:15021475-15045700- | lincRNA-ANKH | lincRNA | 24226 |
| 0.8609015 | BC157840 | lincRNA-SLC45A4 | lincRNA | 3367 |
| 0.8609107 | chr5:137822391-137830175+ | lincRNA-ETF1 | lincRNA | 7785 |
| 0.8616577 | BM041734 | lincRNA-POLA2 | lincRNA | 672 |
| 0.8618383 | BE439827 | lincRNA-MAS1 | lincRNA | 652 |
| 0.8620318 | chr2:143440730-143546030- | lincRNA-LRP1B-1 | lincRNA | 105301 |
| 0.8621882 | ENST00000502027 | AC016525.3 | Ensembl | 2141 |
| 0.8625477 | NR_027456 | LOC100272228 | RefSeq_NR | 3285 |
| 0.863296 | AX721103 |  | misc_RNA | 642 |
| 0.863444 | NR_015369 | FLJ42709 | RefSeq_NR | 3380 |
| 0.8635495 | CV311298 | lincRNA-TP53TG3B | lincRNA | 409 |
| 0.8640133 | BF056782 | lincRNA-ABCB5 | lincRNA | 511 |
| 0.8640563 | uc002rrq.2 | BC042073 | UCSC_knowngene | 1436 |
| 0.8641856 | BM926136 | lincRNA-SERP2-2 | lincRNA | 1101 |
| 0.8642267 | chr10:120035785-120047260- | lincRNA-RAB11FIP2-2 | lincRNA | 11476 |
| 0.8646803 | ENST00000412736 | MEG3 | Ensembl | 1670 |
| 0.8650838 | BX113459 | lincRNA-FUT8-2 | lincRNA | 744 |
| 0.8651216 | BF477013 | lincRNA-CACNA1E-4 | lincRNA | 168 |
| 0.8652011 | AA722342 | lincRNA-TNKS1BP1 | lincRNA | 519 |
| 0.8652115 | BF749301 | lincRNA-ELAC1 | lincRNA | 263 |
| 0.8652256 | chr13:40809650-40814850+ | lincRNA-FOXO1-1 | lincRNA | 5201 |
| 0.8660802 | AW274645 | lincRNA-PPP2R5C-1 | lincRNA | 367 |
| 0.8661405 | chr2:148345005-148356555+ | lincRNA-ACVR2A-4 | lincRNA | 11551 |
| 0.8663283 | BE048608 | lincRNA-RCBTB2-2 | lincRNA | 282 |
| 0.8664166 | ENST00000502001 | RP11-480D4.3 | Ensembl | 1005 |
| 0.8667991 | chr12:132296472-132307497- | lincRNA-SFSWAP-1 | lincRNA | 11026 |
| 0.8670225 | BU664763 | lincRNA-GSDMC-2 | lincRNA | 390 |
| 0.8679923 | chr2:118353605-118365655+ | lincRNA-DDX18 | lincRNA | 12051 |
| 0.8681924 | ENST00000419514 | RP11-366O17.2 | Ensembl | 192 |
| 0.8684774 | ENST00000420185 | RP11-89N17.4 | Ensembl | 1589 |
| 0.8685215 | H50552 | lincRNA-FAN1 | lincRNA | 411 |
| 0.8686698 | ENST00000512170 | RP11-213G21.2 | Ensembl | 500 |
| 0.8688063 | AA628127 | lincRNA-PRDM14 | lincRNA | 354 |
| 0.869092 | chrX:39487152-39491369+ | lincRNA-BCOR-7 | lincRNA | 4218 |
| 0.8697479 | NR_003132 | HSP90AB2P | RefSeq_NR | 4889 |
| 0.8701465 | chr13:103635874-103659174- | lincRNA-ERCC5-1 | lincRNA | 23301 |
| 0.8702019 | BF803219 | lincRNA-RIPK2-4 | lincRNA | 175 |
| 0.8704501 | DB025468 | lincRNA-MAOA-6 | lincRNA | 560 |
| 0.8705957 | NR_015389 | LOC339290 | RefSeq_NR | 3979 |
| 0.8708689 | ENST00000504270 | RP11-564P3.1 | Ensembl | 437 |
| 0.8710476 | BC043263 |  | misc_RNA | 2325 |
| 0.8715431 | chr2:19979944-19990294- | lincRNA-OSR1-1 | lincRNA | 10351 |
| 0.8719055 | BI024558 | lincRNA-INSR | lincRNA | 328 |
| 0.8719844 | DB372250 | lincRNA-INSIG2-1 | lincRNA | 395 |
| 0.8722601 | AK055084 |  | misc_RNA | 2696 |
| 0.872271 | CD172224 | lincRNA-C8orf86 | lincRNA | 576 |
| 0.8725359 | chr16:84296674-84307824+ | lincRNA-WFDC1 | lincRNA | 11151 |
| 0.8725454 | ENST00000438499 | RP11-1M18.1 | Ensembl | 517 |
| 0.8731969 | EG328251 | lincRNA-CYP4F8 | lincRNA | 179 |
| 0.8737223 | AL709726 | lincRNA-TAC4 | lincRNA | 477 |
| 0.8737441 | ENST00000431646 | RP11-157D23.1 | Ensembl | 880 |
| 0.8741602 | chr18:68422120-68432320- | lincRNA-SOCS6-2 | lincRNA | 10201 |
| 0.8743416 | G30682 |  | misc_RNA | 278 |
| 0.8743873 | ENST00000501280 | AC008625.2 | Ensembl | 2751 |
| 0.8748462 | AK096174 |  | misc_RNA | 2220 |
| 0.8750296 | ENST00000510466 | AL136307.1 | Ensembl | 1357 |
| 0.8751656 | ENST00000413525 | AC010090.1 | Ensembl | 362 |
| 0.8751753 | N62920 | lincRNA-SLC2A1-1 | lincRNA | 572 |
| 0.8752765 | chr2:235518511-235598161- | lincRNA-ARL4C-6 | lincRNA | 79651 |
| 0.875352 | DB546676 | lincRNA-CBLN2-1 | lincRNA | 426 |
| 0.8755041 | ENST00000432412 | AP000998.2 | Ensembl | 478 |
| 0.8755406 | AK130515 |  | misc_RNA | 3369 |
| 0.8755645 | BG977395 | lincRNA-PSTPIP1 | lincRNA | 207 |
| 0.8757427 | BF986127 | lincRNA-FOXN2-1 | lincRNA | 145 |
| 0.8762074 | uc002msx.1 | AX747405 | UCSC_knowngene | 1801 |
| 0.8765197 | chr3:118559485-118571910- | lincRNA-LSAMP-6 | lincRNA | 12426 |
| 0.8767331 | chr6:13349175-13352025- | lincRNA-TBC1D7 | lincRNA | 2851 |
| 0.8768914 | uc001znn.1 | LOC729082 | UCSC_knowngene | 1379 |
| 0.8769362 | chr3:45207146-45221396- | lincRNA-CDCP1 | lincRNA | 14251 |
| 0.8771504 | ENST00000424592 | RP11-63G10.2 | Ensembl | 395 |
| 0.8772877 | DA430048 | lincRNA-PLXNA2-1 | lincRNA | 563 |
| 0.8777073 | CR616945 | lincRNA-ZNF385B-1 | lincRNA | 1801 |
| 0.8778267 | uc001czf.2 | AK055150 | UCSC_knowngene | 1123 |
| 0.8779071 | AI201012 | lincRNA-ENOX1 | lincRNA | 453 |
| 0.8779342 | Z41842 | lincRNA-RASGRF1-2 | lincRNA | 336 |
| 0.8780047 | CF887548 | lincRNA-NEDD1-3 | lincRNA | 738 |
| 0.8781665 | chr5:67603294-67617869+ | lincRNA-SLC30A5-5 | lincRNA | 14576 |
| 0.8781777 | BU663707 | lincRNA-RANBP3L-1 | lincRNA | 654 |
| 0.8782396 | uc002uwa.2 | AOX2 | UCSC_knowngene | 978 |
| 0.8784924 | exon99- | lincRNA-C1orf83 | lincRNA | 715 |
| 0.8787862 | uc003xyc.1 | AK055514 | UCSC_knowngene | 2367 |
| 0.879002 | AI024083 | lincRNA-GRHL2 | lincRNA | 344 |
| 0.8794827 | exon2751+ | lincRNA-TPRA1-4 | lincRNA | 101 |
| 0.8797993 | BC068090 |  | misc_RNA | 1322 |
| 0.8799011 | chr13:73819474-73846199+ | lincRNA-KLF12-10 | lincRNA | 26726 |
| 0.8799729 | AK001439 |  | NRED | 2742 |
| 0.8800599 | chr5:148834826-148846630- | lincRNA-IL17B | lincRNA | 11805 |
| 0.8801938 | ENST00000445666 | RP3-389A20.2 | Ensembl | 394 |
| 0.8803318 | ENST00000514392 | RP11-478C6.3 | Ensembl | 1582 |
| 0.8806848 | uc003jnb.1 | AK056817 | UCSC_knowngene | 2757 |
| 0.8807257 | BQ185896 | lincRNA-NPAS4 | lincRNA | 555 |
| 0.8809748 | BQ303919 | lincRNA-RGNEF-3 | lincRNA | 549 |
| 0.8810525 | chr14:76020622-76030872+ | lincRNA-FLVCR2 | lincRNA | 10251 |
| 0.8810899 | AW387651 | lincRNA-TFE3 | lincRNA | 232 |
| 0.8812665 | ENST00000427447 | AP000569.8 | Ensembl | 9124 |
| 0.8813447 | chr13:24122375-24133325- | lincRNA-SACS | lincRNA | 10951 |
| 0.881445 | chr3:193447028-193465304- | lincRNA-OPA1-2 | lincRNA | 18277 |
| 0.8814937 | chr2:3172193-3185593- | lincRNA-MYT1L-1 | lincRNA | 13401 |
| 0.8815442 | chr7:117584264-117586960+ | lincRNA-NAA38 | lincRNA | 2697 |
| 0.8817348 | ENST00000422047 | RP11-245M24.1 | Ensembl | 639 |
| 0.8819479 | uc001zae.2 | SNURF-SNRPN | UCSC_knowngene | 4150 |
| 0.8822587 | uc002ike.2 | LOC644246 | UCSC_knowngene | 513 |
| 0.8825312 | ENST00000512322 | RP11-792D21.2 | Ensembl | 2095 |
| 0.882624 | uc003eoc.1 | BC043572 | UCSC_knowngene | 1768 |
| 0.882667 | AK022887 |  | RNAdb | 2629 |
| 0.8827052 | ENST00000412020 | RP11-12D24.8 | Ensembl | 244 |
| 0.8828205 | AA584758 | lincRNA-LGALS3-1 | lincRNA | 411 |
| 0.8829142 | DB530028 | lincRNA-MMP16-2 | lincRNA | 439 |
| 0.8832831 | AF083120 |  | misc_RNA | 158 |
| 0.8833549 | DA673588 | lincRNA-LITAF-3 | lincRNA | 555 |
| 0.8835269 | NR_027345 | NCRNA00173 | RefSeq_NR | 1597 |
| 0.8840083 | chr2:72079817-72089167- | lincRNA-DYSF | lincRNA | 9351 |
| 0.8840263 | chr2:232219356-232231081- | lincRNA-ARMC9 | lincRNA | 11726 |
| 0.8840387 | NR_015442 | LOC401397 | RefSeq_NR | 1055 |
| 0.8841789 | chr4:5995874-6009524- | lincRNA-CRMP1-2 | lincRNA | 13651 |
| 0.8846544 | DB049930 | lincRNA-PDP1-2 | lincRNA | 551 |
| 0.8846817 | chr3:182007053-182019242+ | lincRNA-ATP11B-2 | lincRNA | 12190 |
| 0.8846826 | uc003oci.1 | AK057104 | UCSC_knowngene | 3143 |
| 0.8851372 | AW293169 | lincRNA-ZNF507 | lincRNA | 480 |
| 0.8853418 | BE551986 | lincRNA-PIRT | lincRNA | 455 |
| 0.8856422 | uc002ulk.1 | DKFZp451M2119 | UCSC_knowngene | 1187 |
| 0.8862469 | ENST00000423797 | AC006038.4 | Ensembl | 603 |
| 0.8863292 | chr1:108595980-108600811- | lincRNA-VAV3-1 | lincRNA | 4832 |
| 0.8864987 | DA758158 | lincRNA-ACSS3 | lincRNA | 535 |
| 0.886672 | AI767623 | lincRNA-CLLU1OS-1 | lincRNA | 466 |
| 0.8869618 | BF680399 | lincRNA-USP54 | lincRNA | 466 |
| 0.8870855 | ENST00000440725 | AC090421.2 | Ensembl | 580 |
| 0.8872485 | DA662617 | lincRNA-GALNT10-1 | lincRNA | 524 |
| 0.8881418 | ENST00000457116 | RP5-991C6.4 | Ensembl | 471 |
| 0.888308 | DB340468 | lincRNA-KLF13-2 | lincRNA | 578 |
| 0.8884326 | ENST00000505358 | AC004611.1 | Ensembl | 1207 |
| 0.8885512 | uc001yfe.2 | BC016484 | UCSC_knowngene | 3144 |
| 0.8895061 | BE092230 | lincRNA-ARL4C-1 | lincRNA | 346 |
| 0.889736 | CA433478 | lincRNA-SLC22A23 | lincRNA | 409 |
| 0.889833 | ENST00000444462 | RP11-423O2.3 | Ensembl | 288 |
| 0.8898497 | ENST00000477321 | RP11-289K10.1 | Ensembl | 437 |
| 0.8901089 | CV356047 | lincRNA-MMS22L-1 | lincRNA | 554 |
| 0.8903556 | ENST00000414488 | AL132709.5 | Ensembl | 800 |
| 0.8908401 | ENST00000440922 | GOT2L1 | Ensembl | 1310 |
| 0.8913476 | exon99+ | lincRNA-CDCP2 | lincRNA | 715 |
| 0.8913707 | BQ027881 | lincRNA-LIPG-1 | lincRNA | 485 |
| 0.8914713 | CD243636 | lincRNA-C5orf30-2 | lincRNA | 853 |
| 0.8915992 | AK296158 | lincRNA-TMEM30B-1 | lincRNA | 1756 |
| 0.8916877 | ENST00000484010 | RP11-428G5.2 | Ensembl | 494 |
| 0.8923622 | chr4:53257568-53279668- | lincRNA-SPATA18-1 | lincRNA | 22101 |
| 0.8925537 | uc010aja.1 | TCRA | UCSC_knowngene | 583 |
| 0.8930604 | BX373435 | lincRNA-FLI1 | lincRNA | 904 |
| 0.8933299 | DR159293 | lincRNA-DCAF12L2-2 | lincRNA | 722 |
| 0.8933953 | chr13:69265749-69290074+ | lincRNA-KLHL1-1 | lincRNA | 24326 |
| 0.8934067 | ENST00000423543 | RP3-453A3.1 | Ensembl | 382 |
| 0.8934481 | ENST00000502344 | RP11-665G4.1 | Ensembl | 630 |
| 0.893712 | AK026861 |  | misc_RNA | 1334 |
| 0.8945048 | chr4:25089927-25101952+ | lincRNA-SEPSECS-1 | lincRNA | 12026 |
| 0.894611 | AK024617 |  | misc_RNA | 1862 |
| 0.8949471 | BM805159 | lincRNA-THSD7A-2 | lincRNA | 1079 |
| 0.8950201 | chr4:60514780-60549030- | lincRNA-IGFBP7-3 | lincRNA | 34251 |
| 0.8951043 | DB341809 | lincRNA-CCDC54-2 | lincRNA | 544 |
| 0.8954132 | CA442294 | lincRNA-PARD3 | lincRNA | 693 |
| 0.8958778 | CN267256 | lincRNA-KCNRG-1 | lincRNA | 606 |
| 0.896084 | DB090998 | lincRNA-GPHN-3 | lincRNA | 556 |
| 0.8961742 | ENST00000447262 | AC006481.1 | Ensembl | 432 |
| 0.8962036 | AA368654 | lincRNA-OPTC | lincRNA | 281 |
| 0.8964424 | chr1:157374626-157390451- | lincRNA-ETV3-1 | lincRNA | 15826 |
| 0.8964988 | ENST00000426519 | RP11-276E17.2 | Ensembl | 701 |
| 0.8965448 | ENST00000413983 | RP11-410N8.3 | Ensembl | 469 |
| 0.8966563 | BG428088 | lincRNA-MCTP2-1 | lincRNA | 745 |
| 0.8968437 | BI830735 | lincRNA-ZNF391 | lincRNA | 899 |
| 0.8969795 | DB307684 | lincRNA-C8orf37-1 | lincRNA | 518 |
| 0.8971244 | CK819477 | lincRNA-APLN-2 | lincRNA | 356 |
| 0.8974871 | ENST00000452496 | ASS1P12 | Ensembl | 1227 |
| 0.8978615 | chr2:105731762-105737443+ | lincRNA-GPR45 | lincRNA | 5682 |
| 0.898083 | BI756930 | lincRNA-GLRA4 | lincRNA | 848 |
| 0.898258 | chr5:82684694-82704544+ | lincRNA-VCAN | lincRNA | 19851 |
| 0.8984504 | chr1:13902316-13904644- | lincRNA-LRRC38 | lincRNA | 2329 |
| 0.8986302 | AW418755 | lincRNA-APLF | lincRNA | 635 |
| 0.8990866 | U26161 | lincRNA-FKBP4 | lincRNA | 422 |
| 0.8991616 | CA422183 | lincRNA-MED4-2 | lincRNA | 681 |
| 0.899662 | chr12:60208733-60258008+ | lincRNA-FAM19A2-1 | lincRNA | 49276 |
| 0.8999894 | chr10:95567958-95575624+ | lincRNA-TMEM20-2 | lincRNA | 7667 |
| 0.9000401 | BF737354 | lincRNA-VDAC2 | lincRNA | 545 |
| 0.9002673 | CV311274 | lincRNA-BLID-3 | lincRNA | 153 |
| 0.9002762 | uc010ssu.1 | BC043551 | UCSC_knowngene | 724 |
| 0.9003547 | chr1:203164802-203174902+ | lincRNA-CHIT1 | lincRNA | 10101 |
| 0.9009009 | uc003yed.2 | CR590356 | UCSC_knowngene | 1430 |
| 0.9011529 | AK055743 |  | misc_RNA | 1824 |
| 0.901166 | chr12:38862405-38887736- | lincRNA-ALG10B-4 | lincRNA | 25332 |
| 0.9012798 | DB522844 | lincRNA-LOC100131199 | lincRNA | 463 |
| 0.9013962 | chr10:60771715-60817064- | lincRNA-BICC1 | lincRNA | 45350 |
| 0.9016844 | chr1:95438681-95440571- | lincRNA-CNN3 | lincRNA | 1891 |
| 0.9017417 | CA389504 | lincRNA-ARHGEF3 | lincRNA | 721 |
| 0.9018466 | uc001qhs.2 | BC112333 | UCSC_knowngene | 1946 |
| 0.9020661 | chr7:93805214-93810289+ | lincRNA-COL1A2-2 | lincRNA | 5076 |
| 0.9021049 | AK021558 |  | misc_RNA | 1746 |
| 0.9022335 | AI208830 | lincRNA-OLFM4-6 | lincRNA | 409 |
| 0.9024277 | ENST00000438775 | AC087763.1 | Ensembl | 303 |
| 0.9024404 | AK026734 |  | misc_RNA | 2218 |
| 0.9025027 | chr2:134708080-134794155+ | lincRNA-MGAT5-3 | lincRNA | 86076 |
| 0.9026069 | BI522654 | lincRNA-SLC20A1-1 | lincRNA | 846 |
| 0.9029304 | AA326780 | lincRNA-GRB14 | lincRNA | 285 |
| 0.9029526 | AW270097 | lincRNA-PDK4 | lincRNA | 450 |
| 0.9030104 | DB311472 | lincRNA-C14orf147-2 | lincRNA | 511 |
| 0.9031317 | ENST00000446594 | AC012512.1 | Ensembl | 718 |
| 0.9033547 | uc002ioy.1 | AK097622 | UCSC_knowngene | 1754 |
| 0.9035868 | chr19:6516276-6522541- | lincRNA-TUBB4 | lincRNA | 6266 |
| 0.9039292 | uc001czt.1 | AK097193 | UCSC_knowngene | 2344 |
| 0.9041097 | uc010txe.1 | MEG3 | UCSC_knowngene | 1760 |
| 0.9043261 | DA422275 | lincRNA-BET1-3 | lincRNA | 561 |
| 0.9043818 | ENST00000441473 | RP11-343J18.2 | Ensembl | 316 |
| 0.9044918 | ENST00000381279 | RP11-15H7.2 | Ensembl | 1580 |
| 0.9045835 | ENST00000445336 | RP11-451L9.3 | Ensembl | 2725 |
| 0.9049384 | AK130123 | lincRNA-PPP2R2A | lincRNA | 1823 |
| 0.9052286 | chr14:51888100-51898875- | lincRNA-TMX1-2 | lincRNA | 10776 |
| 0.9055564 | CA941849 | lincRNA-TSTD1 | lincRNA | 83 |
| 0.9055613 | CV346902 | lincRNA-TMEM45B-1 | lincRNA | 589 |
| 0.9061842 | AL162037 |  | misc_RNA | 3019 |
| 0.9065163 | DB299428 | lincRNA-PAIP2B | lincRNA | 543 |
| 0.9065505 | ENST00000416785 | RPSAP2 | Ensembl | 888 |
| 0.9065883 | AA769057 | lincRNA-UBE2G1 | lincRNA | 456 |
| 0.9066665 | uc001uyq.1 | AK054970 | UCSC_knowngene | 3681 |
| 0.9068982 | ENST00000446220 | RP11-10N16.3 | Ensembl | 2337 |
| 0.9069827 | chr3:193577911-193589292+ | lincRNA-HES1-2 | lincRNA | 11382 |
| 0.9070569 | CR604878 |  | NRED | 1220 |
| 0.9071072 | CR739801 | lincRNA-PARN | lincRNA | 746 |
| 0.907177 | uc004fai.1 | AK055694 | UCSC_knowngene | 2222 |
| 0.9071945 | DB455167 | lincRNA-C6orf99 | lincRNA | 480 |
| 0.9072161 | exon680- | lincRNA-METT5D1-4 | lincRNA | 225 |
| 0.9075301 | AY927639 |  | RNAdb | 1483 |
| 0.9076145 | uc001mkl.1 | AK001432 | UCSC_knowngene | 1558 |
| 0.907761 | BF575745 | lincRNA-MED13L-5 | lincRNA | 818 |
| 0.908243 | NR_024444 | LOC100133985 | RefSeq_NR | 661 |
| 0.9082666 | AA219474 | lincRNA-RUNX2-2 | lincRNA | 361 |
| 0.9082678 | DA088898 | lincRNA-INCENP | lincRNA | 560 |
| 0.908378 | DA492035 | lincRNA-GTDC1 | lincRNA | 564 |
| 0.9087156 | uc001yfx.2 | C14orf64 | UCSC_knowngene | 2166 |
| 0.90888 | ENST00000450527 | RP11-342D14.1 | Ensembl | 722 |
| 0.9096272 | NR_002791 | EMX2OS | RefSeq_NR | 7282 |
| 0.9100112 | uc003bqj.1 | AF279782 | UCSC_knowngene | 1688 |
| 0.9101059 | chr1:235153477-235164377+ | lincRNA-TOMM20-4 | lincRNA | 10901 |
| 0.910302 | BM008250 | lincRNA-FOXE1-2 | lincRNA | 879 |
| 0.910379 | ENST00000446763 | AL035422.2 | Ensembl | 403 |
| 0.910437 | DB036467 | lincRNA-TPCN2 | lincRNA | 579 |
| 0.910548 | chr3:127258794-127273334+ | lincRNA-TPRA1-1 | lincRNA | 14541 |
| 0.9105492 | uc003uip.3 | P53TG1 | UCSC_knowngene | 659 |
| 0.9107262 | ENST00000424605 | RP11-125B21.2 | Ensembl | 746 |
| 0.9115968 | DB445849 | lincRNA-DYNC1I1 | lincRNA | 466 |
| 0.9122274 | AA992250 | lincRNA-ERBB2IP-2 | lincRNA | 552 |
| 0.9125375 | AK094629 |  | misc_RNA | 2028 |
| 0.9128307 | BP232819 | lincRNA-EPHB1-1 | lincRNA | 582 |
| 0.913189 | uc001ooz.1 | AK094674 | UCSC_knowngene | 2376 |
| 0.9132658 | chr15:35025782-35036455+ | lincRNA-GJD2-1 | lincRNA | 10674 |
| 0.9135073 | chr12:47935555-47955076+ | lincRNA-RPAP3-2 | lincRNA | 19522 |
| 0.913732 | ENST00000455390 | RP11-514O12.2 | Ensembl | 627 |
| 0.9143257 | DA194797 | lincRNA-ZMIZ1-6 | lincRNA | 549 |
| 0.9143566 | AK091593 |  | NRED | 3013 |
| 0.9144936 | DB061937 | lincRNA-SHH-4 | lincRNA | 564 |
| 0.9144945 | exon1609+ | lincRNA-PPP2R5C-2 | lincRNA | 198 |
| 0.9145774 | chr11:44720724-44741649+ | lincRNA-TSPAN18 | lincRNA | 20926 |
| 0.9153048 | chr7:1348449-1366599+ | lincRNA-MICALL2 | lincRNA | 18151 |
| 0.9154425 | ENST00000468834 | RP11-462L8.2 | Ensembl | 793 |
| 0.9157883 | chr6:140218607-140237032- | lincRNA-CITED2-1 | lincRNA | 18426 |
| 0.9159577 | AK126994 | lincRNA-PLEKHM1-2 | lincRNA | 3857 |
| 0.9162173 | exon94- | lincRNA-DMBX1 | lincRNA | 161 |
| 0.9162572 | CR743903 | lincRNA-WDR5-1 | lincRNA | 508 |
| 0.9163619 | AK128360 |  | misc_RNA | 2848 |
| 0.9172247 | BX101441 | lincRNA-C10orf46-1 | lincRNA | 712 |
| 0.9176051 | AK297025 | lincRNA-FGD4 | lincRNA | 2924 |
| 0.9178499 | chr7:73684091-73687309- | lincRNA-RFC2 | lincRNA | 3219 |
| 0.9178957 | GD141660 | lincRNA-BAZ1A | lincRNA | 274 |
| 0.9190627 | BX110954 | lincRNA-ZNF572-1 | lincRNA | 620 |
| 0.919102 | ENST00000414613 | AC010733.8 | Ensembl | 1231 |
| 0.9194818 | ENST00000471496 | RP11-393B14.1 | Ensembl | 1490 |
| 0.9198813 | AA972029 | lincRNA-MPDZ-1 | lincRNA | 419 |
| 0.9203045 | ENST00000453558 | HCG17 | Ensembl | 732 |
| 0.9203626 | chr12:61176358-61214183- | lincRNA-SLC16A7-3 | lincRNA | 37826 |
| 0.9204835 | DA233116 | lincRNA-KITLG-2 | lincRNA | 599 |
| 0.9217367 | exon848+ | lincRNA-ZW10 | lincRNA | 153 |
| 0.9219964 | NR_033312 | BDNFOS | RefSeq_NR | 2036 |
| 0.9220145 | BF739255 | lincRNA-APLN-1 | lincRNA | 163 |
| 0.9220851 | exon98+ | lincRNA-CDCP2 | lincRNA | 176 |
| 0.9223942 | CN294846 | lincRNA-STC1-2 | lincRNA | 252 |
| 0.9225663 | AI073577 | lincRNA-PDZRN4-1 | lincRNA | 331 |
| 0.9226436 | BQ184470 | lincRNA-EDEM3 | lincRNA | 542 |
| 0.9226648 | BU621051 | lincRNA-PPARGC1A-2 | lincRNA | 726 |
| 0.9228185 | exon2436- | lincRNA-CEBPB-1 | lincRNA | 264 |
| 0.9229976 | ENST00000426911 | RP11-308D16.3 | Ensembl | 1221 |
| 0.9230641 | ENST00000448650 | AC008069.1 | Ensembl | 596 |
| 0.9231211 | CR738571 | lincRNA-NCOR2 | lincRNA | 504 |
| 0.9232194 | uc002ilc.1 | CR602880 | UCSC_knowngene | 2080 |
| 0.9234951 | AK310205 | lincRNA-SLC15A1 | lincRNA | 1239 |
| 0.9235669 | ENST00000507241 | CTC-428G20.3 | Ensembl | 2231 |
| 0.9241523 | ENST00000428151 | RP5-884C9.2 | Ensembl | 573 |
| 0.9241532 | chr2:91595323-91623173+ | lincRNA-TEKT4-3 | lincRNA | 27851 |
| 0.9244211 | uc001vdo.1 | BCMSUN | UCSC_knowngene | 3068 |
| 0.9244433 | ENST00000456771 | RP11-540K16.2 | Ensembl | 532 |
| 0.9245251 | chr6:150606082-150619632- | lincRNA-PPP1R14C | lincRNA | 13551 |
| 0.9255073 | ENST00000509530 | CTC-276P9.3 | Ensembl | 541 |
| 0.9255916 | ENST00000504119 | AL133167.1 | Ensembl | 2930 |
| 0.9256053 | ENST00000413926 | RP11-547C18.2 | Ensembl | 606 |
| 0.925621 | AX747237 | lincRNA-KIFC3 | lincRNA | 3159 |
| 0.925709 | ENST00000437598 | RP5-1027O11.1 | Ensembl | 787 |
| 0.9258456 | NR_015353 | LOC92249 | RefSeq_NR | 2886 |
| 0.9260343 | NR_026968 | LOC285456 | RefSeq_NR | 3773 |
| 0.9261144 | chr13:77046674-77155049- | lincRNA-LMO7-1 | lincRNA | 108376 |
| 0.9264669 | ENST00000416845 | AC093159.1 | Ensembl | 424 |
| 0.9264754 | chr3:33273197-33275601+ | lincRNA-FBXL2 | lincRNA | 2405 |
| 0.9266385 | chr10:31568469-31585244+ | lincRNA-ZEB1-2 | lincRNA | 16776 |
| 0.9267918 | AK292905 | lincRNA-PHC2-1 | lincRNA | 3360 |
| 0.9267939 | AK054588 |  | misc_RNA | 1826 |
| 0.9269374 | BE501942 | lincRNA-MRPL36-2 | lincRNA | 508 |
| 0.9269637 | chr11:93702877-93731152- | lincRNA-C11orf90 | lincRNA | 28276 |
| 0.9270037 | exon2995+ | lincRNA-FOXD1-2 | lincRNA | 180 |
| 0.9272945 | CB216772 | lincRNA-KCNIP1-2 | lincRNA | 603 |
| 0.9273346 | AA128631 | lincRNA-ADAMTS20 | lincRNA | 448 |
| 0.9273675 | CB265955 | lincRNA-RAB4A-5 | lincRNA | 616 |
| 0.9275429 | NR_002809 | LOC338799 | RefSeq_NR | 1521 |
| 0.9276507 | chr6:47317091-47336991+ | lincRNA-CD2AP-1 | lincRNA | 19901 |
| 0.9278773 | ENST00000439897 | RP11-91I11.1 | Ensembl | 151 |
| 0.9286439 | exon847+ | lincRNA-ZW10 | lincRNA | 440 |
| 0.9287014 | NR_004053 | SOX2OT | RefSeq_NR | 2529 |
| 0.9287469 | uc001vlo.2 | BC038529 | UCSC_knowngene | 1000 |
| 0.9288622 | CU691912 | lincRNA-PRR14 | lincRNA | 1160 |
| 0.929074 | BG538918 | lincRNA-BCL6-2 | lincRNA | 886 |
| 0.9292219 | NR_027301 | LOC148189 | RefSeq_NR | 2085 |
| 0.9292914 | AA054316 | lincRNA-FA2H-1 | lincRNA | 470 |
| 0.9296728 | NR_024586 | LOC100216545 | RefSeq_NR | 3615 |
| 0.9299011 | BQ708152 | lincRNA-EYA1 | lincRNA | 967 |
| 0.9300908 | chr18:47271277-47283077+ | lincRNA-ACAA2-2 | lincRNA | 11801 |
| 0.9301987 | chr1:2952104-2969716+ | lincRNA-PRDM16 | lincRNA | 17613 |
| 0.930418 | NR_026743 | tAKR | RefSeq_NR | 1408 |
| 0.9305212 | NR_026804 | FLJ13197 | RefSeq_NR | 2368 |
| 0.930527 | uc001uiy.2 | LOC338797 | UCSC_knowngene | 1795 |
| 0.9311012 | ENST00000423422 | RP11-336K24.4 | Ensembl | 420 |
| 0.931415 | ENST00000505910 | RP11-723O4.3 | Ensembl | 329 |
| 0.9314502 | BI059095 | lincRNA-UBASH3B-1 | lincRNA | 330 |
| 0.9314847 | ENST00000441053 | AC067960.1 | Ensembl | 812 |
| 0.9321126 | BF895724 | lincRNA-C4orf41 | lincRNA | 426 |
| 0.9322014 | ENST00000507589 | AC025370.2 | Ensembl | 2742 |
| 0.9324186 | chr12:38442633-38454033+ | lincRNA-ALG10B-2 | lincRNA | 11401 |
| 0.9326198 | exon1959+ | lincRNA-RBBP8-2 | lincRNA | 93 |
| 0.9328654 | exon4209- | lincRNA-NIPAL2-2 | lincRNA | 155 |
| 0.9335807 | chr13:77221824-77235874- | lincRNA-LMO7-4 | lincRNA | 14051 |
| 0.933836 | AL832758 |  | NRED | 5277 |
| 0.9340418 | ENST00000427353 | AC115283.1 | Ensembl | 400 |
| 0.9341405 | AK226157 | lincRNA-EHF | lincRNA | 4584 |
| 0.934281 | CA421426 | lincRNA-IRX5 | lincRNA | 632 |
| 0.9342843 | uc003opf.1 | BC132805 | UCSC_knowngene | 3122 |
| 0.9343763 | AK091174 |  | misc_RNA | 2350 |
| 0.9344554 | ENST00000445427 | RP11-5N23.2 | Ensembl | 1133 |
| 0.9347874 | exon1338+ | lincRNA-PRR20A-4 | lincRNA | 181 |
| 0.9351507 | ENST00000511331 | CTC-338M12.4 | Ensembl | 1000 |
| 0.9354751 | AI890481 | lincRNA-CTAGE5 | lincRNA | 538 |
| 0.935489 | BI020545 | lincRNA-C14orf4-1 | lincRNA | 164 |
| 0.9356253 | ENST00000446115 | RP11-439L18.3 | Ensembl | 783 |
| 0.9357579 | ENST00000438536 | RP11-427O13.1 | Ensembl | 1800 |
| 0.9362734 | BG210561 | lincRNA-AGRP-2 | lincRNA | 697 |
| 0.9364873 | AF315716 |  | misc_RNA | 336 |
| 0.9366088 | BG677629 | lincRNA-ARSJ-2 | lincRNA | 928 |
| 0.9370491 | chr16:87577874-87589624- | lincRNA-ZCCHC14 | lincRNA | 11751 |
| 0.9372331 | BE567543 | lincRNA-BCOR-8 | lincRNA | 649 |
| 0.9372946 | ENST00000454208 | AC092640.1 | Ensembl | 705 |
| 0.9372964 | ENST00000414382 | AC098973.2 | Ensembl | 543 |
| 0.9374299 | chr7:136275537-136279697- | lincRNA-LUZP6-2 | lincRNA | 4161 |
| 0.9376479 | AW449673 | lincRNA-PRSS23-3 | lincRNA | 461 |
| 0.9376728 | ENST00000441214 | RP11-290F20.2 | Ensembl | 2250 |
| 0.9380532 | NR_033378 | LOC100288428 | RefSeq_NR | 2147 |
| 0.9380565 | CA412304 | lincRNA-COPS2 | lincRNA | 754 |
| 0.9380924 | AK309134 | lincRNA-KATNAL2 | lincRNA | 1246 |
| 0.9381194 | uc002ytn.2 | CR626360 | UCSC_knowngene | 2657 |
| 0.9381333 | ENST00000503532 | RP11-341G5.1 | Ensembl | 582 |
| 0.9382518 | AK129753 |  | misc_RNA | 2007 |
| 0.9384278 | chr11:102425615-102440915+ | lincRNA-MMP20 | lincRNA | 15301 |
| 0.9384542 | ENST00000453082 | SNORD115-26 | Ensembl | 6087 |
| 0.9386618 | ENST00000441336 | RP11-273F15.1 | Ensembl | 220 |
| 0.9390786 | DA428052 | lincRNA-UBE2CBP-2 | lincRNA | 571 |
| 0.9394025 | AI420530 | lincRNA-TCF4-4 | lincRNA | 350 |
| 0.9401372 | ENST00000444977 | AP001434.2 | Ensembl | 287 |
| 0.9405991 | AA757150 | lincRNA-MBNL2-2 | lincRNA | 508 |
| 0.9410925 | AL049252 |  | NRED | 3343 |
| 0.9413095 | chr3:58165366-58172726+ | lincRNA-DNASE1L3 | lincRNA | 7361 |
| 0.9415427 | ENST00000512421 | AC006499.1 | Ensembl | 1117 |
| 0.9419173 | BQ706775 | lincRNA-KCNA5-2 | lincRNA | 963 |
| 0.942017 | DB458469 | lincRNA-C9orf150 | lincRNA | 483 |
| 0.942452 | chr7:45323806-45407638+ | lincRNA-ADCY1-1 | lincRNA | 83833 |
| 0.9425618 | ENST00000449602 | AJ006998.2 | Ensembl | 1000 |
| 0.9429759 | T50138 | lincRNA-ZNF716-1 | lincRNA | 395 |
| 0.9433981 | BX648912 | lincRNA-GJB4 | lincRNA | 2619 |
| 0.9436412 | chr13:58433499-58448524+ | lincRNA-DIAPH3 | lincRNA | 15026 |
| 0.9437057 | AI252667 | lincRNA-WNT11 | lincRNA | 438 |
| 0.9437309 | chr6:170239050-170268025- | lincRNA-C6orf70-1 | lincRNA | 28976 |
| 0.943783 | BF356369 | lincRNA-THBS1-2 | lincRNA | 288 |
| 0.9440599 | uc001uzl.2 | BC025370 | UCSC_knowngene | 976 |
| 0.9440782 | ENST00000451203 | AC104843.3 | Ensembl | 319 |
| 0.9441208 | BG197454 | lincRNA-NRSN1-4 | lincRNA | 501 |
| 0.9442079 | ENST00000447933 | AC073871.2 | Ensembl | 766 |
| 0.9448635 | NR_024037 | RMST | RefSeq_NR | 2099 |
| 0.9449046 | ENST00000441207 | RP4-742C19.11 | Ensembl | 421 |
| 0.9449173 | CV356473 | lincRNA-RIPK2-2 | lincRNA | 640 |
| 0.944946 | AW516314 | lincRNA-HACL1 | lincRNA | 496 |
| 0.9449524 | CD676340 | lincRNA-FAM84B-3 | lincRNA | 634 |
| 0.9450012 | chrX:45555906-45580831+ | lincRNA-ZNF673-5 | lincRNA | 24926 |
| 0.9450872 | EB386665 | lincRNA-SLN | lincRNA | 821 |
| 0.9451182 | chr5:92312320-92317777- | lincRNA-ARRDC3-5 | lincRNA | 5458 |
| 0.9451633 | chr3:46129526-46140202+ | lincRNA-CCR1 | lincRNA | 10677 |
| 0.9451726 | DB345908 | lincRNA-CYTIP | lincRNA | 547 |
| 0.9452131 | ENST00000446527 | RP11-421L21.3 | Ensembl | 1045 |
| 0.9452278 | chr20:541348-562112+ | lincRNA-TCF15 | lincRNA | 20765 |
| 0.9457048 | DQ916738 | lincRNA-ZC3H12C-2 | lincRNA | 2761 |
| 0.9457195 | DB100323 | lincRNA-ACOXL | lincRNA | 593 |
| 0.9462044 | BE780050 | lincRNA-MPHOSPH8-2 | lincRNA | 727 |
| 0.9462182 | AK092053 |  | misc_RNA | 2347 |
| 0.94647 | BQ446632 | lincRNA-KCNU1-3 | lincRNA | 496 |
| 0.9466395 | ENST00000419357 | C6orf214 | Ensembl | 264 |
| 0.9468949 | ENST00000361447 | RP11-47K11.3 | Ensembl | 1081 |
| 0.9469665 | DB349013 | lincRNA-DCTD | lincRNA | 546 |
| 0.9472574 | chr3:168554930-168560248+ | lincRNA-MECOM-1 | lincRNA | 5319 |
| 0.9473885 | chr7:125224214-125321514+ | lincRNA-GRM8-1 | lincRNA | 97301 |
| 0.9475442 | BF445802 | lincRNA-NR2F2-3 | lincRNA | 375 |
| 0.947554 | chr8:132764618-132856568- | lincRNA-ADCY8-2 | lincRNA | 91951 |
| 0.9476138 | chr13:77909674-77931274+ | lincRNA-SCEL | lincRNA | 21601 |
| 0.9477003 | AA827066 | lincRNA-KIRREL3-1 | lincRNA | 472 |
| 0.9477902 | BG199702 | lincRNA-FAM19A2-5 | lincRNA | 788 |
| 0.947803 | BM716387 | lincRNA-STRA8 | lincRNA | 437 |
| 0.9478126 | AF086167 |  | misc_RNA | 550 |
| 0.9480666 | uc001jef.2 | LOC643650 | UCSC_knowngene | 2876 |
| 0.9481371 | ENST00000411878 | AF254982.2 | Ensembl | 311 |
| 0.9483968 | ENST00000468910 | RP11-12N13.4 | Ensembl | 766 |
| 0.9484731 | CD656234 | lincRNA-CWC15 | lincRNA | 688 |
| 0.9485568 | DB337946 | lincRNA-ZBTB37-2 | lincRNA | 538 |
| 0.948612 | ENST00000418416 | AC079163.1 | Ensembl | 407 |
| 0.9487596 | AA483651 | lincRNA-CD226-2 | lincRNA | 347 |
| 0.9490338 | ENST00000433054 | RP11-1O7.1 | Ensembl | 884 |
| 0.9492567 | chr10:102402035-102412460+ | lincRNA-PAX2-3 | lincRNA | 10426 |
| 0.9493863 | DA942624 | lincRNA-KIAA0146-1 | lincRNA | 581 |
| 0.9499036 | AA054416 | lincRNA-WDR59-2 | lincRNA | 282 |
| 0.9499479 | AV731436 | lincRNA-ANAPC4 | lincRNA | 666 |
| 0.9500478 | BE083440 | lincRNA-COL5A1-1 | lincRNA | 369 |
| 0.9501568 | AW661739 | lincRNA-NPAS2-2 | lincRNA | 421 |
| 0.9502251 | chr5:3737400-3749175+ | lincRNA-ADAMTS16-4 | lincRNA | 11776 |
| 0.9502295 | BX101700 | lincRNA-TACR1-1 | lincRNA | 720 |
| 0.9503946 | ENST00000426575 | RP11-318C24.2 | Ensembl | 657 |
| 0.9504896 | CX165522 | lincRNA-FAT4-1 | lincRNA | 827 |
| 0.9508309 | AK299287 | lincRNA-COLQ | lincRNA | 1840 |
| 0.9509041 | chr12:62007583-62044158+ | lincRNA-FAM19A2-4 | lincRNA | 36576 |
| 0.9512616 | chr2:168476665-168483470- | lincRNA-XIRP2-2 | lincRNA | 6806 |
| 0.9514799 | chr13:104506174-104518474+ | lincRNA-DAOA-4 | lincRNA | 12301 |
| 0.9519925 | chr15:56088155-56092624+ | lincRNA-NEDD4 | lincRNA | 4470 |
| 0.9521168 | chr4:178294426-178298806- | lincRNA-NEIL3 | lincRNA | 4381 |
| 0.9521807 | BX644753 | lincRNA-SFMBT1 | lincRNA | 796 |
| 0.9527725 | CR749802 | lincRNA-MBNL2-1 | lincRNA | 4571 |
| 0.9529331 | chr1:13902316-13904644+ | lincRNA-PDPN | lincRNA | 2329 |
| 0.9530076 | BE727398 | lincRNA-TCL1B | lincRNA | 971 |
| 0.9531451 | AK124249 |  | misc_RNA | 2763 |
| 0.9531546 | CV396150 | lincRNA-TXNRD1-1 | lincRNA | 296 |
| 0.9531781 | chrX:137339159-137368434- | lincRNA-ZIC3-3 | lincRNA | 29276 |
| 0.9532766 | ENST00000434601 | RP11-101P17.9 | Ensembl | 1179 |
| 0.95338 | chr18:47271277-47283077- | lincRNA-LIPG-2 | lincRNA | 11801 |
| 0.9534774 | uc003igi.1 | CR604304 | UCSC_knowngene | 1536 |
| 0.9537486 | DA267910 | lincRNA-ESM1-2 | lincRNA | 570 |
| 0.953831 | CV387322 | lincRNA-SEL1L-3 | lincRNA | 581 |
| 0.9540417 | NR_027139 | SPIN3 | RefSeq_NR | 1721 |
| 0.9540699 | uc010ikd.1 | BC005018 | UCSC_knowngene | 737 |
| 0.9540724 | CA307860 | lincRNA-USPL1-2 | lincRNA | 747 |
| 0.954561 | uc004fea.2 | LOC100272228 | UCSC_knowngene | 3348 |
| 0.9545809 | ENST00000460269 | MT-ND5 | Ensembl | 1703 |
| 0.9545966 | ENST00000425150 | RP11-348F1.3 | Ensembl | 451 |
| 0.9546358 | uc001wjc.2 | FLJ00089 | UCSC_knowngene | 2351 |
| 0.9547947 | chr1:212300402-212317702+ | lincRNA-PPP2R5A | lincRNA | 17301 |
| 0.955185 | chr9:81993205-81999034- | lincRNA-PSAT1-1 | lincRNA | 5830 |
| 0.9553733 | NR_002710 | ALOX12P2 | RefSeq_NR | 2768 |
| 0.9554527 | ENST00000394346 | RP11-512H23.2 | Ensembl | 511 |
| 0.9558871 | ENST00000485582 | MT-CO3 | Ensembl | 781 |
| 0.9561315 | CD298737 | lincRNA-ZEB2-7 | lincRNA | 842 |
| 0.9565486 | FN073667 | lincRNA-COX10-2 | lincRNA | 97 |
| 0.9567347 | NR_024607 | MGC16121 | RefSeq_NR | 786 |
| 0.9567492 | ENST00000441363 | RP11-398K22.12 | Ensembl | 2403 |
| 0.9571558 | AF279774 | lincRNA-GAP43 | lincRNA | 1205 |
| 0.957317 | chr6:22320996-22326999- | lincRNA-PRL | lincRNA | 6004 |
| 0.9573182 | ENST00000449526 | AC005042.5 | Ensembl | 510 |
| 0.9573805 | DB301608 | lincRNA-DUSP22 | lincRNA | 507 |
| 0.9574445 | chr1:18391088-18413388- | lincRNA-ACTL8 | lincRNA | 22301 |
| 0.9575306 | EB386294 | lincRNA-PLXDC2-1 | lincRNA | 795 |
| 0.9575378 | ENST00000440860 | RP11-75F3.1 | Ensembl | 376 |
| 0.9579553 | BM676747 | lincRNA-DCT | lincRNA | 348 |
| 0.9580026 | DB454079 | lincRNA-CSMD3-2 | lincRNA | 478 |
| 0.9582291 | chr15:48164774-48170708+ | lincRNA-SLC24A5 | lincRNA | 5935 |
| 0.9582964 | uc010wse.1 | KIAA1783 | UCSC_knowngene | 9651 |
| 0.9583026 | chr13:106073699-106106224- | lincRNA-SLC10A2-4 | lincRNA | 32526 |
| 0.9583812 | AF090886 |  | NRED | 2337 |
| 0.9584425 | chr11:128286813-128298538- | lincRNA-KIRREL3-3 | lincRNA | 11726 |
| 0.9587907 | NR_003930 | LOC387647 | RefSeq_NR | 2095 |
| 0.9588092 | AK294389 | lincRNA-RNF103 | lincRNA | 1135 |
| 0.9590761 | ENST00000430537 | AC007036.6 | Ensembl | 674 |
| 0.9591975 | BF950033 | lincRNA-ARID1A | lincRNA | 283 |
| 0.959317 | chr5:3565525-3579100+ | lincRNA-IRX1-1 | lincRNA | 13576 |
| 0.9595061 | ENST00000448265 | AC139618.1 | Ensembl | 2329 |
| 0.959605 | ENST00000414193 | RP1-164F3.8 | Ensembl | 469 |
| 0.9598477 | BF245637 | lincRNA-C17orf103 | lincRNA | 825 |
| 0.9599353 | ENST00000414888 | AC080125.1 | Ensembl | 684 |
| 0.9600801 | chr2:237543636-237554786- | lincRNA-CXCR7-1 | lincRNA | 11151 |
| 0.9605704 | AW963243 | lincRNA-H3F3A | lincRNA | 726 |
| 0.9608895 | ENST00000439075 | RP11-510H23.1 | Ensembl | 4507 |
| 0.9609458 | AJ431608 |  | misc_RNA | 349 |
| 0.9609583 | NR_028407 | ARMCX4 | RefSeq_NR | 5421 |
| 0.9610118 | ENST00000500803 | AL355344.1 | Ensembl | 1481 |
| 0.9611488 | NR_033358 | MEG3 | RefSeq_NR | 1735 |
| 0.9615421 | ENST00000433310 | AF131217.1 | Ensembl | 900 |
| 0.961556 | chr20:32034169-32054301+ | lincRNA-CBFA2T2 | lincRNA | 20133 |
| 0.9619443 | uc003klp.2 | FIS | UCSC_knowngene | 2049 |
| 0.9619716 | uc003qdd.2 | AF086187 | UCSC_knowngene | 990 |
| 0.9620941 | chr1:87222750-87234290+ | lincRNA-SEP15 | lincRNA | 11541 |
| 0.9622541 | DA731007 | lincRNA-ADCY2-1 | lincRNA | 667 |
| 0.9622778 | ENST00000427337 | RP11-445P19.1 | Ensembl | 424 |
| 0.9623077 | chrX:46050831-46067706- | lincRNA-CXorf36-7 | lincRNA | 16876 |
| 0.9623444 | chr4:35754355-35766755+ | lincRNA-ARAP2-2 | lincRNA | 12401 |
| 0.9624518 | BX105755 | lincRNA-KIAA1432-2 | lincRNA | 463 |
| 0.9628543 | CF128868 | lincRNA-DLL1-2 | lincRNA | 705 |
| 0.9629304 | chr1:30142313-30155538- | lincRNA-PTPRU | lincRNA | 13226 |
| 0.9630356 | AW072314 | lincRNA-OR2J2 | lincRNA | 371 |
| 0.9633155 | ENST00000481312 | CTD-2230D16.1 | Ensembl | 1970 |
| 0.9634159 | ENST00000506371 | AC010329.1 | Ensembl | 3048 |
| 0.9634804 | CR622249 | lincRNA-C1orf59 | lincRNA | 1585 |
| 0.9636821 | chr4:99107102-99123402+ | lincRNA-RAP1GDS1 | lincRNA | 16301 |
| 0.9639909 | chr15:56088155-56092624- | lincRNA-PRTG | lincRNA | 4470 |
| 0.9643267 | uc003wqb.1 | AK128880 | UCSC_knowngene | 577 |
| 0.9646626 | NR_024480 | LOC100131551 | RefSeq_NR | 3021 |
| 0.9647221 | chr2:238194611-238205061- | lincRNA-COPS8-2 | lincRNA | 10451 |
| 0.9647369 | CN284611 | lincRNA-CUX2 | lincRNA | 731 |
| 0.9647394 | chr5:73882471-73909894- | lincRNA-RGNEF-4 | lincRNA | 27424 |
| 0.9647556 | BU632228 | lincRNA-ERCC4 | lincRNA | 582 |
| 0.9650198 | chr14:66260097-66320947- | lincRNA-FUT8-1 | lincRNA | 60851 |
| 0.9652822 | NR_033182 | LOC100130872-SPON2 | RefSeq_NR | 729 |
| 0.9656979 | BX537809 |  | misc_RNA | 1733 |
| 0.965863 | ENST00000512915 | RP11-499E18.1 | Ensembl | 546 |
| 0.9658858 | ENST00000426284 | AC096772.4 | Ensembl | 444 |
| 0.9659924 | ENST00000422833 | RP1-213J1P__B.2 | Ensembl | 115 |
| 0.9660823 | chr6:3775751-3789451- | lincRNA-C6orf145-2 | lincRNA | 13701 |
| 0.9664887 | uc001dnu.1 | AK022898 | UCSC_knowngene | 2786 |
| 0.9667572 | ENST00000505360 | Z83745.1 | Ensembl | 2234 |
| 0.9668238 | chr5:117728562-117735189+ | lincRNA-DTWD2-3 | lincRNA | 6628 |
| 0.9670218 | ENST00000340424 | RP4-673D20.1 | Ensembl | 1216 |
| 0.967112 | AK094311 |  | misc_RNA | 2618 |
| 0.9671523 | DW444236 | lincRNA-ZNF703-2 | lincRNA | 227 |
| 0.9672457 | BX104557 | lincRNA-SHQ1-3 | lincRNA | 526 |
| 0.9674061 | AK124321 | lincRNA-RNF32-3 | lincRNA | 2538 |
| 0.9674944 | ENST00000426710 | AC013262.1 | Ensembl | 252 |
| 0.967715 | AK026322 |  | misc_RNA | 1284 |
| 0.9680331 | chr11:87230202-87240977- | lincRNA-TMEM135 | lincRNA | 10776 |
| 0.9682194 | ENST00000404155 | RP1-40E16.8 | Ensembl | 871 |
| 0.9691941 | chr9:137160729-137173954- | lincRNA-WDR5-2 | lincRNA | 13226 |
| 0.9693808 | ENST00000434426 | RP11-73B2.6 | Ensembl | 744 |
| 0.970135 | ENST00000455592 | RP11-165F24.2 | Ensembl | 494 |
| 0.9702709 | exon100+ | lincRNA-CDCP2 | lincRNA | 251 |
| 0.9705728 | ENST00000449818 | RP11-40H20.2 | Ensembl | 867 |
| 0.9705908 | BY797754 | lincRNA-ZNF469-2 | lincRNA | 538 |
| 0.9706388 | BG925695 | lincRNA-CASK-2 | lincRNA | 461 |
| 0.9711595 | chr1:232506752-232522177+ | lincRNA-SIPA1L2-2 | lincRNA | 15426 |
| 0.9713973 | uc003mto.2 | BC087858 | UCSC_knowngene | 1322 |
| 0.971596 | chr8:48104990-48105990+ | lincRNA-KIAA0146-2 | lincRNA | 1001 |
| 0.9717302 | chr2:237506710-237517672+ | lincRNA-COPS8-4 | lincRNA | 10963 |
| 0.9719766 | ENST00000399997 | RP11-431J24.4 | Ensembl | 855 |
| 0.9721592 | U93033 | lincRNA-WISP1 | lincRNA | 8427 |
| 0.9722519 | uc003ugj.1 | BC037783 | UCSC_knowngene | 2113 |
| 0.9724833 | uc001lkg.1 | AK124226 | UCSC_knowngene | 3086 |
| 0.9725749 | AK022462 |  | misc_RNA | 1784 |
| 0.9726856 | uc001ldf.2 | EMX2 | UCSC_knowngene | 7024 |
| 0.9727769 | DT216792 | lincRNA-SORL1-3 | lincRNA | 716 |
| 0.9730737 | ENST00000451822 | AP002959.1 | Ensembl | 1071 |
| 0.9732491 | DA569730 | lincRNA-TET2-2 | lincRNA | 869 |
| 0.9746872 | BQ632011 | lincRNA-ERI1-3 | lincRNA | 483 |
| 0.9748254 | exon2236- | lincRNA-INHBB-4 | lincRNA | 466 |
| 0.9751264 | chr3:118559485-118571910+ | lincRNA-IGSF11-4 | lincRNA | 12426 |
| 0.9751497 | AK125021 |  | misc_RNA | 4140 |
| 0.975187 | chr2:68560571-68583471- | lincRNA-CNRIP1 | lincRNA | 22901 |
| 0.9753525 | chr10:44737519-44749219- | lincRNA-ZNF32-1 | lincRNA | 11701 |
| 0.9755573 | BQ222987 | lincRNA-SLC16A7-7 | lincRNA | 921 |
| 0.9758599 | chr1:157945191-157956934- | lincRNA-CD5L | lincRNA | 11744 |
| 0.9762275 | chr11:2448349-2458849- | lincRNA-TRPM5 | lincRNA | 10501 |
| 0.9766132 | chr9:26772419-26808763- | lincRNA-TUSC1 | lincRNA | 36345 |
| 0.9768067 | chr3:140465232-140468555+ | lincRNA-SLC25A36-3 | lincRNA | 3324 |
| 0.977523 | AW063279 | lincRNA-GALNTL4 | lincRNA | 445 |
| 0.9781718 | AI239901 | lincRNA-CD82-1 | lincRNA | 229 |
| 0.9782051 | AK299216 | lincRNA-SKAP2-2 | lincRNA | 1360 |
| 0.9783286 | uc001jab.3 | ZNF37B | UCSC_knowngene | 10237 |
| 0.9785899 | AK311417 | lincRNA-ATPBD4-2 | lincRNA | 1074 |
| 0.9791481 | DB337334 | lincRNA-MLYCD | lincRNA | 535 |
| 0.9791729 | CD357617 | lincRNA-RND3-5 | lincRNA | 749 |
| 0.9793982 | AK126099 |  | misc_RNA | 2985 |
| 0.9794195 | BC150253 | lincRNA-SEMA6D-3 | lincRNA | 5955 |
| 0.9794412 | DB068418 | lincRNA-SERP2-4 | lincRNA | 534 |
| 0.9794717 | R08380 | lincRNA-HSD17B2 | lincRNA | 332 |
| 0.9795743 | ENST00000505974 | AC025261.1 | Ensembl | 2685 |
| 0.9795922 | NR_002612 | DLEU2 | RefSeq_NR | 2768 |
| 0.9800387 | exon3669- | lincRNA-FRMD1-3 | lincRNA | 345 |
| 0.9803586 | CN288742 | lincRNA-MCPH1-2 | lincRNA | 683 |
| 0.9806088 | uc010bfv.1 | AK123700 | UCSC_knowngene | 1913 |
| 0.9808449 | ENST00000458683 | RP11-108M21.1 | Ensembl | 858 |
| 0.9810817 | AI379096 | lincRNA-KCNU1-1 | lincRNA | 488 |
| 0.9812685 | chr3:194505234-194523815+ | lincRNA-C3orf21-1 | lincRNA | 18582 |
| 0.9813276 | chr4:79623626-79634676- | lincRNA-ANXA3-2 | lincRNA | 11051 |
| 0.9815616 | uc002fcp.1 | BC043527 | UCSC_knowngene | 966 |
| 0.9821134 | chr5:137976251-138005451- | lincRNA-HSPA9 | lincRNA | 29201 |
| 0.9828483 | BC036602 |  | RNAdb | 1336 |
| 0.9829153 | ENST00000504402 | RP11-367J11.3 | Ensembl | 292 |
| 0.9830115 | HIT000086764 | | H-invDB | 724 |
| 0.9831374 | chr4:15911552-15921927- | lincRNA-CD38 | lincRNA | 10376 |
| 0.9833512 | ENST00000492365 | KB-1683C8.1 | Ensembl | 877 |
| 0.9834278 | AW976885 | lincRNA-FAM113B-3 | lincRNA | 664 |
| 0.9835128 | uc001yxn.3 | SNURF-SNRPN | UCSC_knowngene | 7846 |
| 0.983616 | ENST00000501400 | AC011118.2 | Ensembl | 1914 |
| 0.9837388 | DB078790 | lincRNA-EBF2-1 | lincRNA | 568 |
| 0.9839656 | chr8:135201218-135216768- | lincRNA-ST3GAL1-2 | lincRNA | 15551 |
| 0.9840257 | ENST00000398460 | AL117190.1 | Ensembl | 3381 |
| 0.9844076 | chr4:12970502-12999702- | lincRNA-HS3ST1-8 | lincRNA | 29201 |
| 0.9844259 | NR_015439 | LOC550112 | RefSeq_NR | 2301 |
| 0.984434 | ENST00000503577 | RP11-113I22.1 | Ensembl | 517 |
| 0.9844609 | BE010595 | lincRNA-NPVF-4 | lincRNA | 374 |
| 0.9847097 | AI828001 | lincRNA-SHQ1-4 | lincRNA | 349 |
| 0.9847681 | chr12:18978108-19004533- | lincRNA-CAPZA3 | lincRNA | 26426 |
| 0.9852751 | AA777491 | lincRNA-C9 | lincRNA | 568 |
| 0.9855199 | ENST00000502331 | OR7E94P | Ensembl | 364 |
| 0.9860122 | AK098597 |  | misc_RNA | 1683 |
| 0.9864948 | ENST00000429825 | AC013717.3 | Ensembl | 488 |
| 0.9865767 | DB317923 | lincRNA-MED13L-4 | lincRNA | 493 |
| 0.9866609 | chr18:46500588-46515984+ | lincRNA-DYM-1 | lincRNA | 15397 |
| 0.9869965 | AI201851 | lincRNA-NFE2L3-3 | lincRNA | 347 |
| 0.9873812 | ENST00000396599 | AC005062.3 | Ensembl | 483 |
| 0.9873984 | uc001dps.2 | CR609342 | UCSC_knowngene | 1003 |
| 0.9874697 | AA219306 | lincRNA-CLIC5-2 | lincRNA | 428 |
| 0.9875464 | DA098543 | lincRNA-EN2-1 | lincRNA | 568 |
| 0.9876819 | AK307796 | lincRNA-ARHGAP18-2 | lincRNA | 1320 |
| 0.9877539 | DA317262 | lincRNA-POLR1A | lincRNA | 529 |
| 0.9882098 | chr5:126055611-126069263+ | lincRNA-LMNB1-1 | lincRNA | 13653 |
| 0.9885389 | chr1:100774937-100789212+ | lincRNA-CDC14A | lincRNA | 14276 |
| 0.9886447 | AW469566 | lincRNA-C11orf74-3 | lincRNA | 360 |
| 0.9888338 | DA731060 | lincRNA-DISP1 | lincRNA | 780 |
| 0.9896763 | ENST00000488511 | RP11-7F17.1 | Ensembl | 344 |
| 0.9896822 | exon92- | lincRNA-DMBX1 | lincRNA | 197 |
| 0.9898221 | CB219568 | lincRNA-TBX3-1 | lincRNA | 88 |
| 0.9900238 | NR_027481 | ZNF876P | RefSeq_NR | 2621 |
| 0.9902784 | chr10:55003944-55021494+ | lincRNA-PCDH15-1 | lincRNA | 17551 |
| 0.9903002 | chrX:39547291-39551460+ | lincRNA-BCOR-10 | lincRNA | 4170 |
| 0.990518 | AA496137 | lincRNA-GJD2-2 | lincRNA | 460 |
| 0.9906479 | AK091713 |  | NRED | 1932 |
| 0.9907713 | chr4:158942228-158975298+ | lincRNA-FAM198B-2 | lincRNA | 33071 |
| 0.9908464 | ENST00000499522 | AC025370.1 | Ensembl | 1753 |
| 0.9908765 | ENST00000504219 | RP11-617D20.1 | Ensembl | 627 |
| 0.9912261 | ENST00000452402 | RP11-250B2.3 | Ensembl | 682 |
| 0.9912282 | BF818610 | lincRNA-PMEPA1-2 | lincRNA | 479 |
| 0.9912444 | ENST00000505438 | AC004069.1 | Ensembl | 1113 |
| 0.9912557 | ENST00000392478 | AC015815.2 | Ensembl | 123 |
| 0.9916597 | NR_002766 | MEG3 | RefSeq_NR | 1595 |
| 0.9918989 | chr6:155661283-155682333- | lincRNA-TFB1M | lincRNA | 21051 |
| 0.9920289 | AA432215 | lincRNA-GXYLT1 | lincRNA | 352 |
| 0.9920588 | NR_027252 | C2orf58 | RefSeq_NR | 1776 |
| 0.9924328 | uc002vga.1 | AX747067 | UCSC_knowngene | 2348 |
| 0.9924557 | AK057475 |  | misc_RNA | 2087 |
| 0.9926175 | chr5:67675269-67719019- | lincRNA-PIK3R1-3 | lincRNA | 43751 |
| 0.992818 | ENST00000510652 | AL358933.1 | Ensembl | 1517 |
| 0.9929852 | chr2:88954435-88968860- | lincRNA-EIF2AK3 | lincRNA | 14426 |
| 0.9929949 | chr8:37801630-37818621- | lincRNA-GOT1L1 | lincRNA | 16992 |
| 0.9930279 | ENST00000412512 | RP5-1003J2.4 | Ensembl | 221 |
| 0.9931873 | chr1:116797827-116838952- | lincRNA-C1orf161 | lincRNA | 41126 |
| 0.9933743 | chr7:143147778-143169303- | lincRNA-TAS2R60 | lincRNA | 21526 |
| 0.9937708 | uc003ttc.1 | AK125429 | UCSC_knowngene | 4369 |
| 0.9938221 | NR_015364 | LOC441204 | RefSeq_NR | 1649 |
| 0.9938415 | chrX:39589029-39596014- | lincRNA-MID1IP1-1 | lincRNA | 6986 |
| 0.9938977 | BF511988 | lincRNA-TOMM20-1 | lincRNA | 521 |
| 0.994067 | ENST00000510784 | AL160400.1 | Ensembl | 1029 |
| 0.9941005 | BE669623 | lincRNA-CCDC85A-1 | lincRNA | 540 |
| 0.9942957 | chr9:111547504-111559779- | lincRNA-KLF4-4 | lincRNA | 12276 |
| 0.9944786 | chr6:47317091-47336991- | lincRNA-TNFRSF21-1 | lincRNA | 19901 |
| 0.9945019 | chr5:158080172-158099447- | lincRNA-CLINT1 | lincRNA | 19276 |
| 0.9945027 | chr6:91127779-91150654- | lincRNA-BACH2 | lincRNA | 22876 |
| 0.9947844 | chr15:74254943-74258662+ | lincRNA-STOML1 | lincRNA | 3720 |
| 0.9950119 | BF897862 | lincRNA-RPS14-1 | lincRNA | 554 |
| 0.9965115 | chr4:27048002-27104452+ | lincRNA-PCDH7-2 | lincRNA | 56451 |
| 0.9968077 | DB131700 | lincRNA-PSD3-2 | lincRNA | 554 |
| 0.9969341 | uc010tgn.1 | XTP6 | UCSC_knowngene | 1348 |
| 0.9971295 | chr2:121182275-121194039+ | lincRNA-GLI2-3 | lincRNA | 11765 |
| 0.9972003 | BM678241 | lincRNA-SOX9 | lincRNA | 707 |
| 0.9977263 | ENST00000458202 | RP11-41L14.1 | Ensembl | 1648 |
| 0.9978642 | chr13:42065250-42122000- | lincRNA-C13orf15-1 | lincRNA | 56751 |
| 0.9978833 | AK054728 | lincRNA-CALCA | lincRNA | 2258 |
| 0.9981234 | ENST00000509834 | RP11-420A23.1 | Ensembl | 747 |
| 0.9981287 | ENST00000309884 | RP4-575N6.3 | Ensembl | 492 |
| 0.998251 | chr7:140344106-140355056+ | lincRNA-ADCK2 | lincRNA | 10951 |
| 0.9982712 | EF363096 | lincRNA-AMIGO1 | lincRNA | 672 |
| 0.998502 | ENST00000447972 | AC007163.6 | Ensembl | 841 |
| 0.9989404 | chr15:74197716-74198633+ | lincRNA-LOXL1-2 | lincRNA | 918 |
| 0.9992212 | ENST00000441399 | RP5-842K24.2 | Ensembl | 3474 |
| 0.9992534 | exon1962- | lincRNA-CTAGE1-2 | lincRNA | 113 |
| 0.999498 | ENST00000457638 | AC093106.4 | Ensembl | 1053 |
| 0.9995433 | ENST00000435559 | RP11-86H7.7 | Ensembl | 2272 |
| 0.999872 | ENST00000452532 | RP13-188A5.1 | Ensembl | 1007 |
| 0.9999767 | ENST00000426835 | AC004945.2 | Ensembl | 738 |
| 1.0000965 | AK098701 |  | misc_RNA | 1416 |
| 1.0001871 | AL832093 |  | RNAdb | 3338 |
| 1.0005918 | AF319524 |  | RNAdb | 2253 |
| 1.0006835 | chr7:136275537-136279697+ | lincRNA-CHRM2-2 | lincRNA | 4161 |
| 1.0007077 | chr10:21548369-21563069+ | lincRNA-C10orf114 | lincRNA | 14701 |
| 1.0007196 | ENST00000420406 | RP4-740C4.4 | Ensembl | 371 |
| 1.0007205 | BG003184 | lincRNA-BLID-2 | lincRNA | 408 |
| 1.001102 | ENST00000440556 | RP11-513G11.2 | Ensembl | 1771 |
| 1.0012603 | ENST00000441036 | AC104782.3 | Ensembl | 581 |
| 1.0014087 | AK128052 |  | misc_RNA | 3587 |
| 1.0014587 | chr8:90889605-90908427- | lincRNA-RIPK2-3 | lincRNA | 18823 |
| 1.0016815 | chr3:65301806-65320211+ | lincRNA-MAGI1-6 | lincRNA | 18406 |
| 1.00216 | AF338234 | lincRNA-FBXO33-2 | lincRNA | 4110 |
| 1.0026013 | uc001wxl.2 | BC084561 | UCSC_knowngene | 2301 |
| 1.0026054 | NR_027106 | LOC285419 | RefSeq_NR | 2222 |
| 1.0028613 | uc003qje.1 | AK057490 | UCSC_knowngene | 1900 |
| 1.0033692 | uc003qup.1 | AK090688 | UCSC_knowngene | 2044 |
| 1.0035318 | chr3:25863830-25873380+ | lincRNA-LRRC3B | lincRNA | 9551 |
| 1.0040245 | BC030956 | lincRNA-TFPI2-2 | lincRNA | 1970 |
| 1.0042956 | ENST00000425356 | RP11-54K16.2 | Ensembl | 484 |
| 1.0044427 | ENST00000434309 | RP11-91K9.1 | Ensembl | 581 |
| 1.004465 | ENST00000452777 | RP11-318C24.1 | Ensembl | 171 |
| 1.0048278 | chr1:39116163-39128263+ | lincRNA-RRAGC-1 | lincRNA | 12101 |
| 1.0048305 | AK090797 |  | misc_RNA | 2827 |
| 1.0051314 | chr13:73862624-73868384+ | lincRNA-KLF12-2 | lincRNA | 5761 |
| 1.0051314 | uc001stu.2 | BC035381 | UCSC_knowngene | 3099 |
| 1.0052802 | AI149906 | lincRNA-NEDD1-7 | lincRNA | 480 |
| 1.0053487 | exon1959- | lincRNA-CTAGE1-2 | lincRNA | 93 |
| 1.0054924 | BM466146 | lincRNA-ZNF396 | lincRNA | 793 |
| 1.0056526 | chr1:22577463-22595288+ | lincRNA-ZBTB40 | lincRNA | 17826 |
| 1.0057834 | ENST00000429281 | RP11-265P11.2 | Ensembl | 673 |
| 1.0062655 | ENST00000426509 | RP11-442J21.1 | Ensembl | 295 |
| 1.006337 | BX477169 | lincRNA-TMEM200A-2 | lincRNA | 470 |
| 1.006686 | uc010lfm.1 | AK310300 | UCSC_knowngene | 1228 |
| 1.0067809 | chr1:88059958-88068318+ | lincRNA-PKN2-2 | lincRNA | 8361 |
| 1.0068796 | ENST00000445083 | AC019100.3 | Ensembl | 611 |
| 1.0072552 | ENST00000364713 | AL591506.1 | Ensembl | 306 |
| 1.0074141 | chr8:20578220-20590170+ | lincRNA-GFRA2-1 | lincRNA | 11951 |
| 1.0074618 | CN479707 | lincRNA-RBFOX2 | lincRNA | 678 |
| 1.0076391 | ENST00000441630 | AC008074.3 | Ensembl | 723 |
| 1.0077309 | AK095608 |  | NRED | 2784 |
| 1.0080232 | NR_003531 | MEG3 | RefSeq_NR | 1735 |
| 1.0080809 | ENST00000466108 | MT-ND4 | Ensembl | 1367 |
| 1.0084899 | ENST00000422732 | RP11-314P12.2 | Ensembl | 2444 |
| 1.0087207 | ENST00000499326 | AC022087.2 | Ensembl | 717 |
| 1.0090723 | ENST00000442418 | RP11-86H7.1 | Ensembl | 748 |
| 1.0095493 | uc001zyi.2 | BX537481 | UCSC_knowngene | 2284 |
| 1.0098011 | ENST00000426688 | RP3-445O10.1 | Ensembl | 643 |
| 1.0098053 | chr18:68717578-68723320+ | lincRNA-CBLN2-3 | lincRNA | 5743 |
| 1.0098731 | AV722915 | lincRNA-PTGS2-2 | lincRNA | 669 |
| 1.0098732 | BC064349 |  | RNAdb | 1448 |
| 1.010062 | BC038545 | lincRNA-C11orf30 | lincRNA | 444 |
| 1.0102822 | DA530362 | lincRNA-PROX1-3 | lincRNA | 608 |
| 1.0105139 | chrX:55801375-55812875- | lincRNA-RRAGB-1 | lincRNA | 11501 |
| 1.010522 | AI218855 | lincRNA-RPS14-2 | lincRNA | 269 |
| 1.0107363 | DB482133 | lincRNA-LUZP6-3 | lincRNA | 482 |
| 1.0107668 | uc004ebo.2 | LOC554203 | UCSC_knowngene | 2212 |
| 1.0108037 | chr8:23609030-23619205- | lincRNA-NKX2-6 | lincRNA | 10176 |
| 1.0110573 | chr3:136790035-136808485- | lincRNA-IL20RB-1 | lincRNA | 18451 |
| 1.0116548 | chr1:82115673-82123396- | lincRNA-ELTD1-1 | lincRNA | 7724 |
| 1.011683 | BM982048 | lincRNA-STRA6 | lincRNA | 667 |
| 1.0117216 | BC036623 |  | misc_RNA | 1315 |
| 1.0118459 | chr13:95301199-95328649- | lincRNA-GPR180 | lincRNA | 27451 |
| 1.0122263 | BM728564 | lincRNA-HHAT | lincRNA | 568 |
| 1.0125237 | chr12:89710086-89721197+ | lincRNA-DUSP6-1 | lincRNA | 11112 |
| 1.0128858 | uc003jbv.1 | BC032469 | UCSC_knowngene | 1757 |
| 1.0131196 | NR_015395 | LOC541471 | RefSeq_NR | 809 |
| 1.0132825 | BM680861 | lincRNA-ADAM7 | lincRNA | 494 |
| 1.0136218 | chr6:130910607-130926232- | lincRNA-TMEM200A-3 | lincRNA | 15626 |
| 1.0138316 | BX116989 | lincRNA-RYBP-4 | lincRNA | 467 |
| 1.0138512 | ENST00000438350 | RP11-40H20.3 | Ensembl | 221 |
| 1.0143131 | AA595877 | lincRNA-C16orf72-1 | lincRNA | 169 |
| 1.0146901 | chr8:65998046-66008971- | lincRNA-CYP7B1-2 | lincRNA | 10926 |
| 1.0148688 | uc001vca.2 | BC039553 | UCSC_knowngene | 1141 |
| 1.0149451 | DA204380 | lincRNA-C6orf118-6 | lincRNA | 592 |
| 1.015034 | ENST00000433747 | RP11-120D5.1 | Ensembl | 611 |
| 1.0150641 | DA538556 | lincRNA-C14orf180-1 | lincRNA | 570 |
| 1.0150727 | exon1961- | lincRNA-CTAGE1-2 | lincRNA | 1130 |
| 1.0152 | AW242585 | lincRNA-BUD13-1 | lincRNA | 367 |
| 1.0152975 | ENST00000448432 | RP11-453O22.1 | Ensembl | 357 |
| 1.0154983 | chr1:181081092-181121244+ | lincRNA-CACNA1E-3 | lincRNA | 40153 |
| 1.0156057 | AA564591 | lincRNA-PPYR1-2 | lincRNA | 385 |
| 1.0156113 | DB224754 | lincRNA-RRAGC-4 | lincRNA | 566 |
| 1.0158043 | DB327252 | lincRNA-PBX1-3 | lincRNA | 544 |
| 1.0158694 | AA420793 | lincRNA-RAP2B-1 | lincRNA | 359 |
| 1.0162555 | BM682695 | lincRNA-ZNF92-3 | lincRNA | 234 |
| 1.0162658 | DB027866 | lincRNA-SDPR-2 | lincRNA | 595 |
| 1.0164305 | ENST00000432783 | RP11-569G13.2 | Ensembl | 1577 |
| 1.0164512 | chr12:77754619-77760162- | lincRNA-E2F7 | lincRNA | 5544 |
| 1.0165481 | DN604327 | lincRNA-CDH11-1 | lincRNA | 160 |
| 1.0166444 | CR608597 | lincRNA-ZNF117 | lincRNA | 1837 |
| 1.0167921 | BX093893 | lincRNA-GLIPR1 | lincRNA | 464 |
| 1.0168656 | ENST00000442687 | AC034228.7 | Ensembl | 1578 |
| 1.0170275 | uc003kzj.2 | BC032795 | UCSC_knowngene | 1045 |
| 1.0170445 | BC036853 | lincRNA-PDCD1LG2-2 | lincRNA | 1660 |
| 1.0172821 | uc002cuc.2 | BC013599 | UCSC_knowngene | 1114 |
| 1.017365 | BG896515 | lincRNA-MAP3K9-2 | lincRNA | 419 |
| 1.0176932 | ENST00000400430 | RP11-350A18.1 | Ensembl | 702 |
| 1.0181731 | AW269809 | lincRNA-KPNA1 | lincRNA | 382 |
| 1.0188715 | exon2995- | lincRNA-TMEM174-2 | lincRNA | 180 |
| 1.0189608 | uc001cil.2 | CR621467 | UCSC_knowngene | 1030 |
| 1.01913 | NR_027022 | LOC127841 | RefSeq_NR | 1290 |
| 1.0196131 | uc002ivh.1 | AK126318 | UCSC_knowngene | 4722 |
| 1.0197661 | DB351988 | lincRNA-RPS24-2 | lincRNA | 541 |
| 1.0200799 | chr12:38784383-38812908- | lincRNA-ALG10B-1 | lincRNA | 28526 |
| 1.0202058 | DB525109 | lincRNA-CENPW-1 | lincRNA | 483 |
| 1.0202717 | AI638100 | lincRNA-ADCYAP1 | lincRNA | 379 |
| 1.0204375 | AW275694 | lincRNA-EIF3H-3 | lincRNA | 408 |
| 1.0205881 | ENST00000512710 | AL359643.3 | Ensembl | 3273 |
| 1.021016 | ENST00000502919 | CTC-236F12.2 | Ensembl | 473 |
| 1.0210724 | chr6:125843301-125854226+ | lincRNA-HEY2-1 | lincRNA | 10926 |
| 1.0212777 | chr6:89272256-89285156+ | lincRNA-RNGTT-1 | lincRNA | 12901 |
| 1.0221045 | uc002tyw.2 | BC032407 | UCSC_knowngene | 1745 |
| 1.0222625 | HIT000395606 | | H-invDB | 386 |
| 1.0228027 | AL133825 | lincRNA-KCND3-1 | lincRNA | 817 |
| 1.0228891 | ENST00000439558 | RP5-907D15.2 | Ensembl | 648 |
| 1.0229711 | ENST00000504349 | RP11-155L15.1 | Ensembl | 1227 |
| 1.0230093 | ENST00000464531 | RP11-171G7.1 | Ensembl | 1192 |
| 1.023114 | CV315884 | lincRNA-ZNF652 | lincRNA | 244 |
| 1.0232266 | ENST00000503950 | RP11-381N20.2 | Ensembl | 539 |
| 1.0233574 | uc003yry.2 | BX648371 | UCSC_knowngene | 5325 |
| 1.0236737 | BC031261 | lincRNA-ZPLD1-2 | lincRNA | 2203 |
| 1.0237312 | NR_003038 | SNHG5 | RefSeq_NR | 524 |
| 1.0239016 | CU444927 | lincRNA-MYEOV-2 | lincRNA | 742 |
| 1.0244443 | ENST00000514243 | RP11-159K7.1 | Ensembl | 533 |
| 1.0245265 | AK123174 |  | misc_RNA | 1951 |
| 1.0246307 | ENST00000510011 | RP11-33B1.1 | Ensembl | 979 |
| 1.0246747 | T57623 | lincRNA-KLF12-8 | lincRNA | 472 |
| 1.0252763 | uc003pqc.2 | BC047952 | UCSC_knowngene | 879 |
| 1.0254027 | chr4:53681743-53706918+ | lincRNA-RASL11B | lincRNA | 25176 |
| 1.0258991 | ENST00000428029 | AC084018.1 | Ensembl | 895 |
| 1.0259959 | ENST00000452377 | RP11-399H11.3 | Ensembl | 527 |
| 1.0262408 | BC043278 |  | misc_RNA | 1616 |
| 1.0264745 | ENST00000454681 | RP11-141M1.3 | Ensembl | 844 |
| 1.0266617 | DB340248 | lincRNA-KCNK9-1 | lincRNA | 555 |
| 1.0267152 | ENST00000415381 | RP11-301M17.1 | Ensembl | 517 |
| 1.0268651 | chr15:64339447-64344761+ | lincRNA-FAM96A | lincRNA | 5315 |
| 1.0269295 | DA829587 | lincRNA-ZNF516-2 | lincRNA | 815 |
| 1.0270032 | ENST00000510743 | AP000998.1 | Ensembl | 2633 |
| 1.0272076 | ENST00000416112 | AP001793.1 | Ensembl | 1595 |
| 1.0282204 | ENST00000506070 | AC008592.7 | Ensembl | 571 |
| 1.0286863 | AI760638 | lincRNA-PTH2R-1 | lincRNA | 500 |
| 1.0287092 | ENST00000444770 | RP11-170M17.1 | Ensembl | 794 |
| 1.0287862 | ENST00000419829 | RP11-465C5.1 | Ensembl | 959 |
| 1.028837 | BX957058 | lincRNA-OR9Q1 | lincRNA | 692 |
| 1.0293065 | chr5:1777673-1784347- | lincRNA-LPCAT1-1 | lincRNA | 6675 |
| 1.0294209 | BC033975 |  | misc_RNA | 1120 |
| 1.029474 | chr21:21942829-22002654+ | lincRNA-NCAM2-6 | lincRNA | 59826 |
| 1.0299691 | chr8:38509143-38523468+ | lincRNA-TACC1 | lincRNA | 14326 |
| 1.0302272 | DB305613 | lincRNA-AIG1-2 | lincRNA | 534 |
| 1.0308687 | AA451649 | lincRNA-ZNF572-2 | lincRNA | 438 |
| 1.0313777 | AA167479 | lincRNA-TMCC2 | lincRNA | 552 |
| 1.0316905 | BQ446739 | lincRNA-NPPC | lincRNA | 522 |
| 1.0318374 | chr2:72079817-72089167+ | lincRNA-CYP26B1 | lincRNA | 9351 |
| 1.0318817 | chr2:192408439-192420559+ | lincRNA-OBFC2A-1 | lincRNA | 12121 |
| 1.0319376 | AA398121 | lincRNA-VCAM1 | lincRNA | 338 |
| 1.0322036 | BM699739 | lincRNA-FHIT-1 | lincRNA | 246 |
| 1.0325465 | ENST00000451325 | RP11-445P17.5 | Ensembl | 435 |
| 1.0325714 | ENST00000443621 | RP11-30C8.2 | Ensembl | 282 |
| 1.0330492 | BX110722 | lincRNA-SGK223-2 | lincRNA | 397 |
| 1.0335993 | ENST00000510677 | AL360215.1 | Ensembl | 3298 |
| 1.0336677 | AK092616 |  | misc_RNA | 1806 |
| 1.0337456 | ENST00000417487 | AC104297.2 | Ensembl | 715 |
| 1.0341781 | BQ447228 | lincRNA-ALG10-1 | lincRNA | 403 |
| 1.0342809 | chr4:147979327-148023300+ | lincRNA-EDNRA-1 | lincRNA | 43974 |
| 1.0344312 | BX508282 | lincRNA-KCNMA1-2 | lincRNA | 808 |
| 1.0348982 | AK097799 |  | misc_RNA | 1756 |
| 1.0359226 | chr9:87653896-87666946- | lincRNA-NTRK2 | lincRNA | 13051 |
| 1.036158 | uc010wkl.1 | DQ589083 | UCSC_knowngene | 1661 |
| 1.0363302 | CX787170 | lincRNA-MAD2L1-2 | lincRNA | 674 |
| 1.036366 | ENST00000435407 | AC024704.2 | Ensembl | 416 |
| 1.0367764 | uc003pgq.2 | AL832252 | UCSC_knowngene | 3358 |
| 1.0370193 | ENST00000442666 | AC005042.4 | Ensembl | 825 |
| 1.037174 | chr2:119422342-119427395- | lincRNA-INSIG2-4 | lincRNA | 5054 |
| 1.0373405 | DB303228 | lincRNA-MYSM1-3 | lincRNA | 551 |
| 1.0373908 | ENST00000412571 | RP1-122P22.2 | Ensembl | 415 |
| 1.0374556 | ENST00000503268 | CTC-340D7.1 | Ensembl | 499 |
| 1.0381792 | AK125572 |  | RNAdb | 3355 |
| 1.0384205 | BG926534 | lincRNA-SLC39A10-1 | lincRNA | 617 |
| 1.0384859 | uc003hlr.1 | BC036848 | UCSC_knowngene | 756 |
| 1.0389628 | uc003kwu.1 | BX538252 | UCSC_knowngene | 916 |
| 1.0390431 | BX100522 | lincRNA-TET2-3 | lincRNA | 467 |
| 1.0394068 | EC575964 | lincRNA-TMTC1 | lincRNA | 106 |
| 1.039506 | BU533451 | lincRNA-ATP6V1C1-2 | lincRNA | 834 |
| 1.0397055 | ENST00000399501 | RP11-292F22.3 | Ensembl | 1273 |
| 1.0399083 | ENST00000427762 | RP4-592A1.2 | Ensembl | 690 |
| 1.0402894 | uc003nih.2 | BC033330 | UCSC_knowngene | 2883 |
| 1.0405022 | AK093407 | lincRNA-FAM84B-5 | lincRNA | 1671 |
| 1.040759 | AW129555 | lincRNA-DEPTOR-2 | lincRNA | 380 |
| 1.0410493 | AI247420 | lincRNA-RPS6KC1-1 | lincRNA | 408 |
| 1.0412152 | ENST00000306634 | RP11-220D10.1 | Ensembl | 318 |
| 1.0418945 | chr3:39278546-39291596+ | lincRNA-CX3CR1 | lincRNA | 13051 |
| 1.0426468 | CA391721 | lincRNA-EFNA5-1 | lincRNA | 514 |
| 1.0426539 | DN920247 | lincRNA-SCARB2 | lincRNA | 527 |
| 1.0429194 | AY927632 |  | RNAdb | 1164 |
| 1.0432729 | ENST00000455973 | RP11-359N11.1 | Ensembl | 470 |
| 1.043386 | chr6:140889232-140902332- | lincRNA-CITED2-2 | lincRNA | 13101 |
| 1.0433997 | ENST00000479610 | RP11-59J16.2 | Ensembl | 697 |
| 1.043454 | DA245301 | lincRNA-C6orf118-3 | lincRNA | 584 |
| 1.0441044 | BX119188 | lincRNA-NRG3-1 | lincRNA | 308 |
| 1.0443702 | uc003dqo.2 | BC050344 | UCSC_knowngene | 4299 |
| 1.0445822 | AK122979 |  | misc_RNA | 2107 |
| 1.0445856 | chr8:140508518-140524643+ | lincRNA-KCNK9-3 | lincRNA | 16126 |
| 1.0449621 | W87727 | lincRNA-LTA4H | lincRNA | 280 |
| 1.0452829 | chr12:96851553-96877162- | lincRNA-CDK17-5 | lincRNA | 25610 |
| 1.0452841 | NR_033360 | MEG3 | RefSeq_NR | 1621 |
| 1.0452922 | BX106769 | lincRNA-HOOK2 | lincRNA | 739 |
| 1.0454604 | AK302893 | lincRNA-MICAL2-1 | lincRNA | 3585 |
| 1.0459042 | BX478947 | lincRNA-CD53 | lincRNA | 685 |
| 1.045932 | chr5:67603294-67617869- | lincRNA-PIK3R1-10 | lincRNA | 14576 |
| 1.046342 | AI222143 | lincRNA-KIAA0802 | lincRNA | 282 |
| 1.0466682 | ENST00000429327 | BX322559.2 | Ensembl | 192 |
| 1.0467616 | ENST00000440744 | AC024084.1 | Ensembl | 457 |
| 1.0467899 | ENST00000406671 | RP3-351K20.4 | Ensembl | 891 |
| 1.047209 | CV326062 | lincRNA-COL12A1 | lincRNA | 435 |
| 1.0473105 | ENST00000420922 | AC073043.2 | Ensembl | 772 |
| 1.0473942 | ENST00000505100 | CTB-179I1.3 | Ensembl | 463 |
| 1.0475091 | ENST00000468320 | MT-ND4L | Ensembl | 276 |
| 1.0476772 | chr10:44737519-44749219+ | lincRNA-CXCL12-4 | lincRNA | 11701 |
| 1.0477325 | AK124423 |  | misc_RNA | 3991 |
| 1.0481562 | AV648429 | lincRNA-IGF1 | lincRNA | 688 |
| 1.0483886 | BM563811 | lincRNA-RPS24-6 | lincRNA | 1081 |
| 1.048482 | uc003cmy.1 | AK124776 | UCSC_knowngene | 4060 |
| 1.048489 | chr2:234783886-234795411- | lincRNA-HJURP-1 | lincRNA | 11526 |
| 1.0492002 | chrX:118607438-118617354+ | lincRNA-CXorf56-3 | lincRNA | 9917 |
| 1.0498841 | AW892975 | lincRNA-AMOTL1-1 | lincRNA | 457 |
| 1.0499267 | AK307134 |  | NRED | 840 |
| 1.0502135 | uc001uih.1 | AK056047 | UCSC_knowngene | 2922 |
| 1.0505873 | uc002ukz.1 | LOC375295 | UCSC_knowngene | 1156 |
| 1.0507543 | chrX:11801246-11812911- | lincRNA-MSL3 | lincRNA | 11666 |
| 1.0508337 | ENST00000454600 | RP4-723E3.1 | Ensembl | 745 |
| 1.0517847 | ENST00000423016 | CTD-2230M5.1 | Ensembl | 168 |
| 1.0519385 | AI859713 | lincRNA-NUDT4-2 | lincRNA | 489 |
| 1.0526912 | ENST00000450834 | RP11-192P3.1 | Ensembl | 564 |
| 1.052769 | chr6:112269457-112274293+ | lincRNA-WISP3-1 | lincRNA | 4837 |
| 1.0529904 | ENST00000340510 | AC004987.7 | Ensembl | 3049 |
| 1.0530306 | AA317289 | lincRNA-DLL1-1 | lincRNA | 416 |
| 1.0530871 | exon1607+ | lincRNA-PPP2R5C-2 | lincRNA | 191 |
| 1.0532326 | chrX:40823014-40854540+ | lincRNA-USP9X-4 | lincRNA | 31527 |
| 1.0532889 | chr4:10120852-10137502+ | lincRNA-ZNF518B-1 | lincRNA | 16651 |
| 1.0533941 | AK124473 |  | misc_RNA | 2210 |
| 1.0534439 | uc002tdf.2 | BC038566 | UCSC_knowngene | 1415 |
| 1.053456 | W19582 | lincRNA-ETS1-2 | lincRNA | 540 |
| 1.0536177 | uc004cxd.2 | BC032506 | UCSC_knowngene | 2419 |
| 1.0537498 | exon90+ | lincRNA-KNCN | lincRNA | 643 |
| 1.0539358 | ENST00000427064 | AC090505.1 | Ensembl | 464 |
| 1.0540614 | exon92+ | lincRNA-KNCN | lincRNA | 197 |
| 1.0540899 | BC014370 |  | NRED | 1587 |
| 1.0541435 | AK125369 |  | NRED | 3146 |
| 1.0541928 | ENST00000422221 | RP11-469E19.1 | Ensembl | 752 |
| 1.0542506 | chr16:15507881-15517161- | lincRNA-MPV17L | lincRNA | 9281 |
| 1.054549 | BG722956 | lincRNA-EGR2 | lincRNA | 833 |
| 1.0546025 | chr11:8215149-8227449+ | lincRNA-LMO1-2 | lincRNA | 12301 |
| 1.0546542 | BC043513 | lincRNA-FSCB | lincRNA | 916 |
| 1.0549435 | uc010aav.2 | BC132836 | UCSC_knowngene | 390 |
| 1.0549755 | ENST00000446783 | RP11-40H20.1 | Ensembl | 640 |
| 1.0549805 | chr11:79223852-79229652+ | lincRNA-FAM181B | lincRNA | 5801 |
| 1.0553345 | ENST00000515436 | RP11-617D20.1 | Ensembl | 513 |
| 1.0555389 | BE043072 | lincRNA-ZSWIM4-2 | lincRNA | 446 |
| 1.0556417 | uc003vrd.1 | AK097607 | UCSC_knowngene | 1998 |
| 1.0556867 | AK097728 | lincRNA-PRLHR-1 | lincRNA | 1798 |
| 1.0558069 | chr1:34821413-34838838+ | lincRNA-GJB5 | lincRNA | 17426 |
| 1.055877 | DA148762 | lincRNA-IRF4 | lincRNA | 529 |
| 1.0560368 | chr8:20578220-20590170- | lincRNA-LZTS1-1 | lincRNA | 11951 |
| 1.0560881 | uc003tbd.2 | BC041636 | UCSC_knowngene | 1130 |
| 1.0561296 | ENST00000503924 | AL121583.1 | Ensembl | 4986 |
| 1.0563997 | DA453447 | lincRNA-CLDN18 | lincRNA | 557 |
| 1.0567557 | NR_026975 | LOC286467 | RefSeq_NR | 2951 |
| 1.0570227 | R58488 | lincRNA-SRBD1-2 | lincRNA | 265 |
| 1.0572848 | CB051377 | lincRNA-ZNF703-3 | lincRNA | 599 |
| 1.0573679 | ENST00000436229 | RP11-185B14.1 | Ensembl | 259 |
| 1.0575066 | AI354683 | lincRNA-FRMD1-1 | lincRNA | 439 |
| 1.0575853 | AK125162 |  | misc_RNA | 2114 |
| 1.0579894 | ENST00000396848 | AC010138.3 | Ensembl | 867 |
| 1.0581746 | BU739232 | lincRNA-TMEM182-1 | lincRNA | 670 |
| 1.0583116 | AK095484 |  | misc_RNA | 2135 |
| 1.0585875 | DB111388 | lincRNA-MATN1 | lincRNA | 574 |
| 1.0587669 | NR_024053 | HCG18 | RefSeq_NR | 6600 |
| 1.058868 | AY421730 |  | RNAdb | 574 |
| 1.0592942 | chr15:78112795-78125995+ | lincRNA-TBC1D2B-2 | lincRNA | 13201 |
| 1.0594373 | uc001zcp.1 | BC043570 | UCSC_knowngene | 2525 |
| 1.0597061 | uc010ivk.2 | AK311398 | UCSC_knowngene | 988 |
| 1.0598392 | ENST00000455863 | IGKV1ORY-1 | Ensembl | 380 |
| 1.0598866 | chr1:203291278-203297051+ | lincRNA-FMOD | lincRNA | 5774 |
| 1.0600332 | ENST00000505771 | AC092275.1 | Ensembl | 1509 |
| 1.0602177 | ENST00000442852 | XXbac-BPG27H4.8 | Ensembl | 799 |
| 1.060461 | uc002nwj.2 | BC045806 | UCSC_knowngene | 2079 |
| 1.0607122 | ENST00000400856 | RP11-106M7.2 | Ensembl | 499 |
| 1.0608489 | ENST00000434020 | AC093609.1 | Ensembl | 952 |
| 1.0609601 | chr15:40446305-40450500+ | lincRNA-BUB1B | lincRNA | 4196 |
| 1.0614004 | chr2:18787244-18801494+ | lincRNA-OSR1-5 | lincRNA | 14251 |
| 1.0618127 | uc003lak.2 | BC045187 | UCSC_knowngene | 2159 |
| 1.0618562 | DB298687 | lincRNA-ERI1-2 | lincRNA | 507 |
| 1.0623029 | ENST00000449111 | RP13-507I23.1 | Ensembl | 3414 |
| 1.0628088 | ENST00000429469 | RP11-553K8.2 | Ensembl | 363 |
| 1.0628452 | chr14:94335247-94345947+ | lincRNA-FAM181A | lincRNA | 10701 |
| 1.0631177 | chr12:32192533-32208358+ | lincRNA-BICD1-2 | lincRNA | 15826 |
| 1.0640556 | chr2:202788106-202795396- | lincRNA-CDK15-2 | lincRNA | 7291 |
| 1.0650169 | chr10:130341500-130364281+ | lincRNA-MGMT-2 | lincRNA | 22782 |
| 1.0653809 | chr6:135420132-135451482+ | lincRNA-MYB | lincRNA | 31351 |
| 1.0655725 | chr2:19979944-19990294+ | lincRNA-TTC32-1 | lincRNA | 10351 |
| 1.0659798 | ENST00000427394 | RP11-497D6.4 | Ensembl | 1821 |
| 1.0661425 | NR_026849 | C18orf18 | RefSeq_NR | 1322 |
| 1.0670238 | CR594623 | lincRNA-TP53INP1-1 | lincRNA | 1606 |
| 1.067038 | uc010dmv.2 | DM004440 | UCSC_knowngene | 109 |
| 1.0676069 | CR620657 |  | RNAdb | 401 |
| 1.0679852 | AB208948 | lincRNA-STON2-2 | lincRNA | 4302 |
| 1.0683118 | chr2:159810159-159821179- | lincRNA-DAPL1-1 | lincRNA | 11021 |
| 1.0691105 | DA015073 | lincRNA-MAP2K3-2 | lincRNA | 587 |
| 1.06925 | NR_024330 | NCRNA00085 | RefSeq_NR | 2017 |
| 1.0694651 | AI609057 | lincRNA-LUZP6-1 | lincRNA | 417 |
| 1.0695993 | HIT000243085 | | H-invDB | 1541 |
| 1.0701869 | uc001gix.1 | AK127238 | UCSC_knowngene | 5619 |
| 1.070194 | ENST00000423187 | RP11-99H8.1 | Ensembl | 432 |
| 1.0702349 | AK126983 |  | misc_RNA | 5637 |
| 1.0704489 | chr20:48363997-48385346+ | lincRNA-SLC9A8 | lincRNA | 21350 |
| 1.0704694 | ENST00000419987 | RP11-188P8.2 | Ensembl | 799 |
| 1.0706069 | AV730200 | lincRNA-CNTN1 | lincRNA | 687 |
| 1.0706439 | chr12:63792383-63819758+ | lincRNA-DPY19L2 | lincRNA | 27376 |
| 1.0707785 | EL585127 | lincRNA-ZNF37A-2 | lincRNA | 805 |
| 1.0708162 | ENST00000435260 | AC046176.4 | Ensembl | 1170 |
| 1.071072 | chr2:151609179-151621879+ | lincRNA-RBM43-3 | lincRNA | 12701 |
| 1.0711221 | ENST00000393779 | AC104297.1 | Ensembl | 870 |
| 1.0712902 | NR_003500 | RP9P | RefSeq_NR | 1405 |
| 1.0714177 | AK124203 |  | misc_RNA | 2182 |
| 1.0714679 | ENST00000506795 | RP11-462C24.1 | Ensembl | 1136 |
| 1.071512 | ENST00000320802 | AC009530.1 | Ensembl | 830 |
| 1.0715724 | AI948514 | lincRNA-EML4 | lincRNA | 444 |
| 1.0730736 | BG494524 | lincRNA-TEKT2 | lincRNA | 772 |
| 1.0731544 | ENST00000417473 | AC099344.4 | Ensembl | 499 |
| 1.0744214 | AK023737 |  | misc_RNA | 1747 |
| 1.0744279 | chr2:192293450-192304436+ | lincRNA-OBFC2A-4 | lincRNA | 10987 |
| 1.0745733 | AL365456 |  | NRED | 2596 |
| 1.0746349 | ENST00000455212 | AC007386.3 | Ensembl | 582 |
| 1.0750697 | U29607 | lincRNA-ELK3 | lincRNA | 2569 |
| 1.0753545 | CD102777 | lincRNA-TPTE2 | lincRNA | 752 |
| 1.075426 | uc001uhk.3 | BC040060 | UCSC_knowngene | 586 |
| 1.075878 | uc003yrb.2 | CR933665 | UCSC_knowngene | 5639 |
| 1.0763605 | chr4:53420371-53428337- | lincRNA-SPATA18-2 | lincRNA | 7967 |
| 1.0768256 | ENST00000451774 | AC074093.1 | Ensembl | 2098 |
| 1.0768665 | ENST00000436430 | RP11-245J24.1 | Ensembl | 556 |
| 1.0771039 | uc003pwf.2 | BC042098 | UCSC_knowngene | 2007 |
| 1.0775102 | uc001wvl.1 | BX248273 | UCSC_knowngene | 871 |
| 1.0776693 | ENST00000419531 | RP4-794H19.2 | Ensembl | 2273 |
| 1.0776747 | AI734132 | lincRNA-RGS6-1 | lincRNA | 438 |
| 1.0777949 | uc003flq.1 | AK057000 | UCSC_knowngene | 1623 |
| 1.0778693 | ENST00000446953 | RP1-130G2.1 | Ensembl | 486 |
| 1.0779152 | uc003xth.2 | BC048118 | UCSC_knowngene | 1829 |
| 1.0779514 | CA430002 | lincRNA-HMGA2-3 | lincRNA | 688 |
| 1.0786501 | CK126152 | lincRNA-MSC | lincRNA | 683 |
| 1.0793163 | DB093487 | lincRNA-KIAA1024-2 | lincRNA | 553 |
| 1.0797294 | AK124909 |  | misc_RNA | 4625 |
| 1.0798973 | DB264073 | lincRNA-EGFL6-1 | lincRNA | 596 |
| 1.0800388 | ENST00000414621 | AC006027.2 | Ensembl | 230 |
| 1.0801496 | BX106988 | lincRNA-XKR4 | lincRNA | 677 |
| 1.0802464 | uc003jhx.2 | CR613092 | UCSC_knowngene | 708 |
| 1.0803944 | ENST00000432629 | RPL12P4 | Ensembl | 497 |
| 1.0807309 | ENST00000515236 | RP11-365H8.3 | Ensembl | 1327 |
| 1.0812584 | chr4:25443352-25460177+ | lincRNA-SLC34A2 | lincRNA | 16826 |
| 1.08154 | ENST00000451515 | AC010733.5 | Ensembl | 400 |
| 1.082436 | exon3074- | lincRNA-ZNF608-1 | lincRNA | 241 |
| 1.0825017 | uc002qsa.2 | LOC113386 | UCSC_knowngene | 1396 |
| 1.0829392 | ENST00000418851 | AC008280.1 | Ensembl | 412 |
| 1.083022 | uc003wma.1 | BC150495 | UCSC_knowngene | 1551 |
| 1.0833823 | ENST00000447876 | AC018730.1 | Ensembl | 607 |
| 1.0834254 | chr3:122764889-122778226- | lincRNA-SEMA5B-1 | lincRNA | 13338 |
| 1.0836894 | ENST00000422415 | AC010145.4 | Ensembl | 517 |
| 1.0839427 | chr3:187039181-187056506+ | lincRNA-RTP4 | lincRNA | 17326 |
| 1.0843622 | uc003fbc.2 | AK311218 | UCSC_knowngene | 2015 |
| 1.0844517 | AI808995 | lincRNA-PLEKHA1 | lincRNA | 438 |
| 1.0844758 | DA335567 | lincRNA-NT5C1B-RDH14-4 | lincRNA | 585 |
| 1.0846063 | chr8:49776604-49779322+ | lincRNA-SNAI2-1 | lincRNA | 2719 |
| 1.0848798 | NR_024507 | LOC646982 | RefSeq_NR | 3972 |
| 1.0852323 | ENST00000413645 | AP000473.5 | Ensembl | 550 |
| 1.0853153 | ENST00000445636 | RP11-369L4.1 | Ensembl | 354 |
| 1.085322 | chr8:82280811-82287539+ | lincRNA-PMP2 | lincRNA | 6729 |
| 1.0856845 | AI741919 | lincRNA-INO80D | lincRNA | 468 |
| 1.085876 | ENST00000423268 | RP11-631F7.1 | Ensembl | 311 |
| 1.0861559 | NR_027046 | LOC145474 | RefSeq_NR | 1859 |
| 1.0863065 | uc002kmn.1 | LOC339290 | UCSC_knowngene | 977 |
| 1.0870192 | ENST00000448776 | GS1-124K5.10 | Ensembl | 712 |
| 1.0871207 | chr8:10448515-10454758+ | lincRNA-RP1L1 | lincRNA | 6244 |
| 1.0871375 | DB288399 | lincRNA-ZNF672 | lincRNA | 558 |
| 1.0872217 | AF085351 |  | NRED | 207 |
| 1.0876825 | AW510921 | lincRNA-C6orf191 | lincRNA | 509 |
| 1.0877603 | ENST00000422346 | AC008280.4 | Ensembl | 732 |
| 1.0880958 | BU732707 | lincRNA-FLJ37543-2 | lincRNA | 601 |
| 1.0886083 | chr1:77167210-77175109- | lincRNA-ST6GALNAC3 | lincRNA | 7900 |
| 1.0887941 | AK289547 | lincRNA-CDH18-2 | lincRNA | 3540 |
| 1.0891774 | ENST00000497346 | RP11-397J20.1 | Ensembl | 1441 |
| 1.0893339 | chrX:40874482-40878593+ | lincRNA-USP9X-7 | lincRNA | 4112 |
| 1.0896699 | chr14:77589570-77596097- | lincRNA-KIAA1737-2 | lincRNA | 6528 |
| 1.0898598 | AF088007 |  | misc_RNA | 706 |
| 1.090213 | DA834198 | lincRNA-CYP7B1-1 | lincRNA | 884 |
| 1.0905346 | ENST00000479812 | RP11-434O22.2 | Ensembl | 475 |
| 1.0923156 | ENST00000416154 | AC079305.5 | Ensembl | 562 |
| 1.0929333 | AK095773 |  | misc_RNA | 2533 |
| 1.0930046 | T54356 | lincRNA-CHST1 | lincRNA | 417 |
| 1.093025 | CB857271 | lincRNA-DLK1-1 | lincRNA | 349 |
| 1.0930711 | BE048920 | lincRNA-FBXL4 | lincRNA | 329 |
| 1.093689 | uc001tcu.2 | AK098304 | UCSC_knowngene | 3324 |
| 1.0942141 | BU663229 | lincRNA-POU3F1-1 | lincRNA | 555 |
| 1.0942827 | chr12:68307933-68321375+ | lincRNA-IFNG-2 | lincRNA | 13443 |
| 1.0944002 | AK095250 |  | misc_RNA | 3037 |
| 1.0947009 | AK293938 | lincRNA-ZNF638 | lincRNA | 3515 |
| 1.0948584 | BF869762 | lincRNA-FLJ37543-1 | lincRNA | 115 |
| 1.0948681 | chr6:8104078-8113119- | lincRNA-EEF1E1 | lincRNA | 9042 |
| 1.0949079 | ENST00000456581 | RP11-500G10.1 | Ensembl | 2843 |
| 1.096318 | chr8:48104990-48105990- | lincRNA-POTEA-2 | lincRNA | 1001 |
| 1.0963836 | AY726573 |  | misc_RNA | 771 |
| 1.0969243 | AA136769 | lincRNA-INSIG2-2 | lincRNA | 356 |
| 1.0972388 | uc003gid.2 | BC025734 | UCSC_knowngene | 2403 |
| 1.0976778 | ENST00000458414 | RP11-423O2.1 | Ensembl | 1069 |
| 1.0984307 | BM807096 | lincRNA-GRIA2-2 | lincRNA | 1041 |
| 1.0986411 | ENST00000414823 | AC009413.2 | Ensembl | 399 |
| 1.098657 | ENST00000476147 | RP11-587D21.1 | Ensembl | 375 |
| 1.0987243 | ENST00000433327 | RP11-423O2.4 | Ensembl | 153 |
| 1.0988566 | AX746929 | lincRNA-SAMD3 | lincRNA | 2376 |
| 1.0989081 | DR423683 | lincRNA-DLG1 | lincRNA | 732 |
| 1.0991213 | AK123657 |  | misc_RNA | 2126 |
| 1.0993019 | AB058771 | lincRNA-B3GNT7 | lincRNA | 3748 |
| 1.09937 | chr9:20028084-20041430+ | lincRNA-MLLT3-1 | lincRNA | 13347 |
| 1.0996164 | ENST00000510145 | CTD-2340E1.3 | Ensembl | 944 |
| 1.0997194 | ENST00000440360 | AC079767.3 | Ensembl | 171 |
| 1.1003136 | chr13:25650975-25663125+ | lincRNA-PABPC3 | lincRNA | 12151 |
| 1.1003877 | AK055989 |  | misc_RNA | 2012 |
| 1.1005031 | DB519945 | lincRNA-DDX6 | lincRNA | 430 |
| 1.1006651 | ENST00000417698 | AC096732.1 | Ensembl | 477 |
| 1.1008627 | AA938966 | lincRNA-FYN-1 | lincRNA | 408 |
| 1.1012559 | chr14:84962047-84980772+ | lincRNA-FLRT2-4 | lincRNA | 18726 |
| 1.1014132 | BX105843 | lincRNA-VCX | lincRNA | 455 |
| 1.1022709 | DB130948 | lincRNA-BARHL2-1 | lincRNA | 346 |
| 1.1023072 | BE350869 | lincRNA-LRFN5-1 | lincRNA | 515 |
| 1.1024134 | chr10:102402035-102412460- | lincRNA-HIF1AN-1 | lincRNA | 10426 |
| 1.1025265 | uc004bad.1 | AK057451 | UCSC_knowngene | 1544 |
| 1.1027333 | ENST00000451906 | AC093038.1 | Ensembl | 497 |
| 1.103171 | DW462947 | lincRNA-GNLY | lincRNA | 365 |
| 1.1032028 | U79248 |  | NRED | 1615 |
| 1.1033125 | chr12:46477908-46488783- | lincRNA-SRSF2IP | lincRNA | 10876 |
| 1.1035021 | ENST00000447943 | RP11-78A18.2 | Ensembl | 299 |
| 1.1035097 | chr7:127179214-127190764+ | lincRNA-GCC1 | lincRNA | 11551 |
| 1.1039238 | ENST00000432358 | RP11-732M18.3 | Ensembl | 424 |
| 1.1046683 | ENST00000467573 | AF064859.2 | Ensembl | 473 |
| 1.1046726 | ENST00000420156 | RP4-543J13.1 | Ensembl | 332 |
| 1.1046791 | chr6:89272256-89285156- | lincRNA-CNR1 | lincRNA | 12901 |
| 1.1049918 | AK128224 |  | misc_RNA | 3579 |
| 1.1051197 | BQ695043 | lincRNA-FRZB | lincRNA | 726 |
| 1.1051227 | ENST00000417957 | RP11-375H17.1 | Ensembl | 451 |
| 1.1052504 | BC045720 |  | RNAdb | 1844 |
| 1.1052531 | BX956284 | lincRNA-MYO1B-2 | lincRNA | 566 |
| 1.1059919 | AF088026 |  | RNAdb | 703 |
| 1.1061019 | CB157495 | lincRNA-NPVF-2 | lincRNA | 608 |
| 1.1063107 | uc003npw.2 | HCG18 | UCSC_knowngene | 6026 |
| 1.1063499 | EG328305 | lincRNA-NCS1 | lincRNA | 333 |
| 1.1064342 | ENST00000446970 | RP11-568F20.1 | Ensembl | 266 |
| 1.1064356 | ENST00000454752 | RP11-151A6.4 | Ensembl | 790 |
| 1.1064643 | ENST00000417997 | AC073472.2 | Ensembl | 600 |
| 1.1065942 | ENST00000449106 | RP11-85P9.3 | Ensembl | 427 |
| 1.1067319 | ENST00000430258 | RP1-298J18.1 | Ensembl | 551 |
| 1.1071087 | DQ275636 |  | RNAdb | 682 |
| 1.1072026 | AW816050 | lincRNA-CCDC54-1 | lincRNA | 147 |
| 1.1072217 | DB471974 | lincRNA-SEC61B-2 | lincRNA | 478 |
| 1.1073309 | uc002nry.1 | BC024732 | UCSC_knowngene | 827 |
| 1.1075185 | T02983 | lincRNA-LOC285733-1 | lincRNA | 500 |
| 1.1083968 | chr5:141438289-141445854- | lincRNA-GNPDA1 | lincRNA | 7566 |
| 1.108536 | BX096603 | lincRNA-CCND2-3 | lincRNA | 733 |
| 1.1093187 | chr15:77989870-78011295+ | lincRNA-TBC1D2B-1 | lincRNA | 21426 |
| 1.109398 | AA384551 | lincRNA-KIF13B | lincRNA | 304 |
| 1.1094179 | chr14:59616072-59635747- | lincRNA-DACT1 | lincRNA | 19676 |
| 1.1099193 | BC065911 |  | NRED | 2987 |
| 1.1099468 | ENST00000433280 | RP11-547C18.3 | Ensembl | 431 |
| 1.1101736 | AK303027 | lincRNA-SMAD4 | lincRNA | 1207 |
| 1.1104155 | AK294916 | lincRNA-CABLES1 | lincRNA | 1404 |
| 1.1109391 | BC002759 | lincRNA-SLC48A1 | lincRNA | 1041 |
| 1.1110178 | CD514437 | lincRNA-WDFY1 | lincRNA | 836 |
| 1.1117534 | ENST00000440255 | RP1-213J1P__B.1 | Ensembl | 819 |
| 1.1118666 | ENST00000419499 | RP11-459P23.1 | Ensembl | 3340 |
| 1.1120252 | DA195606 | lincRNA-FRMD6-3 | lincRNA | 564 |
| 1.1125965 | ENST00000399853 | CTD-2247C11.3 | Ensembl | 1565 |
| 1.1126723 | exon1336- | lincRNA-OLFM4-5 | lincRNA | 358 |
| 1.1129213 | exon2234- | lincRNA-INHBB-4 | lincRNA | 451 |
| 1.1136594 | chr14:66457497-66472547+ | lincRNA-GPHN-4 | lincRNA | 15051 |
| 1.1141866 | ENST00000428766 | RP11-57H14.3 | Ensembl | 317 |
| 1.1147033 | ENST00000427761 | RP11-248N6.3 | Ensembl | 952 |
| 1.1147285 | chr13:49123374-49135499+ | lincRNA-CYSLTR2-2 | lincRNA | 12126 |
| 1.1149209 | uc003enb.1 | CR593374 | UCSC_knowngene | 3495 |
| 1.1152139 | BI459631 | lincRNA-GUCA2B | lincRNA | 732 |
| 1.115312 | chr3:167821006-167840006- | lincRNA-GOLIM4-2 | lincRNA | 19001 |
| 1.1154143 | ENST00000514568 | AC092279.2 | Ensembl | 842 |
| 1.1155485 | HIT000045925 | | H-invDB | 3943 |
| 1.1157595 | AK056959 | lincRNA-C4orf26 | lincRNA | 1122 |
| 1.115778 | chr3:13556998-13571393+ | lincRNA-FBLN2-1 | lincRNA | 14396 |
| 1.1164444 | uc001ijl.2 | AY007155 | UCSC_knowngene | 1012 |
| 1.1167097 | BM969128 | lincRNA-TP53INP1-3 | lincRNA | 794 |
| 1.1177085 | AY927493 |  | misc_RNA | 130 |
| 1.118079 | AF210650 |  | misc_RNA | 1491 |
| 1.1187268 | ENST00000425638 | RP11-173P16.2 | Ensembl | 1082 |
| 1.1195429 | AK054953 |  | misc_RNA | 2228 |
| 1.1196959 | AV651118 | lincRNA-A2ML1 | lincRNA | 681 |
| 1.1204669 | chr13:112169174-112181374- | lincRNA-C13orf16-3 | lincRNA | 12201 |
| 1.1207259 | uc009wxq.1 | BC039493 | UCSC_knowngene | 538 |
| 1.1215052 | ENST00000455561 | RP4-575N6.2 | Ensembl | 343 |
| 1.1216273 | G43210 |  | misc_RNA | 283 |
| 1.1219884 | ENST00000447179 | RP11-146I2.2 | Ensembl | 177 |
| 1.1220989 | chr12:55389508-55403008- | lincRNA-KIAA0748 | lincRNA | 13501 |
| 1.1222194 | NR_033373 | FLJ39534 | RefSeq_NR | 2540 |
| 1.1226208 | chr11:130081529-130089431+ | lincRNA-ZBTB44-1 | lincRNA | 7903 |
| 1.1230493 | ENST00000495944 | CTD-2127O16.1 | Ensembl | 589 |
| 1.1231486 | NR_026862 | PPP1R3E | RefSeq_NR | 4521 |
| 1.1234717 | ENST00000424151 | AC006378.3 | Ensembl | 314 |
| 1.1243675 | BG190216 | lincRNA-MYL2 | lincRNA | 744 |
| 1.124428 | chr17:8905860-8924601+ | lincRNA-NTN1 | lincRNA | 18742 |
| 1.1244299 | uc002vvl.1 | AX748264 | UCSC_knowngene | 1811 |
| 1.1249628 | NR_023938 | C14orf132 | RefSeq_NR | 7504 |
| 1.1253816 | ENST00000470024 | RP11-451G4.3 | Ensembl | 444 |
| 1.1255764 | ENST00000445730 | RP11-525A16.1 | Ensembl | 497 |
| 1.1256447 | CV340376 | lincRNA-CXorf66-1 | lincRNA | 148 |
| 1.1256658 | NR_002578 | GAS5 | RefSeq_NR | 651 |
| 1.1264048 | chr10:112147868-112175330+ | lincRNA-DUSP5-3 | lincRNA | 27463 |
| 1.1264348 | NR_024259 | LOC728606 | RefSeq_NR | 2598 |
| 1.1269511 | ENST00000430650 | AC009236.1 | Ensembl | 721 |
| 1.1273562 | uc001nnk.1 | AB231722 | UCSC_knowngene | 158 |
| 1.1275829 | ENST00000359165 | EEF1A1P12 | Ensembl | 1341 |
| 1.1277954 | AW868654 | lincRNA-ME3 | lincRNA | 206 |
| 1.1279048 | uc010lea.1 | AK311103 | UCSC_knowngene | 3978 |
| 1.1279133 | BF116112 | lincRNA-C5orf53-1 | lincRNA | 588 |
| 1.1282331 | BX497352 | lincRNA-POU5F1B-1 | lincRNA | 800 |
| 1.128436 | FN053659 | lincRNA-ACTBL2-5 | lincRNA | 75 |
| 1.1284944 | exon2236+ | lincRNA-GLI2-4 | lincRNA | 466 |
| 1.128969 | ENST00000441283 | C21orf84 | Ensembl | 448 |
| 1.1290135 | ENST00000485404 | RP11-433A10.1 | Ensembl | 555 |
| 1.1299455 | CN412465 | lincRNA-FGD2 | lincRNA | 563 |
| 1.1300355 | ENST00000509266 | AC026979.1 | Ensembl | 1967 |
| 1.1300958 | DA706379 | lincRNA-C1QTNF4 | lincRNA | 574 |
| 1.1301162 | chr1:157444726-157458601- | lincRNA-ETV3-2 | lincRNA | 13876 |
| 1.13071 | CD050812 | lincRNA-PELP1 | lincRNA | 1033 |
| 1.1333096 | BU164084 | lincRNA-CDH5-1 | lincRNA | 921 |
| 1.1337812 | ENST00000424933 | AP001631.9 | Ensembl | 422 |
| 1.13489 | AW300693 | lincRNA-PTP4A2-2 | lincRNA | 506 |
| 1.1350271 | AV714079 |  | RNAdb | 721 |
| 1.1361293 | AL711564 | lincRNA-PIM1 | lincRNA | 605 |
| 1.1376954 | BE093687 | lincRNA-IL7-1 | lincRNA | 164 |
| 1.1378087 | ENST00000504610 | AP002841.2 | Ensembl | 2746 |
| 1.1378227 | ENST00000471719 | RP11-6F2.6 | Ensembl | 791 |
| 1.1384776 | DB251409 | lincRNA-STARD3NL-2 | lincRNA | 551 |
| 1.1388663 | chrX:36772104-36806404+ | lincRNA-FAM47C | lincRNA | 34301 |
| 1.139086 | AI621345 | lincRNA-SLC26A3 | lincRNA | 351 |
| 1.1394182 | NR_026963 | LOC284900 | RefSeq_NR | 5125 |
| 1.139941 | ENST00000439492 | AP000350.6 | Ensembl | 1763 |
| 1.1399829 | CD106372 | lincRNA-USP9X-1 | lincRNA | 850 |
| 1.1401303 | chr5:141438289-141445854+ | lincRNA-NDFIP1 | lincRNA | 7566 |
| 1.1402913 | BE796744 | lincRNA-ZPLD1-1 | lincRNA | 961 |
| 1.1412599 | ENST00000442155 | GS1-433O24.1 | Ensembl | 382 |
| 1.1414862 | uc002aqr.1 | BC047537 | UCSC_knowngene | 3969 |
| 1.1415486 | exon2751- | lincRNA-PLXNA1-4 | lincRNA | 101 |
| 1.1428086 | ENST00000508241 | RP11-231C18.2 | Ensembl | 540 |
| 1.14297 | BC015716 |  | misc_RNA | 1903 |
| 1.1432831 | ENST00000457669 | AP000696.2 | Ensembl | 1133 |
| 1.1433566 | CN281635 | lincRNA-CDC5L-3 | lincRNA | 717 |
| 1.143811 | chr4:177356004-177360135- | lincRNA-SPCS3-2 | lincRNA | 4132 |
| 1.1447133 | chr13:28387150-28402025- | lincRNA-GSX1 | lincRNA | 14876 |
| 1.1455322 | chr5:172618919-172633619+ | lincRNA-NKX2-5-1 | lincRNA | 14701 |
| 1.1458005 | chr15:56580983-56592133- | lincRNA-RFX7 | lincRNA | 11151 |
| 1.1459203 | BC032831 | lincRNA-DAAM1-1 | lincRNA | 2557 |
| 1.1464454 | AY444560 | lincRNA-C8orf38-2 | lincRNA | 1110 |
| 1.146643 | ENST00000419108 | AC078841.5 | Ensembl | 1849 |
| 1.1469232 | AW749030 | lincRNA-POLE2 | lincRNA | 542 |
| 1.1472965 | DB030353 | lincRNA-RPUSD4-2 | lincRNA | 613 |
| 1.1474004 | CR611332 |  | misc_RNA | 2122 |
| 1.147433 | BQ045000 | lincRNA-NUDT15-1 | lincRNA | 615 |
| 1.1475464 | exon101+ | lincRNA-CDCP2 | lincRNA | 233 |
| 1.1481384 | chr9:33081447-33083405+ | lincRNA-B4GALT1 | lincRNA | 1959 |
| 1.1481425 | exon2440- | lincRNA-CEBPB-1 | lincRNA | 141 |
| 1.1483652 | ENST00000435146 | RP11-12N13.6 | Ensembl | 291 |
| 1.1485758 | ENST00000421002 | RP11-380B4.2 | Ensembl | 903 |
| 1.1501853 | chr8:129188666-129196194- | lincRNA-MYC-5 | lincRNA | 7529 |
| 1.1504502 | chr1:199663652-199681527- | lincRNA-PTPRC-6 | lincRNA | 17876 |
| 1.1508163 | ENST00000456816 | RP11-513G11.1 | Ensembl | 796 |
| 1.1508879 | FN113698 | lincRNA-NFIA-2 | lincRNA | 61 |
| 1.1516697 | AA668276 | lincRNA-TMEM37 | lincRNA | 293 |
| 1.1516992 | NR_033314 | BDNFOS | RefSeq_NR | 1478 |
| 1.1521959 | ENST00000487122 | AC006427.1 | Ensembl | 248 |
| 1.15305 | ENST00000514578 | RP11-83M16.4 | Ensembl | 389 |
| 1.1531129 | chr6:48505291-48527891- | lincRNA-C6orf138-3 | lincRNA | 22601 |
| 1.154032 | AK124936 | lincRNA-FAM47E | lincRNA | 1624 |
| 1.1546283 | NR_003277 | LOC728643 | RefSeq_NR | 542 |
| 1.1552005 | DN918054 | lincRNA-PDE4B | lincRNA | 400 |
| 1.1558549 | ENST00000453261 | AC073387.2 | Ensembl | 709 |
| 1.1563913 | chr6:118113617-118118358+ | lincRNA-SLC35F1-1 | lincRNA | 4742 |
| 1.156519 | NR_015367 | LOC550643 | RefSeq_NR | 722 |
| 1.1570892 | DB330880 | lincRNA-THAP6 | lincRNA | 548 |
| 1.1572455 | ENST00000500949 | AC012652.2 | Ensembl | 4265 |
| 1.1580299 | BX648169 |  | misc_RNA | 3046 |
| 1.1586958 | chr10:4578200-4593225+ | lincRNA-AKR1E2-1 | lincRNA | 15026 |
| 1.1592993 | DB567533 | lincRNA-AMOT-1 | lincRNA | 468 |
| 1.1596649 | AF289584 | lincRNA-NKD1 | lincRNA | 2142 |
| 1.1599777 | chr12:72131475-72142047- | lincRNA-TMEM19 | lincRNA | 10573 |
| 1.1613949 | BX646285 | lincRNA-LZTS1-2 | lincRNA | 659 |
| 1.1615511 | AA745165 | lincRNA-C18orf45 | lincRNA | 231 |
| 1.162139 | ENST00000411449 | RP11-120J20.1 | Ensembl | 460 |
| 1.163716 | chr16:88450663-88475686- | lincRNA-BANP-2 | lincRNA | 25024 |
| 1.1642421 | AB002448 |  | NRED | 1270 |
| 1.1642538 | chr9:33081447-33083405- | lincRNA-SMU1 | lincRNA | 1959 |
| 1.1647288 | ENST00000442229 | RP11-248N6.1 | Ensembl | 888 |
| 1.1652006 | ENST00000415499 | AC005062.2 | Ensembl | 743 |
| 1.1659757 | chr2:174248004-174261129+ | lincRNA-SP3-2 | lincRNA | 13126 |
| 1.1660846 | DB455579 | lincRNA-ABCD3 | lincRNA | 459 |
| 1.1660982 | DA728743 | lincRNA-FOXA1 | lincRNA | 789 |
| 1.1661859 | ENST00000423089 | AP001434.1 | Ensembl | 438 |
| 1.1664562 | ENST00000463466 | RP11-874G11.1 | Ensembl | 464 |
| 1.1667815 | chr5:3223050-3239025- | lincRNA-C5orf38-2 | lincRNA | 15976 |
| 1.1674559 | ENST00000449714 | AC144449.1 | Ensembl | 1022 |
| 1.1681777 | AW800975 | lincRNA-AGRP-1 | lincRNA | 537 |
| 1.1683101 | ENST00000451066 | AC009473.1 | Ensembl | 692 |
| 1.1685638 | DA223008 | lincRNA-PARP11-3 | lincRNA | 495 |
| 1.1689455 | uc003yao.3 | BC062758 | UCSC_knowngene | 1142 |
| 1.1705541 | uc003tab.2 | BC038570 | UCSC_knowngene | 1426 |
| 1.1713659 | BG184634 | lincRNA-OTP | lincRNA | 320 |
| 1.1714694 | uc002nrr.1 | AK092080 | UCSC_knowngene | 1970 |
| 1.1716285 | AK091805 |  | misc_RNA | 2929 |
| 1.1719759 | NR_027898 | COL6A4P2 | RefSeq_NR | 9161 |
| 1.1725121 | ENST00000449539 | AC068491.3 | Ensembl | 1241 |
| 1.1726354 | CN413083 | lincRNA-UFC1 | lincRNA | 687 |
| 1.1731719 | NR_026761 | C1orf97 | RefSeq_NR | 797 |
| 1.1736755 | chr2:102572832-102577023- | lincRNA-MAP4K4 | lincRNA | 4192 |
| 1.1747883 | NR_033313 | BDNFOS | RefSeq_NR | 1279 |
| 1.1758527 | NR_027105 | LOC285419 | RefSeq_NR | 566 |
| 1.1764045 | X75546 | lincRNA-BTG2 | lincRNA | 1247 |
| 1.1769486 | ENST00000449911 | CTD-2230M5.2 | Ensembl | 585 |
| 1.1778766 | chr10:58962869-58979819+ | lincRNA-IPMK-1 | lincRNA | 16951 |
| 1.178503 | chr4:6753049-6763474- | lincRNA-CNO | lincRNA | 10426 |
| 1.17857 | chr14:93595225-93603463- | lincRNA-ITPK1 | lincRNA | 8239 |
| 1.1786763 | AL563761 | lincRNA-WDR59-1 | lincRNA | 1187 |
| 1.1786967 | ENST00000478745 | RP11-654C22.2 | Ensembl | 866 |
| 1.1788095 | AF052103 |  | NRED | 1329 |
| 1.1790405 | chr7:41044125-41053899- | lincRNA-C7orf10-3 | lincRNA | 9775 |
| 1.1798771 | ENST00000438679 | RP5-1065P14.1 | Ensembl | 415 |
| 1.1807779 | CN286266 | lincRNA-PLP1 | lincRNA | 674 |
| 1.1809098 | BC146281 | lincRNA-C14orf104 | lincRNA | 3292 |
| 1.1816254 | uc003mts.1 | AK023629 | UCSC_knowngene | 2287 |
| 1.1821172 | AK296474 | lincRNA-PPP2R2C-1 | lincRNA | 1780 |
| 1.1824024 | ENST00000421078 | AC008155.1 | Ensembl | 329 |
| 1.1827039 | chr7:37833800-37845625- | lincRNA-GPR141-2 | lincRNA | 11826 |
| 1.1830323 | BU690073 | lincRNA-CCDC33 | lincRNA | 416 |
| 1.183055 | DB111273 | lincRNA-P2RY1-1 | lincRNA | 575 |
| 1.1839345 | ENST00000478813 | RP11-941H19.1 | Ensembl | 628 |
| 1.1841353 | ENST00000509878 | RP11-792D21.1 | Ensembl | 289 |
| 1.1843894 | ENST00000457776 | RP11-423O2.6 | Ensembl | 1746 |
| 1.1844442 | AK056943 |  | misc_RNA | 1856 |
| 1.1844821 | BF511390 | lincRNA-STS | lincRNA | 344 |
| 1.1847495 | NR_023915 | IPW | RefSeq_NR | 4498 |
| 1.1855858 | NR_026542 | C21orf88 | RefSeq_NR | 1469 |
| 1.1865099 | ENST00000508720 | RP11-584P21.4 | Ensembl | 297 |
| 1.1870937 | ENST00000413637 | RP11-315H15.1 | Ensembl | 657 |
| 1.1875301 | BG188549 | lincRNA-PDE5A-2 | lincRNA | 499 |
| 1.1896063 | uc003yet.2 | BC067244 | UCSC_knowngene | 2078 |
| 1.189827 | BX415539 | lincRNA-MRPS30 | lincRNA | 932 |
| 1.1900826 | ENST00000450205 | AP001046.4 | Ensembl | 699 |
| 1.1905765 | AI014992 | lincRNA-KLRB1 | lincRNA | 458 |
| 1.1906178 | ENST00000359838 | RP3-337D23.3 | Ensembl | 453 |
| 1.192475 | uc001utu.2 | EEF1DP3 | UCSC_knowngene | 1367 |
| 1.1931281 | ENST00000515403 | RP11-844P9.2 | Ensembl | 3632 |
| 1.1936612 | DA969401 | lincRNA-AKAP7 | lincRNA | 564 |
| 1.1941287 | chr8:66027310-66057986- | lincRNA-CYP7B1-4 | lincRNA | 30677 |
| 1.1944443 | BF679456 | lincRNA-OPCML | lincRNA | 700 |
| 1.19458 | chr20:38843986-38912961- | lincRNA-DHX35 | lincRNA | 68976 |
| 1.1950626 | AL046830 | lincRNA-CBLB-2 | lincRNA | 644 |
| 1.1956511 | ENST00000395772 | EEF1A1P15 | Ensembl | 1352 |
| 1.1959563 | chr1:10911613-10923588- | lincRNA-CASZ1 | lincRNA | 11976 |
| 1.196884 | chr13:105757124-105768999- | lincRNA-SLC10A2-6 | lincRNA | 11876 |
| 1.1980829 | BX114959 | lincRNA-OLA1-2 | lincRNA | 745 |
| 1.198557 | chr15:38053658-38074458+ | lincRNA-TMCO5A-3 | lincRNA | 20801 |
| 1.1989205 | DA444159 | lincRNA-SLCO2A1 | lincRNA | 593 |
| 1.1990408 | ENST00000411804 | RP1-91G5.3 | Ensembl | 707 |
| 1.1993079 | AW470559 | lincRNA-LOC100500938-1 | lincRNA | 507 |
| 1.1993639 | BX485243 | lincRNA-SRP54 | lincRNA | 590 |
| 1.1996681 | ENST00000503606 | CTD-2201E18.1 | Ensembl | 469 |
| 1.199697 | BC041387 | lincRNA-MCTP2-2 | lincRNA | 4605 |
| 1.1999328 | ENST00000404600 | RP11-157J24.1 | Ensembl | 1589 |
| 1.2004121 | BC030521 | lincRNA-PTCD2 | lincRNA | 2134 |
| 1.2012737 | ENST00000399168 | RP11-457D13.1 | Ensembl | 635 |
| 1.2015381 | BX384541 | lincRNA-FAM102B-2 | lincRNA | 1103 |
| 1.2017376 | ENST00000439405 | AF186191.1 | Ensembl | 1155 |
| 1.2018441 | chr3:45207146-45221396+ | lincRNA-TMEM158 | lincRNA | 14251 |
| 1.2020326 | CR603409 | lincRNA-FAM156A | lincRNA | 1248 |
| 1.2026477 | AW845646 | lincRNA-MAP3K1-3 | lincRNA | 612 |
| 1.2028665 | chr1:186587114-186596603+ | lincRNA-PTGS2-3 | lincRNA | 9490 |
| 1.2037709 | G36709 |  | misc_RNA | 463 |
| 1.2040579 | AK299698 | lincRNA-IMPACT-2 | lincRNA | 1470 |
| 1.2050631 | ENST00000425455 | AL592494.3 | Ensembl | 1550 |
| 1.205148 | BX096431 | lincRNA-BBOX1-2 | lincRNA | 738 |
| 1.2052914 | ENST00000473110 | RP11-373E16.4 | Ensembl | 263 |
| 1.2053867 | ENST00000443548 | BX004987.4 | Ensembl | 681 |
| 1.2074568 | U66048 |  | NRED | 2959 |
| 1.2077978 | ENST00000494283 | RP11-58E21.1 | Ensembl | 713 |
| 1.2081969 | uc003kuo.2 | FLJ33630 | UCSC_knowngene | 1870 |
| 1.2095778 | exon91- | lincRNA-DMBX1 | lincRNA | 175 |
| 1.2096627 | uc002nrw.1 | AK094188 | UCSC_knowngene | 2480 |
| 1.2120278 | uc001rjo.1 | BC072678 | UCSC_knowngene | 1645 |
| 1.2121971 | BC036795 | lincRNA-ZNF732-2 | lincRNA | 2220 |
| 1.2130537 | ENST00000435914 | RP1-197O17.2 | Ensembl | 430 |
| 1.2130818 | ENST00000433152 | RP11-222A11.1 | Ensembl | 744 |
| 1.2131269 | AA782034 | lincRNA-CDH11-3 | lincRNA | 196 |
| 1.2149346 | chr6:79218969-79313545+ | lincRNA-IRAK1BP1-1 | lincRNA | 94577 |
| 1.2150373 | uc003plc.3 | SNHG5 | UCSC_knowngene | 1078 |
| 1.2155325 | AK130597 |  | misc_RNA | 2295 |
| 1.2157949 | BC036541 | lincRNA-ZNF280D | lincRNA | 4811 |
| 1.2158282 | BG195666 | lincRNA-NAT1-1 | lincRNA | 580 |
| 1.2159599 | DB306154 | lincRNA-MPHOSPH6 | lincRNA | 518 |
| 1.2162524 | ENST00000500215 | AC007686.1 | Ensembl | 2924 |
| 1.2166414 | BF931474 | lincRNA-EN1-4 | lincRNA | 370 |
| 1.2177184 | exon3076+ | lincRNA-GRAMD3-1 | lincRNA | 926 |
| 1.2177891 | AK128747 |  | misc_RNA | 5937 |
| 1.2195842 | ENST00000450133 | RP9P | Ensembl | 1212 |
| 1.2204901 | ENST00000415506 | AC073254.1 | Ensembl | 743 |
| 1.2212637 | ENST00000433388 | RP1-137D17.1 | Ensembl | 588 |
| 1.222083 | ENST00000470049 | RP11-727C1.1 | Ensembl | 468 |
| 1.222469 | chr2:84696639-84713689- | lincRNA-SUCLG1-3 | lincRNA | 17051 |
| 1.2226226 | ENST00000513734 | RP11-155L15.2 | Ensembl | 295 |
| 1.2226754 | uc001ejn.1 | BC070106 | UCSC_knowngene | 1888 |
| 1.2231765 | EF565096 | lincRNA-CSMD3-1 | lincRNA | 324 |
| 1.2232529 | BC040578 |  | NRED | 3284 |
| 1.2234108 | ENST00000425294 | RP3-471M13.1 | Ensembl | 312 |
| 1.2236751 | AI203030 | lincRNA-UBE2V2-1 | lincRNA | 246 |
| 1.2237157 | NR_026973 | LOC285830 | RefSeq_NR | 698 |
| 1.2269675 | uc003dwf.3 | LOC100302640 | UCSC_knowngene | 2722 |
| 1.2277433 | ENST00000508827 | AL355916.1 | Ensembl | 5103 |
| 1.2285344 | ENST00000421426 | RP11-314P12.3 | Ensembl | 561 |
| 1.2308015 | NR_024490 | LOC100129387 | RefSeq_NR | 4139 |
| 1.2320027 | BM680929 | lincRNA-PLA2G12B | lincRNA | 520 |
| 1.2323435 | uc001usr.2 | CR592782 | UCSC_knowngene | 2567 |
| 1.2324487 | ENST00000450418 | AC023480.1 | Ensembl | 2315 |
| 1.232817 | uc001jzw.1 | AK126491 | UCSC_knowngene | 3842 |
| 1.2328666 | uc003ide.3 | CR610292 | UCSC_knowngene | 980 |
| 1.233335 | AF143322 |  | misc_RNA | 1415 |
| 1.2342848 | AI216374 | lincRNA-BANP-1 | lincRNA | 444 |
| 1.234633 | AI547046 | lincRNA-PRPS1-1 | lincRNA | 735 |
| 1.2351306 | CA391722 | lincRNA-FBXL17 | lincRNA | 663 |
| 1.2353874 | chr11:8303699-8319049+ | lincRNA-STK33 | lincRNA | 15351 |
| 1.2356566 | AK024576 |  | misc_RNA | 912 |
| 1.2374107 | BC034527 |  | misc_RNA | 1077 |
| 1.2392249 | BF328678 | lincRNA-ST14 | lincRNA | 259 |
| 1.2395022 | uc003fah.1 | BC015836 | UCSC_knowngene | 777 |
| 1.240257 | ENST00000444167 | AC006504.1 | Ensembl | 1329 |
| 1.2411584 | NR_026966 | LOC100130691 | RefSeq_NR | 5223 |
| 1.2415251 | ENST00000483942 | RP11-129K20.1 | Ensembl | 1338 |
| 1.2417179 | AA398139 | lincRNA-PARP11-2 | lincRNA | 489 |
| 1.2421767 | CR609715 |  | misc_RNA | 1695 |
| 1.244976 | ENST00000513032 | RP11-399D2.1 | Ensembl | 749 |
| 1.2458215 | ENST00000498256 | RP11-200A24.1 | Ensembl | 554 |
| 1.2463258 | uc001hor.1 | CR625980 | UCSC_knowngene | 1385 |
| 1.2475253 | uc003dpd.1 | AK055831 | UCSC_knowngene | 2693 |
| 1.247788 | uc002nwh.1 | DQ593116 | UCSC_knowngene | 118 |
| 1.2483866 | ENST00000437235 | AC007551.3 | Ensembl | 699 |
| 1.2499873 | CF129145 | lincRNA-LOC100506581-1 | lincRNA | 521 |
| 1.2500411 | DQ345519 | lincRNA-ZNF32-4 | lincRNA | 628 |
| 1.2502911 | NR_021491 | FLJ42709 | RefSeq_NR | 1184 |
| 1.2515143 | ENST00000515205 | RP11-539L10.3 | Ensembl | 422 |
| 1.2519442 | ENST00000430422 | RP11-314A15.1 | Ensembl | 1160 |
| 1.2537974 | ENST00000366365 | C17orf86 | Ensembl | 1028 |
| 1.2538749 | ENST00000505987 | HSP90AA4P | Ensembl | 2173 |
| 1.2543321 | ENST00000412485 | GS1-600G8.5 | Ensembl | 1497 |
| 1.2556614 | AA309472 | lincRNA-SHANK2 | lincRNA | 320 |
| 1.259398 | uc003vwb.1 | AK098095 | UCSC_knowngene | 2750 |
| 1.2596471 | uc003qwu.1 | AK055570 | UCSC_knowngene | 2282 |
| 1.2603008 | AK097341 | lincRNA-GPRC5C | lincRNA | 2227 |
| 1.2609176 | ENST00000515084 | AC007333.1 | Ensembl | 1972 |
| 1.2611427 | ENST00000470614 | RP11-69M1.1 | Ensembl | 315 |
| 1.2612212 | AK127488 |  | misc_RNA | 4343 |
| 1.2618522 | AK023763 |  | misc_RNA | 3568 |
| 1.2631383 | BF336285 | lincRNA-PRSS48 | lincRNA | 402 |
| 1.2651834 | ENST00000448271 | RP11-111F16.2 | Ensembl | 287 |
| 1.2652773 | ENST00000510062 | RP11-366M4.3 | Ensembl | 674 |
| 1.2684998 | ENST00000402768 | RP11-15H7.1 | Ensembl | 767 |
| 1.2694112 | CD242216 | lincRNA-ALX1-1 | lincRNA | 808 |
| 1.2702535 | AW631458 | lincRNA-ZFP36L2-1 | lincRNA | 414 |
| 1.2703669 | EC464921 | lincRNA-LRRC4C-5 | lincRNA | 89 |
| 1.2709938 | ENST00000444235 | RPL5P7 | Ensembl | 813 |
| 1.2712382 | ENST00000450559 | RP11-576N17.3 | Ensembl | 1004 |
| 1.2716551 | ENST00000506148 | RP11-556I14.1 | Ensembl | 472 |
| 1.2718875 | AW450413 | lincRNA-NFE2L3-2 | lincRNA | 431 |
| 1.2725475 | AK096059 |  | misc_RNA | 2036 |
| 1.2729795 | uc001uoi.2 | AK054845 | UCSC_knowngene | 2406 |
| 1.2733325 | T02908 | lincRNA-CD83-4 | lincRNA | 477 |
| 1.2736599 | BX115115 | lincRNA-ACTR2 | lincRNA | 649 |
| 1.2756828 | AK122825 | lincRNA-HMGB1-2 | lincRNA | 4200 |
| 1.2757134 | BC026095 |  | misc_RNA | 2212 |
| 1.2758393 | uc003vcj.1 | CR591034 | UCSC_knowngene | 2178 |
| 1.2777718 | ENST00000426309 | GS1-122H1.2 | Ensembl | 2631 |
| 1.2779358 | chr1:191998602-192012102- | lincRNA-FAM5C-3 | lincRNA | 13501 |
| 1.278412 | ENST00000515142 | RP11-83M16.3 | Ensembl | 405 |
| 1.2799314 | BX112397 | lincRNA-FAM183A-2 | lincRNA | 753 |
| 1.2805715 | chrX:118631438-118670334- | lincRNA-SLC25A5-2 | lincRNA | 38897 |
| 1.2813736 | ENST00000344851 | RP1-274L14.2 | Ensembl | 1979 |
| 1.2815521 | uc003brm.2 | BC036698 | UCSC_knowngene | 3252 |
| 1.2821241 | BU854312 | lincRNA-NHLRC3 | lincRNA | 762 |
| 1.2821514 | BF516116 | lincRNA-TTC8 | lincRNA | 513 |
| 1.2832681 | chr2:177245637-177252100- | lincRNA-MTX2-4 | lincRNA | 6464 |
| 1.2834963 | chr6:27248371-27266346- | lincRNA-PRSS16 | lincRNA | 17976 |
| 1.2910347 | ENST00000406279 | RP11-551A13.1 | Ensembl | 553 |
| 1.2933541 | AK130806 |  | misc_RNA | 1439 |
| 1.2936149 | BE837589 | lincRNA-EGLN3-4 | lincRNA | 540 |
| 1.293927 | AJ493605 | lincRNA-C3orf35 | lincRNA | 981 |
| 1.2940645 | ENST00000374922 | RP11-622K12.1 | Ensembl | 1529 |
| 1.2951856 | chr2:231539456-231550581- | lincRNA-SP100 | lincRNA | 11126 |
| 1.2960172 | AI028364 | lincRNA-PRLHR-2 | lincRNA | 449 |
| 1.2961373 | BG258490 | lincRNA-DYNC1H1 | lincRNA | 800 |
| 1.2962492 | chr4:120932275-120933293+ | lincRNA-MAD2L1-1 | lincRNA | 1019 |
| 1.2966306 | chr20:2142500-2155375- | lincRNA-STK35 | lincRNA | 12876 |
| 1.2984374 | AU252893 | lincRNA-TMEM194B | lincRNA | 700 |
| 1.2997051 | NR_003013 | SCARNA16 | RefSeq_NR | 187 |
| 1.2999328 | exon2438- | lincRNA-CEBPB-1 | lincRNA | 339 |
| 1.3000123 | ENST00000417351 | CTAGE7 | Ensembl | 2823 |
| 1.3004961 | ENST00000470444 | RP11-12N13.3 | Ensembl | 350 |
| 1.3016486 | AF086310 |  | misc_RNA | 578 |
| 1.3020222 | DB462076 | lincRNA-NMBR-4 | lincRNA | 481 |
| 1.3031544 | AK310679 | lincRNA-KCNK12-3 | lincRNA | 974 |
| 1.3032396 | BG897081 | lincRNA-CSNK1G3-1 | lincRNA | 416 |
| 1.3036631 | AK125450 |  | misc_RNA | 2509 |
| 1.305164 | ENST00000433433 | AC018730.3 | Ensembl | 3103 |
| 1.3064914 | uc001lcv.1 | DQ596646 | UCSC_knowngene | 2195 |
| 1.3066888 | DW455445 | lincRNA-C11orf74-2 | lincRNA | 174 |
| 1.3071713 | chr2:238206264-238224402+ | lincRNA-COL6A3-4 | lincRNA | 18139 |
| 1.3073664 | AX747492 | lincRNA-HFE2 | lincRNA | 4510 |
| 1.3081276 | BF515427 | lincRNA-IL12B-2 | lincRNA | 478 |
| 1.3105083 | NR_003272 | PSPC1 | RefSeq_NR | 1716 |
| 1.3111784 | uc003cqw.1 | AK096853 | UCSC_knowngene | 2353 |
| 1.3116841 | AY034104 | lincRNA-TACR1-2 | lincRNA | 306 |
| 1.3124584 | AK091382 |  | misc_RNA | 2368 |
| 1.3157811 | uc002yyn.1 | CR624487 | UCSC_knowngene | 1542 |
| 1.3161454 | AF143879 |  | misc_RNA | 581 |
| 1.3173981 | uc001igd.2 | BC046483 | UCSC_knowngene | 1204 |
| 1.3192712 | AW364455 | lincRNA-C16orf45 | lincRNA | 535 |
| 1.320998 | AK055958 |  | misc_RNA | 2041 |
| 1.3212837 | ENST00000427361 | AC020915.4 | Ensembl | 922 |
| 1.3235752 | BF745951 | lincRNA-PTPN20A | lincRNA | 443 |
| 1.3238982 | BE551891 | lincRNA-ANKRD28-2 | lincRNA | 539 |
| 1.3244163 | AA457263 | lincRNA-GPR61 | lincRNA | 451 |
| 1.3255404 | ENST00000501498 | AC108062.1 | Ensembl | 2882 |
| 1.3275954 | AK125138 | lincRNA-SH3BP5L | lincRNA | 3859 |
| 1.3276319 | AK293062 | lincRNA-GALNT1-2 | lincRNA | 2194 |
| 1.328457 | BX953928 | lincRNA-DOK2 | lincRNA | 442 |
| 1.3312985 | BM990693 | lincRNA-RTTN-2 | lincRNA | 599 |
| 1.3313887 | DW421906 | lincRNA-SIAH3 | lincRNA | 296 |
| 1.3317452 | AK316271 | lincRNA-SUCLA2-1 | lincRNA | 1801 |
| 1.3330542 | BC052961 |  | NRED | 4283 |
| 1.3337228 | BG273037 | lincRNA-CDH5-2 | lincRNA | 387 |
| 1.3350451 | BE091553 | lincRNA-LRRIQ3-2 | lincRNA | 192 |
| 1.3350476 | AK128058 | lincRNA-ZNF532 | lincRNA | 6183 |
| 1.3359532 | chr16:65796674-65812074+ | lincRNA-CDH5-4 | lincRNA | 15401 |
| 1.3364574 | NR_028408 | LOC400027 | RefSeq_NR | 2202 |
| 1.3368005 | ENST00000397097 | ZNF732 | Ensembl | 1032 |
| 1.3375717 | BC030711 | lincRNA-PROKR1 | lincRNA | 1708 |
| 1.3386744 | ENST00000498507 | RP4-612B18.1 | Ensembl | 350 |
| 1.3390276 | chr2:48624186-48643070+ | lincRNA-KLRAQ1-2 | lincRNA | 18885 |
| 1.3402811 | CR590107 | lincRNA-NUDT15-2 | lincRNA | 950 |
| 1.3403061 | ENST00000490357 | RP11-10O22.1 | Ensembl | 1834 |
| 1.3417228 | ENST00000432307 | RP11-111F16.1 | Ensembl | 1813 |
| 1.3447444 | ENST00000420287 | RP11-505F3.4 | Ensembl | 408 |
| 1.3462869 | DB319310 | lincRNA-CDK17-1 | lincRNA | 524 |
| 1.3464768 | chr14:75087240-75099311+ | lincRNA-KIAA0317 | lincRNA | 12072 |
| 1.3469234 | uc001wkc.1 | AX746599 | UCSC_knowngene | 2524 |
| 1.348329 | AK127645 | lincRNA-TCF4-3 | lincRNA | 2558 |
| 1.3487601 | ENST00000340195 | AC004079.4 | Ensembl | 672 |
| 1.3489439 | AL137472 |  | misc_RNA | 2679 |
| 1.349252 | chr1:26937352-26948553- | lincRNA-RPS6KA1 | lincRNA | 11202 |
| 1.3495475 | uc001iuc.2 | BC043365 | UCSC_knowngene | 2078 |
| 1.3538083 | NR_015360 | FLJ33630 | RefSeq_NR | 2977 |
| 1.3543811 | AA663642 | lincRNA-ADRB3-2 | lincRNA | 467 |
| 1.3561128 | ENST00000454702 | RP13-211P9.1 | Ensembl | 592 |
| 1.3576316 | DB344658 | lincRNA-SH2D4A-1 | lincRNA | 568 |
| 1.3598643 | ENST00000455793 | RP11-3D23.1 | Ensembl | 1077 |
| 1.3603927 | AK022980 |  | misc_RNA | 2377 |
| 1.3618406 | AA594021 | lincRNA-GNA13 | lincRNA | 359 |
| 1.3640691 | chr8:30192504-30200352+ | lincRNA-RBPMS-2 | lincRNA | 7849 |
| 1.3641883 | ENST00000507299 | CTD-2325B11.1 | Ensembl | 430 |
| 1.3649753 | CD516039 | lincRNA-THBS4 | lincRNA | 510 |
| 1.3661082 | ENST00000452381 | AC064834.2 | Ensembl | 850 |
| 1.368988 | ENST00000428294 | RP3-449O17.1 | Ensembl | 700 |
| 1.3694397 | AI857708 | lincRNA-PDHA2-3 | lincRNA | 125 |
| 1.3696166 | ENST00000405924 | RP3-451B15.3 | Ensembl | 140 |
| 1.3699122 | BI048312 | lincRNA-WNT9A | lincRNA | 393 |
| 1.3699165 | ENST00000514050 | RP11-689K5.3 | Ensembl | 472 |
| 1.3710554 | AK090477 |  | misc_RNA | 4801 |
| 1.371423 | AL080082 |  | NRED | 1026 |
| 1.371663 | NR_024149 | MEG8 | RefSeq_NR | 501 |
| 1.3750685 | AK096322 | lincRNA-FBRS | lincRNA | 2890 |
| 1.3759901 | AW086055 | lincRNA-PRRC2C | lincRNA | 378 |
| 1.3763439 | chr1:98805012-98815587+ | lincRNA-SNX7-2 | lincRNA | 10576 |
| 1.3764226 | DA687098 | lincRNA-TRPC4-2 | lincRNA | 544 |
| 1.3775739 | chr18:71300370-71356620- | lincRNA-NETO1-3 | lincRNA | 56251 |
| 1.3781839 | AL832443 | lincRNA-BNC2-2 | lincRNA | 2382 |
| 1.3789425 | chr4:108493109-108517451- | lincRNA-DKK2 | lincRNA | 24343 |
| 1.3803791 | AK097798 | lincRNA-ZNF676 | lincRNA | 2091 |
| 1.3815881 | ENST00000377977 | AC074391.1 | Ensembl | 1266 |
| 1.3840297 | ENST00000506460 | RP11-452C8.1 | Ensembl | 741 |
| 1.3898496 | ENST00000427188 | C21orf84 | Ensembl | 967 |
| 1.3901004 | chrX:128828944-128841419+ | lincRNA-XPNPEP2-2 | lincRNA | 12476 |
| 1.3908578 | AA604748 | lincRNA-OXR1-1 | lincRNA | 262 |
| 1.395345 | chr2:113359154-113374304- | lincRNA-CHCHD5-1 | lincRNA | 15151 |
| 1.3963954 | CF594357 | lincRNA-RASSF9-1 | lincRNA | 801 |
| 1.3968464 | NR_024052 | HCG18 | RefSeq_NR | 6814 |
| 1.3968821 | ENST00000392268 | AC008507.1 | Ensembl | 756 |
| 1.3971558 | BM976593 | lincRNA-CDS2 | lincRNA | 719 |
| 1.3977094 | CV370110 | lincRNA-CDH17 | lincRNA | 511 |
| 1.3997489 | AK090904 |  | RNAdb | 2547 |
| 1.4034166 | AX748201 | lincRNA-MARCH3 | lincRNA | 2633 |
| 1.4034921 | BC028019 | lincRNA-C9orf57 | lincRNA | 3343 |
| 1.4053107 | chr9:81993205-81999034+ | lincRNA-TLE4-1 | lincRNA | 5830 |
| 1.405441 | uc010ayj.1 | HBT8 | UCSC_knowngene | 4100 |
| 1.4071442 | ENST00000413128 | RP11-420K5.1 | Ensembl | 774 |
| 1.4076635 | ENST00000441338 | RP11-278H7.4 | Ensembl | 468 |
| 1.4086686 | ENST00000430261 | RP4-551E13.2 | Ensembl | 1569 |
| 1.4089387 | DB296184 | lincRNA-GRM7-1 | lincRNA | 545 |
| 1.411312 | BX096636 | lincRNA-STARD8 | lincRNA | 670 |
| 1.4127369 | BM674971 | lincRNA-GRIK2 | lincRNA | 480 |
| 1.4153277 | ENST00000514008 | RP11-2H3.2 | Ensembl | 125 |
| 1.4206736 | AL832122 |  | misc_RNA | 5572 |
| 1.4237028 | EC557833 | lincRNA-SLC6A15 | lincRNA | 100 |
| 1.4263763 | uc010bol.1 | AK307134 | UCSC_knowngene | 2009 |
| 1.4269641 | ENST00000424980 | RP11-375O18.2 | Ensembl | 328 |
| 1.4328323 | BU626341 | lincRNA-RDX-2 | lincRNA | 692 |
| 1.4328387 | uc001jxd.1 | AK023445 | UCSC_knowngene | 1860 |
| 1.4345109 | ENST00000453103 | AC073046.4 | Ensembl | 601 |
| 1.4348128 | BI029614 | lincRNA-ERF | lincRNA | 493 |
| 1.4355335 | ENST00000476607 | RP11-572M11.2 | Ensembl | 425 |
| 1.4385709 | BC037864 | lincRNA-CD247 | lincRNA | 4577 |
| 1.4386604 | uc001hje.2 | CR624623 | UCSC_knowngene | 838 |
| 1.4430371 | AA164863 | lincRNA-TMEM9 | lincRNA | 472 |
| 1.4442455 | chr10:74002486-74020610+ | lincRNA-DDIT4-1 | lincRNA | 18125 |
| 1.4494705 | NR_024284 | LOC220930 | RefSeq_NR | 2232 |
| 1.4506109 | AK289744 | lincRNA-SUN2 | lincRNA | 2725 |
| 1.4561359 | DA922283 | lincRNA-TH | lincRNA | 568 |
| 1.458194 | AW675401 | lincRNA-GFRA2-4 | lincRNA | 120 |
| 1.4587779 | chr5:100383976-100436626- | lincRNA-ST8SIA4-2 | lincRNA | 52651 |
| 1.4588949 | AK299029 | lincRNA-RAPGEF3 | lincRNA | 2776 |
| 1.4603099 | BX952962 | lincRNA-OBFC2A-2 | lincRNA | 428 |
| 1.462094 | ENST00000393071 | AC013474.3 | Ensembl | 732 |
| 1.469811 | AK124977 | lincRNA-UNKL-2 | lincRNA | 2638 |
| 1.4713416 | chr6:91387079-91405854+ | lincRNA-EPHA7-3 | lincRNA | 18776 |
| 1.475389 | ENST00000397222 | RP4-774G10.1 | Ensembl | 778 |
| 1.476077 | CN274459 | lincRNA-OTX2 | lincRNA | 631 |
| 1.4778231 | chr1:29096302-29111760- | lincRNA-YTHDF2 | lincRNA | 15459 |
| 1.4797588 | chr15:70785696-70790995- | lincRNA-TLE3-1 | lincRNA | 5300 |
| 1.4810258 | uc002aug.2 | BC034424 | UCSC_knowngene | 3146 |
| 1.481986 | AF191495 | lincRNA-F11R | lincRNA | 2187 |
| 1.4845069 | NR_026757 | LOC729082 | RefSeq_NR | 1894 |
| 1.4870534 | ENST00000366408 | RP11-375F2.2 | Ensembl | 1129 |
| 1.4890482 | DA919949 | lincRNA-C14orf101 | lincRNA | 566 |
| 1.4941834 | ENST00000502314 | CCT5P1 | Ensembl | 1618 |
| 1.4964991 | AK026910 | lincRNA-CROCC | lincRNA | 1883 |
| 1.497367 | AA431270 | lincRNA-PARP9 | lincRNA | 408 |
| 1.4977925 | chr2:237543636-237554786+ | lincRNA-COPS8-1 | lincRNA | 11151 |
| 1.5014761 | ENST00000506596 | ANKDD1B | Ensembl | 1815 |
| 1.503775 | CD300628 | lincRNA-CCND2-2 | lincRNA | 927 |
| 1.509108 | BM912149 | lincRNA-RFPL4B-2 | lincRNA | 1116 |
| 1.5113191 | AW182066 | lincRNA-LOC100506581-4 | lincRNA | 277 |
| 1.5158687 | ENST00000464731 | RP11-451G4.4 | Ensembl | 381 |
| 1.5169577 | BF795336 | lincRNA-FNBP1 | lincRNA | 864 |
| 1.5234925 | BM704421 | lincRNA-NDUFA10 | lincRNA | 436 |
| 1.5246455 | uc010wkm.1 | CR624169 | UCSC_knowngene | 416 |
| 1.5257525 | ENST00000403367 | RP1-72A23.1 | Ensembl | 482 |
| 1.52595 | AB074181 | lincRNA-CDRT4 | lincRNA | 2838 |
| 1.5327133 | BC002831 |  | misc_RNA | 1013 |
| 1.5328723 | CK905913 | lincRNA-TTC17-2 | lincRNA | 587 |
| 1.5332996 | uc002uaw.2 | DKFZp686H10114 | UCSC_knowngene | 2528 |
| 1.5353169 | NR_026962 | LOC284900 | RefSeq_NR | 963 |
| 1.5354322 | NR_027451 | LOC647979 | RefSeq_NR | 5378 |
| 1.5423095 | NR_027058 | C17orf86 | RefSeq_NR | 2183 |
| 1.5456467 | BX648197 |  | misc_RNA | 5122 |
| 1.5473304 | uc010qus.1 | EU250747 | UCSC_knowngene | 2229 |
| 1.5479127 | ENST00000431464 | RP11-308D16.4 | Ensembl | 646 |
| 1.5489613 | AK123196 |  | RNAdb | 1909 |
| 1.5496915 | chr2:204428779-204440326- | lincRNA-RAPH1-2 | lincRNA | 11548 |
| 1.5513474 | uc001eiw.1 | CR936796 | UCSC_knowngene | 4096 |
| 1.5555784 | uc003njk.2 | BC035101 | UCSC_knowngene | 2763 |
| 1.5631873 | ENST00000487840 | RP11-167H9.4 | Ensembl | 372 |
| 1.5652481 | uc001yfu.2 | BC038465 | UCSC_knowngene | 1114 |
| 1.5662861 | BC009210 | lincRNA-KIAA1199 | lincRNA | 2959 |
| 1.568954 | uc003jei.2 | BX648541 | UCSC_knowngene | 3239 |
| 1.572952 | NR_003138 | SNHG10 | RefSeq_NR | 1972 |
| 1.5737646 | ENST00000508179 | CTD-2001E22.2 | Ensembl | 1799 |
| 1.5743323 | ENST00000427150 | AC097721.1 | Ensembl | 1253 |
| 1.583676 | uc002rdf.2 | BC068572 | UCSC_knowngene | 3008 |
| 1.5845319 | chr9:126100254-126117679- | lincRNA-STRBP | lincRNA | 17426 |
| 1.5864203 | NR_026765 | C8orf75 | RefSeq_NR | 1413 |
| 1.5876596 | AK095707 |  | misc_RNA | 2984 |
| 1.5890373 | CR599308 | lincRNA-AEBP2-2 | lincRNA | 2138 |
| 1.5930238 | DA438209 | lincRNA-AHR-2 | lincRNA | 547 |
| 1.6004837 | BC070168 | lincRNA-TSPAN8 | lincRNA | 1543 |
| 1.6010891 | AK126698 |  | RNAdb | 3826 |
| 1.6131337 | chr19:22497397-22499384+ | lincRNA-ZNF98 | lincRNA | 1988 |
| 1.6159289 | DT216591 | lincRNA-RAB28-4 | lincRNA | 636 |
| 1.6172674 | AX747038 | lincRNA-BANK1-2 | lincRNA | 3544 |
| 1.6190381 | AK093982 |  | misc_RNA | 2269 |
| 1.6205719 | AA405014 | lincRNA-TMTC2 | lincRNA | 267 |
| 1.6232648 | uc003imv.2 | DKFZp434I0714 | UCSC_knowngene | 2999 |
| 1.6271993 | ENST00000455031 | RP4-756H11.1 | Ensembl | 850 |
| 1.6338032 | NR_024279 | FLJ37453 | RefSeq_NR | 2732 |
| 1.6350756 | AV730760 | lincRNA-LPAR3-2 | lincRNA | 603 |
| 1.6357034 | ENST00000433591 | RP11-423O2.2 | Ensembl | 593 |
| 1.6476794 | uc001gjk.2 | GAS5 | UCSC_knowngene | 1695 |
| 1.6543258 | chr8:129178878-129187868- | lincRNA-MYC-1 | lincRNA | 8991 |
| 1.661007 | CR616931 | lincRNA-HEY1 | lincRNA | 728 |
| 1.6614415 | exon3668+ | lincRNA-DACT2-3 | lincRNA | 101 |
| 1.6720078 | EB388332 | lincRNA-MYOC | lincRNA | 699 |
| 1.6771146 | chr2:68206296-68223571+ | lincRNA-C1D-1 | lincRNA | 17276 |
| 1.683034 | ENST00000440088 | AC090421.1 | Ensembl | 2029 |
| 1.6941009 | CB112975 | lincRNA-UBL3-1 | lincRNA | 379 |
| 1.6957312 | BE080482 | lincRNA-CTSC | lincRNA | 506 |
| 1.7004706 | uc003qet.1 | AK055166 | UCSC_knowngene | 2069 |
| 1.7088528 | BU662692 | lincRNA-ADAMTS1 | lincRNA | 685 |
| 1.7093008 | uc003che.2 | BC040563 | UCSC_knowngene | 3263 |
| 1.7104421 | ENST00000420836 | AC005294.1 | Ensembl | 1083 |
| 1.7132507 | BG219312 | lincRNA-SOCS5-1 | lincRNA | 371 |
| 1.7206725 | chr16:74903295-74907270- | lincRNA-FA2H-2 | lincRNA | 3976 |
| 1.7224743 | BC031073 | lincRNA-EFCAB4B | lincRNA | 2445 |
| 1.7279991 | chr6:70134290-70139867- | lincRNA-BAI3 | lincRNA | 5578 |
| 1.7291229 | uc010fcy.1 | FLJ16124 | UCSC_knowngene | 1113 |
| 1.7298423 | BU618532 | lincRNA-RBFOX1 | lincRNA | 723 |
| 1.7407977 | NR_026994 | LOC340094 | RefSeq_NR | 1763 |
| 1.7515768 | NR_026905 | C17orf69 | RefSeq_NR | 2477 |
| 1.7584734 | CR595169 |  | RNAdb | 1493 |
| 1.7823785 | NR_027064 | PLAC2 | RefSeq_NR | 3693 |
| 1.7893356 | BC034819 |  | NRED | 2882 |
| 1.796075 | CN273898 | lincRNA-TMCO5A-2 | lincRNA | 807 |
| 1.8017935 | ENST00000447834 | RP11-141M3.3 | Ensembl | 2134 |
| 1.804854 | ENST00000452840 | AC007092.1 | Ensembl | 4077 |
| 1.8069325 | NR_026543 | C21orf88 | RefSeq_NR | 1365 |
| 1.8105057 | AK022299 |  | misc_RNA | 1880 |
| 1.8126284 | chr13:32382650-32393700- | lincRNA-RXFP2-2 | lincRNA | 11051 |
| 1.8147338 | AK000839 |  | misc_RNA | 1951 |
| 1.8361348 | uc003tvu.2 | BC017910 | UCSC_knowngene | 487 |
| 1.858495 | D79689 | lincRNA-FOXN3 | lincRNA | 266 |
| 1.8601057 | uc003srb.1 | CR609701 | UCSC_knowngene | 3988 |
| 1.8601925 | ENST00000433480 | RP11-305D15.2 | Ensembl | 829 |
| 1.8690318 | chr12:89622944-89647544+ | lincRNA-DUSP6-4 | lincRNA | 24601 |
| 1.8711653 | ENST00000433544 | RP11-488P3.1 | Ensembl | 1671 |
| 1.8799348 | CF129483 | lincRNA-SMNDC1-3 | lincRNA | 602 |
| 1.8991617 | ENST00000485242 | MT-ND3 | Ensembl | 339 |
| 1.95411 | NR_033315 | BDNFOS | RefSeq_NR | 1437 |
| 1.9560748 | CT001622 | lincRNA-IFT57 | lincRNA | 865 |
| 1.976856 | DB349331 | lincRNA-CTNND1 | lincRNA | 556 |
| 1.9849933 | BU853353 | lincRNA-NT5C1B-2 | lincRNA | 766 |
| **2.0811209** | **BU729004** | **lincRNA-MAP3K1-2** | **lincRNA** | **599** |
| **2.098126** | **chr3:140718187-140723779+** | **lincRNA-SPSB4-2** | **lincRNA** | **5593** |
| **2.1067045** | **AW572416** | **lincRNA-WNT4** | **lincRNA** | **350** |
| **2.1135804** | **CD522236** | **lincRNA-PDHX** | **lincRNA** | **878** |
| **2.1265522** | **chr1:86683137-86705987-** | **lincRNA-COL24A1** | **lincRNA** | **22851** |
| **2.1352023** | **BX093283** | **lincRNA-KLF5-1** | **lincRNA** | **508** |
| **2.1368957** | **NR_015422** | **LOC149134** | **RefSeq_NR** | **1123** |
| **2.2191269** | **chr5:3828075-3841525+** | **lincRNA-ADAMTS16-1** | **lincRNA** | **13451** |
| **2.2698492** | **BP264199** | **lincRNA-GCNT3** | **lincRNA** | **578** |
| **2.4284634** | **chr15:88215746-88230771+** | **lincRNA-NTRK3** | **lincRNA** | **15026** |
| **2.6326666** | **ENST00000437593** | **RP11-500G22.2** | **Ensembl** | **951** |

The bold indicates 11 differently expressed lncRNAs (≥ 2-fold, *P* < 0.05) between six PDAC samples and paired nontumor samples.

**Table S6** 1133 Enhancer lncRNAs detected in PDAC

| **Fold change** | **Seqname** | **GeneSymbol** | **Source** | **RNA**  **length** |
| --- | --- | --- | --- | --- |
| 0.2413095 | uc009wkz.1 | FCGR1C | UCSC_knowngene | 566 |
| 0.285603 | NR_027484 | FCGR1C | RefSeq_NR | 1338 |
| 0.3515558 | ENST00000455038 | RP11-335E6.2 | Ensembl | 823 |
| 0.3517753 | ENST00000400141 | TMED11P | Ensembl | 657 |
| 0.4040882 | uc003qiq.1 | AK123801 | UCSC_knowngene | 1690 |
| 0.4186623 | ENST00000447613 | RP11-327I22.5 | Ensembl | 957 |
| 0.4218133 | uc001daw.1 | BC040309 | UCSC_knowngene | 1093 |
| 0.4258089 | uc001yjz.1 | AK094562 | UCSC_knowngene | 2638 |
| 0.4382946 | uc004fnm.2 | U00684 | UCSC_knowngene | 552 |
| 0.4635364 | ENST00000417884 | RP3-340N1.2 | Ensembl | 1083 |
| 0.4657924 | ENST00000443471 | RP3-393K13.1 | Ensembl | 351 |
| 0.4749159 | ENST00000373171 | RP11-552E20.3 | Ensembl | 554 |
| 0.4764197 | uc003sze.1 | BC034444 | UCSC_knowngene | 728 |
| 0.4812086 | ENST00000455051 | RP11-341A22.2 | Ensembl | 588 |
| 0.4834798 | ENST00000422894 | RP5-894D12.3 | Ensembl | 318 |
| 0.4909149 | ENST00000418379 | RP11-15K3.1 | Ensembl | 981 |
| 0.4985216 | ENST00000451118 | WI2-85898F10.1 | Ensembl | 805 |
| 0.4989467 | uc004bcw.2 | BC039487 | UCSC_knowngene | 1005 |
| 0.4995746 | ENST00000417800 | RP11-240M16.1 | Ensembl | 381 |
| 0.5229052 | ENST00000447206 | RP5-968J1.1 | Ensembl | 346 |
| 0.5252591 | uc004bxt.1 | AX747401 | UCSC_knowngene | 1772 |
| 0.5359379 | ENST00000446337 | RP11-178D16.1 | Ensembl | 415 |
| 0.5435479 | ENST00000435867 | RP13-348B13.2 | Ensembl | 308 |
| 0.5472099 | ENST00000437290 | AC010105.1 | Ensembl | 687 |
| 0.5494129 | ENST00000450850 | RP11-555H7.2 | Ensembl | 390 |
| 0.5529817 | ENST00000450247 | CTA-109P11.1 | Ensembl | 475 |
| 0.55795 | ENST00000454625 | GS1-421I3.2 | Ensembl | 772 |
| 0.5623849 | NR_033557 | LOC100422737 | RefSeq_NR | 1387 |
| 0.5665633 | ENST00000446811 | RP11-503C24.4 | Ensembl | 2246 |
| 0.5711316 | ENST00000443523 | RP11-556E13.1 | Ensembl | 369 |
| 0.5712125 | ENST00000422914 | RP11-733O18.1 | Ensembl | 860 |
| 0.5860294 | ENST00000423943 | RP11-48O20.4 | Ensembl | 1113 |
| 0.5883583 | uc004bxu.2 | BC094873 | UCSC_knowngene | 1213 |
| 0.5966429 | ENST00000442072 | RP11-440G5.2 | Ensembl | 474 |
| 0.6027164 | ENST00000445339 | RP11-552D4.1 | Ensembl | 654 |
| 0.6104476 | ENST00000414308 | RP11-342D11.3 | Ensembl | 552 |
| 0.6106844 | uc004bcx.2 | BC017988 | UCSC_knowngene | 1311 |
| 0.617858 | ENST00000425271 | RP11-479J7.2 | Ensembl | 423 |
| 0.6195918 | ENST00000437035 | RP5-1043L13.1 | Ensembl | 499 |
| 0.6217775 | ENST00000417930 | AC092580.4 | Ensembl | 243 |
| 0.6302939 | ENST00000450248 | TUG1 | Ensembl | 6477 |
| 0.6312684 | NR_026827 | LOC84856 | RefSeq_NR | 2322 |
| 0.631329 | ENST00000444114 | RP5-1172A22.1 | Ensembl | 637 |
| 0.6332713 | ENST00000418518 | RP11-771D21.2 | Ensembl | 518 |
| 0.6382168 | ENST00000455247 | RP5-1109J22.3 | Ensembl | 354 |
| 0.6386082 | BC105019 |  | NRED | 339 |
| 0.6397122 | uc002yky.3 | C21orf131 | UCSC_knowngene | 2124 |
| 0.6411479 | ENST00000422844 | RP11-136B18.2 | Ensembl | 197 |
| 0.6413451 | ENST00000438619 | RP11-93N20.1 | Ensembl | 339 |
| 0.6431902 | NR_001564 | XIST | RefSeq_NR | 19271 |
| 0.6436644 | ENST00000429980 | RP11-143M1.2 | Ensembl | 394 |
| 0.6438325 | uc004aul.1 | DM004128 | UCSC_knowngene | 103 |
| 0.6475766 | ENST00000453837 | RP11-84A19.2 | Ensembl | 2257 |
| 0.64817 | ENST00000448572 | RP4-705F19.2 | Ensembl | 454 |
| 0.6491221 | ENST00000456887 | RP11-343D24.2 | Ensembl | 315 |
| 0.6495232 | ENST00000442182 | RP11-39H13.1 | Ensembl | 511 |
| 0.6529955 | ENST00000454367 | AC007250.4 | Ensembl | 813 |
| 0.6531335 | NR_027349 | MIR17HG | RefSeq_NR | 927 |
| 0.6563453 | NR_026795 | NCRNA00202 | RefSeq_NR | 3650 |
| 0.6583232 | ENST00000421788 | RP4-697P8.2 | Ensembl | 268 |
| 0.6605243 | ENST00000434356 | RP11-347L18.1 | Ensembl | 466 |
| 0.6607425 | ENST00000435554 | RP11-476H20.1 | Ensembl | 440 |
| 0.6619103 | ENST00000422763 | RP11-346D6.6 | Ensembl | 570 |
| 0.6625175 | ENST00000447453 | AC078941.1 | Ensembl | 750 |
| 0.666153 | ENST00000413088 | RP1-266L20.2 | Ensembl | 403 |
| 0.6663623 | ENST00000391359 | RP1-122K4.3 | Ensembl | 544 |
| 0.6684081 | ENST00000446404 | AP000402.3 | Ensembl | 540 |
| 0.6754323 | ENST00000451618 | AP001476.2 | Ensembl | 2198 |
| 0.6793788 | ENST00000433303 | AL035610.2 | Ensembl | 276 |
| 0.6801294 | ENST00000440633 | RP11-252M21.1 | Ensembl | 411 |
| 0.6815907 | ENST00000449812 | RP11-145M4.3 | Ensembl | 623 |
| 0.6816808 | ENST00000449746 | AF127577.12 | Ensembl | 446 |
| 0.6847218 | ENST00000420762 | RP11-95P13.1 | Ensembl | 451 |
| 0.686784 | ENST00000454160 | RP11-54A22.2 | Ensembl | 599 |
| 0.6868131 | uc004dgl.1 | BC015977 | UCSC_knowngene | 271 |
| 0.6869359 | ENST00000454470 | RP11-67B16.1 | Ensembl | 363 |
| 0.687835 | NR_027131 | CXorf42 | RefSeq_NR | 2434 |
| 0.6889591 | uc001gva.3 | BC040869 | UCSC_knowngene | 1784 |
| 0.689829 | uc002yug.2 | AK025786 | UCSC_knowngene | 2525 |
| 0.6904648 | NR_026883 | HSPC072 | RefSeq_NR | 1574 |
| 0.6916334 | uc001alp.1 | AK125078 | UCSC_knowngene | 847 |
| 0.6916916 | ENST00000446887 | RP1-111C20.3 | Ensembl | 331 |
| 0.6936525 | ENST00000429328 | RP1-18D14.4 | Ensembl | 479 |
| 0.6939833 | ENST00000443888 | RP11-442O18.3 | Ensembl | 200 |
| 0.6951817 | uc002sdy.1 | AK123766 | UCSC_knowngene | 2100 |
| 0.6969815 | ENST00000456100 | AL163953.3 | Ensembl | 562 |
| 0.7020336 | uc001dfx.2 | BC041341 | UCSC_knowngene | 3870 |
| 0.7025163 | ENST00000437113 | RP11-214F16.4 | Ensembl | 652 |
| 0.7043796 | ENST00000435828 | RP1-158P9.1 | Ensembl | 731 |
| 0.7047187 | ENST00000441923 | ANTXRL | Ensembl | 634 |
| 0.7077039 | ENST00000449071 | RP11-128B16.3 | Ensembl | 422 |
| 0.7080071 | ENST00000430908 | AC005152.3 | Ensembl | 381 |
| 0.7080622 | ENST00000437648 | RP11-146I2.1 | Ensembl | 1017 |
| 0.709328 | uc002rnf.1 | AK123934 | UCSC_knowngene | 679 |
| 0.7104247 | ENST00000417311 | RP11-84P7.2 | Ensembl | 504 |
| 0.710453 | ENST00000445442 | RP11-503C24.6 | Ensembl | 445 |
| 0.7107533 | uc003pce.2 | AK056584 | UCSC_knowngene | 3102 |
| 0.7114794 | ENST00000437748 | RP11-321C24.1 | Ensembl | 240 |
| 0.7154519 | ENST00000449841 | RP5-1069C8.1 | Ensembl | 484 |
| 0.7159219 | ENST00000425153 | RP11-205K6.2 | Ensembl | 339 |
| 0.7164759 | uc004dbt.2 | BC024027 | UCSC_knowngene | 1879 |
| 0.7172021 | ENST00000423122 | RP11-65J3.1 | Ensembl | 545 |
| 0.7195751 | uc003wml.1 | AX746648 | UCSC_knowngene | 2698 |
| 0.7203455 | ENST00000421237 | RP11-439L18.2 | Ensembl | 402 |
| 0.7207866 | ENST00000456032 | RP11-127L21.1 | Ensembl | 256 |
| 0.7216328 | ENST00000450059 | SIGLECP3 | Ensembl | 271 |
| 0.7232767 | AK127523 |  | misc_RNA | 3301 |
| 0.726533 | ENST00000419300 | RP11-492E3.1 | Ensembl | 1959 |
| 0.7280698 | ENST00000437515 | AL358175.1 | Ensembl | 2504 |
| 0.7281358 | ENST00000417715 | AC073928.2 | Ensembl | 444 |
| 0.7296404 | uc002sdx.2 | BC040863 | UCSC_knowngene | 681 |
| 0.7300399 | ENST00000439823 | AC002480.4 | Ensembl | 557 |
| 0.7302012 | uc003sio.1 | AK024243 | UCSC_knowngene | 4411 |
| 0.7320112 | ENST00000435531 | RP11-363N22.2 | Ensembl | 407 |
| 0.7321803 | ENST00000412424 | AC009411.1 | Ensembl | 548 |
| 0.7323109 | uc001fuu.2 | AF088076 | UCSC_knowngene | 423 |
| 0.7339444 | ENST00000428753 | RP11-343J3.2 | Ensembl | 503 |
| 0.7349691 | ENST00000442197 | AL132709.9 | Ensembl | 625 |
| 0.7355526 | ENST00000443445 | RP1-223B1.1 | Ensembl | 789 |
| 0.7363678 | ENST00000432395 | RP5-943J3.1 | Ensembl | 509 |
| 0.737935 | ENST00000419979 | RP3-525N10.2 | Ensembl | 534 |
| 0.7386001 | ENST00000480386 | RP11-357H14.4 | Ensembl | 566 |
| 0.7389719 | ENST00000445544 | HCG16 | Ensembl | 317 |
| 0.7404608 | uc002yli.1 | AK124194 | UCSC_knowngene | 2908 |
| 0.7409368 | NR_024506 | LOC646982 | RefSeq_NR | 3834 |
| 0.7416781 | uc010nxt.1 | AK125248 | UCSC_knowngene | 2373 |
| 0.7433424 | uc002xts.1 | BX648826 | UCSC_knowngene | 4087 |
| 0.7435186 | ENST00000439844 | RP3-503A6.2 | Ensembl | 400 |
| 0.7440471 | NR_029408 | LOC439994 | RefSeq_NR | 1179 |
| 0.7444142 | ENST00000420469 | RP11-978I15.9 | Ensembl | 1638 |
| 0.7445132 | ENST00000414071 | RP11-143G3.1 | Ensembl | 378 |
| 0.7459759 | uc003adh.2 | BC015159 | UCSC_knowngene | 687 |
| 0.7459974 | ENST00000434552 | RP11-490O24.2 | Ensembl | 631 |
| 0.7464164 | ENST00000411802 | RP11-305E6.1 | Ensembl | 514 |
| 0.7479741 | ENST00000421737 | RP1-124C6.1 | Ensembl | 524 |
| 0.7492714 | ENST00000418471 | RP1-212P9.3 | Ensembl | 1085 |
| 0.7495624 | ENST00000424662 | RP4-614C15.2 | Ensembl | 593 |
| 0.7501797 | ENST00000420876 | RP11-511I2.2 | Ensembl | 483 |
| 0.7508615 | NR_027356 | LOC645166 | RefSeq_NR | 592 |
| 0.7536613 | ENST00000445814 | XIST | Ensembl | 437 |
| 0.756272 | ENST00000433429 | AC096570.1 | Ensembl | 440 |
| 0.7566905 | ENST00000444464 | AC084809.3 | Ensembl | 512 |
| 0.7595554 | ENST00000441790 | RP11-392O17.1 | Ensembl | 811 |
| 0.7606738 | ENST00000452923 | RP11-397D12.4 | Ensembl | 243 |
| 0.7612422 | ENST00000457457 | AC016735.1 | Ensembl | 400 |
| 0.7623787 | NR_026884 | HSPC072 | RefSeq_NR | 1397 |
| 0.7628231 | uc004bya.1 | BC037833 | UCSC_knowngene | 2449 |
| 0.7630611 | ENST00000434849 | XXyac-R12DG2.1 | Ensembl | 2372 |
| 0.7647403 | ENST00000450551 | AC068490.2 | Ensembl | 636 |
| 0.7650921 | ENST00000428006 | RP11-536O18.1 | Ensembl | 477 |
| 0.7699326 | ENST00000427276 | RP11-359N11.2 | Ensembl | 3152 |
| 0.7704618 | ENST00000429250 | RP11-90J7.3 | Ensembl | 607 |
| 0.7711078 | ENST00000440104 | RP11-103C3.1 | Ensembl | 807 |
| 0.771158 | ENST00000451910 | RP11-344J7.2 | Ensembl | 394 |
| 0.7712551 | ENST00000449605 | RP11-147O5.1 | Ensembl | 7619 |
| 0.7718342 | ENST00000420049 | RP11-393K12.2 | Ensembl | 436 |
| 0.7722321 | ENST00000451749 | AC073409.1 | Ensembl | 269 |
| 0.7735224 | uc001hpe.1 | AK124056 | UCSC_knowngene | 1530 |
| 0.7742101 | ENST00000421629 | RP11-184A2.3 | Ensembl | 758 |
| 0.774346 | ENST00000450270 | RP11-307B23.1 | Ensembl | 441 |
| 0.7750008 | ENST00000423681 | RP11-171A24.2 | Ensembl | 648 |
| 0.7763094 | ENST00000427717 | RP11-51B13.2 | Ensembl | 431 |
| 0.7776005 | ENST00000418943 | RP11-473M10.3 | Ensembl | 776 |
| 0.7779518 | ENST00000435485 | RP11-308N19.1 | Ensembl | 1686 |
| 0.7782571 | uc001czn.2 | BC042048 | UCSC_knowngene | 526 |
| 0.7792462 | ENST00000442358 | RP11-404O13.1 | Ensembl | 649 |
| 0.7795273 | ENST00000446476 | RP5-826L7.1 | Ensembl | 600 |
| 0.7808907 | uc009xmf.2 | LOC84856 | UCSC_knowngene | 2042 |
| 0.7811359 | ENST00000414562 | RP11-96M12.1 | Ensembl | 2036 |
| 0.7815792 | ENST00000468538 | RP1-149M18.4 | Ensembl | 743 |
| 0.7822266 | ENST00000455966 | RP1-224A6.3 | Ensembl | 615 |
| 0.7823391 | ENST00000512967 | AL031123.1 | Ensembl | 2641 |
| 0.7830794 | ENST00000428750 | RP1-60O19.1 | Ensembl | 805 |
| 0.7878549 | ENST00000439279 | RP11-436D23.1 | Ensembl | 226 |
| 0.7883644 | ENST00000417112 | RP11-554I8.2 | Ensembl | 917 |
| 0.7889931 | ENST00000431336 | AC097517.2 | Ensembl | 569 |
| 0.7897828 | ENST00000431650 | AC012457.2 | Ensembl | 466 |
| 0.7903704 | ENST00000413993 | RP11-482E14.1 | Ensembl | 832 |
| 0.7903903 | ENST00000441430 | RP11-571G1.2 | Ensembl | 435 |
| 0.7905329 | ENST00000448198 | XXbac-BPG254F23.7 | Ensembl | 381 |
| 0.790674 | BC043238 |  | misc_RNA | 2474 |
| 0.7907674 | HIT000395572 | | H-invDB | 354 |
| 0.7908195 | ENST00000300167 | RP13-49I15.3 | Ensembl | 258 |
| 0.7910956 | uc003pvl.1 | CR598940 | UCSC_knowngene | 1339 |
| 0.7919813 | NR_027124 | LOC339568 | RefSeq_NR | 1624 |
| 0.7924161 | NR_027355 | LOC645166 | RefSeq_NR | 670 |
| 0.7932262 | NR_024373 | LOC541471 | RefSeq_NR | 557 |
| 0.7939404 | ENST00000425900 | RP4-764O22.1 | Ensembl | 381 |
| 0.7940254 | ENST00000426922 | RP11-478H13.3 | Ensembl | 406 |
| 0.7942087 | ENST00000456403 | AC019349.2 | Ensembl | 2141 |
| 0.7945998 | AK096113 |  | misc_RNA | 2617 |
| 0.7948833 | ENST00000417843 | RP11-764K9.1 | Ensembl | 2818 |
| 0.7950056 | ENST00000438897 | AC068491.2 | Ensembl | 617 |
| 0.7957252 | NR_027085 | LOC284551 | RefSeq_NR | 2741 |
| 0.7963459 | ENST00000422253 | RP5-968D22.3 | Ensembl | 519 |
| 0.7965295 | uc001hmu.2 | BC045735 | UCSC_knowngene | 959 |
| 0.797582 | ENST00000446139 | AC011306.2 | Ensembl | 900 |
| 0.7975888 | ENST00000326734 | FAM87B | Ensembl | 1944 |
| 0.7980315 | ENST00000432701 | RP11-269C23.3 | Ensembl | 858 |
| 0.799038 | ENST00000424355 | AC023672.2 | Ensembl | 388 |
| 0.7999213 | ENST00000433761 | RP1-40E16.2 | Ensembl | 398 |
| 0.8010244 | ENST00000418666 | RP11-292F9.1 | Ensembl | 931 |
| 0.8010568 | ENST00000414054 | AL121656.4 | Ensembl | 1794 |
| 0.8017962 | G36891 |  | misc_RNA | 366 |
| 0.801956 | ENST00000413439 | AC006293.3 | Ensembl | 2127 |
| 0.8023868 | ENST00000445631 | RP11-91N2.3 | Ensembl | 206 |
| 0.8023919 | ENST00000451396 | RP11-478J18.2 | Ensembl | 476 |
| 0.8026652 | ENST00000432108 | RP11-219F10.1 | Ensembl | 309 |
| 0.8029049 | ENST00000453636 | AC092620.3 | Ensembl | 680 |
| 0.8046093 | ENST00000447571 | AC079807.4 | Ensembl | 1808 |
| 0.8051261 | ENST00000414598 | RP4-583K8.1 | Ensembl | 431 |
| 0.8060401 | ENST00000440388 | RP11-500G10.5 | Ensembl | 552 |
| 0.806098 | ENST00000412381 | AC142119.1 | Ensembl | 642 |
| 0.8073786 | BC042185 |  | RNAdb | 964 |
| 0.8073948 | uc002the.2 | LOC541471 | UCSC_knowngene | 1583 |
| 0.8076893 | ENST00000415702 | CTA-397C4.2 | Ensembl | 630 |
| 0.8084414 | AK092065 |  | RNAdb | 2496 |
| 0.8085732 | ENST00000444079 | AC093326.3 | Ensembl | 762 |
| 0.809547 | ENST00000445784 | AC002064.5 | Ensembl | 733 |
| 0.8098083 | ENST00000444800 | RP11-423C15.3 | Ensembl | 509 |
| 0.810371 | ENST00000457157 | AP000688.8 | Ensembl | 811 |
| 0.8105168 | ENST00000439014 | RP11-65J3.2 | Ensembl | 800 |
| 0.8108091 | ENST00000443688 | RP11-390F4.3 | Ensembl | 472 |
| 0.810901 | ENST00000417149 | RP11-298E9.6 | Ensembl | 390 |
| 0.8112393 | ENST00000419627 | RP3-323P13.2 | Ensembl | 244 |
| 0.8121204 | ENST00000454588 | RP1-45I4.3 | Ensembl | 552 |
| 0.8123973 | ENST00000412606 | AC096559.1 | Ensembl | 578 |
| 0.8129055 | X91348 |  | RNAdb | 1284 |
| 0.8139839 | ENST00000457044 | RP11-331F9.3 | Ensembl | 679 |
| 0.8151902 | ENST00000467677 | AC068610.3 | Ensembl | 685 |
| 0.8156417 | ENST00000417705 | RP11-54O7.3 | Ensembl | 1389 |
| 0.8167858 | ENST00000422157 | RP6-74O6.3 | Ensembl | 439 |
| 0.8176307 | uc002wsq.1 | BC045663 | UCSC_knowngene | 1827 |
| 0.8176973 | ENST00000446169 | RP11-410C4.4 | Ensembl | 390 |
| 0.8178791 | ENST00000415726 | RP11-336K24.5 | Ensembl | 401 |
| 0.8192011 | ENST00000438287 | RP4-738P15.1 | Ensembl | 468 |
| 0.8194147 | ENST00000444229 | RP11-527F13.1 | Ensembl | 583 |
| 0.8197401 | ENST00000423020 | RP5-963E22.4 | Ensembl | 542 |
| 0.8212197 | ENST00000437610 | AC011995.3 | Ensembl | 578 |
| 0.8216159 | ENST00000427049 | RP3-400B16.1 | Ensembl | 477 |
| 0.821817 | ENST00000434947 | RP3-448I9.1 | Ensembl | 405 |
| 0.8220101 | ENST00000429601 | RP11-368D24__A.1 | Ensembl | 487 |
| 0.8221913 | ENST00000417650 | RP11-439L18.1 | Ensembl | 554 |
| 0.8229241 | ENST00000412266 | AC006028.9 | Ensembl | 3288 |
| 0.8229326 | ENST00000427901 | RP11-63P12.7 | Ensembl | 361 |
| 0.8229769 | uc003ndh.2 | CR591483 | UCSC_knowngene | 2039 |
| 0.8245265 | ENST00000456477 | RP11-459F1.2 | Ensembl | 1035 |
| 0.8246835 | ENST00000432249 | AP000345.4 | Ensembl | 477 |
| 0.8247051 | ENST00000441001 | RP11-491H19.1 | Ensembl | 477 |
| 0.8248358 | ENST00000435156 | RP11-203P2.2 | Ensembl | 342 |
| 0.8250899 | NR_003674 | KGFLP1 | RefSeq_NR | 3612 |
| 0.8256299 | NR_024475 | LOC100216001 | RefSeq_NR | 1394 |
| 0.8262373 | ENST00000411595 | RP4-737E23.2 | Ensembl | 556 |
| 0.8263031 | ENST00000441665 | AP002856.4 | Ensembl | 635 |
| 0.8269045 | ENST00000442526 | RP11-517P14.2 | Ensembl | 303 |
| 0.8272189 | ENST00000457273 | RP11-475O6.1 | Ensembl | 1991 |
| 0.8274673 | ENST00000455039 | RP11-426A6.7 | Ensembl | 795 |
| 0.8277654 | ENST00000424837 | AP000282.3 | Ensembl | 576 |
| 0.8278497 | ENST00000420477 | RP1-66N13.3 | Ensembl | 508 |
| 0.8284266 | uc003zpr.2 | CR627240 | UCSC_knowngene | 4804 |
| 0.8285676 | ENST00000455028 | AF015262.2 | Ensembl | 698 |
| 0.8287595 | ENST00000427208 | AC019100.5 | Ensembl | 1875 |
| 0.829032 | uc002rzk.2 | BC043355 | UCSC_knowngene | 1275 |
| 0.8293962 | uc010ype.1 | DM004471 | UCSC_knowngene | 110 |
| 0.8295469 | uc002zll.1 | CR605662 | UCSC_knowngene | 1732 |
| 0.8296779 | ENST00000426260 | AC007403.2 | Ensembl | 625 |
| 0.8299523 | NR_023918 | HSPC157 | RefSeq_NR | 1103 |
| 0.8303703 | NR_026775 | C6orf41 | RefSeq_NR | 983 |
| 0.830759 | ENST00000439156 | RP11-776H12.1 | Ensembl | 1796 |
| 0.8307813 | ENST00000452637 | RP11-403I13.7 | Ensembl | 695 |
| 0.8314462 | ENST00000424696 | RP1-272L16.1 | Ensembl | 865 |
| 0.831559 | ENST00000441638 | AP002856.7 | Ensembl | 569 |
| 0.8321809 | ENST00000432822 | AC019055.1 | Ensembl | 443 |
| 0.8325505 | ENST00000412132 | RP3-413H6.2 | Ensembl | 554 |
| 0.8326014 | ENST00000450408 | RP3-355C18.7 | Ensembl | 1016 |
| 0.8332526 | ENST00000427290 | RP5-965F6.2 | Ensembl | 547 |
| 0.8348181 | ENST00000457720 | RP11-217B7.3 | Ensembl | 606 |
| 0.8349254 | ENST00000422117 | AC124997.1 | Ensembl | 2491 |
| 0.8350989 | ENST00000416657 | RP11-36D19.8 | Ensembl | 339 |
| 0.8352091 | ENST00000430284 | RP11-508M1.7 | Ensembl | 709 |
| 0.8352748 | ENST00000443926 | AC139712.4 | Ensembl | 639 |
| 0.8355624 | ENST00000446520 | AC069394.1 | Ensembl | 553 |
| 0.8363899 | ENST00000272546 | RP11-143M1.7 | Ensembl | 369 |
| 0.8371626 | ENST00000436113 | RP3-449H6.1 | Ensembl | 473 |
| 0.8377564 | ENST00000418607 | BX004987.7 | Ensembl | 964 |
| 0.8385756 | ENST00000421937 | RP11-458D21.2 | Ensembl | 666 |
| 0.8392362 | ENST00000430391 | AF127577.8 | Ensembl | 781 |
| 0.839677 | ENST00000443504 | RP11-686D16.1 | Ensembl | 381 |
| 0.8398195 | ENST00000423380 | RP11-305L7.1 | Ensembl | 815 |
| 0.8407482 | ENST00000414126 | AC073316.2 | Ensembl | 418 |
| 0.8411421 | ENST00000452494 | RP11-129J12.1 | Ensembl | 392 |
| 0.8423292 | NR_023919 | HSPC157 | RefSeq_NR | 1039 |
| 0.842369 | ENST00000412357 | RP5-1114G22.2 | Ensembl | 775 |
| 0.8431194 | ENST00000418764 | AC004543.2 | Ensembl | 655 |
| 0.843266 | NR_024159 | DGCR9 | RefSeq_NR | 2434 |
| 0.8434714 | ENST00000448840 | AL132709.1 | Ensembl | 520 |
| 0.8442756 | ENST00000457536 | RP5-991B18.1 | Ensembl | 277 |
| 0.8444257 | uc002qgt.1 | BC131616 | UCSC_knowngene | 2014 |
| 0.8445047 | ENST00000413151 | AC009505.4 | Ensembl | 424 |
| 0.8445626 | ENST00000430134 | AC007386.4 | Ensembl | 1018 |
| 0.8453623 | ENST00000450310 | RP3-471C18.2 | Ensembl | 191 |
| 0.8453795 | ENST00000458084 | NCRNA00202 | Ensembl | 1787 |
| 0.845549 | ENST00000423706 | AC012506.1 | Ensembl | 525 |
| 0.8459779 | ENST00000457976 | RP11-305L7.3 | Ensembl | 506 |
| 0.8460293 | uc004dvh.2 | LOC92249 | UCSC_knowngene | 2713 |
| 0.8464378 | ENST00000413994 | RP11-527D7.1 | Ensembl | 473 |
| 0.8465377 | ENST00000457725 | AP003774.6 | Ensembl | 985 |
| 0.8469908 | ENST00000420549 | RP11-147G16.1 | Ensembl | 671 |
| 0.8471249 | ENST00000447908 | RP4-704D23.1 | Ensembl | 579 |
| 0.8472475 | ENST00000458293 | RP5-897D18.1 | Ensembl | 483 |
| 0.8479503 | AK124090 |  | misc_RNA | 1801 |
| 0.8486759 | ENST00000415471 | RP11-100G15.10 | Ensembl | 211 |
| 0.8498248 | ENST00000437080 | RP11-115A15.1 | Ensembl | 349 |
| 0.85032 | ENST00000450820 | RP11-335G20.5 | Ensembl | 777 |
| 0.8509535 | ENST00000439041 | AC061992.2 | Ensembl | 799 |
| 0.8512016 | ENST00000455327 | RP1-40E16.3 | Ensembl | 335 |
| 0.8513186 | uc003vql.1 | CR618431 | UCSC_knowngene | 1618 |
| 0.8517426 | ENST00000425125 | AC017053.1 | Ensembl | 579 |
| 0.8518842 | ENST00000414199 | RP5-1066H13.4 | Ensembl | 471 |
| 0.8529348 | ENST00000450206 | RP11-432J24.3 | Ensembl | 1013 |
| 0.8533591 | ENST00000431997 | RP1-288M22.2 | Ensembl | 454 |
| 0.8554895 | ENST00000438692 | AC013400.2 | Ensembl | 2239 |
| 0.8557601 | ENST00000422082 | RP11-442J17.3 | Ensembl | 746 |
| 0.8560163 | ENST00000426539 | AC130710.1 | Ensembl | 1096 |
| 0.8561829 | ENST00000435044 | RP11-490N5.1 | Ensembl | 374 |
| 0.8562195 | ENST00000434832 | RP11-159J2.2 | Ensembl | 387 |
| 0.8566203 | ENST00000431897 | AC108051.1 | Ensembl | 437 |
| 0.8569668 | HIT000221891 | | H-invDB | 245 |
| 0.8576861 | ENST00000435738 | AP001476.3 | Ensembl | 355 |
| 0.8584175 | ENST00000440163 | RP4-669L17.4 | Ensembl | 462 |
| 0.8591381 | NR_026821 | FAM138B | RefSeq_NR | 1130 |
| 0.8592805 | ENST00000448347 | RP11-282I1.1 | Ensembl | 831 |
| 0.8592969 | ENST00000450928 | AP000302.58 | Ensembl | 666 |
| 0.8608525 | ENST00000456681 | AC007464.1 | Ensembl | 746 |
| 0.8608882 | uc010glg.2 | NCRNA00158 | UCSC_knowngene | 1379 |
| 0.8617796 | uc003prn.1 | AK124400 | UCSC_knowngene | 2232 |
| 0.8623035 | ENST00000434901 | RP1-153P14.4 | Ensembl | 514 |
| 0.8626774 | ENST00000427157 | RP11-367G18.1 | Ensembl | 327 |
| 0.8631198 | ENST00000445585 | RP5-899B16.2 | Ensembl | 786 |
| 0.86396 | ENST00000438770 | AC012309.5 | Ensembl | 462 |
| 0.8641551 | uc002wsk.1 | CR627206 | UCSC_knowngene | 4392 |
| 0.8644101 | ENST00000438185 | LL22NC03-102D1.18 | Ensembl | 451 |
| 0.8647245 | ENST00000497017 | AC093627.11 | Ensembl | 568 |
| 0.8652438 | ENST00000456795 | RP3-331H24.4 | Ensembl | 352 |
| 0.8653095 | ENST00000441617 | RP11-342C20.3 | Ensembl | 840 |
| 0.8657919 | ENST00000438640 | RP11-76K19.5 | Ensembl | 422 |
| 0.86587 | ENST00000445785 | NCRNA00102 | Ensembl | 1340 |
| 0.8663682 | ENST00000458693 | RP1-249I4.2 | Ensembl | 465 |
| 0.8663934 | ENST00000366195 | RP11-101E5.1 | Ensembl | 419 |
| 0.8665241 | ENST00000448282 | RP1-102H19.6 | Ensembl | 542 |
| 0.8673264 | ENST00000416908 | RP11-449J1.1 | Ensembl | 423 |
| 0.8680343 | ENST00000419796 | RP11-716O23.1 | Ensembl | 699 |
| 0.8681042 | ENST00000421153 | RP4-811H24.8 | Ensembl | 267 |
| 0.8681924 | ENST00000419514 | RP11-366O17.2 | Ensembl | 192 |
| 0.868205 | ENST00000436492 | RP1-125N5.2 | Ensembl | 557 |
| 0.8682161 | ENST00000418076 | RP11-37E23.5 | Ensembl | 437 |
| 0.8689536 | ENST00000422074 | RP11-471M2.2 | Ensembl | 344 |
| 0.8691046 | ENST00000429060 | RP11-314C16.1 | Ensembl | 489 |
| 0.8692921 | ENST00000442712 | RP11-439E19.7 | Ensembl | 522 |
| 0.8694248 | NR_028379 | NCRNA00182 | RefSeq_NR | 2356 |
| 0.8696548 | ENST00000452212 | AC097724.3 | Ensembl | 726 |
| 0.8698204 | ENST00000456514 | RP11-422P15.3 | Ensembl | 1168 |
| 0.8702048 | ENST00000450325 | AC010982.2 | Ensembl | 465 |
| 0.8705369 | ENST00000418612 | AC051649.6 | Ensembl | 722 |
| 0.8706135 | uc010gdi.2 | LOC284798 | UCSC_knowngene | 770 |
| 0.8707092 | ENST00000413328 | RP11-298A8.2 | Ensembl | 217 |
| 0.8719403 | EF212288 |  | misc_RNA | 396 |
| 0.8722072 | ENST00000430672 | RP11-138M12.1 | Ensembl | 281 |
| 0.8722143 | uc001dva.2 | BC043293 | UCSC_knowngene | 1824 |
| 0.8725454 | ENST00000438499 | RP11-1M18.1 | Ensembl | 517 |
| 0.8730642 | ENST00000455981 | RP11-344B5.2 | Ensembl | 583 |
| 0.8731192 | ENST00000439450 | RP5-1097P24.1 | Ensembl | 439 |
| 0.8733251 | NR_027341 | C9orf44 | RefSeq_NR | 2000 |
| 0.8733295 | ENST00000456424 | RP1-249H1.2 | Ensembl | 375 |
| 0.8735527 | ENST00000456933 | RP11-286B14.1 | Ensembl | 714 |
| 0.8737441 | ENST00000431646 | RP11-157D23.1 | Ensembl | 880 |
| 0.8741038 | ENST00000443579 | RP1-159A19.4 | Ensembl | 283 |
| 0.8751772 | ENST00000440735 | RP11-365P13.3 | Ensembl | 684 |
| 0.8763259 | uc002rpc.1 | CR617033 | UCSC_knowngene | 1343 |
| 0.8764266 | uc010awi.1 | BC148240 | UCSC_knowngene | 557 |
| 0.8769991 | uc002tpv.1 | LOC151121 | UCSC_knowngene | 2213 |
| 0.8770469 | ENST00000447297 | RP11-428L9.1 | Ensembl | 366 |
| 0.8771504 | ENST00000424592 | RP11-63G10.2 | Ensembl | 395 |
| 0.8775275 | ENST00000434938 | NCRNA00106 | Ensembl | 357 |
| 0.8778267 | uc001czf.2 | AK055150 | UCSC_knowngene | 1123 |
| 0.8780403 | ENST00000417696 | RP11-23O13.1 | Ensembl | 390 |
| 0.8792146 | ENST00000422574 | RP5-1195D24.1 | Ensembl | 833 |
| 0.8803141 | ENST00000431300 | RP11-182L21.2 | Ensembl | 886 |
| 0.8810354 | ENST00000437233 | AC084149.2 | Ensembl | 284 |
| 0.8811555 | ENST00000412427 | RP11-380J14.1 | Ensembl | 4676 |
| 0.8812665 | ENST00000427447 | AP000569.8 | Ensembl | 9124 |
| 0.8814128 | ENST00000420901 | RP11-251P6.1 | Ensembl | 410 |
| 0.8817348 | ENST00000422047 | RP11-245M24.1 | Ensembl | 639 |
| 0.8821147 | ENST00000438113 | RP1-90L6.3 | Ensembl | 517 |
| 0.8830833 | ENST00000458006 | RP3-416J7.3 | Ensembl | 751 |
| 0.8852537 | ENST00000443419 | AC073628.1 | Ensembl | 635 |
| 0.8860433 | ENST00000446201 | RP11-406A20.1 | Ensembl | 457 |
| 0.8862436 | ENST00000411451 | RP11-308N19.4 | Ensembl | 690 |
| 0.8862469 | ENST00000423797 | AC006038.4 | Ensembl | 603 |
| 0.8867245 | ENST00000425901 | AC091178.1 | Ensembl | 3239 |
| 0.8874923 | NR_026951 | C17orf44 | RefSeq_NR | 2115 |
| 0.8882629 | ENST00000442478 | RP11-51B13.1 | Ensembl | 353 |
| 0.888602 | ENST00000440802 | AC097532.2 | Ensembl | 322 |
| 0.8887844 | NR_024351 | NCRNA00160 | RefSeq_NR | 694 |
| 0.8893064 | uc004bbt.2 | BC035187 | UCSC_knowngene | 3729 |
| 0.8893099 | ENST00000453810 | RP11-1148L6.5 | Ensembl | 450 |
| 0.8893731 | ENST00000423323 | AC015842.1 | Ensembl | 2998 |
| 0.8897779 | ENST00000454647 | C18orf20 | Ensembl | 339 |
| 0.8903556 | ENST00000414488 | AL132709.5 | Ensembl | 800 |
| 0.8905811 | ENST00000422473 | AP000688.29 | Ensembl | 2336 |
| 0.8919501 | NR_026965 | LOC121952 | RefSeq_NR | 2312 |
| 0.8924866 | ENST00000424968 | RP11-373D17.1 | Ensembl | 507 |
| 0.8926125 | ENST00000437892 | RP11-124L5.7 | Ensembl | 674 |
| 0.8927286 | NR_026927 | LOC115110 | RefSeq_NR | 2216 |
| 0.8928211 | ENST00000432331 | RP11-478K15.1 | Ensembl | 340 |
| 0.8934367 | ENST00000429444 | RP1-232L24.3 | Ensembl | 474 |
| 0.8934847 | ENST00000448901 | AC105253.1 | Ensembl | 4209 |
| 0.8935272 | ENST00000439157 | RP11-94M14.2 | Ensembl | 784 |
| 0.8939131 | ENST00000439438 | RP11-305L7.6 | Ensembl | 454 |
| 0.8946483 | ENST00000423739 | RP11-359P14.1 | Ensembl | 419 |
| 0.8946649 | ENST00000417801 | RP11-394O9.1 | Ensembl | 446 |
| 0.8948812 | NR_003948 | HCG22 | RefSeq_NR | 5670 |
| 0.8953681 | ENST00000455554 | RP11-35J1.1 | Ensembl | 569 |
| 0.8955316 | NR_029401 | LOC731275 | RefSeq_NR | 3834 |
| 0.8964213 | ENST00000453798 | AC073094.4 | Ensembl | 433 |
| 0.896892 | ENST00000429305 | RP11-302L19.1 | Ensembl | 429 |
| 0.8972024 | ENST00000449585 | RP11-631M21.1 | Ensembl | 347 |
| 0.8972586 | ENST00000428643 | RP11-216B9.7 | Ensembl | 334 |
| 0.8975775 | ENST00000436706 | RP11-365O16.3 | Ensembl | 790 |
| 0.8978465 | uc004dgk.1 | AK098783 | UCSC_knowngene | 1961 |
| 0.8993272 | ENST00000423023 | RP11-523H24.3 | Ensembl | 376 |
| 0.8995672 | ENST00000435612 | XXyac-YX65C7_A.3 | Ensembl | 479 |
| 0.8996197 | ENST00000425322 | AC006041.1 | Ensembl | 328 |
| 0.8998907 | ENST00000452716 | AC023115.3 | Ensembl | 1074 |
| 0.9005215 | ENST00000437330 | AC103564.7 | Ensembl | 562 |
| 0.9007243 | uc001ali.1 | AK124708 | UCSC_knowngene | 3242 |
| 0.900965 | ENST00000435367 | RP11-475D12.2 | Ensembl | 500 |
| 0.9010941 | ENST00000437014 | RP11-20J15.2 | Ensembl | 841 |
| 0.9024812 | ENST00000412360 | AC102948.2 | Ensembl | 682 |
| 0.9026117 | ENST00000430772 | NCRNA00182 | Ensembl | 708 |
| 0.90317 | ENST00000411453 | RP11-112L6.4 | Ensembl | 573 |
| 0.9031967 | NR_002171 | OR7E156P | RefSeq_NR | 2647 |
| 0.9039292 | uc001czt.1 | AK097193 | UCSC_knowngene | 2344 |
| 0.9039849 | ENST00000369027 | RP11-10J18.3 | Ensembl | 266 |
| 0.9040611 | ENST00000455068 | AC011286.1 | Ensembl | 561 |
| 0.9041874 | ENST00000455568 | NCRNA00087 | Ensembl | 471 |
| 0.9042743 | ENST00000436140 | RP11-80K21.3 | Ensembl | 789 |
| 0.9043818 | ENST00000441473 | RP11-343J18.2 | Ensembl | 316 |
| 0.9046878 | ENST00000411791 | FAM25E | Ensembl | 322 |
| 0.9047565 | NR_024100 | C21orf130 | RefSeq_NR | 2019 |
| 0.9048012 | uc002xid.1 | AX746690 | UCSC_knowngene | 2002 |
| 0.9061803 | ENST00000457321 | RP5-1011O1.3 | Ensembl | 684 |
| 0.9064348 | ENST00000444265 | RP1-67M12.1 | Ensembl | 327 |
| 0.9066498 | ENST00000414654 | AC092580.3 | Ensembl | 469 |
| 0.907209 | ENST00000366181 | RP11-296A18.3 | Ensembl | 2366 |
| 0.9076518 | ENST00000417409 | RP11-161I10.1 | Ensembl | 506 |
| 0.9076606 | ENST00000444042 | RP1-18D14.3 | Ensembl | 363 |
| 0.9079269 | ENST00000412149 | LL22NC03-2H8.4 | Ensembl | 524 |
| 0.908243 | NR_024444 | LOC100133985 | RefSeq_NR | 661 |
| 0.9088297 | ENST00000433460 | RP11-275N1.1 | Ensembl | 439 |
| 0.9088336 | NR_027248 | LOC100101938 | RefSeq_NR | 3794 |
| 0.9090796 | ENST00000451596 | RP11-63P12.6 | Ensembl | 672 |
| 0.9090995 | ENST00000412339 | RP11-390F4.10 | Ensembl | 422 |
| 0.9094605 | AK096133 |  | misc_RNA | 2007 |
| 0.9098773 | ENST00000434902 | RP11-34C15.1 | Ensembl | 869 |
| 0.9103055 | ENST00000414967 | AC062031.1 | Ensembl | 747 |
| 0.9107262 | ENST00000424605 | RP11-125B21.2 | Ensembl | 746 |
| 0.9107524 | uc004elm.1 | AK026512 | UCSC_knowngene | 994 |
| 0.9119778 | ENST00000451108 | RP11-410K21.2 | Ensembl | 635 |
| 0.9128722 | ENST00000436162 | XXbac-B33L19.3 | Ensembl | 1069 |
| 0.9131496 | ENST00000421174 | RP11-403I13.2 | Ensembl | 905 |
| 0.91325 | ENST00000449126 | CTA-929C8.5 | Ensembl | 274 |
| 0.9132756 | ENST00000449941 | RP13-455A7.1 | Ensembl | 219 |
| 0.913282 | uc010nxz.1 | FLJ39609 | UCSC_knowngene | 436 |
| 0.913408 | uc010gei.1 | BASE | UCSC_knowngene | 558 |
| 0.9135228 | ENST00000411599 | RP11-254K3.1 | Ensembl | 479 |
| 0.9135671 | ENST00000367716 | RP11-296O14.3 | Ensembl | 1815 |
| 0.913732 | ENST00000455390 | RP11-514O12.2 | Ensembl | 627 |
| 0.9153201 | NR_027312 | LOC153910 | RefSeq_NR | 2293 |
| 0.9155279 | ENST00000414795 | AC017076.5 | Ensembl | 506 |
| 0.9159517 | ENST00000439760 | RP11-374M1.2 | Ensembl | 747 |
| 0.916208 | ENST00000417506 | AC004941.3 | Ensembl | 3120 |
| 0.9170948 | ENST00000456240 | RP11-323I1.1 | Ensembl | 347 |
| 0.9173989 | ENST00000411511 | RP3-462D8.2 | Ensembl | 428 |
| 0.9192989 | ENST00000418451 | AC010967.3 | Ensembl | 333 |
| 0.9196559 | ENST00000432272 | RP13-297E16.4 | Ensembl | 382 |
| 0.9196946 | ENST00000445662 | AL583842.1 | Ensembl | 965 |
| 0.920212 | ENST00000436739 | RP5-858B6.1 | Ensembl | 311 |
| 0.9207082 | ENST00000418836 | AC009413.3 | Ensembl | 534 |
| 0.9226415 | ENST00000454439 | RP1-102D24.5 | Ensembl | 545 |
| 0.9227209 | ENST00000420391 | CTA-796E4.3 | Ensembl | 188 |
| 0.9230641 | ENST00000448650 | AC008069.1 | Ensembl | 596 |
| 0.9230689 | ENST00000416190 | RP5-1010E17.1 | Ensembl | 1340 |
| 0.9232098 | ENST00000448835 | RP11-554I8.1 | Ensembl | 499 |
| 0.9232868 | ENST00000440848 | RP11-145H9.3 | Ensembl | 834 |
| 0.9236191 | ENST00000444956 | RP11-384P7.6 | Ensembl | 486 |
| 0.9239152 | ENST00000453451 | AC012593.1 | Ensembl | 580 |
| 0.9239454 | ENST00000445346 | RP11-406O16.1 | Ensembl | 674 |
| 0.9239848 | uc004eqv.2 | AK057519 | UCSC_knowngene | 2146 |
| 0.9240246 | ENST00000435429 | RP3-470L22.1 | Ensembl | 388 |
| 0.9241426 | ENST00000421423 | RP11-450H6.1 | Ensembl | 545 |
| 0.9244433 | ENST00000456771 | RP11-540K16.2 | Ensembl | 532 |
| 0.924623 | ENST00000432481 | AC009410.1 | Ensembl | 678 |
| 0.9247256 | ENST00000443747 | RP11-164D18.2 | Ensembl | 566 |
| 0.9247991 | ENST00000442318 | RP11-576D8.4 | Ensembl | 2885 |
| 0.9249076 | ENST00000424052 | XXbac-B444P24.8 | Ensembl | 2183 |
| 0.9250778 | ENST00000432697 | RP11-71L7.1 | Ensembl | 708 |
| 0.9256053 | ENST00000413926 | RP11-547C18.2 | Ensembl | 606 |
| 0.925709 | ENST00000437598 | RP5-1027O11.1 | Ensembl | 787 |
| 0.9258295 | ENST00000419347 | AC019118.3 | Ensembl | 416 |
| 0.9258456 | NR_015353 | LOC92249 | RefSeq_NR | 2886 |
| 0.926068 | uc003sme.2 | BC034268 | UCSC_knowngene | 2066 |
| 0.9264669 | ENST00000416845 | AC093159.1 | Ensembl | 424 |
| 0.9271197 | NR_027120 | LOC729467 | RefSeq_NR | 1043 |
| 0.9273982 | ENST00000433121 | RP5-908M14.5 | Ensembl | 477 |
| 0.9276173 | ENST00000433081 | RP11-458D21.1 | Ensembl | 814 |
| 0.9278447 | ENST00000437718 | RP1-90J20.2 | Ensembl | 484 |
| 0.9287469 | uc001vlo.2 | BC038529 | UCSC_knowngene | 1000 |
| 0.9291077 | ENST00000461248 | RP1-300I2.2 | Ensembl | 556 |
| 0.9293063 | ENST00000435973 | RP11-286M16.1 | Ensembl | 385 |
| 0.929488 | uc010jzo.2 | DM004406 | UCSC_knowngene | 118 |
| 0.929584 | ENST00000473370 | RP1-149M18.3 | Ensembl | 441 |
| 0.9301609 | ENST00000468838 | RP11-490N5.3 | Ensembl | 439 |
| 0.9306572 | NR_026878 | MGC12982 | RefSeq_NR | 2527 |
| 0.9311012 | ENST00000423422 | RP11-336K24.4 | Ensembl | 420 |
| 0.9311273 | ENST00000457746 | AP006216.5 | Ensembl | 333 |
| 0.931931 | uc010gjw.1 | AK309218 | UCSC_knowngene | 3684 |
| 0.9319813 | ENST00000441326 | RP11-38P6.1 | Ensembl | 792 |
| 0.9325101 | ENST00000429512 | AP001476.4 | Ensembl | 415 |
| 0.9325574 | ENST00000446392 | RP1-12G14.5 | Ensembl | 378 |
| 0.9329561 | ENST00000412439 | RP11-810B23.1 | Ensembl | 187 |
| 0.9330762 | ENST00000448672 | AC068492.1 | Ensembl | 469 |
| 0.9333227 | ENST00000453470 | RP11-90M5.5 | Ensembl | 913 |
| 0.933985 | ENST00000446372 | SFTA1P | Ensembl | 873 |
| 0.9342379 | ENST00000447511 | ANTXRL | Ensembl | 506 |
| 0.9342843 | uc003opf.1 | BC132805 | UCSC_knowngene | 3122 |
| 0.9343747 | ENST00000427579 | AC007403.3 | Ensembl | 567 |
| 0.9345808 | ENST00000452933 | RP11-318G21.2 | Ensembl | 546 |
| 0.9349451 | ENST00000419576 | RP11-561O23.7 | Ensembl | 416 |
| 0.9356253 | ENST00000446115 | RP11-439L18.3 | Ensembl | 783 |
| 0.9362243 | ENST00000435291 | AC108051.2 | Ensembl | 549 |
| 0.9363741 | ENST00000447500 | RP11-206L10.8 | Ensembl | 523 |
| 0.9367926 | ENST00000453579 | RP1-257I9.2 | Ensembl | 554 |
| 0.9368865 | ENST00000428426 | AC002511.3 | Ensembl | 376 |
| 0.9369131 | ENST00000425194 | RP13-614K11.2 | Ensembl | 937 |
| 0.9370296 | ENST00000444978 | RP3-380B8.3 | Ensembl | 361 |
| 0.9373992 | uc003pji.2 | BC038576 | UCSC_knowngene | 553 |
| 0.9377258 | uc009xhl.2 | BC127786 | UCSC_knowngene | 269 |
| 0.9378832 | ENST00000420330 | AC009404.2 | Ensembl | 3090 |
| 0.9379014 | ENST00000434384 | AC004383.5 | Ensembl | 724 |
| 0.9379093 | ENST00000450187 | RP11-25J23.3 | Ensembl | 348 |
| 0.9380174 | ENST00000448791 | RP1-50O24.6 | Ensembl | 306 |
| 0.9381194 | uc002ytn.2 | CR626360 | UCSC_knowngene | 2657 |
| 0.9381328 | ENST00000438736 | AC016735.3 | Ensembl | 569 |
| 0.9382084 | ENST00000421828 | RP11-195F19.5 | Ensembl | 835 |
| 0.9387693 | uc001kpt.2 | chromosome 10 open reading frame 139 | UCSC_knowngene | 730 |
| 0.938835 | ENST00000450546 | AL592494.1 | Ensembl | 426 |
| 0.9388716 | ENST00000417514 | RP11-439L8.4 | Ensembl | 1122 |
| 0.9391169 | ENST00000450983 | RP4-669L17.2 | Ensembl | 607 |
| 0.9391462 | ENST00000442865 | AC007255.7 | Ensembl | 590 |
| 0.9393148 | ENST00000454149 | AC099552.3 | Ensembl | 367 |
| 0.9393292 | ENST00000439703 | RP11-417E7.1 | Ensembl | 647 |
| 0.9396485 | ENST00000438797 | RP1-159G19.1 | Ensembl | 481 |
| 0.9397231 | ENST00000443207 | RP4-580N22.2 | Ensembl | 190 |
| 0.939971 | NR_027311 | LOC153910 | RefSeq_NR | 2290 |
| 0.9400771 | BC024156 |  | misc_RNA | 2195 |
| 0.9407854 | ENST00000448204 | AC114752.1 | Ensembl | 289 |
| 0.9408118 | uc001eng.1 | AX747132 | UCSC_knowngene | 1711 |
| 0.9416121 | ENST00000440189 | RP11-179B15.5 | Ensembl | 328 |
| 0.9420349 | ENST00000454331 | RP4-610C12.3 | Ensembl | 553 |
| 0.9422089 | ENST00000443373 | AC002331.1 | Ensembl | 294 |
| 0.9425618 | ENST00000449602 | AJ006998.2 | Ensembl | 1000 |
| 0.9428345 | ENST00000451792 | AC017060.1 | Ensembl | 442 |
| 0.9434575 | ENST00000445072 | RP11-373J16.1 | Ensembl | 784 |
| 0.9440599 | uc001uzl.2 | BC025370 | UCSC_knowngene | 976 |
| 0.9443871 | ENST00000447999 | AC060834.3 | Ensembl | 618 |
| 0.9455978 | ENST00000367276 | C9orf44 | Ensembl | 738 |
| 0.9456542 | ENST00000439455 | RP11-78O9.1 | Ensembl | 352 |
| 0.9457598 | ENST00000441444 | AC144525.1 | Ensembl | 592 |
| 0.9463388 | ENST00000377547 | RP11-374M1.5 | Ensembl | 439 |
| 0.9463818 | ENST00000411542 | AC011288.2 | Ensembl | 526 |
| 0.9471027 | ENST00000421071 | AC068057.2 | Ensembl | 482 |
| 0.9472068 | ENST00000431171 | RP11-125I23.3 | Ensembl | 580 |
| 0.9474447 | uc002zsa.1 | AK094309 | UCSC_knowngene | 2167 |
| 0.9477133 | ENST00000414480 | RP11-14O22.1 | Ensembl | 511 |
| 0.9477897 | uc002jiu.1 | AK094963 | UCSC_knowngene | 3032 |
| 0.9480666 | uc001jef.2 | LOC643650 | UCSC_knowngene | 2876 |
| 0.9480676 | AK026168 |  | NRED | 2638 |
| 0.948612 | ENST00000418416 | AC079163.1 | Ensembl | 407 |
| 0.9487789 | uc002jgo.1 | CR595591 | UCSC_knowngene | 607 |
| 0.9500467 | uc001hwj.1 | AK125357 | UCSC_knowngene | 3574 |
| 0.9503946 | ENST00000426575 | RP11-318C24.2 | Ensembl | 657 |
| 0.9518993 | ENST00000445296 | DAQB-12N14.5 | Ensembl | 399 |
| 0.9519451 | ENST00000420956 | RP11-408O13.2 | Ensembl | 518 |
| 0.9523602 | ENST00000443771 | RP11-53B5.1 | Ensembl | 391 |
| 0.9524199 | ENST00000445932 | RP11-298E9.5 | Ensembl | 597 |
| 0.9528467 | ENST00000437899 | RP11-38L15.2 | Ensembl | 369 |
| 0.9528791 | uc001dmk.2 | BC043534 | UCSC_knowngene | 884 |
| 0.9530433 | ENST00000420981 | RP11-420G6.4 | Ensembl | 1588 |
| 0.9530522 | ENST00000434510 | CTA-796E4.4 | Ensembl | 357 |
| 0.9531486 | ENST00000438372 | RP11-69C17.1 | Ensembl | 777 |
| 0.9538931 | ENST00000381105 | RP11-706O15.7 | Ensembl | 2118 |
| 0.9545872 | ENST00000422609 | RP11-484H12.5 | Ensembl | 458 |
| 0.9545966 | ENST00000425150 | RP11-348F1.3 | Ensembl | 451 |
| 0.95494 | ENST00000419463 | AC019117.1 | Ensembl | 837 |
| 0.9553064 | NR_024411 | LOC254312 | RefSeq_NR | 946 |
| 0.9555723 | ENST00000416100 | AC102953.6 | Ensembl | 242 |
| 0.9558655 | ENST00000413005 | RP1-79C4.1 | Ensembl | 1923 |
| 0.9566584 | uc011mgz.1 | NCRNA00107 | UCSC_knowngene | 668 |
| 0.9566793 | ENST00000439454 | RP11-527N12.1 | Ensembl | 348 |
| 0.9568016 | NR_027354 | LOC645166 | RefSeq_NR | 1104 |
| 0.9568481 | ENST00000417651 | RP3-437I16.1 | Ensembl | 2148 |
| 0.9570543 | ENST00000424640 | RP11-423O2.7 | Ensembl | 967 |
| 0.9573144 | ENST00000427132 | RP13-30A9.1 | Ensembl | 499 |
| 0.9575327 | uc010aeh.1 | AK127969 | UCSC_knowngene | 3223 |
| 0.9575378 | ENST00000440860 | RP11-75F3.1 | Ensembl | 376 |
| 0.9578319 | NR_002323 | TUG1 | RefSeq_NR | 7115 |
| 0.9584757 | uc010ypd.1 | DL231926 | UCSC_knowngene | 110 |
| 0.9590761 | ENST00000430537 | AC007036.6 | Ensembl | 674 |
| 0.959124 | ENST00000413762 | RP11-261C10.3 | Ensembl | 124 |
| 0.9591325 | ENST00000414398 | HCG24 | Ensembl | 717 |
| 0.9600459 | ENST00000423408 | RP4-794H19.1 | Ensembl | 1211 |
| 0.9608563 | ENST00000431117 | AC008278.3 | Ensembl | 568 |
| 0.961113 | ENST00000447883 | RP11-277L2.3 | Ensembl | 561 |
| 0.9615421 | ENST00000433310 | AF131217.1 | Ensembl | 900 |
| 0.9616152 | ENST00000417298 | AC100848.1 | Ensembl | 637 |
| 0.9618796 | ENST00000415883 | RP11-346D19.1 | Ensembl | 597 |
| 0.9619716 | uc003qdd.2 | AF086187 | UCSC_knowngene | 990 |
| 0.9621756 | ENST00000449345 | RP11-85G21.1 | Ensembl | 399 |
| 0.9622778 | ENST00000427337 | RP11-445P19.1 | Ensembl | 424 |
| 0.9623692 | NR_033380 | NCRNA00103 | RefSeq_NR | 2915 |
| 0.9624901 | NR_026807 | C6orf155 | RefSeq_NR | 2933 |
| 0.9625776 | ENST00000450520 | RP11-216M21.7 | Ensembl | 3200 |
| 0.9636476 | uc002goc.1 | AK123263 | UCSC_knowngene | 2800 |
| 0.9639 | ENST00000424705 | RP3-461P17.10 | Ensembl | 514 |
| 0.9644858 | ENST00000425517 | RP11-424D14.1 | Ensembl | 627 |
| 0.9652731 | ENST00000415530 | XX-C2158C6.3 | Ensembl | 439 |
| 0.965336 | ENST00000433825 | XX-FW83128A1.2 | Ensembl | 341 |
| 0.9655836 | ENST00000443030 | AC092168.4 | Ensembl | 236 |
| 0.9659924 | ENST00000422833 | RP1-213J1P__B.2 | Ensembl | 115 |
| 0.9662512 | uc002zgn.1 | BC046564 | UCSC_knowngene | 744 |
| 0.9664887 | uc001dnu.1 | AK022898 | UCSC_knowngene | 2786 |
| 0.9678489 | NR_024464 | LOC100188949 | RefSeq_NR | 1589 |
| 0.9696338 | ENST00000442821 | AC009236.2 | Ensembl | 553 |
| 0.9699128 | ENST00000438217 | XX-C2158C12.2 | Ensembl | 758 |
| 0.9702653 | ENST00000453787 | RP11-151D14.1 | Ensembl | 583 |
| 0.9705012 | ENST00000431369 | RP11-354I10.1 | Ensembl | 661 |
| 0.9724833 | uc001lkg.1 | AK124226 | UCSC_knowngene | 3086 |
| 0.9746002 | ENST00000426112 | RP3-439F8.1 | Ensembl | 2949 |
| 0.9746071 | ENST00000444436 | RP5-1031J8.1 | Ensembl | 480 |
| 0.9748933 | uc004abv.1 | BC121813 | UCSC_knowngene | 1106 |
| 0.9759622 | ENST00000416646 | RP11-347D21.2 | Ensembl | 456 |
| 0.9759779 | ENST00000431697 | AC106053.1 | Ensembl | 368 |
| 0.9765755 | ENST00000457945 | RP11-125M16.1 | Ensembl | 894 |
| 0.9769802 | uc004bxy.1 | AK024177 | UCSC_knowngene | 1670 |
| 0.9769877 | ENST00000420096 | Z83851.4 | Ensembl | 544 |
| 0.9775637 | ENST00000366224 | RP11-571E6.3 | Ensembl | 477 |
| 0.9781537 | ENST00000449215 | RP11-344F13.1 | Ensembl | 282 |
| 0.9784202 | ENST00000438148 | AC104655.3 | Ensembl | 415 |
| 0.9787444 | ENST00000442260 | RP11-284P20.3 | Ensembl | 436 |
| 0.980787 | NR_027029 | MGC34034 | RefSeq_NR | 3517 |
| 0.9808449 | ENST00000458683 | RP11-108M21.1 | Ensembl | 858 |
| 0.980921 | ENST00000430449 | CTA-342B11.1 | Ensembl | 473 |
| 0.9816802 | ENST00000423389 | AP001342.1 | Ensembl | 525 |
| 0.9825517 | ENST00000452288 | RP11-165D7.3 | Ensembl | 413 |
| 0.9831019 | ENST00000455894 | RP11-521H3.1 | Ensembl | 324 |
| 0.9833026 | ENST00000450944 | AC023115.4 | Ensembl | 574 |
| 0.9838568 | ENST00000437712 | RP11-168K11.3 | Ensembl | 3043 |
| 0.984074 | NR_026760 | BASE | RefSeq_NR | 1203 |
| 0.9843906 | ENST00000435157 | RP11-344B5.3 | Ensembl | 395 |
| 0.9844493 | ENST00000444986 | HCG14 | Ensembl | 413 |
| 0.985458 | ENST00000449711 | AP000344.3 | Ensembl | 917 |
| 0.9861935 | ENST00000435552 | RP3-340N1.6 | Ensembl | 480 |
| 0.9862876 | uc002ree.1 | AK090620 | UCSC_knowngene | 2098 |
| 0.9864916 | ENST00000449072 | RP11-518I13.1 | Ensembl | 533 |
| 0.9866051 | ENST00000454113 | RP6-1O2.1 | Ensembl | 611 |
| 0.9867837 | uc001ihe.3 | LOC100216001 | UCSC_knowngene | 2553 |
| 0.9880109 | uc003kbl.1 | AK123868 | UCSC_knowngene | 2767 |
| 0.988224 | ENST00000442188 | RP11-107I14.2 | Ensembl | 382 |
| 0.9882619 | ENST00000441348 | RP11-472G21.2 | Ensembl | 525 |
| 0.9883066 | ENST00000441942 | AC002511.1 | Ensembl | 474 |
| 0.9889271 | ENST00000453951 | AC104135.4 | Ensembl | 1266 |
| 0.9900683 | NR_033374 | LOC100289019 | RefSeq_NR | 3842 |
| 0.9912261 | ENST00000452402 | RP11-250B2.3 | Ensembl | 682 |
| 0.991421 | ENST00000429099 | RP11-117D22.1 | Ensembl | 383 |
| 0.9918515 | ENST00000421848 | RP11-374M1.4 | Ensembl | 492 |
| 0.9930279 | ENST00000412512 | RP5-1003J2.4 | Ensembl | 221 |
| 0.994067 | ENST00000510784 | AL160400.1 | Ensembl | 1029 |
| 0.994432 | ENST00000443593 | RP1-212P9.2 | Ensembl | 1746 |
| 0.9951761 | ENST00000416894 | RP11-54A4.8 | Ensembl | 566 |
| 0.9969341 | uc010tgn.1 | XTP6 | UCSC_knowngene | 1348 |
| 0.9970877 | AK057967 |  | misc_RNA | 1871 |
| 0.9972005 | ENST00000421320 | RP11-124O11.1 | Ensembl | 1714 |
| 0.997434 | ENST00000422697 | AC004535.2 | Ensembl | 449 |
| 0.9982831 | ENST00000449749 | AC068580.7 | Ensembl | 338 |
| 0.9985239 | ENST00000434627 | RP11-195F19.9 | Ensembl | 665 |
| 0.9986841 | ENST00000417636 | RP5-857K21.1 | Ensembl | 842 |
| 0.9995433 | ENST00000435559 | RP11-86H7.7 | Ensembl | 2272 |
| 0.9999299 | ENST00000430058 | RP11-23B15.1 | Ensembl | 204 |
| 0.9999978 | ENST00000421181 | AC080094.1 | Ensembl | 201 |
| 1.0007196 | ENST00000420406 | RP4-740C4.4 | Ensembl | 371 |
| 1.0007302 | ENST00000420693 | RP11-290D2.3 | Ensembl | 310 |
| 1.0012603 | ENST00000441036 | AC104782.3 | Ensembl | 581 |
| 1.0024944 | ENST00000439570 | RP11-61J19.2 | Ensembl | 359 |
| 1.0028613 | uc003qje.1 | AK057490 | UCSC_knowngene | 1900 |
| 1.0028693 | ENST00000434493 | RP1-177I10.1 | Ensembl | 442 |
| 1.0033384 | ENST00000420601 | RP11-235G24.3 | Ensembl | 379 |
| 1.0034209 | ENST00000417299 | RP1-278O22.1 | Ensembl | 438 |
| 1.0034303 | NR_024090 | C21orf131 | RefSeq_NR | 2014 |
| 1.0037241 | ENST00000420522 | RP1-37J18.2 | Ensembl | 682 |
| 1.0046254 | ENST00000450216 | CTA-150C2.13 | Ensembl | 678 |
| 1.0047355 | ENST00000422600 | AC159540.3 | Ensembl | 555 |
| 1.0048817 | ENST00000412445 | RP11-815M8.1 | Ensembl | 605 |
| 1.0053088 | uc003pwa.2 | BC037331 | UCSC_knowngene | 2746 |
| 1.0056373 | uc001abo.2 | BC006361 | UCSC_knowngene | 1317 |
| 1.0057834 | ENST00000429281 | RP11-265P11.2 | Ensembl | 673 |
| 1.0062629 | ENST00000417460 | AC003986.7 | Ensembl | 692 |
| 1.0062655 | ENST00000426509 | RP11-442J21.1 | Ensembl | 295 |
| 1.0068796 | ENST00000445083 | AC019100.3 | Ensembl | 611 |
| 1.0084762 | ENST00000418415 | AC016912.3 | Ensembl | 570 |
| 1.0084899 | ENST00000422732 | RP11-314P12.2 | Ensembl | 2444 |
| 1.0095299 | ENST00000439050 | AC009299.3 | Ensembl | 2333 |
| 1.0095336 | AK025759 |  | NRED | 2209 |
| 1.0098732 | BC064349 |  | RNAdb | 1448 |
| 1.0100623 | ENST00000430123 | RP11-354K1.1 | Ensembl | 357 |
| 1.0107668 | uc004ebo.2 | LOC554203 | UCSC_knowngene | 2212 |
| 1.0113321 | ENST00000423663 | AC073218.2 | Ensembl | 1109 |
| 1.0115206 | ENST00000445437 | RP1-172N19.1 | Ensembl | 453 |
| 1.0117254 | ENST00000437145 | AC024028.1 | Ensembl | 437 |
| 1.0118566 | ENST00000434593 | RP11-557H15.4 | Ensembl | 534 |
| 1.0121068 | ENST00000399543 | AC025171.1 | Ensembl | 2423 |
| 1.0122241 | uc010zye.1 | LOC284749 | UCSC_knowngene | 3080 |
| 1.0122496 | ENST00000425325 | AC012354.6 | Ensembl | 2681 |
| 1.0131196 | NR_015395 | LOC541471 | RefSeq_NR | 809 |
| 1.0138566 | ENST00000443359 | RP11-570H19.2 | Ensembl | 237 |
| 1.013919 | ENST00000437838 | RP11-498J9.2 | Ensembl | 229 |
| 1.01411 | ENST00000412362 | RP11-236B18.2 | Ensembl | 380 |
| 1.014368 | L08443 |  | misc_RNA | 1241 |
| 1.0144504 | ENST00000448134 | RP4-811H24.9 | Ensembl | 687 |
| 1.014673 | G65685 |  | misc_RNA | 205 |
| 1.015611 | ENST00000426067 | RP11-253D19.2 | Ensembl | 378 |
| 1.0157806 | ENST00000484550 | AC093627.10 | Ensembl | 5008 |
| 1.0164305 | ENST00000432783 | RP11-569G13.2 | Ensembl | 1577 |
| 1.0169311 | ENST00000429070 | RP11-589B3.6 | Ensembl | 606 |
| 1.017045 | uc003nsj.1 | AK094433 | UCSC_knowngene | 1980 |
| 1.0176932 | ENST00000400430 | RP11-350A18.1 | Ensembl | 702 |
| 1.0196656 | ENST00000432296 | RP11-31E23.1 | Ensembl | 930 |
| 1.0216756 | ENST00000444217 | AC013472.6 | Ensembl | 407 |
| 1.0221497 | ENST00000440714 | AF064858.10 | Ensembl | 292 |
| 1.0224524 | ENST00000450653 | AP000431.1 | Ensembl | 790 |
| 1.0224596 | AK055120 |  | RNAdb | 2021 |
| 1.02283 | ENST00000451579 | AC009878.2 | Ensembl | 606 |
| 1.0229015 | ENST00000439575 | RP11-118K6.2 | Ensembl | 608 |
| 1.0230439 | ENST00000433065 | AC007422.1 | Ensembl | 1008 |
| 1.0231625 | ENST00000424689 | RP11-431K24.4 | Ensembl | 477 |
| 1.0232477 | ENST00000422994 | RP11-551M15.1 | Ensembl | 650 |
| 1.0240063 | ENST00000436804 | XXbac-BPG13B8.10 | Ensembl | 877 |
| 1.0252103 | ENST00000444374 | RP11-316P17.2 | Ensembl | 822 |
| 1.0259959 | ENST00000452377 | RP11-399H11.3 | Ensembl | 527 |
| 1.027347 | ENST00000447028 | AP003774.1 | Ensembl | 672 |
| 1.0284146 | uc002ubt.1 | AK027541 | UCSC_knowngene | 2013 |
| 1.0287866 | NR_027082 | SFTA1P | RefSeq_NR | 693 |
| 1.0302573 | uc001ker.1 | BC082979 | UCSC_knowngene | 888 |
| 1.0308368 | ENST00000433036 | AC133528.2 | Ensembl | 415 |
| 1.0316536 | NR_024493 | NCRNA00087 | RefSeq_NR | 2808 |
| 1.0323416 | ENST00000449990 | RP11-555F9.2 | Ensembl | 791 |
| 1.0325714 | ENST00000443621 | RP11-30C8.2 | Ensembl | 282 |
| 1.0330569 | ENST00000432535 | RP11-456A18.1 | Ensembl | 477 |
| 1.0337567 | ENST00000440268 | AC011995.2 | Ensembl | 472 |
| 1.0343923 | ENST00000438753 | RP11-526P5.2 | Ensembl | 918 |
| 1.0355809 | ENST00000439434 | RP11-265D19.6 | Ensembl | 405 |
| 1.0360514 | AB073661 |  | NRED | 2686 |
| 1.0365421 | NR_003261 | CDRT15P | RefSeq_NR | 1101 |
| 1.0373908 | ENST00000412571 | RP1-122P22.2 | Ensembl | 415 |
| 1.038797 | ENST00000454922 | AC005154.5 | Ensembl | 752 |
| 1.0397126 | ENST00000440750 | RP11-534L6.2 | Ensembl | 335 |
| 1.0399281 | ENST00000436340 | RP11-195B3.1 | Ensembl | 463 |
| 1.0410795 | uc002wrm.1 | BC090059 | UCSC_knowngene | 1294 |
| 1.0413762 | ENST00000443123 | AC006227.1 | Ensembl | 860 |
| 1.0420742 | ENST00000434081 | NCRNA00163 | Ensembl | 2155 |
| 1.0432729 | ENST00000455973 | RP11-359N11.1 | Ensembl | 470 |
| 1.0447424 | ENST00000416595 | RP3-425P12.1 | Ensembl | 430 |
| 1.0448274 | uc010gbl.1 | BC048109 | UCSC_knowngene | 1564 |
| 1.0474627 | ENST00000458662 | RP11-122M14.3 | Ensembl | 174 |
| 1.0493097 | ENST00000434983 | RP11-309H21.2 | Ensembl | 299 |
| 1.0500653 | ENST00000433357 | RP11-255A11.21 | Ensembl | 902 |
| 1.0502469 | ENST00000456301 | RP3-341D10.4 | Ensembl | 3216 |
| 1.0508337 | ENST00000454600 | RP4-723E3.1 | Ensembl | 745 |
| 1.0508463 | ENST00000428930 | BX571672.1 | Ensembl | 614 |
| 1.0512426 | ENST00000437559 | RP11-456H18.2 | Ensembl | 784 |
| 1.0513317 | NR_027091 | LOC284798 | RefSeq_NR | 3159 |
| 1.0516794 | NR_029407 | LOC642361 | RefSeq_NR | 1712 |
| 1.051847 | ENST00000446989 | AL845154.2 | Ensembl | 514 |
| 1.0521513 | ENST00000418242 | RP11-148B18.3 | Ensembl | 480 |
| 1.0525502 | ENST00000455671 | AC051649.16 | Ensembl | 315 |
| 1.0538367 | ENST00000412174 | GS1-122H1.1 | Ensembl | 628 |
| 1.0539739 | ENST00000450784 | RP11-452F19.3 | Ensembl | 543 |
| 1.0543871 | ENST00000426653 | AC023490.2 | Ensembl | 853 |
| 1.0555795 | ENST00000416473 | RP11-439K3.1 | Ensembl | 444 |
| 1.0557109 | NR_015365 | LOC553137 | RefSeq_NR | 2491 |
| 1.0559303 | ENST00000420825 | RP1-251M9.2 | Ensembl | 537 |
| 1.0571639 | ENST00000419863 | RP5-1164C1.2 | Ensembl | 168 |
| 1.0572815 | ENST00000451362 | RP11-139I14.2 | Ensembl | 5586 |
| 1.0587696 | ENST00000439302 | BX571672.5 | Ensembl | 1163 |
| 1.05923 | uc002rng.2 | AK123934 | UCSC_knowngene | 1512 |
| 1.0597228 | ENST00000435492 | RP11-317P15.3 | Ensembl | 259 |
| 1.0601391 | ENST00000443205 | AC096669.3 | Ensembl | 1539 |
| 1.0601463 | ENST00000454056 | GS1-756B1.2 | Ensembl | 365 |
| 1.0602177 | ENST00000442852 | XXbac-BPG27H4.8 | Ensembl | 799 |
| 1.0603374 | ENST00000458351 | AC020601.1 | Ensembl | 1269 |
| 1.0608489 | ENST00000434020 | AC093609.1 | Ensembl | 952 |
| 1.0608607 | ENST00000465755 | AC093627.7 | Ensembl | 759 |
| 1.0621889 | ENST00000420169 | RP11-49G10.8 | Ensembl | 570 |
| 1.0628088 | ENST00000429469 | RP11-553K8.2 | Ensembl | 363 |
| 1.0632714 | ENST00000458170 | RP11-69A21.2 | Ensembl | 451 |
| 1.0634611 | ENST00000412422 | RP4-562J12.2 | Ensembl | 2290 |
| 1.0646335 | ENST00000446579 | RP11-309H8.3 | Ensembl | 734 |
| 1.065037 | NR_002733 | DGCR5 | RefSeq_NR | 5427 |
| 1.0652261 | ENST00000456848 | RP11-270C18.2 | Ensembl | 643 |
| 1.0680254 | ENST00000428814 | RP11-432J24.2 | Ensembl | 297 |
| 1.0684399 | uc001xyn.2 | BC036259 | UCSC_knowngene | 1375 |
| 1.0697769 | ENST00000438401 | RP5-845O24.8 | Ensembl | 378 |
| 1.070194 | ENST00000423187 | RP11-99H8.1 | Ensembl | 432 |
| 1.0707653 | ENST00000413708 | AC096669.1 | Ensembl | 616 |
| 1.0731544 | ENST00000417473 | AC099344.4 | Ensembl | 499 |
| 1.0733549 | ENST00000442627 | AC118653.2 | Ensembl | 561 |
| 1.0751724 | ENST00000450750 | RP4-754E20__A.5 | Ensembl | 951 |
| 1.0757436 | ENST00000416279 | AL133249.1 | Ensembl | 418 |
| 1.0757594 | ENST00000444259 | RP5-1112D6.4 | Ensembl | 557 |
| 1.0762494 | ENST00000445172 | RP1-153P14.3 | Ensembl | 735 |
| 1.0767158 | ENST00000444731 | RP11-552E20.1 | Ensembl | 539 |
| 1.0768256 | ENST00000451774 | AC074093.1 | Ensembl | 2098 |
| 1.0768665 | ENST00000436430 | RP11-245J24.1 | Ensembl | 556 |
| 1.0769031 | ENST00000445589 | RP5-859D4.3 | Ensembl | 932 |
| 1.0771594 | uc001vlm.1 | AF339814 | UCSC_knowngene | 1670 |
| 1.0776693 | ENST00000419531 | RP4-794H19.2 | Ensembl | 2273 |
| 1.0783193 | ENST00000455487 | RP11-54H7.2 | Ensembl | 698 |
| 1.0792563 | uc004ebr.1 | AK057701 | UCSC_knowngene | 2597 |
| 1.0796357 | uc003ajj.2 | TUG1 | UCSC_knowngene | 5561 |
| 1.079644 | ENST00000426353 | RP11-276H7.3 | Ensembl | 544 |
| 1.0814277 | ENST00000441170 | AC092661.2 | Ensembl | 1663 |
| 1.0833823 | ENST00000447876 | AC018730.1 | Ensembl | 607 |
| 1.0836894 | ENST00000422415 | AC010145.4 | Ensembl | 517 |
| 1.0847411 | uc003zzp.1 | BC067112 | UCSC_knowngene | 1868 |
| 1.0847796 | ENST00000422226 | GS1-421I3.4 | Ensembl | 279 |
| 1.0848551 | ENST00000435092 | RP11-408N14.1 | Ensembl | 443 |
| 1.0848798 | NR_024507 | LOC646982 | RefSeq_NR | 3972 |
| 1.0852323 | ENST00000413645 | AP000473.5 | Ensembl | 550 |
| 1.0863255 | ENST00000450776 | AP000343.2 | Ensembl | 346 |
| 1.0869198 | uc001vii.1 | AK057471 | UCSC_knowngene | 1878 |
| 1.0872918 | NR_015352 | CECR7 | RefSeq_NR | 2726 |
| 1.0874009 | uc004arq.1 | BC128384 | UCSC_knowngene | 1184 |
| 1.0877603 | ENST00000422346 | AC008280.4 | Ensembl | 732 |
| 1.0886554 | uc002hrd.1 | FLJ43826 | UCSC_knowngene | 3580 |
| 1.0916264 | AK024457 |  | NRED | 4665 |
| 1.095107 | ENST00000413103 | RP11-148B18.1 | Ensembl | 212 |
| 1.0958726 | uc003bcm.2 | BC038245 | UCSC_knowngene | 1074 |
| 1.0968602 | ENST00000423162 | RP11-508N22.8 | Ensembl | 790 |
| 1.097868 | ENST00000430364 | XXbac-BPG181B23.4 | Ensembl | 2141 |
| 1.0982307 | uc003qmi.2 | BC041998 | UCSC_knowngene | 2592 |
| 1.098421 | ENST00000450618 | RP3-340B19.3 | Ensembl | 595 |
| 1.1000341 | ENST00000415101 | RP11-402G3.5 | Ensembl | 404 |
| 1.1001226 | NR_002724 | MBL1P | RefSeq_NR | 1424 |
| 1.1018346 | ENST00000441329 | RP11-395L14.16 | Ensembl | 413 |
| 1.1021107 | ENST00000430562 | NCRNA00103 | Ensembl | 984 |
| 1.1023571 | ENST00000442403 | AC007064.24 | Ensembl | 500 |
| 1.1025265 | uc004bad.1 | AK057451 | UCSC_knowngene | 1544 |
| 1.1028714 | ENST00000439926 | RP11-402P6.11 | Ensembl | 220 |
| 1.103671 | ENST00000420389 | RP1-290I10.3 | Ensembl | 539 |
| 1.1037261 | ENST00000413945 | C6orf155 | Ensembl | 662 |
| 1.1042011 | ENST00000429998 | RP11-513I15.6 | Ensembl | 4785 |
| 1.1046726 | ENST00000420156 | RP4-543J13.1 | Ensembl | 332 |
| 1.1051227 | ENST00000417957 | RP11-375H17.1 | Ensembl | 451 |
| 1.1053966 | ENST00000422638 | RP4-656G21.1 | Ensembl | 498 |
| 1.1056089 | ENST00000443778 | RP11-760M1.2 | Ensembl | 398 |
| 1.1059351 | ENST00000431343 | AC005703.3 | Ensembl | 1773 |
| 1.1059919 | AF088026 |  | RNAdb | 703 |
| 1.1078376 | uc004ads.2 | LOC643648 | UCSC_knowngene | 1168 |
| 1.1078899 | ENST00000414677 | RP5-828H9.3 | Ensembl | 442 |
| 1.1081942 | ENST00000437867 | RP11-478K15.2 | Ensembl | 266 |
| 1.1090474 | ENST00000422194 | CXorf49B | Ensembl | 697 |
| 1.1093023 | uc001gyh.2 | LOC641515 | UCSC_knowngene | 2796 |
| 1.1098578 | ENST00000422129 | BX571672.2 | Ensembl | 697 |
| 1.1099468 | ENST00000433280 | RP11-547C18.3 | Ensembl | 431 |
| 1.1106028 | ENST00000416002 | AF240627.2 | Ensembl | 879 |
| 1.1107708 | uc002rop.1 | LOC285045 | UCSC_knowngene | 868 |
| 1.1111091 | uc003qev.1 | AJ606331 | UCSC_knowngene | 2477 |
| 1.1117534 | ENST00000440255 | RP1-213J1P__B.1 | Ensembl | 819 |
| 1.1118666 | ENST00000419499 | RP11-459P23.1 | Ensembl | 3340 |
| 1.1131249 | ENST00000436510 | RP11-65J3.3 | Ensembl | 1846 |
| 1.1138024 | ENST00000452500 | AP000472.3 | Ensembl | 530 |
| 1.1141866 | ENST00000428766 | RP11-57H14.3 | Ensembl | 317 |
| 1.11594 | ENST00000441217 | AC073046.25 | Ensembl | 390 |
| 1.1174627 | uc002rse.2 | DKFZp781C08198 | UCSC_knowngene | 1809 |
| 1.118074 | ENST00000456265 | RP5-1069C8.2 | Ensembl | 3033 |
| 1.1188616 | ENST00000456414 | RP5-1086K13.1 | Ensembl | 2295 |
| 1.1191768 | ENST00000428569 | RP11-541F9.1 | Ensembl | 583 |
| 1.1205128 | ENST00000431017 | RP11-252P19.2 | Ensembl | 359 |
| 1.1207259 | uc009wxq.1 | BC039493 | UCSC_knowngene | 538 |
| 1.1208104 | uc001hwe.2 | BC040195 | UCSC_knowngene | 2856 |
| 1.120971 | NR_027036 | LOC100271722 | RefSeq_NR | 1625 |
| 1.1214849 | ENST00000455754 | AC106875.1 | Ensembl | 1059 |
| 1.1219884 | ENST00000447179 | RP11-146I2.2 | Ensembl | 177 |
| 1.1225486 | ENST00000457890 | AC063976.3 | Ensembl | 381 |
| 1.1271122 | ENST00000419003 | RP4-779E11.3 | Ensembl | 480 |
| 1.1285015 | ENST00000415932 | RP11-199O14.1 | Ensembl | 1484 |
| 1.1305164 | uc002qym.1 | AK124409 | UCSC_knowngene | 2130 |
| 1.1326718 | ENST00000429853 | RP4-726N1.2 | Ensembl | 400 |
| 1.1336582 | ENST00000439839 | AC004485.3 | Ensembl | 527 |
| 1.1350271 | AV714079 |  | RNAdb | 721 |
| 1.1353693 | ENST00000446689 | RP11-78J21.4 | Ensembl | 721 |
| 1.1365449 | ENST00000452701 | AC011995.1 | Ensembl | 574 |
| 1.1383406 | ENST00000449463 | RP3-390M24.1 | Ensembl | 387 |
| 1.1394816 | ENST00000423442 | RP11-168P6.1 | Ensembl | 1058 |
| 1.1399139 | ENST00000450640 | RP5-1022P6.4 | Ensembl | 358 |
| 1.1412599 | ENST00000442155 | GS1-433O24.1 | Ensembl | 382 |
| 1.1415402 | ENST00000412656 | AC011718.2 | Ensembl | 727 |
| 1.142892 | ENST00000425609 | RP11-460G11.1 | Ensembl | 547 |
| 1.1432372 | ENST00000452944 | RP1-230L10.1 | Ensembl | 719 |
| 1.1432831 | ENST00000457669 | AP000696.2 | Ensembl | 1133 |
| 1.1447281 | ENST00000447956 | RP11-128M1.1 | Ensembl | 415 |
| 1.1453451 | uc002jil.2 | BC094791 | UCSC_knowngene | 3088 |
| 1.1462146 | NR_026762 | C10orf75 | RefSeq_NR | 1003 |
| 1.1462832 | ENST00000431729 | RP11-191N8.2 | Ensembl | 423 |
| 1.1466957 | ENST00000440932 | RP11-253D19.1 | Ensembl | 541 |
| 1.1485758 | ENST00000421002 | RP11-380B4.2 | Ensembl | 903 |
| 1.1487297 | ENST00000452690 | RP11-320G24.1 | Ensembl | 3000 |
| 1.149308 | ENST00000451285 | RP11-401E14.2 | Ensembl | 845 |
| 1.1502304 | ENST00000466530 | RP3-477O4.15 | Ensembl | 3965 |
| 1.1513803 | ENST00000420404 | AC010745.2 | Ensembl | 839 |
| 1.1526245 | uc001efz.2 | BC043254 | UCSC_knowngene | 1395 |
| 1.1528833 | uc002sdd.2 | BC041356 | UCSC_knowngene | 1720 |
| 1.153295 | ENST00000435106 | RP11-271F18.2 | Ensembl | 1251 |
| 1.1538768 | ENST00000453395 | LA16c-83F12.6 | Ensembl | 624 |
| 1.1564799 | ENST00000421642 | RP4-715N11.2 | Ensembl | 486 |
| 1.1615397 | NR_027002 | LOC388692 | RefSeq_NR | 12267 |
| 1.1621697 | ENST00000421572 | DGCR5 | Ensembl | 568 |
| 1.1625124 | ENST00000446754 | RP11-321L2.2 | Ensembl | 689 |
| 1.1629745 | ENST00000366097 | RP11-560A15.3 | Ensembl | 770 |
| 1.1640808 | ENST00000430633 | NCRNA00094 | Ensembl | 2239 |
| 1.1656003 | uc003adi.2 | CR936633 | UCSC_knowngene | 5051 |
| 1.1678572 | ENST00000424521 | RP3-453D15.1 | Ensembl | 138 |
| 1.1679438 | ENST00000412946 | RP11-290D2.4 | Ensembl | 225 |
| 1.1683101 | ENST00000451066 | AC009473.1 | Ensembl | 692 |
| 1.1692299 | ENST00000438659 | AC008281.1 | Ensembl | 613 |
| 1.171692 | ENST00000442864 | U91324.1 | Ensembl | 447 |
| 1.1736863 | ENST00000456235 | AC087393.1 | Ensembl | 750 |
| 1.1743446 | ENST00000451766 | RP11-443B7.1 | Ensembl | 426 |
| 1.1745153 | ENST00000431637 | NCRNA00210 | Ensembl | 1586 |
| 1.1749681 | ENST00000416924 | RP11-484I6.6 | Ensembl | 361 |
| 1.175053 | ENST00000417161 | RP11-554K11.2 | Ensembl | 440 |
| 1.1792608 | ENST00000452477 | RP11-292F9.2 | Ensembl | 528 |
| 1.1801015 | ENST00000435469 | AC005332.1 | Ensembl | 707 |
| 1.1808736 | ENST00000414039 | RP11-148B18.4 | Ensembl | 592 |
| 1.1812121 | uc004are.1 | AK095132 | UCSC_knowngene | 2745 |
| 1.1819197 | DQ860411 |  | RNAdb | 773 |
| 1.1841683 | ENST00000454262 | RP11-102N11.1 | Ensembl | 335 |
| 1.185586 | AK057050 |  | NRED | 1971 |
| 1.1870937 | ENST00000413637 | RP11-315H15.1 | Ensembl | 657 |
| 1.1873721 | ENST00000441660 | RP5-827L5.1 | Ensembl | 391 |
| 1.1874488 | ENST00000413291 | RP11-555J4.3 | Ensembl | 839 |
| 1.1881553 | ENST00000417084 | RP11-6J21.2 | Ensembl | 917 |
| 1.190234 | ENST00000451570 | RP11-473M10.2 | Ensembl | 415 |
| 1.1904576 | ENST00000398216 | RP11-54O7.2 | Ensembl | 443 |
| 1.1906178 | ENST00000359838 | RP3-337D23.3 | Ensembl | 453 |
| 1.1970892 | uc002wvk.2 | BC036544 | UCSC_knowngene | 1212 |
| 1.1992875 | ENST00000425109 | RP4-704D21.2 | Ensembl | 727 |
| 1.1993204 | ENST00000426713 | NCRNA00116 | Ensembl | 461 |
| 1.1993886 | ENST00000433572 | RP11-542K23.7 | Ensembl | 830 |
| 1.2001529 | ENST00000442416 | AC009502.3 | Ensembl | 361 |
| 1.2053867 | ENST00000443548 | BX004987.4 | Ensembl | 681 |
| 1.2130818 | ENST00000433152 | RP11-222A11.1 | Ensembl | 744 |
| 1.2134773 | ENST00000445035 | AC005863.2 | Ensembl | 627 |
| 1.2149971 | ENST00000457668 | AC007381.3 | Ensembl | 2005 |
| 1.2171181 | ENST00000417264 | RP11-533K9.2 | Ensembl | 155 |
| 1.2178537 | ENST00000412197 | AC091705.1 | Ensembl | 209 |
| 1.2179484 | ENST00000423877 | RP11-453N3.2 | Ensembl | 493 |
| 1.2181775 | ENST00000423907 | RP4-655J12.4 | Ensembl | 454 |
| 1.2185457 | ENST00000427722 | RP11-509J21.3 | Ensembl | 537 |
| 1.2212637 | ENST00000433388 | RP1-137D17.1 | Ensembl | 588 |
| 1.2226754 | uc001ejn.1 | BC070106 | UCSC_knowngene | 1888 |
| 1.2239281 | ENST00000456103 | C6orf38 | Ensembl | 537 |
| 1.226591 | ENST00000447524 | XXyac-YM21GA2.4 | Ensembl | 452 |
| 1.2266398 | ENST00000427547 | AL162400.1 | Ensembl | 157 |
| 1.2267654 | ENST00000437696 | RP11-359G22.2 | Ensembl | 565 |
| 1.2274512 | ENST00000419013 | AC096570.2 | Ensembl | 569 |
| 1.227698 | ENST00000455005 | RP5-1120P11.3 | Ensembl | 1608 |
| 1.2286173 | ENST00000434596 | RP11-503C24.3 | Ensembl | 473 |
| 1.2289519 | ENST00000452982 | RP11-431K24.3 | Ensembl | 497 |
| 1.229555 | ENST00000419207 | RP11-160N1.9 | Ensembl | 719 |
| 1.2323435 | uc001usr.2 | CR592782 | UCSC_knowngene | 2567 |
| 1.232817 | uc001jzw.1 | AK126491 | UCSC_knowngene | 3842 |
| 1.2333745 | ENST00000426374 | RP1-135L22.1 | Ensembl | 387 |
| 1.2365118 | ENST00000425763 | AC107057.1 | Ensembl | 319 |
| 1.2406626 | uc004adg.1 | AK126080 | UCSC_knowngene | 3223 |
| 1.2412978 | ENST00000439904 | RP11-9E13.2 | Ensembl | 1780 |
| 1.2432687 | ENST00000442558 | RP11-147C23.1 | Ensembl | 479 |
| 1.2435926 | ENST00000430921 | RP11-66A2.1 | Ensembl | 372 |
| 1.2441168 | NR_026835 | FLJ37201 | RefSeq_NR | 3662 |
| 1.2461913 | ENST00000429269 | NCRNA00184 | Ensembl | 2270 |
| 1.2462121 | ENST00000430699 | BX004987.3 | Ensembl | 943 |
| 1.2484897 | ENST00000444744 | RP11-428O18.4 | Ensembl | 420 |
| 1.2488223 | ENST00000454935 | C10orf75 | Ensembl | 2551 |
| 1.2493095 | AL109791 |  | misc_RNA | 2110 |
| 1.2496919 | uc003ojj.2 | CR624291 | UCSC_knowngene | 2513 |
| 1.250477 | uc010pbe.1 | BC062745 | UCSC_knowngene | 1072 |
| 1.2530295 | ENST00000441160 | RP11-400N13.2 | Ensembl | 1148 |
| 1.2531004 | ENST00000453722 | AC007639.1 | Ensembl | 1747 |
| 1.2538707 | ENST00000418979 | RP4-673D20.3 | Ensembl | 1372 |
| 1.2543321 | ENST00000412485 | GS1-600G8.5 | Ensembl | 1497 |
| 1.2555793 | NR_027245 | C18orf20 | RefSeq_NR | 885 |
| 1.2559204 | ENST00000437831 | RP11-441L11.1 | Ensembl | 701 |
| 1.256264 | uc004dgj.1 | AK127697 | UCSC_knowngene | 2924 |
| 1.2596471 | uc003qwu.1 | AK055570 | UCSC_knowngene | 2282 |
| 1.2611818 | ENST00000435301 | RP11-112L6.3 | Ensembl | 733 |
| 1.2641864 | uc001kat.2 | CR592318 | UCSC_knowngene | 1146 |
| 1.2654304 | ENST00000451438 | RP11-168L22.2 | Ensembl | 391 |
| 1.2677195 | ENST00000417262 | RP11-480I12.3 | Ensembl | 748 |
| 1.2684346 | NR_027418 | LOC651250 | RefSeq_NR | 11210 |
| 1.2721156 | ENST00000445738 | AC007365.1 | Ensembl | 1386 |
| 1.2749318 | ENST00000436500 | RP11-514F8.2 | Ensembl | 370 |
| 1.2767781 | ENST00000450290 | AC084193.1 | Ensembl | 769 |
| 1.2770406 | ENST00000415629 | RP4-726F1.1 | Ensembl | 342 |
| 1.2771885 | NR_026903 | AMZ2P1 | RefSeq_NR | 3442 |
| 1.2788995 | ENST00000455253 | AF127936.3 | Ensembl | 743 |
| 1.2906287 | NR_015413 | LOC254312 | RefSeq_NR | 1127 |
| 1.2922677 | ENST00000439046 | AC019117.2 | Ensembl | 589 |
| 1.2966874 | ENST00000400817 | AC103965.1 | Ensembl | 2545 |
| 1.3006392 | ENST00000417989 | RP11-24H2.2 | Ensembl | 577 |
| 1.3034764 | ENST00000411690 | RP11-472K17.1 | Ensembl | 437 |
| 1.3069262 | ENST00000449772 | AC068535.3 | Ensembl | 493 |
| 1.3086809 | ENST00000412500 | RP5-885L7.10 | Ensembl | 540 |
| 1.3095752 | ENST00000425979 | AP000475.2 | Ensembl | 482 |
| 1.3106026 | ENST00000457813 | AC018685.2 | Ensembl | 368 |
| 1.3113022 | ENST00000453968 | AC002115.5 | Ensembl | 362 |
| 1.3117207 | ENST00000448431 | AC010744.1 | Ensembl | 303 |
| 1.3131991 | ENST00000458154 | XXbac-B33L19.4 | Ensembl | 446 |
| 1.3157811 | uc002yyn.1 | CR624487 | UCSC_knowngene | 1542 |
| 1.3171106 | ENST00000456072 | L29074.3 | Ensembl | 534 |
| 1.3202589 | uc001voi.1 | AK123584 | UCSC_knowngene | 2796 |
| 1.3262009 | ENST00000426187 | AC073133.2 | Ensembl | 432 |
| 1.3270073 | uc002wrh.3 | HSPC072 | UCSC_knowngene | 1951 |
| 1.3303017 | ENST00000413093 | RP11-8J9.6 | Ensembl | 488 |
| 1.3325152 | ENST00000440477 | RP11-46E17.6 | Ensembl | 770 |
| 1.3413323 | ENST00000427911 | AP000281.2 | Ensembl | 384 |
| 1.3453571 | ENST00000454780 | RP11-538C21.1 | Ensembl | 807 |
| 1.3458019 | ENST00000432067 | RP4-610C12.1 | Ensembl | 460 |
| 1.3474124 | ENST00000376445 | RP13-401N8.1 | Ensembl | 320 |
| 1.3481099 | uc003qis.2 | BC038188 | UCSC_knowngene | 1351 |
| 1.3482026 | ENST00000467630 | AC000123.3 | Ensembl | 461 |
| 1.3499119 | NR_026679 | NCRNA00032 | RefSeq_NR | 2913 |
| 1.350068 | ENST00000415655 | RP1-231P7P.1 | Ensembl | 577 |
| 1.3577626 | ENST00000417443 | NCRNA00086 | Ensembl | 2913 |
| 1.3581886 | ENST00000446589 | RP11-442O18.2 | Ensembl | 450 |
| 1.3600872 | ENST00000477499 | AC108004.2 | Ensembl | 787 |
| 1.360239 | ENST00000435526 | RP11-414H17.6 | Ensembl | 409 |
| 1.361691 | uc002jix.2 | AK000477 | UCSC_knowngene | 1697 |
| 1.3621452 | ENST00000440100 | RP4-739H11.3 | Ensembl | 471 |
| 1.3690508 | ENST00000435810 | RP11-252P19.1 | Ensembl | 1122 |
| 1.3713766 | ENST00000415801 | RP11-276H19.1 | Ensembl | 4387 |
| 1.376415 | ENST00000452667 | NHEDC1P1 | Ensembl | 1524 |
| 1.3786127 | ENST00000452795 | RP4-798A10.4 | Ensembl | 342 |
| 1.379008 | ENST00000447250 | RP4-625H18.2 | Ensembl | 1016 |
| 1.3849027 | ENST00000416835 | AC110299.2 | Ensembl | 822 |
| 1.3952214 | ENST00000441203 | RP3-416J7.2 | Ensembl | 844 |
| 1.4057121 | ENST00000418403 | RP11-462G2.1 | Ensembl | 397 |
| 1.4093719 | ENST00000423628 | CTA-215D11.3 | Ensembl | 410 |
| 1.4110999 | uc003qmb.2 | BC043173 | UCSC_knowngene | 1473 |
| 1.4226665 | uc001ksy.1 | AX747408 | UCSC_knowngene | 2222 |
| 1.4252016 | ENST00000443716 | RP11-165J3.5 | Ensembl | 641 |
| 1.4269641 | ENST00000424980 | RP11-375O18.2 | Ensembl | 328 |
| 1.4328387 | uc001jxd.1 | AK023445 | UCSC_knowngene | 1860 |
| 1.4340969 | ENST00000412060 | Z83851.1 | Ensembl | 475 |
| 1.4428654 | NR_026885 | LOC100270804 | RefSeq_NR | 2034 |
| 1.447361 | ENST00000447848 | RP11-394G3.2 | Ensembl | 777 |
| 1.4488331 | ENST00000445428 | RP1-206D15.3 | Ensembl | 1491 |
| 1.4509613 | ENST00000450811 | RP4-771M4.3 | Ensembl | 447 |
| 1.4531781 | ENST00000445225 | RP11-277L2.2 | Ensembl | 5730 |
| 1.4583779 | uc002rby.2 | BC035112 | UCSC_knowngene | 3502 |
| 1.460032 | ENST00000447315 | RP11-552E20.4 | Ensembl | 824 |
| 1.4606281 | uc002khh.2 | LOC388312 | UCSC_knowngene | 4812 |
| 1.4839819 | uc001guy.2 | DM004468 | UCSC_knowngene | 109 |
| 1.488896 | ENST00000455771 | RP11-347K2.2 | Ensembl | 439 |
| 1.4919032 | ENST00000446593 | AC093642.6 | Ensembl | 561 |
| 1.4924819 | ENST00000435735 | RP11-180A14.5 | Ensembl | 2625 |
| 1.510188 | uc003mud.2 | MGC39372 | UCSC_knowngene | 1251 |
| 1.5197721 | ENST00000414159 | AC114776.3 | Ensembl | 4394 |
| 1.5204098 | ENST00000445279 | AC113607.2 | Ensembl | 694 |
| 1.5458918 | ENST00000441809 | CTA-520D8.2 | Ensembl | 1007 |
| 1.5513474 | uc001eiw.1 | CR936796 | UCSC_knowngene | 4096 |
| 1.5608773 | ENST00000366185 | RP11-258C19.5 | Ensembl | 1063 |
| 1.563107 | ENST00000434300 | RP5-1011O1.2 | Ensembl | 396 |
| 1.5704008 | ENST00000452578 | RP11-20J15.3 | Ensembl | 915 |
| 1.5750897 | ENST00000442382 | RP4-781K5.6 | Ensembl | 3564 |
| 1.583676 | uc002rdf.2 | BC068572 | UCSC_knowngene | 3008 |
| 1.5979497 | uc002tcj.1 | AK096621 | UCSC_knowngene | 2436 |
| 1.6061811 | ENST00000444356 | AL050303.7 | Ensembl | 458 |
| 1.6256501 | AL832916 |  | RNAdb | 1390 |
| 1.6298439 | ENST00000428292 | RP11-18B16.2 | Ensembl | 1223 |
| 1.6316264 | ENST00000491363 | AC000123.2 | Ensembl | 4975 |
| 1.635826 | ENST00000427890 | RP11-101P17.6 | Ensembl | 621 |
| 1.6507915 | ENST00000449144 | RP11-384P7.5 | Ensembl | 1144 |
| 1.6708483 | ENST00000418196 | RP11-292F22.5 | Ensembl | 167 |
| 1.6752548 | uc001jah.1 | AK123067 | UCSC_knowngene | 1298 |
| 1.6843661 | ENST00000444137 | RP11-475D12.1 | Ensembl | 403 |
| 1.6854267 | NR_026790 | HCG11 | RefSeq_NR | 5688 |
| 1.6919244 | AK055023 |  | misc_RNA | 1974 |
| 1.6943542 | ENST00000431981 | RP11-112J3.16 | Ensembl | 407 |
| 1.7040394 | ENST00000442780 | RP11-560A15.4 | Ensembl | 377 |
| 1.715748 | ENST00000434292 | RP5-1077H22.1 | Ensembl | 403 |
| 1.7342269 | NR_026816 | PSORS1C3 | RefSeq_NR | 600 |
| 1.7478454 | ENST00000449954 | AL078621.3 | Ensembl | 745 |
| 1.761183 | AL110130 |  | misc_RNA | 2409 |
| 1.7725882 | ENST00000420710 | RP5-827L5.2 | Ensembl | 500 |
| 1.7820061 | ENST00000419956 | RP1-187N21.2 | Ensembl | 2279 |
| 1.7988439 | ENST00000437350 | RP5-843L14.1 | Ensembl | 298 |
| 1.8036258 | uc003gcl.1 | AX747945 | UCSC_knowngene | 2165 |
| 1.840826 | uc004bcy.1 | AK093363 | UCSC_knowngene | 2054 |
| 1.8516329 | NR_027087 | LOC284632 | RefSeq_NR | 1863 |
| 1.8964929 | ENST00000447720 | KB-1183D5.13 | Ensembl | 3215 |
| 1.9005189 | ENST00000429493 | RP11-220I1.1 | Ensembl | 4375 |
| 1.9284547 | ENST00000435444 | RP11-29B9.2 | Ensembl | 501 |
| 1.9301023 | uc001aio.1 | AK054708 | UCSC_knowngene | 1954 |
| 1.9776904 | ENST00000449713 | AP001065.15 | Ensembl | 912 |
| **2.0228474** | **uc003zfy.2** | **AY343902** | **UCSC_knowngene** | **581** |
| **2.03408** | **ENST00000452746** | **RP11-32F11.2** | **Ensembl** | **886** |
| **2.0427826** | **ENST00000421563** | **AC012506.4** | **Ensembl** | **541** |
| **2.0490355** | **uc001kyk.2** | **BC036309** | **UCSC_knowngene** | **2093** |
| **2.0610563** | **ENST00000420211** | **RP11-422P22.1** | **Ensembl** | **491** |
| **2.2628114** | **uc002nzf.1** | **AK128099** | **UCSC_knowngene** | **3312** |
| **2.4043955** | **uc001ggd.2** | **BC041451** | **UCSC_knowngene** | **1707** |
| **2.5595311** | **uc002rsu.1** | **CR600703** | **UCSC_knowngene** | **2156** |
| **2.6580433** | **NR_026651** | **DGCR10** | **RefSeq_NR** | **944** |

The bold indicates 9 differently expressed lncRNAs (≥ 2-fold, *P* < 0.05) between six PDAC samples and paired nontumor samples.

**Table S7** 222 differentially expressed mRNAs (≥ 2-fold, *P* < 0.05) between six PDAC samples and paired nontumor samples

| ***P*-value** | **Absolute Fold change([T1] vs [N1])** | **Regulation([T1] vs [N1])** | **seqname** | **GeneSymbol** | **RNAlength** |
| --- | --- | --- | --- | --- | --- |
| 0.006727856 | 33.80595192 | up | NM_002722 | PPY | 457 |
| 0.004501379 | 16.15646228 | up | NM_018951_Exon2- | NM_018951 | 1582 |
| 0.008443222 | 11.08962545 | up | NM_203451 | C13orf36 | 3219 |
| 0.000699974 | 9.194987827 | up | NM_001819 | CHGB | 2666 |
| 0.000484915 | 8.033222566 | up | NM_003695 | LY6D | 806 |
| 0.036028802 | 6.101522371 | up | NM_001135057 | LRRC15 | 5940 |
| 0.014317401 | 4.664030478 | up | NM_025216 | WNT10A | 2375 |
| 0.014982707 | 4.27758863 | up | NM_004626 | WNT11 | 1927 |
| 0.01989552 | 3.725613689 | up | NM_003469 | SCG2 | 2490 |
| 0.002682998 | 3.710084045 | up | NM_002846 | PTPRN | 3649 |
| 0.017205561 | 3.678409645 | up | NM_014951 | ZNF365 | 4223 |
| 0.019070318 | 3.582534785 | up | NM_020167 | NMUR2 | 2067 |
| 0.016006885 | 3.544333138 | up | NM_004004 | GJB2 | 2347 |
| 0.040590296 | 3.269550498 | up | NM_152739_Exon2- | NM_152739 | 1403 |
| 6.77183E-05 | 3.15542797 | up | NM_003378 | VGF | 2586 |
| 0.030392173 | 3.12183423 | up | NM_144613 | COX6B2 | 1679 |
| 0.019005875 | 2.980024983 | up | NM_000745 | CHRNA5 | 2468 |
| 0.040648731 | 2.9728177 | up | NM_001189 | NKX3-2 | 2241 |
| 0.021218406 | 2.862690039 | up | NM_153350 | FBXL16 | 3543 |
| 0.020872068 | 2.799322835 | up | NM_000475 | NR0B1 | 1591 |
| 0.014890402 | 2.758068861 | up | NM_000130 | F5 | 9179 |
| 0.021095783 | 2.757881353 | up | NM_018952_Exon4- | NM_018952 | 936 |
| 0.036620914 | 2.68196189 | up | NM_003013 | SFRP2 | 2005 |
| 0.043261433 | 2.673355887 | up | NM_173481 | C19orf21 | 2907 |
| 0.005899608 | 2.644513036 | up | NM_020655 | JPH3 | 3997 |
| 0.01152016 | 2.637997529 | up | NM_033260 | FOXQ1 | 2338 |
| 0.048303335 | 2.620228776 | up | NM_002638 | PI3 | 579 |
| 0.025237046 | 2.598464149 | up | NM_002421 | MMP1 | 2081 |
| 0.046420899 | 2.575147755 | up | NM_130441 | CLEC4C | 1314 |
| 0.040586174 | 2.557036927 | up | NM_021214 | FAM108C1 | 2408 |
| 0.006449651 | 2.554969737 | up | NM_024017 | HOXB9 | 2711 |
| 0.02059383 | 2.548319793 | up | NM_002147_Exon2- | NM_002147 | 1199 |
| 0.011396883 | 2.547918128 | up | NM_024017_Exon2- | NM_024017 | 1972 |
| 0.048749684 | 2.535937375 | up | NM_021920 | SCT | 514 |
| 0.002425795 | 2.522051364 | up | NM_002237 | KCNG1 | 2237 |
| 0.032223944 | 2.512177622 | up | NM_002275 | KRT15 | 1861 |
| 0.006507142 | 2.510878647 | up | NM_003528 | HIST2H2BE | 2223 |
| 0.025466529 | 2.502359975 | up | NM_013381 | TRHDE | 5552 |
| 0.041883359 | 2.487091922 | up | NM_004503_Exon2+ | NM_004503 | 1168 |
| 0.043720991 | 2.471902775 | up | NM_007350 | PHLDA1 | 5913 |
| 0.011306806 | 2.462347925 | up | NM_018665 | DDX43 | 2707 |
| 0.033794718 | 2.380013478 | up | NM_144577 | CCDC114 | 3234 |
| 0.031356894 | 2.369111581 | up | NM_001130448 | C15orf62 | 2503 |
| 0.041260313 | 2.335494988 | up | NM_001135610 | PRDM2 | 2715 |
| 0.003977331 | 2.331614431 | up | NM_000238 | KCNH2 | 3900 |
| 0.048300988 | 2.319380127 | up | NM_181608 | KRTAP19-2 | 159 |
| 0.020974887 | 2.308695838 | up | NM_000758 | CSF2 | 781 |
| 0.014907023 | 2.276522055 | up | NM_000474 | TWIST1 | 1669 |
| 0.027045758 | 2.230510917 | up | NM_020704 | FAM40B | 5115 |
| 0.029058334 | 2.229330322 | up | NM_006577 | B3GNT2 | 2788 |
| 0.036358166 | 2.219756014 | up | NM_138720 | HIST1H2BD | 829 |
| 0.0229608 | 2.213962199 | up | NM_001144757 | SCG5 | 1244 |
| 0.02191065 | 2.190385811 | up | NM_004015 | DMD | 4623 |
| 0.033136286 | 2.174260569 | up | NM_007289 | MME | 5710 |
| 0.025157787 | 2.119455068 | up | NM_002146_Exon4- | NM_002146 | 2311 |
| 0.042167657 | 2.106534495 | up | NM_016339 | RAPGEFL1 | 3727 |
| 0.022963205 | 2.079760039 | up | NM_001007538 | SHISA2 | 2889 |
| 0.047543782 | 2.066478552 | up | NM_006735_Exon2- | NM_006735 | 1111 |
| 0.04712288 | 2.060909237 | up | NM_206967 | C16orf74 | 899 |
| 0.012669092 | 2.056111262 | up | NM_199262 | SP6 | 3794 |
| 0.048105193 | 2.05581316 | up | NM_001080441 | TTC36 | 679 |
| 0.042216485 | 2.037072111 | up | NM_198488 | FAM83H | 5604 |
| 0.013436374 | 2.036742266 | up | NM_005842 | SPRY2 | 2126 |
| 0.009084071 | 2.032792901 | up | NM_013271 | PCSK1N | 990 |
| 0.032291136 | 2.01311207 | up | NM_003778 | B4GALT4 | 2283 |
| 0.038781439 | 2.007690181 | up | NM_001785 | CDA | 985 |
| 0.011985758 | 28.90142902 | down | NM_002054 | GCG | 1298 |
| 0.002036643 | 13.2013934 | down | NM_000518 | HBB | 626 |
| 0.000764124 | 8.882192581 | down | NM_000184 | HBG2 | 583 |
| 0.027173902 | 8.346364071 | down | NM_030754 | SAA2 | 545 |
| 0.000668468 | 7.85402841 | down | NM_002964 | S100A8 | 428 |
| 0.001670622 | 7.517035364 | down | NM_002965 | S100A9 | 586 |
| 0.00137732 | 6.125857362 | down | NM_000517 | HBA2 | 622 |
| 0.031819209 | 5.655504754 | down | NM_198492 | CLEC4G | 1377 |
| 0.008638236 | 4.902039462 | down | NM_002704 | PPBP | 1307 |
| 0.004849846 | 4.837707285 | down | NM_138296 | PTCRA | 1097 |
| 0.002258126 | 4.784464533 | down | NM_000558 | HBA1 | 576 |
| 0.002465103 | 4.531486184 | down | NM_020530 | OSM | 1880 |
| 0.000948279 | 4.492659114 | down | NM_003480 | MFAP5 | 2900 |
| 0.026495534 | 4.441923269 | down | NM_001645 | APOC1 | 464 |
| 0.000485866 | 4.373775745 | down | NM_003381 | VIP | 1618 |
| 0.002018815 | 4.306122602 | down | NM_032849 | C13orf33 | 2394 |
| 0.004999385 | 4.164160305 | down | NM_006418 | OLFM4 | 2844 |
| 0.000441231 | 4.070921738 | down | NM_000566 | FCGR1A | 2268 |
| 0.02685818 | 3.780886974 | down | NM_002988 | CCL18 | 793 |
| 0.002919468 | 3.704566557 | down | NM_003063 | SLN | 738 |
| 0.018027019 | 3.626168274 | down | NM_005860 | FSTL3 | 2525 |
| 0.015861232 | 3.5387629 | down | NM_024015 | HOXB4 | 2042 |
| 0.043575016 | 3.454674907 | down | NM_024501_intron1- | NM_024501 | 353 |
| 0.007739005 | 3.411750095 | down | NM_145260 | OSR1 | 1911 |
| 0.006598729 | 3.40241288 | down | NM_000491 | C1QB | 1044 |
| 0.000164505 | 3.394937508 | down | NM_002029 | FPR1 | 1334 |
| 0.003084355 | 3.38642341 | down | NM_002164 | IDO1 | 1572 |
| 0.000670779 | 3.358774022 | down | NM_022136 | SAMSN1 | 1893 |
| 0.000913568 | 3.333985868 | down | NM_172313 | CSF3R | 2583 |
| 0.011509949 | 3.306418441 | down | NM_007268 | VSIG4 | 1869 |
| 0.008233065 | 3.27861663 | down | NM_001295 | CCR1 | 2690 |
| 0.007727925 | 3.21281214 | down | NM_033423 | GZMH | 1046 |
| 0.005887036 | 3.155553529 | down | NM_001114735 | BCL2A1 | 955 |
| 0.018787941 | 3.138515846 | down | NM_003332 | TYROBP | 608 |
| 0.015594828 | 3.097030324 | down | NM_003974 | DOK2 | 1870 |
| 0.008548225 | 3.095368232 | down | NM_001100431 | VSIG4 | 1586 |
| 0.000580907 | 3.094604889 | down | NM_001017986 | FCGR1B | 1139 |
| 0.00248619 | 3.082498456 | down | NM_000905 | NPY | 576 |
| 0.027819416 | 3.074434012 | down | NM_206939 | MS4A7 | 2976 |
| 0.01072589 | 3.028531935 | down | NM_005601 | NKG7 | 826 |
| 0.009866236 | 2.956792879 | down | NM_000483 | APOC2 | 753 |
| 0.00896685 | 2.929333558 | down | NM_005621 | S100A12 | 466 |
| 0.007141871 | 2.921562862 | down | NM_203416 | CD163 | 4148 |
| 0.033170983 | 2.890228702 | down | NM_001928 | CFD | 1173 |
| 0.010390113 | 2.875760856 | down | NM_152852 | MS4A6A | 1564 |
| 0.00027766 | 2.87570613 | down | NM_021175 | HAMP | 430 |
| 0.001127405 | 2.839696104 | down | NM_000760 | CSF3R | 3003 |
| 0.002406789 | 2.806378086 | down | NM_002432 | MNDA | 1670 |
| 0.00209187 | 2.786345967 | down | NM_002445 | MSR1 | 2960 |
| 0.036216008 | 2.781736963 | down | NM_031950 | FGFBP2 | 1188 |
| 0.002647145 | 2.767289229 | down | NM_024015_Exon1- | NM_024015 | 519 |
| 0.021894062 | 2.73324816 | down | NM_004049 | BCL2A1 | 899 |
| 0.023744002 | 2.713910166 | down | NM_000803 | FOLR2 | 1145 |
| 0.007277182 | 2.677281338 | down | NM_001523 | HAS1 | 2116 |
| 0.001991116 | 2.673703925 | down | NM_002982 | CCL2 | 760 |
| 0.047000722 | 2.654612378 | down | NM_002993 | CXCL6 | 1677 |
| 0.01321129 | 2.65414906 | down | NM_022355 | DPEP2 | 1724 |
| 0.012589452 | 2.634949434 | down | NM_014470 | RND1 | 1665 |
| 0.012701839 | 2.617952408 | down | NM_015991 | C1QA | 1098 |
| 0.012240763 | 2.595382889 | down | NM_001166538 | LST1 | 560 |
| 0.005779097 | 2.584920323 | down | NM_003254 | TIMP1 | 931 |
| 0.001217446 | 2.569605577 | down | NM_001172132 | HCK | 2234 |
| 0.029444194 | 2.569501764 | down | NM_001098612 | SIGLEC14 | 2113 |
| 0.005621243 | 2.567807421 | down | NM_005849 | IGSF6 | 1058 |
| 0.00428713 | 2.564781988 | down | NM_001145645 | TNFSF13B | 1147 |
| 0.029791928 | 2.555865575 | down | NM_001144063 | OSBPL5 | 3700 |
| 0.016378328 | 2.554752203 | down | NM_021209 | NLRC4 | 3384 |
| 0.012015858 | 2.549617809 | down | NM_002619 | PF4 | 476 |
| 0.00503797 | 2.529444209 | down | NM_018965 | TREM2 | 1059 |
| 0.002683554 | 2.529348514 | down | NM_002727 | SRGN | 1270 |
| 0.001043315 | 2.489440633 | down | NM_001010933 | HGF | 1292 |
| 0.011612154 | 2.467426609 | down | NM_206938 | MS4A7 | 2889 |
| 0.003947791 | 2.461584235 | down | NM_001024465 | SOD2 | 1035 |
| 0.009013115 | 2.432091456 | down | NM_153225 | C8orf84 | 3804 |
| 0.025042363 | 2.419834474 | down | NM_002089 | CXCL2 | 1234 |
| 0.01120081 | 2.409698099 | down | NM_198565 | LRRC33 | 2488 |
| 0.004392683 | 2.396050025 | down | NM_001623 | AIF1 | 639 |
| 0.022335683 | 2.382797318 | down | NM_001081637 | LILRB1 | 2802 |
| 0.001289407 | 2.380539857 | down | NM_207581 | DUOXA2 | 1451 |
| 0.003811619 | 2.376850204 | down | NM_030984 | TBXAS1 | 1919 |
| 0.02046662 | 2.372399641 | down | NM_001736 | C5AR1 | 2342 |
| 0.012239713 | 2.371393454 | down | NM_002423 | MMP7 | 1147 |
| 0.008319754 | 2.360482611 | down | NM_004106 | FCER1G | 591 |
| 0.011665736 | 2.356847251 | down | NM_000631 | NCF4 | 1401 |
| 0.006270848 | 2.349758505 | down | NM_152942 | TNFRSF8 | 2361 |
| 0.003485932 | 2.340669018 | down | NM_001010919 | FAM26F | 1109 |
| 0.009912897 | 2.329959175 | down | NM_178012 | TUBB2B | 2019 |
| 0.004588132 | 2.329299222 | down | NM_001024466 | SOD2 | 918 |
| 0.013575914 | 2.328304149 | down | NM_152851 | MS4A6A | 1460 |
| 0.001073437 | 2.327547271 | down | NM_001024457 | RGPD1 | 6711 |
| 0.031455121 | 2.324448224 | down | NM_018690 | APOB48R | 3733 |
| 0.04980976 | 2.304136037 | down | NM_006741 | PPP1R1A | 1849 |
| 0.000345303 | 2.303596851 | down | NM_004417 | DUSP1 | 2040 |
| 0.003503574 | 2.295890108 | down | NM_004054 | C3AR1 | 1985 |
| 0.013833656 | 2.292947399 | down | NM_012429 | SEC14L2 | 4284 |
| 0.017864577 | 2.292460159 | down | NM_004877 | GMFG | 674 |
| 0.030100143 | 2.284760257 | down | NM_148975 | MS4A4A | 1543 |
| 0.007054307 | 2.281314888 | down | NM_001166002 | APOBEC3H | 1067 |
| 0.037442493 | 2.280320052 | down | NM_002507 | NGFR | 3420 |
| 0.014578194 | 2.275930085 | down | NM_000570 | FCGR3B | 2300 |
| 0.020987981 | 2.27232846 | down | NM_198482 | SH2D6 | 909 |
| 8.88298E-05 | 2.268539182 | down | NM_139018 | CD300LF | 1759 |
| 0.026955097 | 2.264514782 | down | NM_002989 | CCL21 | 878 |
| 0.007518865 | 2.26392732 | down | NM_205840 | LST1 | 609 |
| 0.018151545 | 2.262084256 | down | NM_022054 | KCNK13 | 2517 |
| 0.001664922 | 2.260338636 | down | NM_001025159 | CD74 | 1519 |
| 0.017847722 | 2.255744899 | down | NM_001007469 | HCST | 521 |
| 0.027970453 | 2.248696163 | down | NM_004364 | CEBPA | 2591 |
| 0.01646049 | 2.237066186 | down | NM_000632 | ITGAM | 4742 |
| 0.006394759 | 2.236249462 | down | NM_206940 | MS4A7 | 2841 |
| 0.037017989 | 2.234947331 | down | NM_174878 | CLRN1 | 2359 |
| 0.009999395 | 2.231576945 | down | NM_006332 | IFI30 | 1051 |
| 0.000823076 | 2.222577681 | down | NM_002178 | IGFBP6 | 980 |
| 0.003113613 | 2.217355727 | down | NM_024872 | DOK3 | 1747 |
| 0.02190811 | 2.210011639 | down | NM_001123041 | CCR2 | 2689 |
| 0.000780859 | 2.209024593 | down | NM_004355 | CD74 | 1327 |
| 0.001681859 | 2.205792323 | down | NM_006120 | HLA-DMA | 1122 |
| 0.014610043 | 2.201804316 | down | NM_030769 | NPL | 1552 |
| 0.020554423 | 2.189020733 | down | NM_012252 | TFEC | 6631 |
| 0.018458005 | 2.172072162 | down | NM_002030 | FPR3 | 2517 |
| 0.026234618 | 2.166258052 | down | NM_001223 | CASP1 | 1301 |
| 0.009652746 | 2.161173213 | down | NM_005755 | EBI3 | 1149 |
| 0.007724235 | 2.157711462 | down | NM_002984 | CCL4 | 667 |
| 0.004486399 | 2.156597604 | down | NM_033554 | HLA-DPA1 | 1157 |
| 0.004786303 | 2.149074595 | down | NM_003006 | SELPLG | 2550 |
| 0.009710544 | 2.147445277 | down | NM_000361 | THBD | 4048 |
| 0.010302484 | 2.144123455 | down | NM_173203 | IL1F7 | 604 |
| 0.040931774 | 2.132512389 | down | NM_022304 | HRH2 | 3095 |
| 0.003001177 | 2.129591222 | down | NM_002118 | HLA-DMB | 1358 |
| 0.000265385 | 2.127388796 | down | NM_001002235 | SERPINA1 | 3199 |
| 0.001238134 | 2.12387768 | down | NM_003955 | SOCS3 | 2746 |
| 0.003340871 | 2.122142558 | down | NM_006682 | FGL2 | 4268 |
| 0.031459747 | 2.120750014 | down | NM_174896 | C1orf162 | 946 |
| 0.047261103 | 2.104905865 | down | NM_024575 | TNFAIP8L2 | 1248 |
| 0.012631017 | 2.10357367 | down | NM_006678 | CD300C | 1548 |
| 0.0121118 | 2.103541466 | down | NM_007074 | CORO1A | 1620 |
| 0.000373903 | 2.100567528 | down | NM_001710 | CFB | 2646 |
| 0.034340999 | 2.099294016 | down | NM_012387 | PADI4 | 2265 |
| 0.020294671 | 2.097731219 | down | NM_003851 | CREG1 | 2048 |
| 0.004856569 | 2.096422118 | down | NM_001671 | ASGR1 | 1514 |
| 0.033775783 | 2.095346405 | down | NM_002124 | HLA-DRB1 | 1229 |
| 0.011791212 | 2.093824642 | down | NM_207322 | C2CD4A | 3461 |
| 0.004923551 | 2.093717644 | down | NM_004244 | CD163 | 4231 |
| 0.016834584 | 2.091206608 | down | NM_022349 | MS4A6A | 1432 |
| 0.016846485 | 2.088901496 | down | NM_033293 | CASP1 | 1085 |
| 0.042764029 | 2.084510935 | down | NM_001145721 | HOMER3 | 1428 |
| 0.028637466 | 2.067335306 | down | NM_000146 | FTL | 889 |
| 0.017293826 | 2.063570176 | down | NM_000477 | ALB | 2264 |
| 0.007447961 | 2.060771056 | down | NM_004585 | RARRES3 | 779 |
| 0.037754764 | 2.052810304 | down | NM_005204 | MAP3K8 | 3096 |
| 0.001280212 | 2.052347798 | down | NM_001004340 | FCGR1B | 863 |
| 0.048118615 | 2.048092497 | down | NM_001128850 | RRAD | 1476 |
| 0.004680888 | 2.0271583 | down | NM_032152 | PRAM1 | 2189 |
| 0.003029762 | 2.026129928 | down | NM_001131027 | PDLIM4 | 2189 |
| 0.002399074 | 2.015542409 | down | NM_172247 | CSF2RA | 1590 |
| 0.005726326 | 2.011492132 | down | NM_014485 | HPGDS | 1615 |
|  |  |  |  |  |  |

There are upregulation of 66 mRNAs and downregulation of 156 mRNAs.

**Table S8** Clinical characteristics in 109 patients with pancreatic cancer

| **Characteristics** | | **Frenquence** | **Percentage (%)** |
| --- | --- | --- | --- |
| Gender | Femal | 52 | 47.7 |
|  | Male | 57 | 52.3 |
| Ages | ≤60 | 40 | 36.7 |
|  | ＞60 | 69 | 63.3 |
| Location of tumor | Head-neck | 67 | 61.5 |
|  | Body-tail | 42 | 38.5 |
| Tumor grade | I | 4 | 3.7 |
|  | II | 35 | 32.1 |
|  | III | 70 | 64.2 |
| Tumor size | T1 | 6 | 5.5 |
|  | T2 | 27 | 24.8 |
|  | T3 | 76 | 69.7 |
| Lymph node metastasis | No | 67 | 61.5 |
|  | Yes | 45 | 38.5 |
| Perineural invasion | No | 36 | 33.0 |
|  | Yes | 73 | 67.0 |
| Vascular invasion | No | 81 | 74.3 |
|  | Yes | 28 | 25.7 |
| Lymphovascular invasion | No | 88 | 80.7 |
|  | Yes | 21 | 19.3 |
| TNM stage | I | 25 | 22.9 |
|  | II | 84 | 77.1 |

**Table S9** Correlation between DKFZp434J0226 and clinical characteristics

|  |  | **Expression of DKFZp434J0226** | | **χ2** | ***P* values** |
| --- | --- | --- | --- | --- | --- |
|  |  | **Low level** | **High level** |  |  |
| Gender | Femal | 23 | 29 | 1.543 | 0.214 |
|  | Male | 32 | 25 |  |  |
| Ages | ≤60 | 23 | 17 | 1.253 | 0.263 |
|  | ＞60 | 32 | 37 |  |  |
| Location of tumor | Head-neck | 33 | 34 | 0.101 | 0.751 |
|  | Body-tail | 22 | 20 |  |  |
| Tumor grade | I | 4 | 0 | 8.363 | **0.015** |
|  | II | 22 | 13 |  |  |
|  | III | 29 | 41 |  |  |
| Tumor size | T1 | 4 | 2 | 1.201 | 0.548 |
|  | T2 | 15 | 12 |  |  |
|  | T3 | 36 | 40 |  |  |
| Lymph node metastasis | No | 36 | 25 | 0.745 | 0.388 |
|  | Yes | 19 | 23 |  |  |
| Perineural invasion | No | 28 | 8 | 16.048 | **0.001** |
|  | Yes | 27 | 46 |  |  |
| Vascular invasion | No | 40 | 41 | 0.146 | 0.702 |
|  | Yes | 15 | 13 |  |  |
| Lymphovascular invasion | No | 44 | 44 | 0.038 | 0.854 |
|  | Yes | 11 | 10 |  |  |
| TNM stage | I | 17 | 8 | 3.993 | **0.046** |
|  | II | 38 | 46 |  |  |

Bold *P* values less than 0.05 indicates statistical significance.

**Table S10** Univariate analysis of factors associated with survival and recurrence

| **Variables** | **OS** | | **TTP** | |
| --- | --- | --- | --- | --- |
|  | **Hazard ratio(95％CI)** | ***P*** | **Hazard ratio(95％CI)** | ***P*** |
| Gender  (female vs. male) | 0.783  (0.481-1.276) | 0.327 | 0.768 (0.484-1.220) | 0.264 |
| Age, years  (≤60 vs. >60) | 1.541  (0.901-2.641) | 0.114 | 1.840  (1.086-3.119) | **0.023** |
| Tumor location  (head-neck vs. body-tail) | 0.910  (0.549-1.507) | 0.714 | 0.941  (0.567-1.473) | 0.711 |
| Tumor grade  (I vs. II vs. III) | 1.917  (1.170-3.140) | **0.010** | 1.951  (1.229-3.096) | **0.005** |
| Tumor size  (T1 vs. T2 vs. T3) | 2.756  (1.533-4.953) | **0.001** | 2.750  (1.598-4.734) | **<0.001** |
| Lymph node metastasis (negative vs. positive) | 1.665  (1.005-2.759) | **0.048** | 1.876  (1.161-3.034) | **0.010** |
| Perineural invasion  (negative vs. positive) | 1.869  (1.064-3.286) | **0.030** | 1.799  (1.063-3.045) | **0.029** |
| Vascular invasion  (negative vs. positive) | 2.020  (1.231-3.315) | **0.005** | 1.892  (1.169-3.063) | **0.009** |
| Lymphovascular invasion  (negative vs. positive) | 1.138  (0.618-2.094) | 0.678 | 1.351  (0.775-2.357) | 0.288 |
| TNM stage  (I vs.II) | 4.484  (1.930-10.421) | **<0.001** | 4.621  (2.104-10.149) | **<0.001** |
| DKFZp434J0226 Expression (low vs. high) | 2.148  (1.312-3.517) | **0.002** | 2.029  (1.271-3.239) | **0.003** |
| Cox proportional hazards regression model was used in univariate analysis. Bold *P* values less than 0.05 indicate statistical significance. **Abbreviation:** OS; overall survival. TTP; time to progression. | | | | |
