# Supplementary figures and images for: The long non-coding RNA DKFZp434J0226 regulates the alternative splicing process through phosphorylation of SF3B6 in PDAC
Source: Mol Med. 2021 Aug 28;27:95. doi: 10.1186/s10020-021-00347-7 (PMC8411526; doi:10.1186/s10020-021-00347-7)

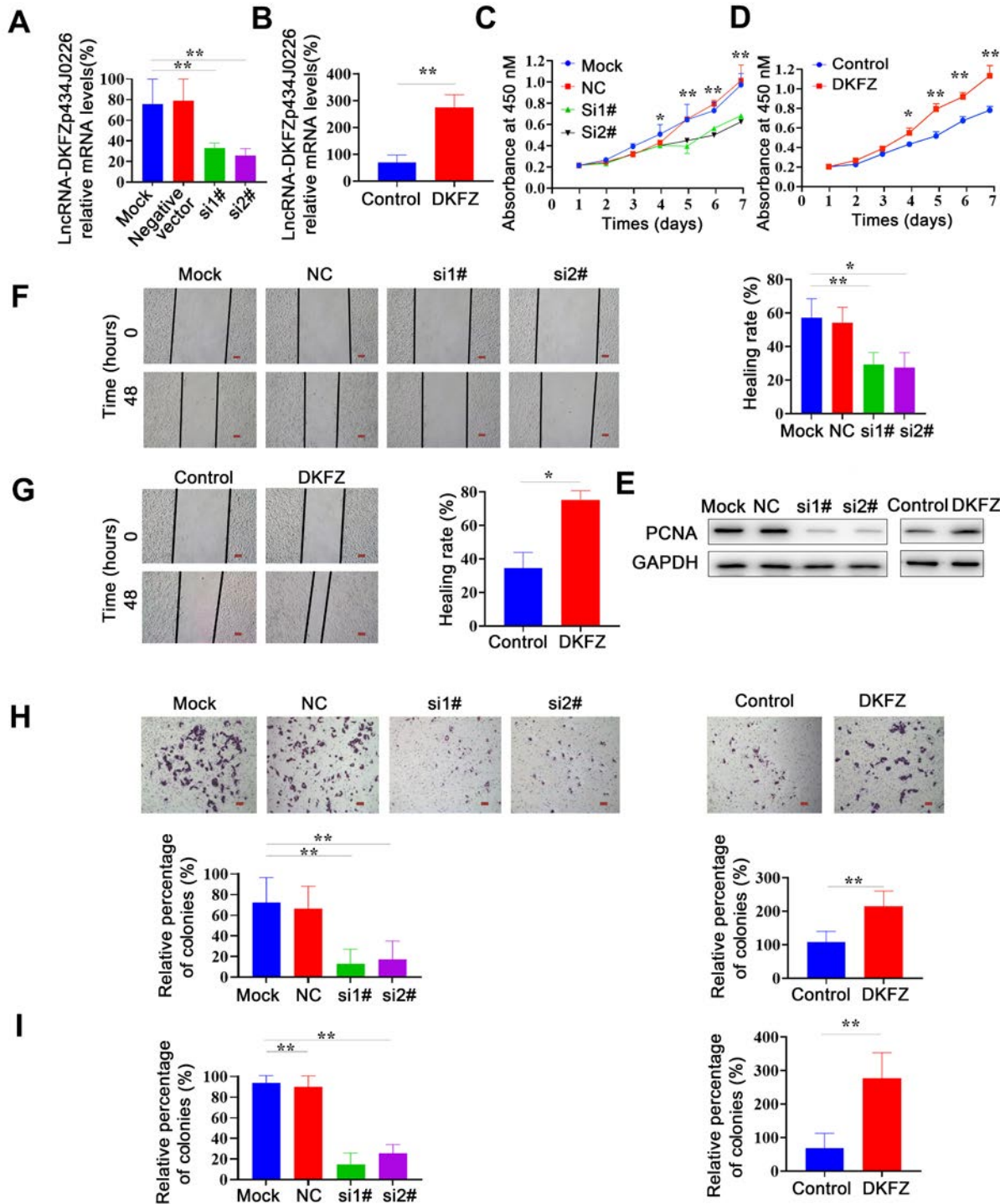

**A**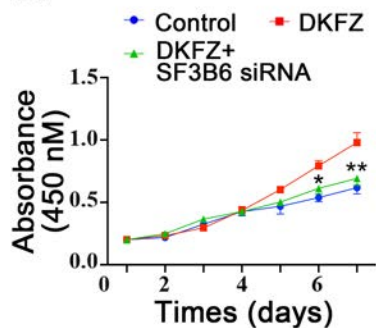**B**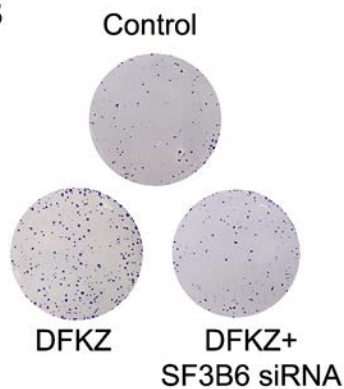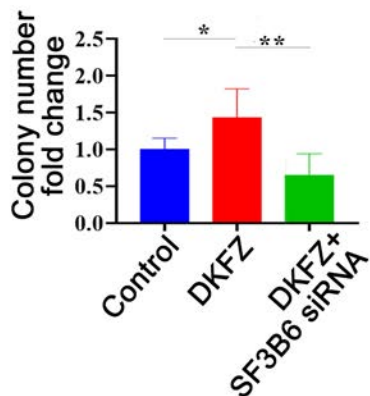**C**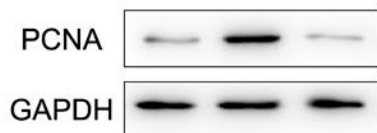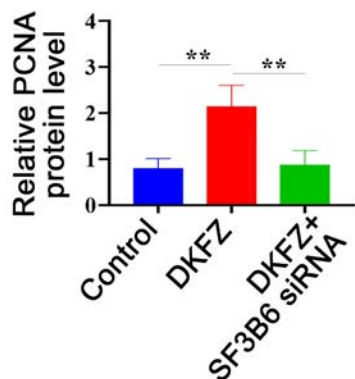**E**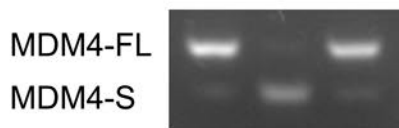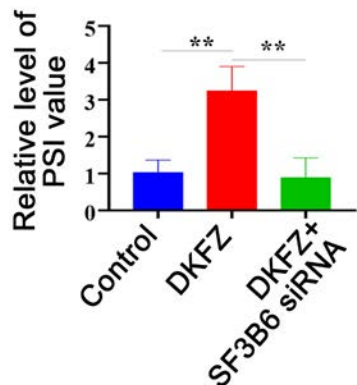**D**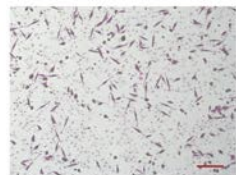

Control

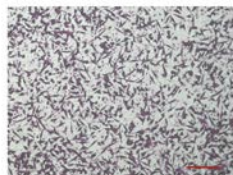

DKFZ

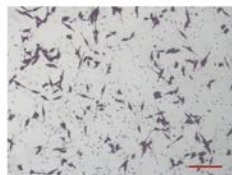DKFZ+  
SF3B6 siRNA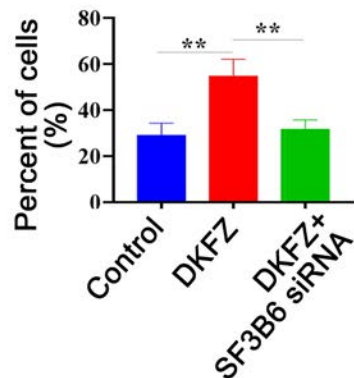

Supplement: Supplementary file 2 — Additional file 2: Figure S1.DKFZp434J0226 (DKFZ) promotes PDAC cell proliferation and migration. (A, B) Verification of DKFZ knockdown (A) and DKFZ overexpression (B) efficiency in PANC-1 and CFPAC-1 cells (n = 3). Data are shown as mean ± SD; *P < 0.05, **P < 0.01. (C) Cell growth curves of lipofectamine-treated PANC-1 (Mock) cells, negative siRNA-transfected PANC-1 (NC) cells, and two DKFZ siRNA-transfected PANC-1 (si1 and si2; n = 4). Data are shown as mean ± SD; *P < 0.05 vs. Mock and NC; **P < 0.01 vs. Mock and NC. (D) Cell growth curves of control lentivirus-infected CFPAC-1 cells (control) and DKFZ lentivirus-infected CFPAC-1 cells (DKFZ) (n = 4). Data are shown as mean ± SD; *P < 0.05 vs. Mock and NC; **P < 0.01 vs. Mock and NC. (E) Western blotting analysis of PCNA expression in the indicated PDAC cells. (F-I) Wound healing (F, G), migration (H) assays and soft agar colony formation assay (I) using the indicated PDAC cells. (n = 4). (F-I) Data are shown as mean ± SD; *P < 0.05, **P < 0.01. Scale bars refer to 100 μm. Figure S2. (A–C) Cell growth curves assay (A), soft agar colony formation assay (B), and western blotting of PCNA (C) demonstrating that SF3B6 knockdown rescues DKFZ-induced cell proliferation in CFPAC-1 cells. (D) Migration assay demonstrating that SF3B6 knockdown rescues DKFZ-induced cell migration in CFPAC-1 cells. (E) RT-qPCR demonstrating that SF3B6 knockdown rescues DKFZ-induced alternative splicing of MDM-4. (A) Data are shown as mean ± SD; *P < 0.05 vs. DKFZ; **P < 0.01 vs. DKFZ. (B–E) Data are shown as mean ± SD; *P < 0.05, **P < 0.01. Scale bars refer to 100 μm. [file 10020_2021_347_MOESM2_ESM.pdf]
